# Synergy and oxygen adaptation for development of next-generation probiotics

**DOI:** 10.1038/s41586-023-06378-w

**Published:** 2023-08-02

**Authors:** Muhammad Tanweer Khan, Chinmay Dwibedi, Daniel Sundh, Meenakshi Pradhan, Jamie D. Kraft, Robert Caesar, Valentina Tremaroli, Mattias Lorentzon, Fredrik Bäckhed

**Affiliations:** 1 Wallenberg Laboratory, Department of Molecular and Clinical Medicine, Institute of Medicine, University of Gothenburg, Gothenburg, Sweden; 2 Metabogen, Mölndal, Sweden; 3 Institute of Neuroscience and Physiology, University of Gothenburg, Gothenburg, Sweden; 4 Sahlgrenska Osteoporosis Centre, Department of Internal Medicine and Clinical Nutrition, Institute of Medicine, University of Gothenburg, Gothenburg, Sweden; 5 Region Västra Götaland, Geriatric Medicine Clinic, Sahlgrenska University Hospital Mölndal, Mölndal, Sweden; 6 Mary MacKillop Institute for Health Research, Australian Catholic University, Melbourne, Victoria Australia; 7 Department of Clinical Physiology, Sahlgrenska University Hospital, Gothenburg, Sweden; 8 Novo Nordisk Foundation Center for Basic Metabolic Research, Faculty of Health Sciences, University of Copenhagen, Copenhagen, Denmark

**Keywords:** Microbiology, Bacteriology

## Abstract

The human gut microbiota has gained interest as an environmental factor that may contribute to health or disease^1^. The development of next-generation probiotics is a promising strategy to modulate the gut microbiota and improve human health; however, several key candidate next-generation probiotics are strictly anaerobic^2^ and may require synergy with other bacteria for optimal growth. *Faecalibacterium prausnitzii* is a highly prevalent and abundant human gut bacterium associated with human health, but it has not yet been developed into probiotic formulations^2^. Here we describe the co-isolation of *F. prausnitzii* and *Desulfovibrio piger*, a sulfate-reducing bacterium, and their cross-feeding for growth and butyrate production. To produce a next-generation probiotic formulation, we adapted *F. prausnitzii* to tolerate oxygen exposure, and, in proof-of-concept studies, we demonstrate that the symbiotic product is tolerated by mice and humans (ClinicalTrials.gov identifier: NCT03728868) and is detected in the human gut in a subset of study participants. Our study describes a technology for the production of next-generation probiotics based on the adaptation of strictly anaerobic bacteria to tolerate oxygen exposures without a reduction in potential beneficial properties. Our technology may be used for the development of other strictly anaerobic strains as next-generation probiotics.

## Abstract (web summary)

The anaerobic gut bacterium *Faecalibacterium prausnitzii* was isolated and adapted for oxygen tolerance to develop a next-generation probiotic for the treatment of conditions such as inflammatory bowel disease and type 2 diabetes.

## Main

The adult human gut microbiota consists of at least as many bacterial cells as our total number of somatic and germ cells^3^ and their collective genomes (microbiome) contain more than 500-fold more genes than our human genome^4^. Comparative metagenomics has revealed that, compared with the microbiome of patients with type 2 diabetes^5–7^, hyperlipidaemia^8^ and inflammatory bowel disease^9,10^, a healthy microbiome is frequently associated with an increased microbial diversity and an increased abundance of butyrate-producing bacteria, such as *Faecalibacterium prausnitzii*. In particular, *F. prausnitzii* is a keystone species whose abundance varies with age and lifestyle, and is relatively depleted in the gut microbiota of people living in the Western world^11^.

Human gut microorganisms do not act in isolation but form complex ecological interactions that are important for intestinal homeostasis. One key attribute of the gut microbiota is the fermentation of carbohydrates to short-chain fatty acids (SCFAs), including butyrate, which is associated with several host benefits^12^. Fermentation is the major energy-generating process utilized by gut microorganisms, and the removal of fermentation electron sink by-products such as lactate and hydrogen is essential to maintain fermentative processes^13^. Accordingly, hydrogen scavengers such as methanogens and sulfate-reducing bacteria are important for establishing gut metabolic networks^14^.

Here we report the isolation of a novel strain of *F. prausnitzii* in co-culture with a novel strain of the sulfate reducer *Desulfovibrio piger*. We describe the development of a technology for the production of *F. prausnitzii* as a next-generation probiotic and we assess its safety for human consumption.

**Fig. 1:**
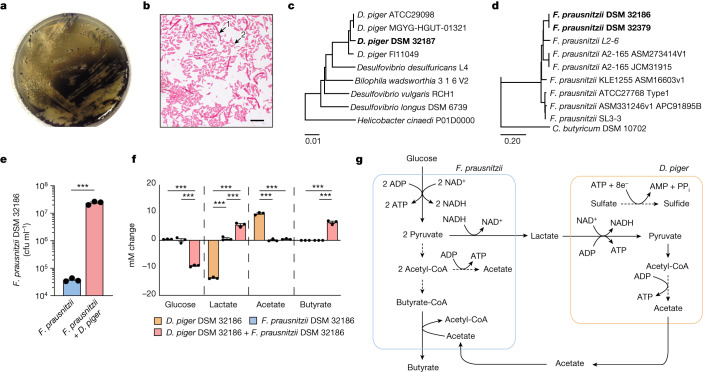
Co-isolation and cross-feeding of *F. prausnitzii* and *D. piger* in vitro. **a**, Co-culture of *F. prausnitzii* DSM 32186 and *D. piger* DSM 32187 on PGM plates without supplementation of glucose or acetate. **b**, Gram staining of colonies from isolation of *F. prausnitzii* DSM 32186 and *D. piger* DSM 32187. Arrows indicate *F. prausnitzii* (long fusiform rods) (1) and *D. piger* (short rods) (2). Scale bar, 10 μm. **c**, Dendrogram illustrating the relationship between *D. piger* DSM 32187 and related genomes. **d**, Dendrogram illustrating the relationship between *F. prausnitzii* DSM 32186 and related genomes. **e**, The number of colony-forming units of *F. prausnitzii* DSM 32186 in monoculture and in co-culture with *D. piger* DSM 32187 under anaerobic conditions in mPGM (PGM containing 25 mM of glucose) for 24 h. *P* = 0.0003. **f**, Metabolite profiles of *F. prausnitzii* DSM 32186 and *D. piger* DSM 32187 cultivated as monocultures or co-culture under anaerobic conditions in mPGM medium for 24 h. Glucose: *P* = 0.0000031 (*F. prausnitzii* + *D. piger* versus *D. piger*), *P* = 0.0000038 (*F. prausnitzii* + *D. piger* versus *F. prausnitzii*); lactate: *P* = 0.0000001 (*F. prausnitzii* + *D. piger* versus *D. piger*), *P* = 0.0000005 (*F. prausnitzii* versus *D. piger*), *P* = 0.00014 (*F. prausnitzii* + *D. piger* versus *F. prausnitzii*); acetate: *P* = 0.0000004 (*F. prausnitzii* + *D. piger* versus *D. piger*), *P* = 0.0000003 (*F. prausnitzii* versus *D. piger*); butyrate: *P* = 0.000001(*F. prausnitzii* + *D. piger* versus *D. piger*), *P* = 0.0000031 (*F. prausnitzii* + *D. piger* versus *F. prausnitzii*). ‘mM change’ on the *y* axis indicates the difference in concentration from the inoculated medium at baseline. **g**, Schematic of the suggested cross-feeding between *F. prausnitzii* and *D. piger* as co-culture in mPGM. *n* = 3 independent experiments, ***P < 0.001 determined by two-tailed *t*-test (**e**) or one-way ANOVA (**f**). Data are mean ± s.e.m.

To preserve microorganism–microorganism interactions and isolate bacteria that are able to serve as an electron sink and remove lactate, we plated faecal material from a healthy individual directly on agar plates of Postgate’s medium (PGM) in anaerobic conditions (Methods). Under these conditions, we isolated a strain of *F. prausnitzii* (DSM32186) that grew in PGM as co-culture with a strain of *D. piger* (DSM 32187) (Fig. 1a,b). *D. piger* are obligate anaerobic, non-fermenting, Gram-negative bacilli^15^ and prevalent sulfate reducers in the human gut^16^, do not occur in isolation outside the intestine, and are thus considered gut-specific commensals^17^.

**Extended Data Fig. 1:**
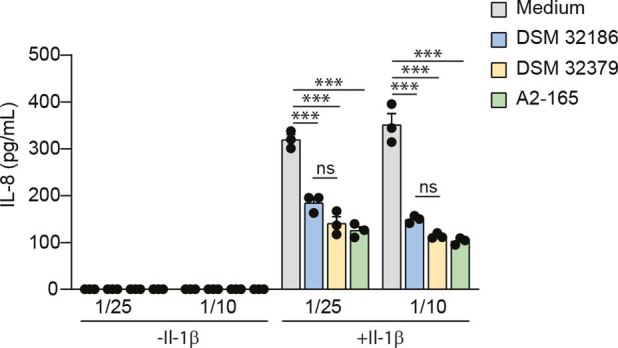
Anti-inflammatory properties of parental *F. prausnitzii* DSM 32186 and oxygen tolerant DSM 32379. Modulation of IL-1β-induced IL-8 secretion by Caco-2 cells in contact with *F. prausnitzii* supernatants (strain A2-165 (DSM 17677) included as reference). Cells were exposed to bacterial supernatant with or without 4 ng/mL IL-1β for 6 hr. The horizontal axis group labels indicate dilution of filtered medium from the *F. prausnitzii* culture. p = 0.025 (DSM 32186 *vs*. A2-165, 1/25), p = 0.0000089 (LYBHI *vs*. A2-165, 1/25), p = 0.00013 (LYBHI *vs*. DSM32186, 1/25), p = 0.000016 (LYBHI *vs*. DSM32379, 1/25), p = 0.0000028 (LYBHI *vs*. A2-165, 1/10), p = 0.000013 (LYBHI *vs*. DSM32186, 1/10), p = 0.0000039 (LYBHI *vs*. DSM32379, 1/10). *n* = 3, ***p < 0.001, *p < 0.05 as determined by One-way ANOVA. The results were repeated in two independent experiments. Data are presented as mean ± s.e.m.

To confirm the identity of the isolates, we sequenced their genomes, and observed that *D. piger* DSM 32187 clustered with other sequenced *D. piger* strains (Fig. 1c), whereas *F. prausnitzii* DSM 32186 clustered with *F. prausnitzii* phylogroup group II, including strain A2-165 (Fig. 1d). The genomic analyses showed that both isolates formed specific clades in the phylogenetic tree, indicating that they represented previously uncharacterized strains. Since A2-165 has anti-inflammatory properties^18^ and can interact at the mucosal interface^19^, we verified the probiotic potential of DSM 32186 by assessing its immunomodulatory properties compared with A2-165. We observed similar reductions of interleukin-1β (IL-1β)-induced secretion of IL-8 when supernatants from the different *F. prausnitzii* strains were applied to Caco-2 cells (Extended Data Fig. 1), confirming the relatedness to A2-165 at the phenotypic level.

We hypothesized that the co-isolation of *F. prausnitzii* and *D. piger* in PGM and their putative symbiotic relationship resulted from complementary metabolic requirements. To verify this hypothesis, we co-cultured the two strains in a modified PGM medium that contained glucose to support *F. prausnitzii*, and observed that the growth of *F. prausnitzii* was significantly increased in co-culture compared with monoculture in the same medium (Fig. 1e). Analysis of metabolites in conditioned medium after 24 h of growth revealed that, as expected, monocultures of *D. piger* consumed lactate and produced acetate without consuming glucose, whereas monocultures of *F. prausnitzii* produced very small amounts of lactate (Fig. 1f). However, co-cultures of *F. prausnitzii* with *D. piger* promoted fermentation of glucose and production of lactate and butyrate (Fig. 1f). *F. prausnitzii* produces butyrate through the butyryl-coenzyme A (CoA):acetate CoA-transferase pathway and accordingly, acetate did not accumulate in the medium in co-cultures (Fig. 1f), as acetate is required for butyrate production^20,21^. Thus, our findings suggest that *D. piger* functioned as an electron sink in the co-culture in PGM as it consumed lactate; *D. piger* generated acetate that was used by *F. prausnitzii* for growth and butyrate production (Fig. 1g).

**Fig. 2:**
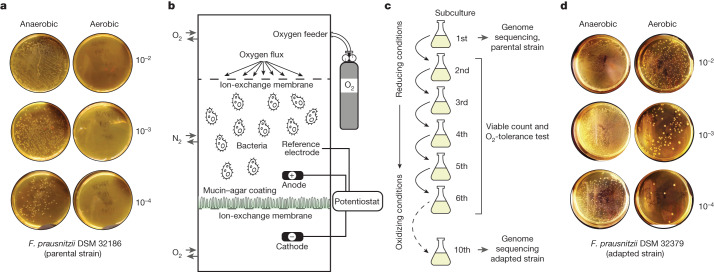
Development of oxygen tolerance in *F. prausnitzii* by stepwise adaptation. **a**, Oxygen tolerance of *F. prausnitzii* DSM 32186 in YCFAG medium after exposure to ambient air for 20 min compared with control plates incubated in anaerobiosis. **b**, Schematic presentation of the modified simulated human redox intestinal model (m-SHIRM) bioreactor. **c**, The oxidative adaptation strategy used to develop oxygen-tolerant strains of *F. prausnitzii*. **d**, Oxygen tolerance of the oxygen-adapted *F. prausnitzii* DSM 32379 developed from the parental strain DSM 32186. Oxygen exposure performed as in Fig. 2a. Numbers to the right of agar plates in **a**,**d** indicate dilution.

**Extended Data Fig. 2:**
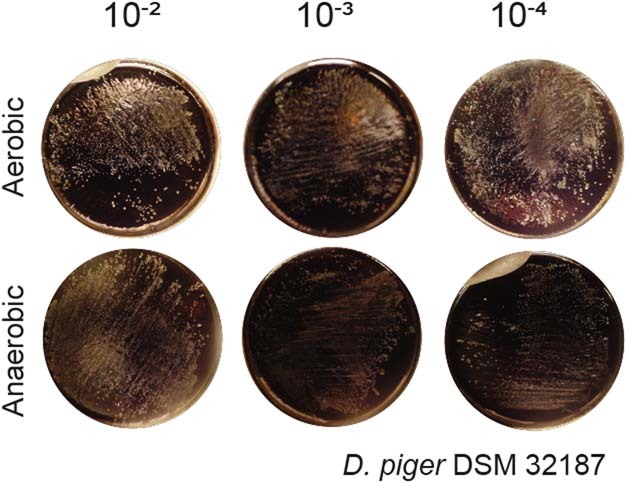
Oxygen tolerance profile of *D. piger* DSM 32187 isolated as co-culture with *F. prausnitzii* DSM 32186. Serial dilutions were inoculated on PGM plates that were exposed to ambient air for 20 min, and compared with control plates incubated in a Coy anaerobic chamber. Numbers beside agar plates indicate dilution.

Product development for next-generation probiotics is challenging, owing to the oxygen sensitivity of human gut bacteria. This process also requires the optimization of growth conditions to obtain sufficient biomass yields, as well as novel strategies to preserve the viability of the final product^2^. We used the co-culture of *F. prausnitzii* with *D. piger* to increase growth yields in fermentations as shown in Fig. 1e. However, as *F. prausnitzii* is extremely sensitive to oxygen in ambient air^21^, no colonies were recovered when *F. prausnitzii* DSM 32186 was exposed to air for 20 min (Fig. 2a). By contrast, *D. piger* is relatively oxygen-tolerant under the same conditions (Extended Data Fig. 2).

**Extended Data Fig. 3:**
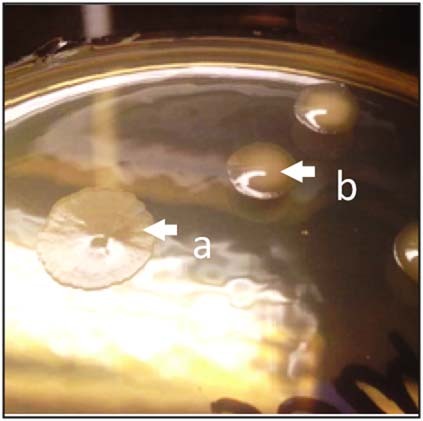
Morphotype of the oxygen adapted *F. prausnitzii* DSM 32379. Morphotype of *F. prausnitzii* DSM 32379 (arrow b) isolated from m-SHIRM bioreactor during the 10^th^ subculture step. The parental strain *F. prausnitzii* DSM 32186 is shown as reference (arrow a).

**Extended Data Fig. 4:**
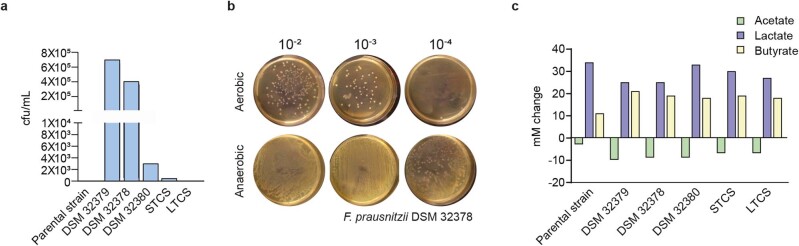
Oxygen tolerance and fermentation profiles of *F. prausnitzii* oxygen tolerant variants. **a**, Quantification of oxygen tolerance of parental strain and oxygen adapted variants in YCFAG. **b**, Oxygen tolerance of the oxygen adapted *F. prausnitzii* DSM 32378. Serial dilutions (shown on top) were inoculated on YCFAG plates and exposed to ambient air for 20 min, and compared with control plates incubated in anaerobiosis. **c**, Metabolite profile of parental DSM 32186 and oxygen adapted variants cultured in YCFAG medium. mM change on the y-axis indicates difference from inoculated medium at baseline.

**Extended Data Fig. 5:**
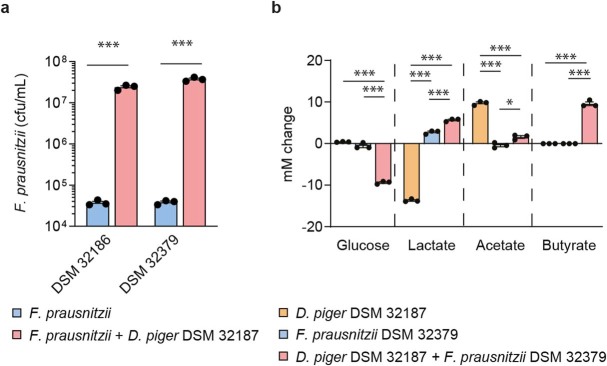
Growth and cross-feeding of parental *F. prausnitzii* DSM 32186 or oxygen tolerant DSM 32379 with *D. piger* DSM 32187. **a**, Colony forming units of parental *F. prausnitzii* DSM 32186 and oxygen tolerant DSM 32379 in mono-culture or co-culture with *D. piger* DSM 32187 in modified Postgate’s medium (i.e., Postgate’s medium containing 25 mM of glucose; mPGM) after 24 h of growth. p = 0.00000073 (DSM 32186), p = 0.00000022 (DSM 32379). **b**, Metabolite profiles of *F. prausnitzii* DSM 32379 and *D. piger* DSM 32187 as mono-cultures or co-culture in mPGM after 24 h of growth. Glucose: p = 0.0000008 (*F. prausnitzii* + *D. piger vs. D. piger*), p = 0.0000012 (*F. prausnitzii* + *D. piger vs. F. prausnitzii*); lactate: p = 0.0000000001 (*F. prausnitzii* + *D. piger vs. D. piger*), p = 0.0000000013 (*F. prausnitzii vs. D. piger*), p = 0.00018 (*F. prausnitzii* + *D. piger vs. F. prausnitzii*); acetate: p = 0.000005 (*F. prausnitzii* + *D. piger vs. D. piger*), p = 0.0000015 (*F. prausnitzii vs. D. piger*), p = 0.012 (*F. prausnitzii* + *D. piger vs. F. prausnitzii*); butyrate: p = 0.0000007 (*F. prausnitzii* + *D. piger vs. D. piger*), p = 0.0000007 (*F. prausnitzii* + *D. piger vs. F. prausnitzii*); mM change on the y-axis indicates difference from inoculated medium at baseline. *n* = 3 independent experiments. ***p < 0.001, *p < 0.05 as determined by two-tailed t-test (**a**) or one-way ANOVA (**b**) Data are presented as mean ± s.e.m.

As shown previously, the shelf life of formulations containing *F. prausnitzii* can be increased by using antioxidants such as cysteine, although with limited applicability for industrial-scale production, as viability is lost after a 24 h exposure to ambient air^22^. To improve *F. prausnitzii* oxygen tolerance we used an adaption strategy using an m-SHIRM bioreactor^23^, in which DSM 32186 was exposed to oxidized conditions for ten consecutive subculture steps, with decreasing concentrations of cysteine and increasing anodic potential (Fig. 2b,c). At each step, a sample from the previous step was inoculated on yeast extract casitone fatty acid glucose (YCFAG) growth medium anaerobically. Visual inspection of plates revealed distinct colony morphotypes at the sixth and tenth subcultures (Extended Data Fig. 3), and five colony morphotypes were selected for characterization of oxygen tolerance after taxonomic confirmation as *F. prausnitzii* by 16S rRNA gene sequencing. Increased oxygen tolerance was clearly observed for two morphotypes (Extended Data Fig. 4a)—DSM 32378 (Extended Data Fig. 4b) and DSM 32379 (Fig. 2d)—and occurred without the loss of butyrate production capacity (Extended Data Fig. 4c). DSM 32379 had the highest increase of oxygen tolerance (Extended Data Fig. 4a) and was thus further selected for analysis of synergistic growth with *D. piger* DSM 32187, which was also not affected (Extended Data Fig. 5).

Therefore, as a result of oxygen tolerance and co-culture with *D. piger*, we were able to produce *F. prausnitzii* in sufficient amounts for administration to humans: oxygen-tolerant DSM 32379 could be freeze-dried, and met the 2-weeks adequate stability criteria at −20 °C to be developed into capsules with limited loss of viability (log_10_(colony-forming units (CFU) g^−1^) = 9.6 versus 9.5 before and after two weeks storage, respectively). By contrast, the parental strain DSM 32186 yielded lower biomass (log_10_(CFU g^−1^) = 8.5) and underwent a 97% loss of viability.

**Extended Data Fig. 6:**
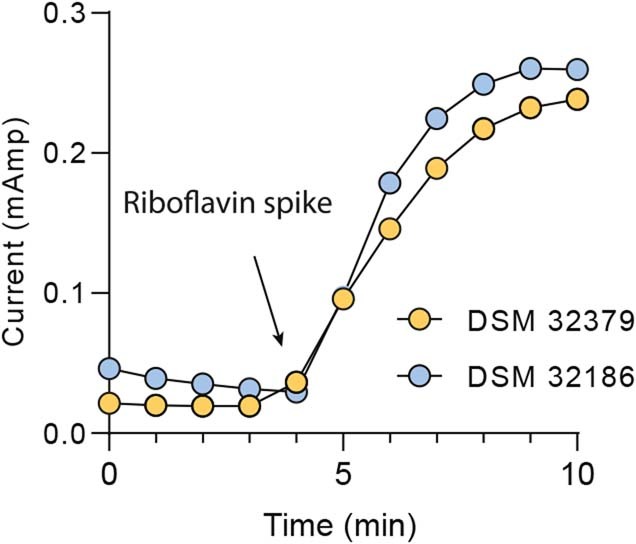
Riboflavin-mediated extracellular electron shuttling of parental *F. prausnitzii* DSM 32186 and oxygen tolerant DSM 32379. After energising the resting cells with glucose (100 mM), both parental *F. prausnitzii* DSM 32186 and oxygen tolerant DSM 32379 generated measurable current waves when riboflavin was spiked (200 µM), as previously described for A2-165^19^.

To describe possible molecular mechanisms leading to increased oxygen tolerance, we performed genome sequencing of DSM 32379, which revealed 15 variants in 10 loci affecting 23 nucleotides (0.0007% of the total genome). These variants occurred in genes with known and unknown functions (Extended Data Table 1), but since we were unable to genetically modify DSM 32186 using molecular biology approaches, we could not verify their role for development of oxygen tolerance and thus the molecular mechanisms remain unknown. However, oxygen tolerance in DSM 32379 did not alter immune-modulatory properties (Extended Data Fig. 1) and DSM 32379 retained the ability to exploit a riboflavin-dependent extracellular electron shuttle (Extended Data Fig. 6) that we previously characterized in *F. prausnitzii* A2-165 (ref. ^19^). These results indicate that oxygen tolerance in DSM 32379 did not alter cellular physiology, metabolism and the potential to interact with the host at the mucosal interface, and this variant was selected for the production of an investigational product.

We next assessed the safety of the combined product by gavaging Swiss Webster male and female mice with a bacterial suspension containing *F. prausnitzii* DSM 32379 and *D. piger* DSM 32187. The mice received 10^10^ CFU per strain and dose, 5 times during the first week and then twice a week for 3 weeks, and we observed no adverse responses. The caecal levels of *F. prausnitzii* and *D. piger* were assessed by quantitative PCR (qPCR) but we did not observe increased levels for either species at the end of the study, perhaps owing to the mode and frequency of the administration and the colonic localization of *F. prausnitzii*, and/or host specificity.

To investigate tolerability of the bacteria, we recruited 50 healthy men and women aged 20–40 years for a randomized placebo-controlled study for supplementation of low (1 × 10^8^–5 × 10^8^ CFU per capsule) or high (1 × 10^9^–5 × 10^9^ CFU per capsule) doses of *F. prausnitzii* DSM 32379 and *D. piger* for 8 weeks compared with placebo (Supplementary Table 1). The groups had matching clinical features with the exceptions of age (higher in the low-dose group compared with placebo), alanine transaminase levels (lower in the low-dose group compared with placebo) and alkaline phosphatase levels (lower in the high-dose group compared with placebo). The investigational product was well tolerated, regardless of dose. No study participant discontinued the study owing to an adverse event (Supplementary Table 2) and there was no increased frequency of adverse events or gastrointestinal symptoms in the treatment groups (Supplementary Tables 3 and 4). There were no clinically relevant or statistically significant group-to-group differences in change between baseline and eight weeks in any blood biochemistry secondary end points (including renal function, blood cell count, liver enzymes, markers of inflammation, haemoglobin, glycosylated haemoglobin or fasting blood glucose; Supplementary Tables 5 and 6).

**Fig. 3:**
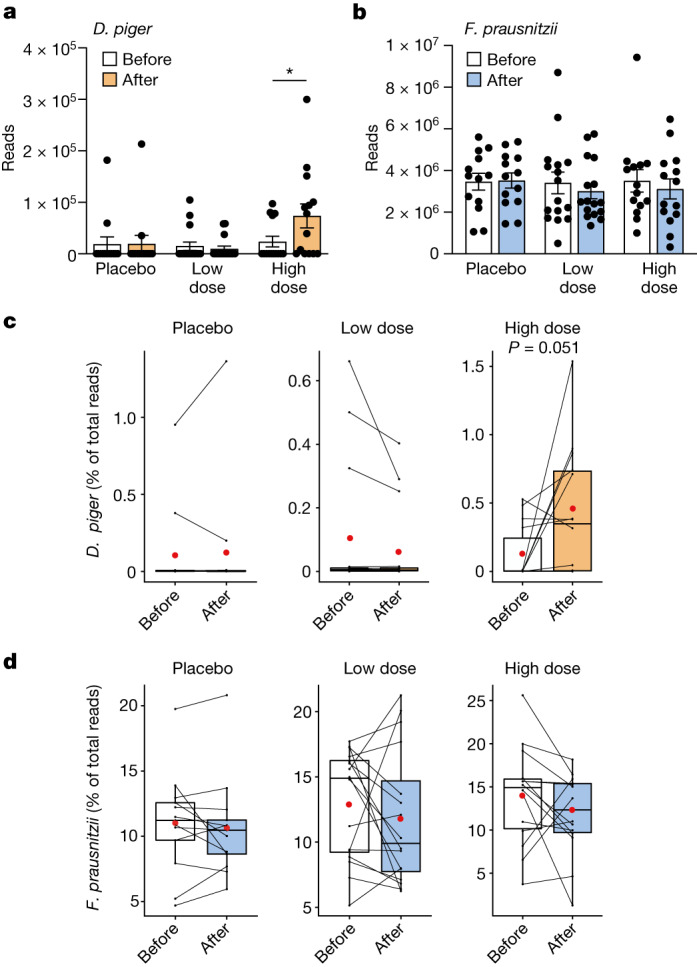
Abundance of *D. piger* and *F. prausnitzii* in healthy volunteers after administration of *D. piger* DSM 32187 and *F. prausnitzii* DSM 32379 for eight weeks. **a**,**b**, Total faecal counts of *D. piger* (*P* = 0.033) (high dose) (**a**) and *F. prausnitzii* (**b**) in metagenomic data before and after administration of the investigational product. **c**,**d**, The relative abundance of *D. piger* DSM 32187 (**c**) and *F. prausnitzii* DSM 32186 (**d**) in metagenomic data before and after administration. *n* = 13 (placebo), 16 (low dose) and 14 (high dose); **P < 0.01, two-sided Wilcoxon signed-rank test. Data are mean ± s.e.m.

**Extended Data Fig. 7:**
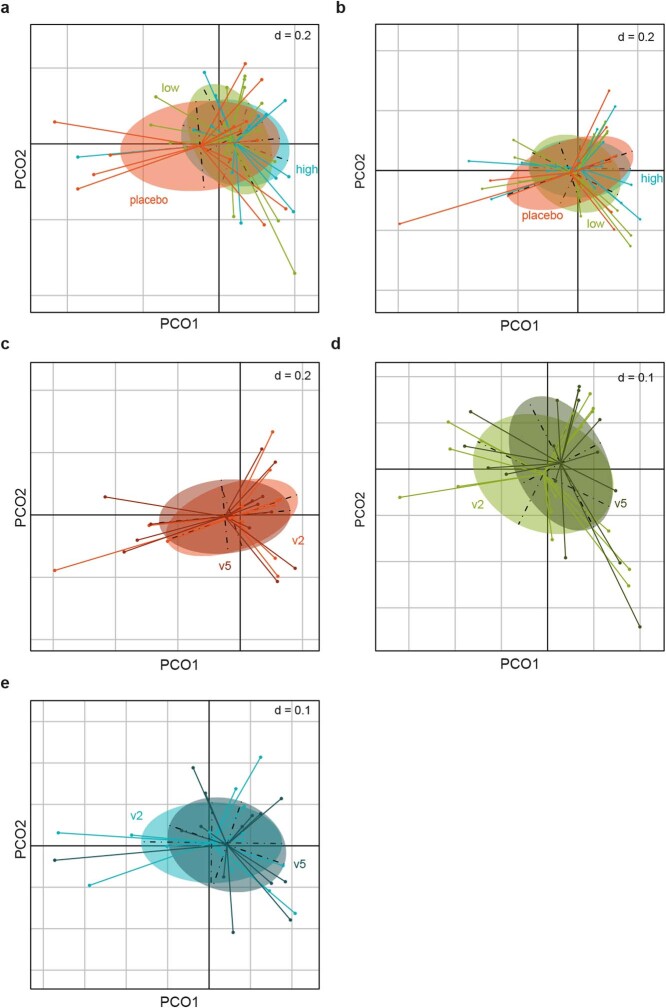
Analysis of overall fecal microbiota composition in the healthy volunteers after administration of *D. piger* DSM 32187 and *F. prausnitzii* DSM 32379 for 8 weeks. Principal coordinate analysis for the Bray-Curtis dissimilarity calculated based on species abundances for: **a**, fecal samples at baseline (r^2^ = 0.018; p = 1; adonis); **b**, fecal samples at the end of the administration (r^2^ = 0.040; p = 0.660; adonis); **c**, fecal samples from the placebo group at baseline (v2) and at the end of the administration (v5) (r^2^ = 0.009; p = 0.997; adonis); **d**, fecal samples from the low dose group at baseline (v2) and at the end of the administration (v5) (r^2^ = 0.014; p=0.971; adonis); **e**, fecal samples from the high dose group at baseline (v2) and at the end of the administration (v5) (r^2^ = 0.001; p = 0.996; adonis). Differences in composition were tested by a permutational multivariate ANOVA using the adonis2 function with 10,000 permutations in the vegan package in R (https://github.com/vegandevs/vegan/). Adjustments for multiple comparisons were made. The dots in the plots indicate fecal samples the 43 individuals with metagenomic data: placebo, n = 13; low dose, n = 16; high dose, n = 14. The analyses show no difference in microbiota composition among the groups at baseline or at the end of the administration (panels **a** and **b**, respectively), and no difference at the end of the administration compared to baseline in any of the groups (panels **c**, **d** and **e**).

**Extended Data Fig. 8:**
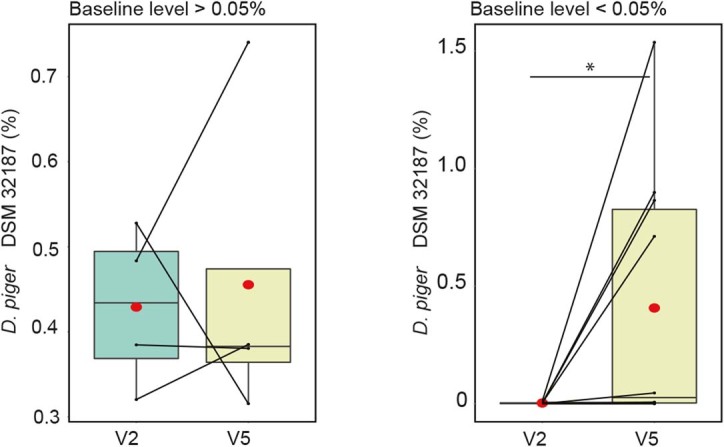
Genome capture for the abundance of *F. prausnitzii* DSM 32186 and *D. piger* DSM 32187 in fecal samples from the high dose group. Change in relative abundance in samples with high baseline *D. piger* DSM 32187 (> 0.05% of total microbiota; *n* = 4, p = 0.875, two-sided Wilcoxon signed-rank test) and low baseline *D.piger* DSM 32187 (<0.05% of total microbiota; n = 10, p = 0.042, Wilcoxon signed-rank test). v2, fecal samples at baseline; v5, fecal sample at the end of the administration. For box plots the middle line is the median, the lower and upper hinges are the first and third quartiles, the whiskers extend from the hinge to the largest and smallest value no further than 1.5 × the inter-quartile range (IQR).

**Extended Data Fig. 9:**
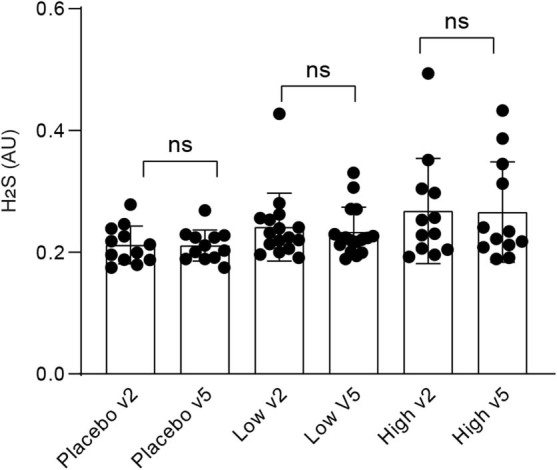
Measurement of hydrogen sulfide in fecal samples. Hydrogen sulfide was measured in fecal samples at baseline as well as at the end of the administration (40 individuals: placebo, n = 12; low dose, n = 16; high dose, n = 12; no sufficient material available for 1 individual in the placebo and 2 in the high dose groups). Two-sided Wilcoxon signed-rank test was performed. Data are presented as mean ± s.e.m.

We performed whole-genome metagenomic sequencing to evaluate possible effects on the human gut microbiota. There were no group-to-group differences in overall composition at baseline or at the end of the administration (Extended Data Fig. 7a,b), and no change was observed in any of the groups compared with baseline (Extended Data Fig. 7c–e). However, the number of sequencing reads for *D. piger* assessed at species level increased in the participants receiving the high dose (*P* < 0.01) (Fig. 3a), whereas reads for species-level *F. prausnitzii* did not change (Fig. 3b). We used genome capture to specifically quantify the strains and observed that both parental *F. prausnitzii* DSM 32186 and *D. piger* DSM 32187 were highly prevalent at baseline (43 out of 43 and 35 out of 43, respectively). In line with the species-level results, the proportions of *D. piger* DSM 32187 increased in the high-dose group (*P* = 0.051; Fig. 3c), and particularly in those with low relative abundance at baseline (less than 0.05%, *P* = 0.042; Extended Data Fig. 8), although remaining within the range observed for the placebo group (Fig. 3c) and for healthy Swedes sampled multiple times during one year^16^. We also found that the proportions of *F. prausnitzii* DSM 32186 did not change (Fig. 3d). Consistent with the lack of compositional shifts, the levels of faecal SCFAs did not change (Supplementary Table 7) and—despite the increase in *D. piger*—there was no change in faecal hydrogen sulfide levels (Extended Data Fig. 9). These results suggest that our probiotic formulation is safe for both the host (that is, lack of adverse events and gastrointestinal symptoms) and the gut microbiota (that is, lack of compositional and metabolic shifts).

The total species-level abundance of *F. prausnitzii* ranged between 3.4 and 25.9% (mean 13.2%), which was similar to the range observed for healthy individuals of similar age from the USA, as well as older healthy individuals from Sweden and the UK^16^, and higher than the species-level abundance in a recent large meta-analysis including 7,907 faecal metagenomes^11^ (mean 6.5 ± 7.6%). Thus, further increase of *F. prausnitzii* was probably limited by saturation of the niche. However, as we observed an increase for *D. piger* DSM 32187, on the basis of the model in Fig. 1g, we hypothesized that there was an effect on the abundance of other butyrate producers, such as those that require extracellular acetate for butyrate production similar to *F. prausnitzii*^20^. We quantified terminal genes for butyrate production and observed a significant positive correlation for the change in butyrate production potential and the change in *D. piger* DSM 32187 at the end of the administration compared with baseline in all individuals (Spearman’s rho = 0.48, *P* = 0.001) and in individuals who received a low or a high dose (Spearman’s rho = 0.49, *P* = 0.006), but not in the placebo group (Spearman rho = 0.39, *P* = 0.185). These results are in line with a recent study showing co-occurrence of *Desulfovibrio* with different butyrate producers, such as *Faecalibacterium, Roseburia*, *Oscillospira* and *Coprococcus*^24^, and suggest that our probiotic formulation might support the overall butyrate production potential in complex communities within the human gut. These results also stress the potential importance of baseline and/or specific gut microbiota configurations for microbiota-based treatment strategies^25–28^.

Finally, we attempted to detect *F. prausnitzii* DSM 32379 in faecal samples. qPCR assays targeting one or more of the genetic variants were not sufficiently discriminating, and genome capture methods could not be used to differentiate the oxygen-tolerant variant DSM 32379 from the parental DSM 32186, as these methods require less than 96% whole-genome similarity to identify unique strains^29^, and DSM 32379 is 99.9993% identical to DSM 32186. Thus, the counts of *F. prausnitzii* DSM 32186 detected by genome capture at the end of the administration probably represent the sum of the endogenous DSM 32186 and of the oxygen-tolerant DSM 32379 administered with the probiotic formulation. To track the possible presence of DSM 32379 in faecal samples, we detected the specific genetic variants in the metagenomic data (Methods). We observed few marker variants (for example, variant 7) in some participants at baseline or in the placebo group, in line with the observed genetic plasticity of *F. prausnitzii*^10^, and we found additional variants and/or several combinations at the end of the administration in a subset of the study participants in the low-dose and high-dose groups (Supplementary Table 8). The low detection of DSM 32379 in the faecal metagenomes might result from the high genomic identity between DSM 32379 and the parental strain and the consequent insufficient coverage in the metagenomic data, and might be influenced by the high levels of DSM 32186 at baseline indicating a saturated niche in the intestinal lumen. However, as *F. prausnitzii* is also present at the mucosal interface^19^, faecal sampling might not reflect the counts of DSM 32379 close to the mucosa, where the oxygen-tolerant DSM 32379 might have a competitive advantage. Therefore, we hypothesize that our next-generation probiotic might be able to increase *F. prausnitzii* in patient groups with lower abundance of this bacterium (such as those with type 2 diabetes) and in individuals with intestinal inflammation (such as inflammatory bowel disease), as supported by the observation that administration of *F. prausnitzii* A2-165 improves colitis and is able to partially restore the microbiota in mice^9^.

Our study has some limitations. We have not explored transient or personalized gut microbiota responses to the administered formulation owing to limited longitudinal data. Furthermore, we could not determine the molecular mechanisms leading to increased oxygen tolerance in *F. prausnitzii*. Nevertheless, we have developed an approach based on the syntrophic interaction between *F. prausnitzii* and *D. piger* isolated in this study, that leads to increased growth of *F. prausnitzii* and butyrate production in vitro and might influence butyrate production potential in vivo in the human gut.

## Conclusion

Targeting the gut microbiota holds great potential for improving human health, and metagenomics studies in the past two decades have identified a broad range of bacteria that might be candidates for development of next-generation probiotics^2^. However, as 70% of the bacterial species detected in metagenomics surveys lacks cultured representatives^30^, few potential candidates have been assessed in human studies. For example, *Akkermansia muciniphila*^31^ and *Anaerobutyricum soehngenii*^25^ as single species or in combination with spore-forming bacteria^32^ have been found to be safe for human consumption and preliminary data show that they have positive effects on glucose metabolism in mice and humans^25,31,32^.

Major challenges for the development of human gut bacteria as next-generation probiotics are fastidious growth (that is, requirements for specific nutrients or conditions) and sensitivity to oxygen. Indeed, examples of human isolates selected as next-generation probiotics so far include *Bacteroides* and *Clostridium* strains^2^ (such as *Bacteroides fragilis* and *Clostridium butyricum*) that have relatively high oxygen tolerance among gut anaerobic bacteria^33,34^. Gut bacteria with inherent oxygen tolerance may develop increased oxygen tolerance upon direct exposure to oxygen, as shown by Meehan et al., who isolated oxygen-enabled variants of *B. fragilis*^34^. This approach cannot be applied to extremely oxygen-sensitive bacteria such as *F. prausnitzii*, which, however, can be targeted using the approach described here.

To our knowledge, strictly anaerobic bacteria such as *F. prausnitzii* had not been described in live formulations for human consumption. Since the abundance of *F. prausnitzii* is reduced in patients with hyperlipidaemia^8^, prediabetes and type 2 diabetes^5,6^, non-alcoholic fatty liver disease^35^ and inflammatory bowel disease^9^, the production of *F. prausnitzii* as next-generation probiotic is of great interest. Our strategy based on the exploitation of existing synergies between gut microorganisms and improved oxygen tolerance shows how *F. prausnitzii* could be developed as a next-generation probiotic for human consumption. This technology may be used for the development of other extremely oxygen-sensitive bacteria as next-generation probiotics to target patient populations with reduced abundances of these bacteria.

## Methods

### Co-isolation and cultivation of *F. prausnitzii* and *D. piger*

Ten micrograms of fresh faecal sample from a healthy male donor, 36 years of age, who had not received antibiotics in the previous 6 months, was inoculated directly on PGM agar plates and incubated anaerobically (5% H_2_, 10% CO_2_ and N_2_ as ground gas) at 37 °C in a Coy chamber (Coy Laboratory Products). PGM is a growth medium widely used for the isolation of sulfate-reducing bacteria.

Classical microbiological techniques were used to obtain pure cultures. After consecutive subcultures, random colonies were selected and subjected to Gram staining. Presumptive cell types of *F. prausnitzii* and *D. piger* were observed. After repeated sub-culturing on YCFAG and PGM media, which support growth of *F. prausnitzii* and *D. piger*, respectively, pure cultures of *F. prausnitzii* and *D. piger* were obtained and the isolates were identified by full length 16S rRNA gene sequencing. The isolates were deposited under the Budapest Treaty to Leibniz Institute DSMZ-German Collection of Microorganisms and Cell Cultures GmbH and were listed in the collection as *F. prausnitzii* DSM 32186 and *D. piger* DSM 32187.

*F. prausnitzii* strain DSM 32186, was routinely maintained under strictly anaerobic conditions in a Coy anaerobic chamber. The routine culture medium was YCFAG, containing: 2.5 g l^−1^ yeast extract, 10 g l^−1^ casitone, 4.5 g l^−1^ glucose, 0.9 g l^−1^ sodium chloride, 0.45 g l^−1^ dipotassium phosphate, 0.45 g l^−1^ potassium dihydrogen phosphate, 1.32 g l^−1^ ammonium sulfate, 4 g l^−1^ sodium bicarbonate, 1 g l^−1^ cysteine, 0.001 g l^−1^ resazurin, 0.01 g l^−1^ hemin, 100 µg l^−1^ biotin, 100 µg l^−1^ cobalamin, 300 µg l^−1^ *p*-aminobenzoic acid, 500 µg l^−1^ folic acid and 1,500 µg l^−1^ pyridoxamine. All components were added aseptically while the tubes were flushed with CO_2_. The media was autoclaved at 100 kPa at 121 °C for 15 min. Finally, thiamine and riboflavin were added through a 0.22-µm filter to final concentrations of 0.05 µg ml^−1^. The final concentrations of SCFAs in the medium were 33 mM acetate, 9 mM propionate and 1 mM each of isobutyrate, isovalerate and valerate.

*D. piger* DSM 32187 was maintained in PGM. PGM contains: 0.5 g l^−1^ dipotassium phosphate, 1 g l^−1^ ammonium chloride: 3. 5 g l^−1^ sodium lactate, 1 g l^−1^ yeast extract, 0.1 g l^−1^ ascorbate, 0.5 g l^−1^ cysteine, 1 g l^−1^ sodium chloride, 10 g l^−1^ peptone, 1 g l^−1^ sodium sulfate, 1 g l^−1^ calcium chloride, 2 g l^−1^ magnesium sulfate, 0.5 g l^−1^ ferrous sulfate, 0.5 g l^−1^ heptahydrate. Sodium sulfate, magnesium sulfate heptahydrate and calcium chloride were autoclaved separately while ferrous sulfate heptahydrate was filter-sterilized with a 0.22-µm filter and added after autoclaving and mixing of all components. The final pH of the medium was adjusted to 7.2 ± 0.2.

For co-culture experiments, modified PGM medium (mPGM) was prepared by adding 25 mM of glucose to PGM.

### Oxygen adaptation strategy

To increase oxygen tolerance in *F. prausnitzii* DSM 32186, a custom-made bioreactor (m-SHIRM) was used (Fig. 2b). In an anaerobic Coy chamber, an inoculum was prepared by inoculating a single bacterial colony in 7 ml of YCFAG. After 16 h of incubation at 37 °C (optical density at 600 nm (OD_600_) ≈ 0.7), 2.5 ml of this pre-culture was inoculated into the anodic chamber of m-SHIRM bioreactor containing 250 ml of YCFAG. The applied electrical oxidizing potentials were maintained via external voltage on a graphite anode (8.5 cm × 0.25 cm × 2.5 cm) via a potentiostat (CHI, 660C). The m-SHIRM bioreactor was maintained at 37 °C and purged for 15 min with nitrogen gas before inoculation. After 24 h (OD_600_ ≈ 0.7) 2.5 ml of the bacterial culture was re-inoculated into another m-SHIRM bioreactor with the same growth conditions, except for shifts in anodic potential and cysteine/cystine concentrations. This procedure was repeated ten times with increasing anodic potential and decreasing cysteine/cystine ratio (as graphically outlined in Fig. 2c).

### Selection of oxygen-adapted variants of *F. prausnitzii* DSM 32186

During the subculture steps presented in Fig. 2c, aliquots of 100 µl were collected and serially diluted in 900 µl of phosphate buffer saline (PBS). Aliquots of 50 µl from each dilution were inoculated on YCFAG and incubated anaerobically for 72 h. After incubation, viable counts were assessed. Based on differential colony morphotype, colonies were picked (Extended Data Fig. 3) and preserved as glycerol stocks (YCFAG containing 20% glycerol) at −80 °C. The oxygen-adapted variants were checked for purity via Gram staining.

### Assessment of oxygen tolerance

Oxygen tolerance of *F. prausnitzii* DSM 32186 and its variants was assessed in YCFAG, and in PGM medium for *D. piger* DSM 32187. Strains were precultured anaerobically in broth medium for 14 h. After cultivation, tenfold serial dilutions were prepared anaerobically and 100 µl of each dilution were inoculated on two sets of each YCFAG or PGM agar medium. Plates incubated under anaerobic conditions served as control of viability for plates exposed to ambient air for 20 min, which were used to determine oxygen tolerance. Oxygen diffusion was confirmed by the oxidation of the resazurin dye present in YCFAG medium. After exposure to ambient air, plates were incubated anaerobically in a Coy chamber for 72 h before the CFUs were counted.

### Quantification of bacterial metabolites

Glucose, SCFA and lactate were measured by high-performance liquid chromatography (HPLC) with refractive index detection. Twenty microlitres of cultured broth centrifuged and filter-sterilized was injected on a Reprogel H 9 µm column (250 × 4.6 mm) with a guard column. Jasco AS-2507 plus auto-injector Samples were cooled at 4 °C and 0.0025 M sulfuric acid was used as eluent at a flow rate of 400 µl min^−1^ with a UltiMate 3000 pump from Dionex. Peaks were detected with Bischoff 8020 RI detector.

### Extracellular electron transfer with riboflavin

As previously described, *F. prausnitzii* can exploit riboflavin for extracellular electron transfer to the anode in a microbial fuel cell^19^. YCFAG agar plates were inoculated with *F. prausnitzii* DSM 32186 and DSM 32379 from frozen glycerol stocks kept at −80 °C and incubated anaerobically (5% H_2_, 10% CO_2_ and N_2_) at 37 °C in a Coy chamber (Coy Laboratory Products). Single colonies were inoculated in 6 ml YCFAG broth and incubated overnight anaerobically, at 37 °C. When cultures reached an OD_600_ of ~0.9, cells were harvested by centrifugation at 4,000 rpm for 20 min. The cell pellet was resuspended in 200 µl of anolyte and injected in the anode chamber. Cells were incubated for 5 min before challenging with riboflavin 200 µM as electron mediator.

The custom-made two-chambered, microbial fuel cell was assembled as previously described with some modifications^36^. The bed volume of the cathode and anode chambers was 9 ml and the working volume was 6 ml. The two chambers were separated by a 1.8 cm diameter septum of CMI-7000S cation exchange membrane (Membranes International). Graphite plates of dimensions of 2 cm × 1 cm × 0.2 cm were used as cathode and anode. The distance between the two electrodes was 10 cm. The electrodes were connected to the external circuit with insulated copper wires and the circuit was closed via a fixed resistance of 150 Ω. The anode chamber contained 50 mM potassium phosphate buffer (pH 7.0) as anolyte and 0.1 M glucose. The cathode chamber contained 100 mM potassium phosphate buffer (pH 7.0) with 50 mM potassium ferricyanide as catholyte. The cell was maintained at 37 °C and the anode and cathode chambers were purged continuously with nitrogen gas and air, respectively. The data was recorded using a LabJack data acquisition system (LabJack Corporation) at an interval of 1 min.

### Immunomodulatory function in Caco-2 cells

Caco-2 from the European Collection of Cell Cultures (batch 18H036, Merck) were cultured in supplemented Dulbecco’s modified Eagle’s medium (DMEM) (PAA Laboratories) at 37 °C in a 5% CO2 incubator. Cells were incubated with different *F. prausnitzii* supernatant fractions cultured in LYBHI (1:25 and 1:10 in DMEM medium) and stimulated with (4 ng ml^−1^ IL-1β) for 6 h. IL-8 levels were determined in duplicate in cell supernatants using ELISA kit DuoSet (R&D systems). Cells were regularly tested for mycoplasma infections.

### Safety of the oral administration of *F. prausnitzii* and *D. piger* in mice

Male and female 8-week-old Swiss Webster mice were co-housed with 5 mice per cage at a temperature of 20 ± 1 °C and an air humidity of 45–70% under specific pathogen-free conditions at a 12-h light:dark cycle (light from 07:00 to 19:00) and were fed an autoclaved chow diet (LabDiet) and water ad libitum. The mice were administered either a bacterial culture containing *F. prausnitzii* DSM 32379 and *D. piger *DSM 32187 or a medium/glycerol vehicle, five times during the first week and then twice a week for the following three weeks. Total genomic DNA was isolated from mouse caecal contents as previously described^37^ and quantified by the Quant-iT PicoGreen dsDNA Assay Kit (Invitrogen). *F. prausnitzii* and *D. piger* were quantified by qPCR using primers Fpr-2F (GGAGGAAGAAGGTCTTCGG)/Fprau645-R (AATTCCGCCTACCTCTGCAC)^38,39^ and DSV691-F (CCGTAGATATCTGGAGGAACATCAG)/DSV826-R (ACATCTAGCATCCATCGTTTACAGC)^40^. Clinical observations were made once a day. Clonic or tonic movements, stereotypic or abnormal behaviour were monitored. Body weight and food consumption were monitored. Haematological examinations performed included haematocrit, haemoglobin concentration, erythrocyte count, total and differential leukocyte count and platelet count. Clinical biochemistry examinations of blood samples performed included sodium, potassium, urea, total cholesterol, blood urea nitrogen, creatinine, total protein, total albumin, alanine aminotransferase, alkaline phosphatase, gamma glutamyl transpeptidase and bile acids. Histopathological examination was performed on stomach duodenum, small and large intestines (including Peyer’s patches), liver, spleen, thymus and mesenteric lymph nodes. Clinical observations, body weight, food consumption, organ weight assessments and autopsies were performed without blinding. Blood haematology, clinical biochemistry and histopathology were assessed by blinded external personnel. No sample size calculation or randomization were performed. All animal procedures were approved by the Gothenburg Animal Ethics Committee (Dnr 5.8.18-16056/2019).

### Safety of the oral administration of *F. prausnitzii* and *D. piger* in young, healthy men and women

#### Study design

The study was a double-blind, randomized, placebo-controlled, single-centre trial of 10 weeks in healthy men and women 20 to 40 years of age. Eligible participants were randomly allocated to receive capsules once daily with a high (1 × 10^9^ to 5 × 10^9^ CFU per bacterial strain; *n* = 18) or low dose (1 × 10^8^ to 5 × 10^8^ CFU; *n* = 16) of *D. piger* and *F. prausnitzii* or placebo (*n* = 16) for 8 weeks, followed by a 2-week period without supplementation. In total, 16 (high dose), 16 (low dose) and 14 (placebo) participants completed the whole study. No study participant discontinued the study owing to an adverse event. Randomization was performed by the sponsor (Metabogen) using Sealed Envelope (2017, https://www.sealedenvelope.com/simple-randomiser/v1/lists). Randomization was stratified according to sex. Information regarding study design, analysis and study objectives was published on Clinicaltrials.gov (NCT03728868) prior to study start. The study was approved by the Regional Ethics Review Board in Gothenburg.

#### Participants

Participating volunteers were recruited through advertising in social media (for example, on Facebook and Instagram) and through posters in public areas (for example, in Universities, hospitals and gyms). Participants who met all inclusion criteria (20–40 years of age, signed informed consent, healthy without any known disease, willingness and able to participate at planned visits, phone interviews and to follow instructions, understand spoken and written Swedish), lacked all exclusion criteria (ongoing treatment with prescribed medication, intake of probiotic supplementation, treatment with antibiotics within the last three months, pregnancy, gastrointestinal tract symptoms during the last months that could affect study participation, current tobacco use, participation in other clinical studies), had normal blood biochemistry, blood pressure and heart rate, were invited to participate.

#### Primary and secondary end points

The primary outcome was tolerability and was tested using discontinuation (yes/no) due to investigational product during 8 weeks of treatment. Secondary end points including change (between baseline and 8 weeks) in Gastrointestinal Symptom Rating Scale (GSRS), fasting blood glucose, glycosylated haemoglobin (HbA1c), renal function (estimated glomerular filtration rate (eGFR) based on serum creatinine), red and white blood cell count, serum alanine transaminase (ALT), serum aspartate transaminase (AST), serum alkaline phosphatase (ALP), serum bilirubin, serum C-reactive protein (CRP), serum total protein, faeces SCFA levels (butyrate, acetate, lactate, proprionate, isovalerate, isobutyrate and succinate) were evaluated between baseline to week 4 and 8 (and after 10 weeks for SCFAs).

#### Procedures

Six visits to the study clinic (Geriatric Medicine, Sahlgenska University Hospital, Mölndal) were required during the study duration. Participants received information about the study both in writing and verbally. Eligible participants with no exclusion criteria provided a signed informed consent prior to any study procedures and enrolment. Heart rate and blood pressure were measured twice at the screening visit, using a Carescape V100 device (GE Healthcare). Body height, weight, and waist and hip circumferences were measured with a stadiometer as well as a scale and measuring tape. Venous blood was drawn from the cubital vein and used for blood biochemistry analyses. All blood biochemistry was analysed within 4 h after sampling at the Clinical Chemistry laboratory (Sahlgrenska University Hospital Mölndal). All women also completed a pregnancy test (urine human chorionic gonadotropin) which had to be negative for inclusion.

At the randomization visit, faecal samples were collected and GSRS was completed in order to collect information of any gastrointestinal symptoms the preceding week. All participants received a diary for recording daily doses taken and to make notes about any potential adverse events. During study visits three to five, faeces and blood samples as well as data from the GSRS questionnaire form were collected. Two weeks after treatment completion, a last study visit took place to collect data on gastrointestinal symptoms (GSRS) and to collect stool samples. The first 15 randomized subjects were contacted by telephone daily the first week to enquire about any potential adverse events. Thereafter, all participants were contacted by telephone once a week for inquiries about adverse events and to collect information about gastrointestinal symptoms (GSRS) the preceding week.

#### Intervention

The study product was provided as freeze-dried bacteria packed into capsules designed to disintegrate when reaching the small intestine. Identical capsules and excipient were used for the placebo and interventional product.

#### Assessment of gastrointestinal symptoms

Assessment of gastrointestinal symptoms the last week, was performed using the GSRS questionnaire^41^. GSRS contains 15 items in total and was analysed as a total score ranging from 0 to 45. Values 0–9 correspond to none to minimal gastrointestinal issues, 10–19 correspond to minimal gastrointestinal issues, 20–29 correspond to moderate gastrointestinal issues, 30–39 correspond to moderate to severe gastrointestinal issues, and 40–45 correspond to severe gastrointestinal issues.

#### Blood biochemistry

All blood biochemistry analyses were performed at the Swedac accredited (accreditation number 1240) clinical chemistry laboratory at the Sahlgrenska University Hospital. Blood glucose was measured using Glucose HK on a Cobas 6000 instrument (Roche Diagnostics Scandinavia). The coefficient of variance (CV) was 3% at concentrations of 5 and 15 mM. HbA1c was measured using HPLC (Mono S, Tricorn 50/50 GL (CDP), MonoBeads Column (GE Healthcare)). The separated haemoglobin fractions were measured using an UV-detector and absorbance quantified at 417 nm. The CV was 2% at concentrations 42 mmol per mol, 63 mmol per mol and 94 mmol per mol. Erythrocyte sedimentation rate was measured using the Starrsed ST Instrument, Mechatronics (Triolab). Erythrocyte count (CV: 3% at 2, 4 and 5 × 10^12^ l^−1^) was measured using anti coagulated venous blood with K2-EDTA and measurement of the absorption of light. The instrument used was the ADVIA 2120i (Siemens Medical Solutions Diagnostics). Leukocyte count was measured using anti coagulated venous blood with K2-EDTA and measurement of the absorption of light, using the ADVIA 2120i instrument (Siemens Medical Diagnostics AB), with a CV of 7% at concentrations 3 × 10^9^ l^−1^ to 16 × 10^9^ l^−1^. Thrombocyte count was measured using anti coagulated venous blood with K2-EDTA and measurement of the absorption of light, with a CV of 9% at 80, 200 and 500 × 10^9^ l^−1^, analysed on a ADVIA 2120i instrument. ALT catalyses the reaction between l-alanine and 2-oxoglutarate. Further reaction between the produced pyruvate and NADH generates a measure of NADH oxidation, which was directly proportional to the ALT activity, which was measured via the decrease in absorbance. The CV was 6% at 1 µkat l^−1^ and 4% at 4 µkat l^−1^ and the instrument used was the Cobas 6000. AST catalyses l-aspartate and 2-oxoglutatrate to oxaloacetate and l-glutamate. The reaction between oxaloacetate and NADH generates a measure of NADH oxidation, which was directly proportional to the AST activity, which was measured via the decrease in absorbance. The CV was 5% at 1 µkat l^−1^ and 3% at 3 µkat l^−1^ and the instrument used was the Cobas 6000. ALP was analysed using a colorimetric assay using Cobas 6000 with a CV of 4% at 7 μkat l^−1^. Serum total bilirubin was measured using a colometric assay on a Cobas system (Roche Diagnostics Scandinavia), with a CV of 5% at concentrations 20 and 130 µM. Serum creatinine was measured using CREP2 on a Cobas 6000 instrument, with a CV of 4% at concentrations 85 and 400 μM. The estimated glomerular filtration rate (eGFR) was calculated using the Lund-Malmö formula based on serum creatinine, age and sex^42^. Total protein was measured using on a Cobas 6000 with a CV of 3% at concentrations 50 and 75 g l^−1^.

#### Faecal SCFAs

Faecal concentrations of the SCFAs acetate, propionate and butyrate, as well as succinate and lactate, were determined using gas chromatography–mass spectrometry (Agilent Technologies) as previously described^43^. In brief, 100 mg of frozen faecal material was transferred to a 16 × 125 mm tube fitted with a screw cap, and a volume of 100 µl of internal standard stock solution ([1-^13^C]acetate, [^2^H6]propionate 1 M, [^13^C4]butyrate 0.5 M, [1-^13^C1]isobutyrate and [1-^13^C]isovalerate 0.1 M) was added. Prior to extraction, samples were freeze-dried overnight. After acidification with 50 µl of 37% HCl, the organic acids were extracted twice in 2 ml of diethyl ether. A 500 µl aliquot of the extracted sample was mixed with 50 µl of *N*-tert-butyldimethylsilyl-*N*-methyltrifluoracetamide (Sigma) at 20 °C. One microlitre of the derived material was injected into a gas chromatograph (Agilent Technologies 7890 A) coupled to a mass spectrometer detector (Agilent Technologies 5975 C). Temperature was increased in a linear gradient consisting of initial temperature of 65 °C for 6 min, increase to 260 °C at 15 °C min^−1^, and increase to and held at 280 °C for 5 min. The injector and transfer line temperatures were 250 °C. Quantitation was completed in ion-monitoring acquisition mode by comparison to labelled internal standards, with the *m*/*z* ratios 117 (acetic acid), 131 (propionic acid), 145 (butyric acid), 146 (isobutyric acid), 159 (isovaleric acid), 121 ([^2^H2,1-^13^C]acetate), 136 ([^2^H5]propionate), 146 ([1-^13^C1]isobutyrate), 149 ([^13^C4] butyrate), 160 ([1-^13^C]isovalerate).

#### Statistical analyses

Statistical power was calculated based on anticipated differences in the proportions of study subjects discontinuing due to adverse events. With a discontinuation rate of 0.50 versus 0.05 due to investigational product in the two treatment groups versus placebo group (randomized in 2:1, 32 versus 16 subjects), respectively, with an alpha level of 0.05, using the two-sided Fisher’s exact test, a power of 88% was achieved.

All analyses were done both in the intention to treat and in the per protocol populations. Comparison of continuous variables between treatment groups (low and high dose) and placebo was performed with Fisher’s non-parametric permutation test and the Fisher’s exact test (lowest one-sided *P* value multiplied by 2) was used for dichotomous variables. The primary outcome was tolerability and was tested using discontinuation (yes/no) due to investigational product during 8 weeks of treatment. The potential differences in the secondary end point variables, were evaluated by relative change adjusted for placebo and compared with the Fisher’s non-parametric permutation test, which also generated the confidence interval for the mean difference. All analyses were performed on complete cases—that is, no imputations were used. Statistical significance was considered for *P* values below 0.05 and all statistics were performed with SAS Software version 9.4 (SAS Institute). Confidence intervals form primary outcomes were calculated using the Newcombe hybrid score interval^44^. Permutation-based confidence intervals (Supplementary Tables 5–8) were calculated using a user-written SAS macro^45,46^ (https://github.com/imbhe/PermTestCI).

### Measurement of faecal hydrogen sulfide

Hydrogen sulfide was quantified as previously described^47^. All reagents and buffers were degassed by purging with nitrogen. Faecal samples were cut and aliquoted (~150 mg) on dry ice and kept frozen in 2-ml airtight propylene tubes. Samples were then transferred to anaerobic chamber (COY) and diluted in phosphate buffered saline. Diluted faecal slurries were treated with a zinc acetate solution before addition of reagent solution consisting of *N*,*N*-dimethyl-*p*-phenylenediamine sulfate. The tubes were immediately closed, vortexed, and maintained at room temperature for 20 min and absorbance was measures at a wavelength of 670 nm. Hydrogen sulfide was measured in faecal samples of 40 individuals (placebo, *n* = 12; low dose, *n* = 16; high dose, *n* = 12) who had stools both at baseline and at the end of the administration; no sufficient material was available for 1 individual in the placebo and 2 in the high-dose groups.

### DNA extraction from faecal samples and shotgun metagenomic sequencing

Stool samples were collected by the participants at home and stored at room temperature until delivery to the clinic, where samples were stored at −80 °C. The maximum time between sampling and delivery to the clinic was 24 h. Total genomic DNA was isolated from 100–150 mg of faecal material using a modification of the IHMS DNA extraction protocol Q^48^. Samples were extracted in Lysing Matrix E tubes (MP Biomedicals) containing ASL buffer (Qiagen), vortexed for 2 min and lysed by two cycles of heating at 90 °C for 10 min followed by two bursts of bead beating at 5.5 m s^−1^ for 60 s in a FastPrep-24 Instrument (MP Biomedicals). After each bead-beating burst, samples were placed on ice for 5 min. Supernatants were collected after each cycle by centrifugation at 4 °C. Supernatants from the two centrifugations steps were pooled and a 600-µl aliquot from each sample was purified using the QIAamp DNA Mini kit (QIAGEN) in the QIAcube (QIAGEN) instrument using the procedure for human DNA analysis. Samples were eluted in 200 µl of AE buffer (10 mM Tris·Cl; 0.5 mM EDTA; pH 9.0). Libraries for shotgun metagenomic sequencing were prepared by a PCR-free method; library preparation and sequencing were performed at Novogene (China) on a NovaSeq instrument (Illumina) with 150-bp paired-end reads and at least 6G data per sample.

### DNA extraction from bacterial cultures for genome sequencing

Total genomic DNA was extracted from microbial biomass harvested after an overnight growth or at the stationary phase. Biomass obtained from liquid cultures was collected by centrifugation for 10 min at 4,500 rpm at 4 °C, and washed once with PBS to remove carry-over contaminants.

DNA for Illumina short-reads sequencing was extracted using the NucleoSpin Soil kit (740780.50, Macherey-Nagel) as described by the manufacturer in the presence of SL2 lysis buffer and Sx enhancer. Cells were lysed by 2 rounds of bead beating at 5.5 m s^−1^ for 60 s in a FastPrep-24 Instrument (MP Biomedicals), with incubation on ice for 5 min in between the two bead-beating bursts. DNA quality was evaluated using Tapestation 4200 with Genomic DNA ScreenTape and reagents (Agilent), and quantification was made using the Quant-iT dsDNA BR Assay Kit (ThermoFisher Scientific). Libraries for sequencing were prepared using Covaris S220 Focused-ultrasonicator (Covaris), fragmented to average 550-bp insert size, and the TruSeq DNA PCR-free Library Preparation kit (20015963 and 20015949, Illumina). Libraries were quantified using Quant-iT dsDNA HS Assay Kit (ThermoFisher Scientific) and sequenced on an Illumina Miseq instrument using MiSeq Reagent Kit v3, 600 cycles.

Large amounts of high-quality DNA for Nanopore long-reads sequencing were obtained with a modified version of the Marmur procedure^49^. Cells were suspended in Tris-EDTA buffer (20 mM Tris HCl pH 8, 2 mM EDTA) and lysed with lysozyme (20 mg ml^−1^) and SDS (2% w/v) in the presence of proteinase K. The extracted total DNA was purified by repeated extraction in phenol:chloroform:isoamyl alcohol (25:24:1 v/v) and chloroform:isoamyl alcohol (24:1 v/v), followed by precipitation in cold ethanol (99.5% v/v) and spooling of the DNA on a glass rod. The DNA was washed with ethanol 70% (v/v), dried at room temperature and resuspended in water overnight at 4 °C. DNA integrity and concentration were evaluated using Tapestation 4150 with Genomic DNA ScreenTape and reagents (Agilent) and Qubit 3.0 Fluorometer and Qubit dsDNA BR assay kit (ThermoFisher Scientific). Isolated DNA was prepared using Rapid barcoding kit (SQK-RBK004) following the manufacturer’s instructions (ONT) and sequenced on a ONT’s MinION device on a R9.4.1 flow cell (FLO-MIN106D). Base-calling was performed using ONT’s guppy v. 4.2.2.

### Bioinformatics methods

#### Genome analyses

The genomes of *F. prausnitzii* DSM 32186 and DSM 32379 and *D. piger* DSM 32187, were obtained by hybrid assembly of Nanopore and Illumina reads. The Unicycler pipeline v0.4.8 in hybrid mode used to obtain de novo assemblies. All dependencies for Unicycler were installed in a conda environment. The dependency programs include SPAdes v3.13.0, racon v1.4.1, bowtie2 v2.3.5.1, and pilon v1.23. The hybrid assemblies were annotated using Prokka v1.14.5 (https://github.com/tseemann/prokka).

To infer evolutionary relationships, the *F. prausnitzii* and *D. piger* genomes were aligned with publicly available high-quality genomes of the same species and/or representative sequences of previously known clades, their near neighbours and outgroups respectively using progressiveMauve^50^. Multiple alignments were used to reconstruct phylogenies of both strains in MEGA X^51^. Evolutionary distances were calculated using the maximum composite likelihood method and are in the units of number of base substitutions per site^52^.

#### Whole-genome metagenomics analyses and genome capture

Illumina reads were quality filtered and trimmed using fastq_quality_trimmer from the fastX toolkit (https://github.com/lianos/fastx-toolkit/); human reads were removed by mapping the high-quality reads against the human genome (hg19) using Bowtie2 (ref. ^53^) (v2.4.4). After removal of low-quality (quality score <20) and human reads, we obtained high-quality paired-end microbial reads with and average depth of 45 million for each faecal sample.

For genome capture, high-quality microbial reads were mapped using Kraken 2 (ref. ^54^) (v2.1.2) with default settings against a custom database designed by adding the closed genomes of the novel strains *F. prausnitzii* DSM 32186 and *D. piger* DSM 32187 to the RefSeq database (release 107). Estimations of strain abundances were obtained using Bracken^55^ (v2.6.2) for reads with minimum length of 100 bp.

The overall composition of the gut microbiota was assessed for the abundance of species using principal coordinates analysis on Bray-Curtis dissimilarity. Differences in composition were tested by a permutational multivariate ANOVA using the *adonis2* function with 10,000 permutations in the vegan package in R (https://github.com/vegandevs/vegan/).

Gene counts in the metagenomic data were estimated using MEDUSA^56^ with a gene catalogue containing 15,186,403 non-redundant microbial genes^6^. The butyrate production potential was quantified based on five genes (*but*, *buk 4hbt* and *ato*A/D) coding for the terminal enzymes in the four intestinal butyrate-producing pathways^57^. Profile hidden Markov models were used to screen those genes in the gene catalogue, as previously described^6^.

Genetic variants were detected for the oxygen-tolerant *F. prausnitzii* DSM 32379 by mapping the raw reads against the assembled genome of the parental strain DSM 32186 using snippy v4.4.5 in default setting (https://github.com/tseemann/snippy). To detect the genetic variants in the faecal metagenomes, we obtained *F. prausnitzii* reads mapping to DSM 32379 in each sample using bowtie2 v2.3.5.1, and then performed variant calling against the parental genome DSM 32186 using snippy v4.4.5. *F. prausnitzii* DSM 32379 was considered as possibly detected in a sample only if: (1) genetic variants of DSM 32379 were only detected at the end of the administration; (2) the genetic variants covering the related genomic regions had an abundance of at least 10% of all reads; and (3) in case of detection at baseline, multiple variants must be detected at the end of the administration with an increase in all of their frequencies in that faecal sample (Supplementary Table 1).

### Statistics

Statistical analyses were conducted using GraphPad Prism v_8.4.3. Two-sided Student’s *t*-tests were used to compare two groups and one-way ANOVA with Tukey’s multiple comparison were used to compare three groups.

Non-parametric tests were used to compare the abundance of species and strains in the faecal microbiomes. Wilcoxon signed-rank tests were used to compare abundances at the end of the administration compared to baseline in matching samples from an individual. Kruskal–Wallis tests were used to compare three groups.

### Reporting summary

Further information on research design is available in the Nature Portfolio Reporting Summary linked to this article.

## Online content

Any methods, additional references, Nature Portfolio reporting summaries, source data, extended data, supplementary information, acknowledgements, peer review information; details of author contributions and competing interests; and statements of data and code availability are available at 10.1038/s41586-023-06378-w.

### Supplementary information

Supplementary Information Supplementary Information containing Supplementary Tables 1–7, trial details and statistical report.

Reporting Summary

Supplementary Table 8 Number of reads in fecal metagenomes mapping to the genetic variants that characterize the oxygen tolerant *F. prausnitzii* DSM 32379.

Supplementary Table 9 Data from mouse safety study

## Figures and Tables

**Extended Data Table 1:**
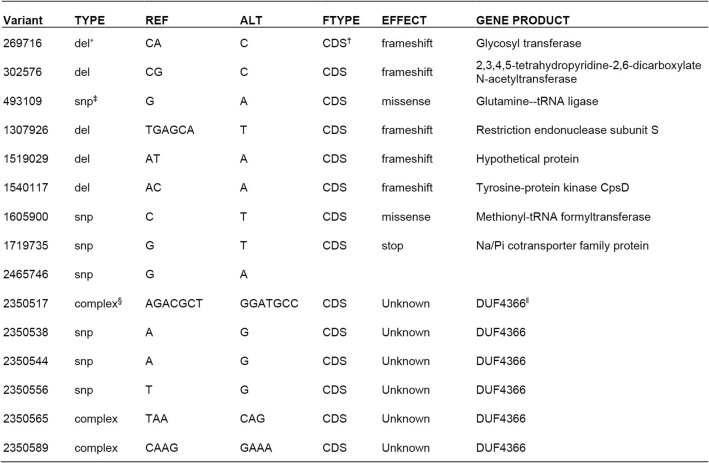
Genetic variants present in the genome of the oxygen tolerant *F. prausnitzii* DSM 32379

^*^del, deletion

^†^CDS, coding sequence

^‡^SNP, single nucleotide polymorphism

^§^complex, combination of mutation types, eg deletion & single base substitution

^ǁ^DUF, domain of unknown function

## Data Availability

Supplementary information on data availability is linked to the online version of the paper. Genome assemblies and raw metagenomic sequence data have been deposited in the EMBL-EBI European Nucleotide Archive (ENA) under accession number PRJEB62463. Processed sequence data required for reanalysis are available at GitHub (https://zenodo.org/record/8019851). Processed pseudonymized per-subject metadata are provided in Supplementary Tables 2–8. Data from the mouse safety study are available in Supplementary Table 9. For questions on the clinical cohort, contact M.L. Bacterial strains are proprietary to Metabogen AB and should be requested from them.

## References

[1] Schroeder, B. O. & Backhed, F. Signals from the gut microbiota to distant organs in physiology and disease. *Nat. Med.* **22**, 1079–1089 (2016). 27711063 10.1038/nm.4185

[2] O’Toole, P. W., Marchesi, J. R. & Hill, C. Next-generation probiotics: the spectrum from probiotics to live biotherapeutics. *Nat. Microbiol.* **2**, 17057 (2017). 28440276 10.1038/nmicrobiol.2017.57

[3] Sender, R., Fuchs, S. & Milo, R. Revised estimates for the number of human and bacteria cells in the body. *PLoS Biol.* **14**, e1002533 (2016). 27541692 10.1371/journal.pbio.1002533 PMC4991899

[4] Li, J. et al. An integrated catalog of reference genes in the human gut microbiome. *Nat. Biotechnol.* **32**, 834–841 (2014). 24997786 10.1038/nbt.2942

[5] Allin, K. H. et al. Aberrant intestinal microbiota in individuals with prediabetes. *Diabetologia* **61**, 810–820 (2018). 29379988 10.1007/s00125-018-4550-1 PMC6448993

[6] Wu, H. et al. The gut microbiota in prediabetes and diabetes: a population-based cross-sectional study. *Cell Metab.* **32**, 379–390.e3 (2020). 32652044 10.1016/j.cmet.2020.06.011

[7] Qin, J. et al. A metagenome-wide association study of gut microbiota in type 2 diabetes. *Nature* **490**, 55–60 (2012). 23023125 10.1038/nature11450

[8] Fu, J. Y. et al. The gut microbiome contributes to a substantial proportion of the variation in blood lipids. *Circ. Res.* **117**, 817–824 (2015). 26358192 10.1161/CIRCRESAHA.115.306807 PMC4596485

[9] Sokol, H. et al. *Faecalibacterium prausnitzii* is an anti-inflammatory commensal bacterium identified by gut microbiota analysis of Crohn disease patients. *Proc. Natl Acad. Sci. USA* **105**, 16731–16736 (2008). 18936492 10.1073/pnas.0804812105 PMC2575488

[10] Cao, Y., Shen, J. & Ran, Z. H. Association between *Faecalibacterium prausnitzii* reduction and inflammatory bowel disease: a meta-analysis and systematic review of the literature. *Gastroent. Res. Pract.* **2014**, 872725 (2014). 10.1155/2014/872725 PMC3985188 24799893

[11] De Filippis, F., Pasolli, E. & Ercolini, D. Newly explored *Faecalibacterium* diversity is connected to age, lifestyle, geography, and disease. *Curr. Biol.* **30**, 4932–4943 (2020). 33065016 10.1016/j.cub.2020.09.063

[12] Koh, A., De Vadder, F., Kovatcheva-Datchary, P. & Backhed, F. From dietary fiber to host physiology: short-chain fatty acids as key bacterial metabolites. *Cell* **165**, 1332–1345 (2016). 27259147 10.1016/j.cell.2016.05.041

[13] Wang, S. P. et al. Pivotal roles for pH, lactate, and lactate-utilizing bacteria in the stability of a human colonic microbial ecosystem. *mSystems* **5**, e00645-20 (2020). 32900872 10.1128/mSystems.00645-20 PMC7483512

[14] Smith, N. W., Shorten, P. R., Altermann, E. H., Roy, N. C. & McNabb, W. C. Hydrogen cross-feeders of the human gastrointestinal tract. *Gut Microbes* **10**, 270–288 (2019). 30563420 10.1080/19490976.2018.1546522 PMC6546324

[15] Warren, Y. A., Citron, D. M., Merriam, C. V. & Goldstein, E. J. C. Biochemical differentiation and comparison of *Desulfovibrio* species and other phenotypically similar genera. *J. Clin. Microbiol.* **43**, 4041–4045 (2005). 16081948 10.1128/JCM.43.8.4041-4045.2005 PMC1233901

[16] Olsson, L. M. et al. Dynamics of the normal gut microbiota: a longitudinal one-year population study in Sweden. *Cell Host Microbe* **30**, 726–739.e3 (2022). 10.1016/j.chom.2022.03.002 35349787

[17] Rey, F. E. et al. Metabolic niche of a prominent sulfate-reducing human gut bacterium. *Proc. Natl Acad. Sci. USA* **110**, 13582–13587 (2013). 23898195 10.1073/pnas.1312524110 PMC3746858

[18] Lopez-Siles, M., Duncan, S. H., Garcia-Gil, L. J. & Martinez-Medina, M. *Faecalibacterium**prausnitzii*: from microbiology to diagnostics and prognostics. *ISME J.* **11**, 841–852 (2017). 10.1038/ismej.2016.176 PMC5364359 28045459

[19] Khan, M. T. et al. The gut anaerobe *Faecalibacterium prausnitzii* uses an extracellular electron shuttle to grow at oxic–anoxic interphases. *ISME J.* **6**, 1578–1585 (2012). 10.1038/ismej.2012.5 PMC3400418 22357539

[20] Duncan, S. H. et al. Contribution of acetate to butyrate formation by human faecal bacteria. *Br. J. Nutr.* **91**, 915–923 (2004). 15182395 10.1079/BJN20041150

[21] Duncan, S. H., Hold, G. L., Harmsen, H. J. M., Stewart, C. S. & Flint, H. J. Growth requirements and fermentation products of *Fusobacterium prausnitzii*, and a proposal to reclassify it as *Faecalibacterium prausnitzii* gen. nov., comb. nov. *Int. J. Syst. Evol. Microbiol.* **52**, 2141–2146 (2002). 12508881 10.1099/00207713-52-6-2141

[22] Khan, M. T., van Dijl, J. M. & Harmsen, H. J. Antioxidants keep the potentially probiotic but highly oxygen-sensitive human gut bacterium *Faecalibacterium prausnitzii* alive at ambient air. *PLoS ONE* **9**, e96097 (2014). 24798051 10.1371/journal.pone.0096097 PMC4010535

[23] Koh, A. et al. Microbially produced imidazole propionate impairs insulin signaling through mTORC1. *Cell* **175**, 947–961.e17 (2018). 10.1016/j.cell.2018.09.055 30401435

[24] Chen, Y. R. et al. *Desulfovibrio* is not always associated with adverse health effects in the Guangdong Gut Microbiome Project. *PeerJ* **9**, e12033 (2021). 10.7717/peerj.12033 PMC8380029 34466295

[25] Gilijamse, P. W. et al. Treatment with *Anaerobutyricum soehngenii*: a pilot study of safety and dose-response effects on glucose metabolism in human subjects with metabolic syndrome. *NPJ Biofilms Microbiomes* **6**, 16 (2020). 32221294 10.1038/s41522-020-0127-0 PMC7101376

[26] Jie, Z. et al. The baseline gut microbiota directs dieting-induced weight loss trajectories. *Gastroenterology* **160**, 2029–2042.e16 (2021). 33482223 10.1053/j.gastro.2021.01.029

[27] Rodriguez, J. et al. Discovery of the gut microbial signature driving the efficacy of prebiotic intervention in obese patients. *Gut* **69**, 1975–1987 (2020). 32041744 10.1136/gutjnl-2019-319726 PMC7569399

[28] Kootte, R. S. et al. Improvement of insulin sensitivity after lean donor feces in metabolic syndrome is driven by baseline intestinal microbiota composition. *Cell Metab.* **26**, 611–619.e616 (2017). 28978426 10.1016/j.cmet.2017.09.008

[29] Aggarwala, V. et al. Precise quantification of bacterial strains after fecal microbiota transplantation delineates long-term engraftment and explains outcomes. *Nat. Microbiol.* **6**, 1309–1318 (2021). 34580445 10.1038/s41564-021-00966-0 PMC8993687

[30] Almeida, A. et al. A unified catalog of 204,938 reference genomes from the human gut microbiome. *Nat. Biotechnol.* **39**, 105–114 (2021). 32690973 10.1038/s41587-020-0603-3 PMC7801254

[31] Depommier, C. et al. Supplementation with *Akkermansia muciniphila* in overweight and obese human volunteers: a proof-of-concept exploratory study. *Nat. Med.* **25**, 1096–1103 (2019). 31263284 10.1038/s41591-019-0495-2 PMC6699990

[32] Perraudeau, F. et al. Improvements to postprandial glucose control in subjects with type 2 diabetes: a multicenter, double blind, randomized placebo-controlled trial of a novel probiotic formulation. *BMJ Op. Diabetes Res. Care* **8**, e001319 (2020). 10.1136/bmjdrc-2020-001319 PMC7368581 32675291

[33] Morvan, C., Folgosa, F., Kint, N., Teixeira, M. & Martin-Verstraete, I. Responses of Clostridia to oxygen: from detoxification to adaptive strategies. *Environ. Microbiol.* **23**, 4112–4125 (2021). 34245087 10.1111/1462-2920.15665

[34] Meehan, B. M., Baughn, A. D., Gallegos, R. & Malamy, M. H. Inactivation of a single gene enables microaerobic growth of the obligate anaerobe *Bacteroides fragilis*. *Proc. Natl Acad. Sci. USA* **109**, 12153–12158 (2012). 22778399 10.1073/pnas.1203796109 PMC3409759

[35] Loomba, R. et al. Gut microbiome-based metagenomic signature for non-invasive detection of advanced fibrosis in human nonalcoholic fatty liver disease. *Cell Metab.* **25**, 1054–1062.e5 (2017). 10.1016/j.cmet.2017.04.001 PMC5502730 28467925

[36] Khan, M. T., Browne, W. R., van Dijl, J. M. & Harmsen, H. J. How can *Faecalibacterium prausnitzii* employ riboflavin for extracellular electron transfer? *Antioxid. Redox Signal.* **17**, 1433–1440 (2012). 22607129 10.1089/ars.2012.4701

[37] Caesar, R., Tremaroli, V., Kovatcheva-Datchary, P., Cani, P. D. & Backhed, F. Crosstalk between gut microbiota and dietary lipids aggravates WAT inflammation through TLR signaling. *Cell Metab.* **22**, 658–668 (2015). 26321659 10.1016/j.cmet.2015.07.026 PMC4598654

[38] Wang, R. F., Cao, W. W. & Cerniglia, C. E. PCR detection and quantitation of predominant anaerobic bacteria in human and animal fecal samples. *Appl. Environ. Microbiol.* **62**, 1242–1247 (1996). 8919784 10.1128/aem.62.4.1242-1247.1996 PMC167889

[39] Suau, A. et al. Fusobacterium prausnitzii and related species represent a dominant group within the human fecal flora. *Syst. Appl. Microbiol.* **24**, 139–145 (2001). 11403393 10.1078/0723-2020-00015

[40] Fite, A. et al. Identification and quantitation of mucosal and faecal desulfovibrios using real time polymerase chain reaction. *Gut* **53**, 523–529 (2004). 15016746 10.1136/gut.2003.031245 PMC1774019

[41] Svedlund, J., Sjodin, I. & Dotevall, G. GSRS—a clinical rating scale for gastrointestinal symptoms in patients with irritable bowel syndrome and peptic ulcer disease. *Dig. Dis. Sci.* **33**, 129–134 (1988). 3123181 10.1007/BF01535722

[42] Bjork, J. et al. Prediction of relative glomerular filtration rate in adults: new improved equations based on Swedish Caucasians and standardized plasma-creatinine assays. *Scand. J. Clin. Lab. Invest.* **67**, 678–695 (2007). 17852799 10.1080/00365510701326891

[43] Djekic, D. et al. Effects of a vegetarian diet on cardiometabolic risk factors, gut microbiota, and plasma metabolome in subjects with ischemic heart disease: a randomized, crossover study. *J. Am. Heart Assoc.* **9**, e016518 (2020). 32893710 10.1161/JAHA.120.016518 PMC7726986

[44] Fagerland, M. W., Lydersen, S. & Laake, P. Recommended confidence intervals for two independent binomial proportions. *Stat. Methods Med. Res.* **24**, 224–254 (2015). 21996567 10.1177/0962280211415469

[45] Bradley, J. V. *Distribution-Free Statistical Tests* 78–80 (Prentice–Hall, 1968).

[46] Good, P. *Permutation Tests: a Practical Guide to Resampling Methods for Testing Hypotheses* 36–37 (Springer, 2000).

[47] Strocchi, A., Furne, J. K. & Levitt, M. D. A modification of the methylene-blue method to measure bacterial sulfide production in feces. *J. Microbiol. Methods* **15**, 75–82 (1992). 10.1016/0167-7012(92)90071-B

[48] Costea, P. I. et al. Towards standards for human fecal sample processing in metagenomic studies. *Nat. Biotechnol.* **35**, 1069–1076 (2017). 28967887 10.1038/nbt.3960

[49] Marmur, J. A procedure for the isolation of deoxyribonucleic acid from micro-organisms. *J. Mol. Biol.* **3**, 208–218 (1961). 10.1016/S0022-2836(61)80047-8

[50] Darling, A. E., Mau, B. & Perna, N. T. progressiveMauve: multiple genome alignment with gene gain, loss and rearrangement. *PLoS ONE* **5**, e11147 (2010). 20593022 10.1371/journal.pone.0011147 PMC2892488

[51] Kumar, S., Stecher, G., Li, M., Knyaz, C. & Tamura, K. MEGA X: molecular evolutionary genetics analysis across computing platforms. *Mol. Biol. Evol.* **35**, 1547–1549 (2018). 29722887 10.1093/molbev/msy096 PMC5967553

[52] Tamura, K., Nei, M. & Kumar, S. Prospects for inferring very large phylogenies by using the neighbor-joining method. *Proc. Natl Acad. Sci. USA* **101**, 11030–11035 (2004). 15258291 10.1073/pnas.0404206101 PMC491989

[53] Langmead, B. & Salzberg, S. L. Fast gapped-read alignment with Bowtie 2. *Nat. Methods* **9**, 357–359 (2012). 22388286 10.1038/nmeth.1923 PMC3322381

[54] Wood, D. E., Lu, J. & Langmead, B. Improved metagenomic analysis with Kraken 2. *Genome Biol.* **20**, 257 (2019). 31779668 10.1186/s13059-019-1891-0 PMC6883579

[55] Lu, J., Breitwieser, F. P., Thielen, P. & Salzbergt, S. L. Bracken: estimating species abundance in metagenomics data. *PeerJ Comput. Sci.* **3**, e104 (2017). 10.7717/peerj-cs.104 PMC12016282 40271438

[56] Karlsson, F. H., Nookaew, I. & Nielsen, J. Metagenomic data utilization and analysis (MEDUSA) and construction of a global gut microbial gene catalogue. *PLoS Comput. Biol.* **10**, e1003706 (2014). 25010449 10.1371/journal.pcbi.1003706 PMC4091689

[57] Vital, M., Howe, A. C. & Tiedje, J. M. Revealing the bacterial butyrate synthesis pathways by analyzing (meta)genomic data. *mBio* **5**, e00889 (2014). 24757212 10.1128/mBio.00889-14 PMC3994512

