# Supplementary material for: Synergy and oxygen adaptation for development of next-generation probiotics
Source: Nature. 2023 Aug 2;620(7973):381–5. doi: 10.1038/s41586-023-06378-w (PMC10412450; doi:10.1038/s41586-023-06378-w)

nature portfolio

https://doi.org/10.1038/s41586-023-06378-w

Supplementary information

Synergy and oxygen adaptation for development of next-generation probiotics

In the format provided by the authors and unedited

Synergy and oxygen adaptation for development of next-generation probiotics

Khan et al.

Table of content

Supplementary Data Table 1	1
Supplementary Data Table 2	4
Supplementary Data Table 3	5
Supplementary Data Table 4	6
Supplementary Data Table 5	7
Supplementary Data Table 6	9
Supplementary Data Table 7	10
META002 CONSORT Checklist	12
META002 RCT Report	14
META002 Protocol (Swedish) Signed	20
META002 Protocol (translation to English)	34
META002 Statistical Analysis Plan	48
META002 Database Lock	62
META002 Statistical Report	63

Supplementary Data Table 1. Demographics and baseline characteristics of the intention to treat (ITT) population.

					Test between groups		
**Variable**	**High + Low dose (n=34)**	**High dose (n=18)**	**Low dose (n=16)**	**Placebo (n=16)**	**High + Low dose vs Placebo**	**High dose vs Placebo**	**Low dose vs Placebo**
Age (years)	29.8 (5.7)<br>30.0 (20; 40)<br>n=34	29.1 (6.3)<br>28.5 (20; 39)<br>n=18	30.5 (4.9)<br>31.0 (21; 40)<br>n=16	26.3 (4.6)<br>25.5 (20; 38)<br>n=16	0.035	0.15	0.020
Sex							
Female	18 (52.9%)	10 (55.6%)	8 (50.0%)	8 (50.0%)			
Male	16 (47.1%)	8 (44.4%)	8 (50.0%)	8 (50.0%)	1.00	1.00	1.00
Body Mass Index (kg/m^2)	24.8 (3.8)<br>24.4 (18.1; 35.7)<br>n=34	25.3 (4.6)<br>24.4 (18.1; 35.7)<br>n=18	24.3 (2.8)<br>24.1 (19.6; 28.1)<br>n=16	23.8 (3.3)<br>23.5 (20.3; 34)<br>n=16	0.37	0.30	0.64
Waist to hip ratio	0.798 (0.078)<br>0.798 (0.63; 0.98)<br>n=34	0.808 (0.093)<br>0.803 (0.63; 0.98)<br>n=18	0.787 (0.059)<br>0.796 (0.692; 0.896)<br>n=16	0.799 (0.087)<br>0.800 (0.688; 1.009)<br>n=16	0.97	0.78	0.65
Systolic blood pressure (mmHg)	117.9 (12.4)<br>116.8 (92; 157)<br>n=34	121.9 (11.9)<br>120.0 (101.5; 157)<br>n=18	113.4 (11.7)<br>113.5 (92; 138.5)<br>n=16	117.2 (8.5)<br>116.8 (102; 139)<br>n=16	0.85	0.20	0.31
Diastolic blood pressure (mmHg)	69.9 (9.0)<br>69.3 (55; 94.5)<br>n=34	71.6 (9.3)<br>70.3 (57.5; 94.5)<br>n=18	68.0 (8.4)<br>68.3 (55; 85)<br>n=16	67.2 (7.1)<br>66.5 (55.5; 83)<br>n=16	0.29	0.13	0.78
GSRS (score 0-45) at randomization	3.0 (0; 12)<br>n=34	2.75 (0; 12)<br>n=18	3.0 (0; 11)<br>n=16	2.5 (1; 7.5)<br>n=16	0.23	0.31	0.24
Blood Biochemistry at screening							
Renal function eGFR (ml/min)	90.6 (10.0)<br>91.0 (71; 115)<br>n=34	90.1 (8.8)<br>91.0 (71; 104)<br>n=18	91.3 (11.4)<br>90.0 (71; 115)<br>n=16	89.6 (9.3)<br>87.5 (76; 117)<br>n=16	0.73	0.88	0.66
Haemoglobin (g/l)	140.2 (13.2)<br>139.5 (118; 164)<br>n=34	140.0 (13.9)<br>139.5 (118; 164)<br>n=18	140.4 (13.0)<br>141.0 (124; 159)<br>n=16	141.8 (14.6)<br>140.0 (123; 169)<br>n=16	0.71	0.72	0.79
Total protein (g/l)	70.5 (4.6)<br>70 (62; 80)<br>n=33	70.9 (4.9)<br>72.5 (62; 80)<br>n=18	70.1 (4.2)<br>70 (64; 78)<br>n=15	72.9 (5.1)<br>73 (62; 81)<br>n=16	0.12	0.27	0.12
Fasting blood glucose (mmol/L)	5.15 (0.40)<br>5.15 (4.5; 6)<br>n=34	5.29 (0.44)<br>5.25 (4.5; 6)<br>n=18	5.00 (0.28)<br>5.00 (4.5; 5.5)<br>n=16	5.21 (0.35)<br>5.25 (4.6; 5.7)<br>n=16	0.68	0.58	0.082
HbA1c (mmol/mol)	30.4 (2.6)<br>30 (26; 39)<br>n=34	30.6 (3.2)<br>30 (26; 39)<br>n=18	30.2 (1.8)<br>30.5 (26; 33)<br>n=16	30.4 (2.0)<br>31 (26; 33)<br>n=16	1.00	0.91	0.84
Erythrocyte sedimentation rate (mm/hour)	4.66 (0.45)<br>4.60 (3.7; 5.4)<br>n=34	4.67 (0.47)<br>4.60 (3.7; 5.4)<br>n=18	4.65 (0.45)<br>4.65 (3.8; 5.4)<br>n=16	4.70 (0.57)<br>4.65 (3.9; 5.6)<br>n=16	0.83	0.90	0.80
C-reactive protein (mg/L)	0.91 (3.63)<br>0 (0; 21)<br>n=34	1.50 (4.94)<br>0 (0; 21)<br>n=18	0.25 (0.68)<br>0 (0; 2)<br>n=16	0.94 (2.27)<br>0 (0; 9)<br>n=16	0.82	0.87	0.36

Red blood cell count (10^9/L)	241.0 (44.1)<br>242 (172; 331)<br>n=34	253.6 (49.9)<br>261 (173; 331)<br>n=18	226.9 (32.5)<br>229 (172; 295)<br>n=16	238.1 (41.8)<br>235 (175; 324)<br>n=16	0.82	0.34	0.41
White blood cell count (10^9/L)	4.73 (1.16)<br>4.50 (2.9; 7.1)<br>n=34	4.83 (1.28)<br>4.65 (2.9; 7.1)<br>n=18	4.61 (1.03)<br>4.15 (3.6; 6.8)<br>n=16	4.91 (0.87)<br>5.05 (3.6; 6.7)<br>n=16	0.59	0.86	0.39
Platelet count (10^9/L)	6.03 (6.74)<br>3 (1; 29)<br>n=34	7.33 (8.35)<br>3 (1; 29)<br>n=18	4.56 (4.10)<br>3 (1; 15)<br>n=16	6.69 (4.29)<br>5 (2; 16)<br>n=16	0.73	0.79	0.17
Alanine transaminase (µkat/L)	0.35 (0.151)<br>0.32 (0.13; 0.77)<br>n=34	0.41 (0.175)<br>0.35 (0.21; 0.77)<br>n=18	0.29 (0.09)<br>0.30 (0.13; 0.47)<br>n=16	0.42 (0.20)<br>0.35 (0.19; 0.83)<br>n=16	0.21	0.86	0.028
Aspartate transaminase (µkat/L)	0.42 (0.157)<br>0.37 (0.28; 1.2)<br>n=34	0.43 (0.207)<br>0.36 (0.28; 1.2)<br>n=18	0.40 (0.073)<br>0.38 (0.29; 0.55)<br>n=16	0.47 (0.123)<br>0.43 (0.26; 0.69)<br>n=16	0.28	0.53	0.069
Alkaline phosphatase (µkat/L)	0.99 (0.23)<br>0.94 (0.65; 1.6)<br>n=34	0.92 (0.15)<br>0.92 (0.68; 1.3)<br>n=18	1.06 (0.28)<br>0.99 (0.65; 1.6)<br>n=16	1.15 (0.38)<br>1.10 (0.44; 1.6)<br>n=16	0.076	0.030	0.45
Bilirubin (µmol/L) at screening	11.4 (8.0)<br>9.0 (3.8; 37)<br>n=34	10.4 (7.7)<br>8.1 (3.8; 37)<br>n=18	12.5 (8.5)<br>9.5 (5.3; 37)<br>n=16	10.5 (5.0)<br>9.5 (3.7; 23)<br>n=16	0.73	0.95	0.45
Colonization with F. prausnitzii in stool at randomization (strain)							
Colonization with *F. prausnitzii* (SL3.3)	399113 (300581)<br>261943 (64423; 1322383)<br>n=30	486085 (381409)<br>386948 (87115; 1322383)<br>n=14	323012 (188047)<br>250282 (64423; 648169)<br>n=16	447790 (427354)<br>344404 (65739; 1495280)<br>n=13	0.65	0.82	0.32
Colonization with *F. prausnitzii* (L2.6)	445500 (455431)<br>296051 (97458; 2395226)<br>n=30	473748 (604906)<br>282870 (97458; 2395226)<br>n=14	420783 (287205)<br>298545 (155219; 1110434)<br>n=16	407073 (270661)<br>334194 (105446; 910201)<br>n=13	0.88	0.82	0.90
Colonization with *F.prausnitzii* (KLE1255)	421180 (455384)<br>317422 (56316; 2104040)<br>n=30	388569 (440720)<br>298151 (56316; 1795932)<br>n=14	449714 (480340)<br>325233 (64786; 2104040)<br>n=16	462245 (308874)<br>349653 (147476; 1214343)<br>n=13	0.74	0.65	0.92
Colonization with *F. prausnitzii* (A2.165)	475369 (558059)<br>282274 (68859; 2981157)<br>n=30	406020 (320415)<br>275428 (117096; 1027944)<br>n=14	536050 (710426)<br>292417 (68859; 2981157)<br>n=16	447652 (240108)<br>515762 (108885; 882020)<br>n=13	0.98	0.72	0.86
Colonization with F. *prausnitzii* (Total)	1741162 (1032122)<br>1492610 (503848; 4691391)<br>n=30	1754422 (1020550)<br>1492610 (532972; 4691391)<br>n=14	1729559 (1075441)<br>1500193 (503848; 4531826)<br>n=16	1764760 (783823)<br>1791817 (533480; 3106899)<br>n=13	0.91	0.98	0.91
Stool short chain fatty acids (nmol/mg dry weight) at randomisation							
Butyrate, nmol/mg	54.0 (43.2)<br>40.1 (6.2; 221.3)<br>n=32	56.7 (52.5)<br>33.2 (6.2; 221.3)<br>n=16	51.4 (33.0)<br>41.6 (11.7; 122.7)<br>n=16	85.3 (60.9)<br>70.1 (23.8; 241.4)<br>n=14	0.064	0.18	0.064
Propionate, nmol/mg	83.0 (75.1)<br>58.3 (10.7; 393.5)<br>n=32	76.2 (53.2)<br>64.5 (10.7; 190)<br>n=16	89.7 (93.5)<br>51.1 (17.2; 393.5)<br>n=16	155.2 (174.8)<br>79.5 (27.9; 651.5)<br>n=14	0.074	0.090	0.22

Acetate, nmol/mg	222.4 (120.5)<br>207.7 (56.8; 597.7)<br>n=32	222.0 (130.2)<br>203.4 (56.8; 597.7)<br>n=16	222.9 (114.1)<br>207.7 (71.2; 485.5)<br>n=16	352.6 (213.3)<br>293.3 (98; 719.3)<br>n=14	0.015	0.047	0.044
Lactate, nmol/mg	0.458 (0.418)<br>0.262 (0.106; 1.419)<br>n=32	0.454 (0.429)<br>0.22 (0.118; 1.367)<br>n=16	0.461 (0.421)<br>0.308 (0.106; 1.419)<br>n=16	0.980 (1.446)<br>0.494 (0.098; 5.712)<br>n=14	0.086	0.16	0.18
Isobutyrate, nmol/mg	10.5 (4.7)<br>9.8 (2.8; 20.6)<br>n=32	10.0 (4.8)<br>9.7 (2.8; 20.6)<br>n=16	11.1 (4.7)<br>9.8 (4.6; 19)<br>n=16	10.4 (6.0)<br>9.3 (2.8; 27.7)<br>n=14	0.96	0.83	0.75
Isovalerate, nmol/mg	8.26 (3.50)<br>8.37 (2.51; 14.79)<br>n=32	7.96 (3.65)<br>8.12 (2.51; 14.27)<br>n=16	8.56 (3.43)<br>8.47 (3.65; 14.79)<br>n=16	7.61 (4.98)<br>6.88 (1.61; 23)<br>n=14	0.64	0.85	0.58
Succinate, nmol/mg	3.39 (14.19)<br>0.78 (0.15; 81.05)<br>n=32	0.911 (0.871)<br>0.681 (0.189; 3.104)<br>n=16	5.86 (20.06)<br>0.91 (0.15; 81.05)<br>n=16	28.4 (89.8)<br>0.9 (0.2; 337.3)<br>n=14	0.21	0.17	0.49

For categorical variables n (%) is presented. For continuous variables Mean (SD) (top) and Median (Min; Max) / and n are presented. For comparison between groups Fisher´s Exact test (lowest 1-sided p-value multiplied by 2) was used for dichotomous variables and the Fisher´s Non-Parametric Permutation Test was used for continuous variables.

Supplementary Data Table 2. Primary tolerability analysis - discontinuation due to investigational product.

					Difference and test between groups		
	**High + Low dose (n=34)**	**High dose (n=18)**	**Low dose (n=16)**	**Placebo (n=16)**	**High + Low dose vs Placebo**	**High dose vs Placebo**	**Low dose vs Placebo**
Discontinuation of treatment	0	0	0	0	0.0 (-0.19; 0.10)	0.0 (-0.19; 0.18)	0.0 (-0.19; 0.19)

IP=Investigational product. Analysis in the intention to treat population. High and low dose=10^9-$5x10^9$ CFU/bacteria strain and 10^8-$5x10^8$

Supplementary Data Table 3. Distribution of adverse events in the intention to treat (ITT) population

	Treatment Allocation					
	**High dose** (n=18)		**Low dose** (n=16)		**Placebo** (n=16)	
	Events	Subjects with events n (%)	Events	Subjects with Events n (%)	Events	Subjects with events n (%)
**Any adverse event**	**26**	**13 (72.2%)**	**14**	**7 (43.8%)**	**12**	**10 (62.5%)**
**Any adverse event related to IP**		**6 (33.3%)**		**6 (37.5%)**		**6 (37.5%)**
**Gastrointestinal disorders**	**14**	**6 (33.3%)**	**9**	**6 (37.5%)**	**6**	**6 (37.5%)**
Dyspepsia NOS	1	1 (5.6%)				
Change in bowel habit NOS	13	6 (33.3%)	9	6 (37.5%)	6	6 (37.5%)
**General disorders and administration site conditions**	**3**	**2 (11.1%)**	**3**	**3 (18.8%)**	**2**	**2 (12.5%)**
Abnormal liver enzyme levels					1	1 (6.3%)
Headache	1	1 (5.6%)	2	2 (12.5%)		
Pain					1	1 (6.3%)
Tiredness	2	1 (5.6%)	1	1 (6.3%)		
**Infections and infestations**	**2**	**2 (11.1%)**			**1**	**1 (6.3%)**
**Injury, poisoning and procedural complications**	**1**	**1 (5.6%)**				
Sprain and strain of ankle	1	1 (5.6%)				
**Psychiatric disorder - Insomnia**	**1**	**1 (5.6%)**				
**Reproductive system and breast disorders**	**1**	**1 (5.6%)**	**1**	**1 (6.3%)**		
Dysmenorrhoea, unspecified			1	1 (6.3%)		
Irregular menstruation, unspecified	1	1 (5.6%)				
**Respiratory, thoracic and mediastinal disorders**	**4**	**4 (22.2%)**	**1**	**1 (6.3%)**	**3**	**3 (18.8%)**
Acute nasopharyngitis [common cold]	3	3 (16.7%)	1	1 (6.3%)	3	3 (18.8%)
Acute sinusitis, unspecified	1	1 (5.6%)				

Adverse events presented as number of events for all three groups (low and high dose as well as placebo) together with number of subjects with events for each group. IP=Investigational Product. High and low dose=10^9-$5x10^9$ CFU/bacteria strain and 10^8-$5x10^8$ CFU twice daily of *D. piger* and *F. praunitzii,* respectively. NOS=Not otherwise specified.

Supplementary Data Table 4. Change in gastrointestinal symptoms (GSRS) and effects of the investigational product vs. placebo in the intention to treat (ITT) population.

					Difference and test between groups		
**Variable (change)**	**High + Low dose (n=34)**	**High dose (n=18)**	**Low dose (n=16)**	**Placebo (n=16)**	**High + Low dose vs Placebo**	**High dose vs Placebo**	**Low dose vs Placebo**
Change in GSRS (score 0-45) from randomization to week 4	0.047 (2.525)<br>0 (-5; 4.5)<br>n=32	0.000 (2.751)<br>-0.25 (-5; 4.5)<br>n=16	0.094 (2.368)<br>0 (-4; 4)<br>n=16	0.929 (3.710)<br>-0.25 (-2.5; 11)<br>n=14	-0.882 (-2.667; 1.111)<br>p=0.37	-0.929 (-3.300; 1.500)<br>p=0.48	-0.835 (-3.071; 1.437)<br>p=0.50
Change in GSRS (score 0-45) from randomization to week 8	-0.156 (3.298)<br>-0.25 (-5.5; 9)<br>n=32	-0.219 (3.728)<br>-0.25 (-5.5; 9)<br>n=16	-0.094 (2.928)<br>-0.25 (-4; 7.5)<br>n=16	-0.607 (2.816)<br>-1 (-4.5; 6)<br>n=14	0.451 (-1.500; 2.545)<br>p=0.68	0.388 (-2.083; 2.917)<br>p=0.77	0.513 (-1.611; 2.667)<br>p=0.65

For continuous variables Mean (SD) / Median (Min; Max) / n= is presented. For comparison between groups the two-sided Fisher´s Non-Parametric Permutation Test was used for continuous variables. The confidence interval for the mean difference between groups is based on Fishers non-parametric permutation test. IP=Investigational Product. High and low dose=10^9-$5x10^9$ CFU/bacteria strain and 10^8-$5x10^8$ CFU twice daily of *D. piger* and *F. praunitzii*, respectively. GSRS= Gastrointestinal Symptom Rating Scale.

Supplementary Data Table 5. Change in blood biochemistry variables and effects of the investigational product vs. placebo in the intention to treat (ITT) population.

					Difference between groups		
**Variable (change)**	**High + Low dose (n=34)**	**High dose (n=18)**	**Low dose (n=16)**	**Placebo (n=16)**	**High + Low dose vs Placebo**	**High dose vs Placebo**	**Low dose vs Placebo**
Change in Erythrocyte sedimentation rate (mm/hour) from screening to week 4	0.009 (0.212)<br>0 (-0.5; 0.4)<br>n=32	0.025 (0.205)<br>-0.05 (-0.2; 0.4)<br>n=16	-0.006 (0.224)<br>0 (-0.5; 0.4)<br>n=16	-0.079 (0.219)<br>0 (-0.5; 0.2)<br>n=14	0.088 (-0.050; 0.225)<br>p=0.23	0.104 (-0.057; 0.262)<br>p=0.22	0.072 (-0.100; 0.237)<br>p=0.43
Change in Erythrocyte sedimentation rate (mm/hour) from screening to week 8	0.056 (0.203)<br>0.1 (-0.4; 0.5)<br>n=32	0.081 (0.201)<br>0.1 (-0.3; 0.5)<br>n=16	0.031 (0.209)<br>0.1 (-0.4; 0.3)<br>n=16	-0.021 (0.249)<br>0 (-0.4; 0.3)<br>n=14	0.078 (-0.064; 0.212)<br>p=0.30	0.103 (-0.067; 0.271)<br>p=0.25	0.053 (-0.125; 0.225)<br>p=0.59
Change in C-reactive protein (mg/L) from screening to week 4	0.250 (4.280)<br>0 (-15; 18)<br>n=32	0.500 (6.088)<br>0 (-15; 18)<br>n=16	0.000 (0.816)<br>0 (-2; 2)<br>n=16	-0.071 (0.917)<br>0 (-2; 2)<br>n=14	0.321 (-1.778; 2.571)<br>p=0.68	0.571 (-2.333; 3.667)<br>p=0.59	0.071 (-0.571; 0.714)<br>p=1.00
Change in C-reactive protein (mg/L) from screening to week 8	-0.219 (2.871)<br>0 (-15; 3)<br>n=32	-0.250 (4.058)<br>0 (-15; 3)<br>n=16	-0.188 (0.750)<br>0 (-2; 1)<br>n=16	-0.071 (2.235)<br>0 (-6; 4)<br>n=14	-0.147 (-2.000; 1.222)<br>p=1.00	-0.179 (-2.600; 1.875)<br>p=0.99	-0.116 (-1.286; 1.000)<br>p=0.94
Change in Red blood cell count (10^9/L) from screening to week 4	19.5 (31.3)<br>11.5 (-20; 100)<br>n=32	27.1 (37.5)<br>23.5 (-20; 100)<br>n=16	11.9 (22.3)<br>8.0 (-14; 69)<br>n=16	12.6 (25.5)<br>9.5 (-36; 71)<br>n=14	6.83 (-11.42; 26.50)<br>p=0.50	14.4 (-9.4; 38.6)<br>p=0.24	-0.768 (-18.667; 17.167)<br>p=0.93
Change in Red blood cell count (10^9/L) from screening to week 8	13.1 (25.8)<br>7.0 (-41; 66)<br>n=32	18.9 (29.0)<br>16.0 (-41; 66)<br>n=16	7.31 (21.56)<br>0.0 (-22; 46)<br>n=16	5.79 (39.50)<br>15.0 (-90; 88)<br>n=14	7.34 (-12.87; 26.92)<br>p=0.46	13.2 (-12.5; 38.6)<br>p=0.32	1.53 (-22.00; 24.67)<br>p=0.90
Change in White blood cell count (10^9/L) from screening to week 4	0.609 (0.798)<br>0.45 (-0.5; 3)<br>n=32	0.625 (0.770)<br>0.45 (-0.5; 2.2)<br>n=16	0.594 (0.850)<br>0.45 (-0.5; 3)<br>n=16	0.514 (1.156)<br>0.4 (-1.4; 2.8)<br>n=14	0.095 (-0.475; 0.700)<br>p=0.76	0.111 (-0.617; 0.833)<br>p=0.78	0.079 (-0.662; 0.837)<br>p=0.85
Change in White blood cell count (10^9/L) from screening to week 8	0.694 (0.998)<br>0.6 (-1.1; 3)<br>n=32	0.988 (1.110)<br>0.8 (-0.8; 3)<br>n=16	0.400 (0.802)<br>0.4 (-1.1; 1.8)<br>n=16	0.500 (1.173)<br>0.1 (-0.9; 2.8)<br>n=14	0.194 (-0.487; 0.891)<br>p=0.59	0.487 (-0.360; 1.350)<br>p=0.26	-0.100 (-0.850; 0.650)<br>p=0.80
Change in Platelet count (10^9/L) from screening to week 4	1.000 (4.853)<br>0.0 (-6; 20)<br>n=32	1.56 (5.81)<br>0.0 (-6; 20)<br>n=16	0.438 (3.777)<br>0.0 (-6; 11)<br>n=16	-1.430 (2.24)<br>-1.0 (-7; 1)<br>n=14	2.43 (0.00; 5.25)<br>p=0.053	2.99 (0.00; 6.33)<br>p=0.059	1.87 (-0.43; 4.25)<br>p=0.13
Change in Platelet count (10^9/L) from screening to week 8	-0.250 (2.185)<br>0.0 (-7; 3)<br>n=32	0.375 (1.455)<br>0.0 (-3; 3)<br>n=16	-0.875 (2.630)<br>0.0 (-7; 3)<br>n=16	-0.857 (3.655)<br>-0.5 (-7; 5)<br>n=14	0.607 (-1.200; 2.375)<br>p=0.52	1.23 (-0.83; 3.29)<br>p=0.26	-0.018 (-2.429; 2.333)<br>p=1.00
Change in Alanine transaminase (µkat/L) from screening to week 4	-0.026 (0.084)<br>-0.01 (-0.28; 0.11)<br>n=32	-0.039 (0.110)<br>-0.015 (-0.28; 0.11)<br>n=16	-0.013 (0.046)<br>-0.01 (-0.15; 0.05)<br>n=16	-0.031 (0.148)<br>-0.025 (-0.39; 0.31)<br>n=14	0.006 (-0.065; 0.076)<br>p=0.87	-0.007 (-0.104; 0.090)<br>p=0.89	0.019 (-0.058; 0.097)<br>p=0.65
Change in Alanine transaminase (µkat/L) from screening to week 8	0.005 (0.102)<br>-0.01 (-0.23; 0.33)<br>n=32	0.006 (0.137)<br>-0.01 (-0.23; 0.33)<br>n=16	0.004 (0.054)<br>-0.015 (-0.07; 0.12)<br>n=16	-0.029 (0.203)<br>-0.025 (-0.44; 0.51)<br>n=14	0.034 (-0.057; 0.129)<br>p=0.46	0.036 (-0.092; 0.162)<br>p=0.60	0.033 (-0.071; 0.137)<br>p=0.57
Change in Aspartate transaminase (µkat/L) from screening to week 4	-0.034 (0.158)<br>-0.015 (-0.8; 0.17)<br>n=32	-0.053 (0.215)<br>-0.015 (-0.8; 0.17)<br>n=16	-0.016 (0.068)<br>-0.01 (-0.12; 0.13)<br>n=16	-0.051 (0.083)<br>-0.055 (-0.21; 0.11)<br>n=14	0.017 (-0.078; 0.087)<br>p=0.66	-0.002 (-0.120; 0.101)<br>p=0.98	0.035 (-0.021; 0.092)<br>p=0.22
Change in Aspartate transaminase (µkat/L) from screening to week 8	-0.028 (0.149)<br>-0.005 (-0.73; 0.12)<br>n=32	-0.026 (0.202)<br>0.02 (-0.73; 0.12)<br>n=16	-0.029 (0.070)<br>-0.03 (-0.18; 0.07)<br>n=16	-0.042 (0.107)<br>-0.025 (-0.22; 0.12)<br>n=14	0.015 (-0.081; 0.090)<br>p=0.69	0.016 (-0.103; 0.123)<br>p=0.83	0.013 (-0.053; 0.080)<br>p=0.69

Change in Alkaline phosphatase (µkat/L) from screening to week 4	0.015 (0.113)<br>0.005 (-0.3; 0.2)<br>n=32	0.013 (0.107)<br>0.045 (-0.2; 0.2)<br>n=16	0.016 (0.122)<br>0 (-0.3; 0.2)<br>n=16	-0.047 (0.159)<br>0 (-0.4; 0.28)<br>n=14	0.062 (-0.021; 0.143)<br>p=0.14	0.060 (-0.040; 0.161)<br>p=0.24	0.063 (-0.041; 0.167)<br>p=0.23
Change in Alkaline phosphatase (µkat/L) from screening to week 8	0.021 (0.109)<br>0.015 (-0.22; 0.21)<br>n=32	0.027 (0.089)<br>0.03 (-0.22; 0.2)<br>n=16	0.014 (0.130)<br>0.00 (-0.2; 0.21)<br>n=16	-0.056 (0.147)<br>-0.01 (-0.4; 0.14)<br>n=14	0.077 (-0.002; 0.155)<br>p=0.060	0.083 (-0.006; 0.174)<br>p=0.069	0.071 (-0.034; 0.176)<br>p=0.18
Change in Bilirubin (µmol/L) from screening to week 4	-0.925 (5.923)<br>0.00 (-24; 11)<br>n=32	-1.840 (6.49)<br>-0.15 (-24; 4)<br>n=16	-0.006 (5.351)<br>0.10 (-12; 11)<br>n=16	-0.657 (3.922)<br>-1.35 (-5.8; 9.3)<br>n=14	-0.268 (-3.910; 2.933)<br>p=0.92	-1.19 (-5.22; 2.49)<br>p=0.63	0.651 (-2.925; 4.114)<br>p=0.72
Change in Bilirubin (µmol/L) from screening to week 8	-1.200 (5.15)<br>0.0 (-19; 5.9)<br>n=32	-1.250 (5.18)<br>0.4 (-14; 5.9)<br>n=16	-1.160 (5.30)<br>0.0 (-19; 5)<br>n=16	-0.236 (5.372)<br>-1.0 (-7.8; 11.3)<br>n=14	-0.967 (-4.470; 2.262)<br>p=0.57	-1.01 (-5.08; 2.90)<br>p=0.61	-0.921 (-4.983; 2.920)<br>p=0.67
Change in eGFR (ml/min) from screening to week 4	0.688 (6.332)<br>0.5 (-10; 14)<br>n=32	-0.375 (6.270)<br>0.0 (-10; 11)<br>n=16	1.750 (6.41)<br>2.0 (-8; 14)<br>n=16	2.210 (6.99)<br>4.5 (-12; 12)<br>n=14	-1.53 (-5.80; 2.70)<br>p=0.49	-2.59 (-7.50; 2.33)<br>p=0.30	-0.464 (-5.500; 4.500)<br>p=0.88
Change in eGFR (ml/min) from screening to week 8	0.281 (5.687)<br>0.5 (-14; 13)<br>n=32	1.000 (5.610)<br>1.0 (-9; 13)<br>n=16	-0.438 (5.853)<br>-1.0 (-14; 9)<br>n=16	1.860 (5.32)<br>2.0 (-8; 10)<br>n=14	-1.58 (-5.22; 2.00)<br>p=0.40	-0.857 (-4.875; 3.250)<br>p=0.69	-2.29 (-6.50; 1.87)<br>p=0.29
Change in Total protein (g/l) from screening to week 4	1.000 (4.457)<br>1.0 (-8; 13)<br>n=31	1.380 (5.28)<br>1.0 (-8; 13)<br>n=16	0.600 (3.521)<br>0.0 (-4; 8)<br>n=15	-1.210 (5.55)<br>-1.0 (-10; 6)<br>n=14	2.21 (-0.92; 5.33)<br>p=0.17	2.59 (-1.43; 6.67)<br>p=0.22	1.81 (-1.67; 5.33)<br>p=0.31
Change in Total protein (g/l) from screening to week 8	0.742 (3.473)<br>1.0 (-9; 6)<br>n=31	1.810 (2.93)<br>2.0 (-3; 6)<br>n=16	-0.400 (3.738)<br>-1.0 (-9; 5)<br>n=15	-1.000 (4.574)<br>-0.5 (-8; 4)<br>n=14	1.74 (-0.86; 4.22)<br>p=0.19	2.81 (0.00; 5.71)<br>p=0.061	0.600 (-2.571; 3.833)<br>p=0.72
Change in Haemoglobin (g/l) from screening to week 4	-0.125 (5.988)<br>-1.0 (-13; 14)<br>n=32	0.063 (6.060)<br>-1.5 (-8; 12)<br>n=16	-0.313 (6.107)<br>-0.5 (-13; 14)<br>n=16	-1.430 (5.33)<br>-0.5 (-12; 6)<br>n=14	1.30 (-2.44; 5.00)<br>p=0.50	1.49 (-2.80; 5.71)<br>p=0.51	1.12 (-3.17; 5.43)<br>p=0.63
Change in Haemoglobin (g/l) from screening to week 8	0.969 (6.498)<br>1.5 (-14; 17)<br>n=32	2.000 (6.35)<br>0.0 (-10; 17)<br>n=16	-0.063 (6.688)<br>2.5 (-14; 7)<br>n=16	-1.570 (7.08)<br>-0.5 (-16; 8)<br>n=14	2.54 (-1.80; 6.78)<br>p=0.25	3.57 (-1.43; 8.62)<br>p=0.16	1.51 (-3.78; 6.67)<br>p=0.57

For continuous variables Mean (SD) (top) and Median (Min; Max) / n= are presented. For comparison between groups the two-sided Fisher´s Non-Parametric Permutation Test was used for continuous variables. The confidence interval for the mean difference between groups is based on Fishers non-parametric permutation test. IP=Investigational Product. High and low dose=10^9-$5x10^9$ CFU/bacteria strain and 10^8-$5x10^8$ CFU twice daily of *D. piger and F. praunitzii*, respectively. eGFR=estimated Glomerular Filtration Rate.

Supplementary Data Table 6. Change in blood glucose variables and effects of the investigational product vs. placebo in the intention to treat (ITT) population.

					Difference between groups		
**Variable (change)**	**High + Low dose (n=34)**	**High dose (n=18)**	**Low dose (n=16)**	**Placebo (n=16)**	**High + Low dose vs Placebo**	**High dose vs Placebo**	**Low dose vs Placebo**
Change in Fasting blood glucose (mmol/L) from screening to week 4	0.116 (0.336)<br>0.05 (-0.4; 1)<br>n=32	0.056 (0.333)<br>-0.05 (-0.4; 0.6)<br>n=16	0.175 (0.340)<br>0.15 (-0.2; 1)<br>n=16	0.100 (0.306)<br>0.10 (-0.6; 0.6)<br>n=14	0.016 (-0.189; 0.229)<br>p=0.93	-0.044 (-0.283; 0.200)<br>p=0.76	0.075 (-0.167; 0.320)<br>p=0.58
Change in Fasting blood glucose (mmol/L) from screening to week 8	0.028 (0.368)<br>0.00 (-0.7; 0.7)<br>n=32	-0.044 (0.379)<br>-0.10 (-0.7; 0.7)<br>n=16	0.100 (0.354)<br>0.00 (-0.5; 0.7)<br>n=16	0.021 (0.340)<br>0.15 (-0.7; 0.5)<br>n=14	0.007 (-0.222; 0.240)<br>p=0.98	-0.065 (-0.333; 0.200)<br>p=0.66	0.079 (-0.180; 0.340)<br>p=0.58
Change in Blood HbA1c (mmol/mol) from screening to week 4	0.063 (1.523)<br>0.0 (-3; 3)<br>n=32	0.500 (1.211)<br>1.0 (-2; 3)<br>n=16	-0.375 (1.708)<br>0.0 (-3; 2)<br>n=16	-0.357 (1.646)<br>0.0 (-3; 2)<br>n=14	0.420 (-0.625; 1.400)<br>p=0.46	0.857 (-0.200; 2.000)<br>p=0.15	-0.018 (-1.286; 1.250)<br>p=1.00
Change in Blood HbA1c (mmol/mol) from screening to week 8	-0.344 (1.359)<br>0.0 (-3; 2)<br>n=32	-0.063 (1.124)<br>0.0 (-2; 2)<br>n=16	-0.625 (1.544)<br>-1.0 (-3; 2)<br>n=16	-0.500 (1.871)<br>-0.5 (-3; 3)<br>n=14	0.156 (-0.818; 1.143)<br>p=0.84	0.438 (-0.714; 1.600)<br>p=0.51	-0.125 (-1.429; 1.167)<br>p=0.92

For continuous variables Mean (SD) (top) and Median (Min; Max) / n= are presented. For comparison between groups the two-sided Fisher´s Non-Parametric Permutation Test was used for continuous variables. The confidence interval for the mean difference between groups is based on Fishers non-parametric permutation test. IP=Investigational Product. High and low dose=10^9-$5x10^9$ CFU/bacteria strain and 10^8-$5x10^8$ CFU twice daily of *D. piger* and *F. praunitzii*, respectively. HbA1c=glycosylated hemoglobin.

Supplementary Data Table 7. Change in stool SCFAs and effects of high or low dose of the investigational product vs. placebo in the intention of teat (ITT) population.

					Difference between groups		
**Variable (relative change, %)**	**High + Low dose (n=34)**	**High dose (n=18)**	**Low dose (n=16)**	**Placebo (n=16)**	**High + Low dose vs Placebo**	**High dose vs Placebo**	**Low dose vs Placebo**
**Butyrate** Randomization to week 4	85.1 (160.3)<br>22 (-83.8; 522.3)<br>n=31	53.3 (144.2)<br>18 (-83.8; 396.5)<br>n=15	114.9 (173.2)<br>81.4 (-58.2; 522.3)<br>n=16	71.0 (144.7)<br>20.9 (-75.6; 433)<br>n=14	14.0 (-83.1; 119.6)<br>p=0.80	-17.7 (-127.7; 93.4)<br>p=0.75	43.8 (-75.0; 165.9)<br>p=0.45
**Butyrate** Randomization to week 8	128.2 (429.8)<br>1.5 (-75.3; 2239.4)<br>n=30	32.8 (127.1)<br>-22.2 (-75.3; 309.2)<br>n=14	211.7 (572.0)<br>14.9 (-58.6; 2239.4)<br>n=16	53.4 (131.3)<br>-0.1 (-81.8; 351.8)<br>n=13	74.8 (-82.7; 335.7)<br>p=0.74	-20.6 (-123.7; 81.1)<br>p=0.68	158.3 (-79.2; 472.6)<br>p=0.43
**Butyrate** Randomization to week 10	177.2 (575.9)<br>39.5 (-86.3; 3229.9)<br>n=32	68.7 (173.7)<br>12.2 (-70; 605.3)<br>n=16	285.7 (793.8)<br>72.5 (-86.3; 3229.9)<br>n=16	2.84 (58.79)<br>-3.07 (-84.3; 120.3)<br>n=14	174.4 (6.7; 495.5)<br>p=0.030	65.9 (-23.3; 163.9)<br>p=0.18	282.9 (25.2; 665.6)<br>p=0.0093
**Propionate,** Randomization to week 4	51.5 (143.7)<br>0.3 (-61.3; 677.5)<br>n=31	39.6 (100.5)<br>2.5 (-61.3; 250)<br>n=15	62.7 (177.7)<br>-3.5 (-43.5; 677.5)<br>n=16	21.1 (85.0)<br>-15.8 (-80.6; 184.1)<br>n=14	30.4 (-40.5; 120.0)<br>p=0.51	18.5 (-51.9; 91.2)<br>p=0.60	41.6 (-49.9; 146.4)<br>p=0.52
**Propionate** Randomization to week 8	33.2 (111.0)<br>0.8 (-75.9; 413)<br>n=30	36.7 (138.1)<br>-21.1 (-74.3; 413)<br>n=14	30.2 (85.3)<br>2.4 (-75.9; 228.3)<br>n=16	41.0 (118.3)<br>33.1 (-87.9; 375.5)<br>n=13	-7.82 (-78.5; 72.1)<br>p=0.81	-4.32 (-104.7; 97.3)<br>p=0.94	-10.9 (-86.0; 65.0)<br>p=0.77
**Propionate** Randomization to week 10	59.8 (132.5)<br>22.7 (-83.7; 615.3)<br>n=32	34.9 (81.2)<br>21.0 (-61.7; 256.6)<br>n=16	84.7 (168.5)<br>46.9 (-83.7; 615.3)<br>n=16	-12.6 (38.7)<br>-13.7 (-64.5; 57.5)<br>n=14	72.4 (11.0; 149.6)<br>p=0.016	47.5 (1.2; 95.9)<br>p=0.044	97.3 (14.1; 188.8)<br>p=0.017
**Acetate** Randomization to week 4	45.4 (117.3)<br>25.7 (-54.8; 522.3)<br>n=31	40.6 (92.5)<br>12.7 (-54.8; 268.4)<br>n=15	50.0 (139.6)<br>31.8 (-54.3; 522.3)<br>n=16	16.1 (77.6)<br>-6.4 (-83.4; 224.3)<br>n=14	29.3 (-31.5; 103.2)<br>p=0.43	24.5 (-39.6; 90.9)<br>p=0.45	33.9 (-42.7; 120.2)<br>p=0.49
**Acetate** Randomization to week 8	36.6 (94.4)<br>19.3 (-69.2; 296.7)<br>n=30	25.0 (100.1)<br>-7 (-69.2; 296.7)<br>n=14	46.8 (91.1)<br>26.9 (-62.9; 262.9)<br>n=16	29.6 (87.9)<br>21.3 (-83; 248)<br>n=13	7.00 (-52.7; 70.2)<br>p=0.85	-4.64 (-78.4; 69.7)<br>p=0.90	17.2 (-50.2; 86.1)<br>p=0.61
**Acetate** Randomization to week 10	54.0 (105.5)<br>35.7 (-84.3; 380.3)<br>n=32	25.1 (66.3)<br>14.9 (-56.5; 144.8)<br>n=16	83.0 (129.8)<br>45.6 (-84.3; 380.3)<br>n=16	-12.0 (38.2)<br>-17.8 (-62.9; 38.6)<br>n=14	66.0 (12.8; 127.6)<br>p=0.0094	37.1 (-3.6; 78.7)<br>p=0.072	95.0 (25.4; 169.2)<br>p=0.0042
**Lactate** Randomization to week 4	447.5 (773.3)<br>252.5 (-87.2; 3082.5)<br>n=30	297.5 (404.6)<br>272.8 (-87.2; 1426.7)<br>n=15	598.0 (1013)<br>232.0 (-83; 3083)<br>n=15	198.5 (371.8)<br>74.1 (-73.9; 1352.2)<br>n=14	249.0 (-114.4; 718.5)<br>p=0.28	98.9 (-194.6; 392.4)<br>p=0.51	399.1 (-145.0; 991.8)<br>p=0.23
**Lactate** Randomization to week 8	124.0 (353.6)<br>36.5 (-47.6; 1908.3)<br>n=30	53.1 (124.7)<br>20.3 (-47.6; 378.1)<br>n=14	186.0 (468.5)<br>48.0 (-31.2; 1908.3)<br>n=16	137.1 (364.7)<br>-2.8 (-97.3; 1301.3)<br>n=13	-13.1 (-192.0; 257.7)<br>p=0.91	-83.9 (-281.3; 93.9)<br>p=0.59	48.9 (-225.2; 375.2)<br>p=0.71
**Lactate** Randomization to week 10	338.4 (388.5)<br>235.1 (-93.9; 1395.9)<br>n=31	326.9 (343.4)<br>321.1 (-93.9; 881.7)<br>n=16	350.6 (443.6)<br>173.3 (-79.3; 1395.9)<br>n=15	342.6 (447.8)<br>185.6 (-79.3; 1346.3)<br>n=14	-4.22 (-264.1; 266.9)<br>p=0.96	-15.7 (-314.3; 281.7)<br>p=0.90	8.04 (-326.28; 350.1)<br>p=0.96
**Isobutyrate** Randomization to week 4	30.7 (60.0)<br>14.7 (-44.1; 188)<br>n=31	48.8 (67.9)<br>32.3 (-41.1; 188)<br>n=15	13.7 (47.4)<br>-6.3 (-44.1; 110.1)<br>n=16	55.1 (133.4)<br>18.9 (-78.7; 439)<br>n=14	-24.4 (-79.0; 37.2)<br>p=0.42	-6.29 (-85.3; 69.62)<br>p=0.88	-41.4 (-111.8; 28.2)<br>p=0.30
**Isobutyrate** Randomization to week 8	21.3 (70.2)<br>-3.9 (-60.1; 189.2)<br>n=30	36.6 (86.9)<br>17.1 (-55.1; 189.2)<br>n=14	7.95 (50.78)<br>-4.9 (-60.11; 100.62)<br>n=16	17.4 (61.1)<br>-4.1 (-71.7; 142.8)<br>n=13	3.89 (-39.9; 50.1)<br>p=0.89	19.2 (-40.5; 79.4)<br>p=0.51	-9.50 (-51.9; 32.82)<br>p=0.64

**Isobutyrate**<br>Randomization to week 10	32.2 (83.4)<br>19.6 (-85; 268.1)<br>n=32	33.9 (85.0)<br>18.6 (-62.1; 250.6)<br>n=16	30.4 (84.6)<br>29.9 (-85; 268.1)<br>n=16	19.5 (116.3)<br>-13.6 (-66.9; 399.4)<br>n=14	12.6 (-44.0; 77.2)<br>p=0.72	14.4 (-59.5; 86.6)<br>p=0.72	10.9 (-61.9; 83.3)<br>p=0.78
**Isovalerate**<br>Randomization to week 4	31.4 (61.3)<br>15.5 (-44.1; 194)<br>n=31	50.6 (69.3)<br>20.2 (-38.7; 194)<br>n=15	13.3 (48.0)<br>-5.4 (-44.1; 125)<br>n=16	79.0 (176.9)<br>37.0 (-74.7; 601.5)<br>n=14	-47.6 (-113.4; 29.0)<br>p=0.23	-28.4 (-126.5; 65.3)<br>p=0.62	-65.7 (-156.2; 21.2)<br>p=0.19
**Isovalerate**<br>Randomization to week 8	17.9 (66.3)<br>-7.3 (-57.9; 190)<br>n=30	33.3 (76.3)<br>19.6 (-57.9; 190)<br>n=14	4.34 (55.13)<br>-15.9 (-57.63; 143.87)<br>n=16	28.9 (92.8)<br>14.2 (-70.4; 295.3)<br>n=13	-11.1 (-59.1; 40.8)<br>p=0.64	4.39 (-61.43; 69.54)<br>p=0.90	-24.6 (-80.9; 30.5)<br>p=0.41
**Isovalerate**<br>Randomization to week 10	29.0 (84.0)<br>10.7 (-81.8; 280.2)<br>n=32	31.3 (90.3)<br>-0.8 (-63.4; 280.2)<br>n=16	26.8 (80.2)<br>17.9 (-81.8; 228.7)<br>n=16	47.9 (184.0)<br>-7.4 (-71.5; 660)<br>n=14	-18.9 (-89.3; 64.2)<br>p=0.61	-16.6 (-115.9; 78.8)<br>p=0.81	-21.2 (-117.8; 70.0)<br>p=0.79
**Succinate**<br>Randomization to week 4	11.8 (103.9)<br>-29.9 (-99; 384.3)<br>n=31	33.7 (130.9)<br>-29.9 (-77.6; 384.3)<br>n=15	-8.76 (68.37)<br>-26.06 (-99.0; 121.1)<br>n=16	-34.6 (77.8)<br>-59.8 (-99.8; 209.1)<br>n=14	46.4 (-11.1; 112.2)<br>p=0.12	68.3 (-12.4; 150.7)<br>p=0.099	25.8 (-28.5; 79.3)<br>p=0.35
**Succinate**<br>Randomization to week 8	260.4 (962.3)<br>15.0 (-99.4; 4988.2)<br>n=30	164.0 (512.6)<br>13.2 (-51.5; 1928.7)<br>n=14	344.8 (1243.5)<br>15.0 (-99.4; 4988.2)<br>n=16	-19.6 (42.1)<br>-11.2 (-99.8; 40.5)<br>n=13	280.1 (8.2; 854.5)<br>p=0.023	183.6 (7.9; 443.2)<br>p=0.026	364.4 (-1.2; 1016.1)<br>p=0.055
**Succinate**<br>Randomization to week 10	26.1 (152.9)<br>-15.2 (-98.3; 720.2)<br>n=32	24.6 (105.3)<br>-15.2 (-75.3; 334.4)<br>n=16	27.6 (193.0)<br>-23.8 (-98.3; 720.2)<br>n=16	-9.19 (99.21)<br>-57.04 (-99.86; 216.73)<br>n=14	35.3 (-37.7; 130.4)<br>p=0.48	33.8 (-40.2; 109.3)<br>p=0.38	36.8 (-60.9; 149.9)<br>p=0.66

For continuous variables Mean (SD) / Median (Min; Max) / n= are presented. For comparison between groups the two-sided Fisher´s Non-Parametric Permutation Test was used for continuous variables. The confidence interval for the mean difference between groups is based on Fishers non-parametric permutation test. High and low dose=10^9-$5x10^9$ CFU/bacteria strain and 10^8-$5x10^8$ CFU twice daily of *D. piger* and *F. praunitzii*, respectively. SCFA=Short Chain Fatty Acid. IP=Investigational Product.

CONSORT 2010 checklist of information to include when reporting a randomised trial*

Section/Topic	Item No	Checklist item	Reported on page No
**Title and abstract**			
	1a	Identification as a randomised trial in the title	SI Page 15
	1b	Structured summary of trial design, methods, results, and conclusions (for specific guidance see CONSORT for abstracts)	SI Page 15-16
**Introduction**			
Background and objectives	2a	Scientific background and explanation of rationale	SI Page 40-41
	2b	Specific objectives or hypotheses	SI Page 42-33
**Methods**			
Trial design	3a	Description of trial design (such as parallel, factorial) including allocation ratio	SI Page 16, 45
	3b	Important changes to methods after trial commencement (such as eligibility criteria), with reasons	NA
Participants	4a	Eligibility criteria for participants	SI Page 20
	4b	Settings and locations where the data were collected	SI Page 17
Interventions	5	The interventions for each group with sufficient details to allow replication, including how and when they were actually administered	SI Page 46
Outcomes	6a	Completely defined pre-specified primary and secondary outcome measures, including how and when they were assessed	SI Page 42-43
	6b	Any changes to trial outcomes after the trial commenced, with reasons	NA
Sample size	7a	How sample size was determined	SI Page 47
	7b	When applicable, explanation of any interim analyses and stopping guidelines	NA
Randomisation:			
Sequence generation	8a	Method used to generate the random allocation sequence	SI Page 16, 46
	8b	Type of randomisation; details of any restriction (such as blocking and block size)	SI Page 16, 46
Allocation concealment mechanism	9	Mechanism used to implement the random allocation sequence (such as sequentially numbered containers), describing any steps taken to conceal the sequence until interventions were assigned	SI Page 16, 46
Implementation	10	Who generated the random allocation sequence, who enrolled participants, and who assigned participants to interventions	SI Page16-17, 46
Blinding	11a	If done, who was blinded after assignment to interventions (for example, participants, care providers, those assessing outcomes) and how	SI Page 46

	11b	If relevant, description of the similarity of interventions	NA
Statistical methods	12a	Statistical methods used to compare groups for primary and secondary outcomes	SI Page 18, 58-61
	12b	Methods for additional analyses, such as subgroup analyses and adjusted analyses	MS Page 28-29, 31-33
**Results**			
Participant flow (a diagram is strongly recommended)	13a	For each group, the numbers of participants who were randomly assigned, received intended treatment, and were analysed for the primary outcome	MS Page 22-23
	13b	For each group, losses and exclusions after randomisation, together with reasons	NA
Recruitment	14a	Dates defining the periods of recruitment and follow-up	SI Page 17
	14b	Why the trial ended or was stopped	NA
Baseline data	15	A table showing baseline demographic and clinical characteristics for each group	S D Table 2
Numbers analysed	16	For each group, number of participants (denominator) included in each analysis and whether the analysis was by original assigned groups	S D Table 2
Outcomes and estimation	17a	For each primary and secondary outcome, results for each group, and the estimated effect size and its precision (such as 95% confidence interval)	S D Table 3-7
	17b	For binary outcomes, presentation of both absolute and relative effect sizes is recommended	S D Table 3-7
Ancillary analyses	18	Results of any other analyses performed, including subgroup analyses and adjusted analyses, distinguishing pre-specified from exploratory	MS Page 8-10
Harms	19	All important harms or unintended effects in each group (for specific guidance see CONSORT for harms)	S D Table 3-4
**Discussion**			
Limitations	20	Trial limitations, addressing sources of potential bias, imprecision, and, if relevant, multiplicity of analyses	MS Page 10
Generalisability	21	Generalisability (external validity, applicability) of the trial findings	MS Page 10-11
Interpretation	22	Interpretation consistent with results, balancing benefits and harms, and considering other relevant evidence	MS Page 10-11
**Other information**			
Registration	23	Registration number and name of trial registry	SI Page 24
Protocol	24	Where the full trial protocol can be accessed, if available	SI Pages 35-48
Funding	25	Sources of funding and other support (such as supply of drugs), role of funders	SI Page 15

*We strongly recommend reading this statement in conjunction with the CONSORT 2010 Explanation and Elaboration for important clarifications on all the items. If relevant, we also recommend reading CONSORT extensions for cluster randomised trials, non-inferiority and equivalence trials, non-pharmacological treatments, herbal interventions, and pragmatic trials. Additional extensions are forthcoming: for those and for up to date references relevant to this checklist, see www.consort-statement.org.

Randomised and placebo controlled trial report

Tolerability and Risk of Adverse Events with a Probiotic Supplement: A Randomised and Placebo Controlled Study in Healthy Individuals

Funding
The study was funded by Vinnova (grant 2017-02964) and Metabogen AB. The investigator had the sole responsibility of planning and conducting the study, as well as all analysis of study results. The sponsor provided the investigational product, but had no role in the study conduct, in the analysis or interpretation of trial results.

Summary
Background and objectives
A probiotic supplement with *D. piger* and *F. praunitzii* has not previously been tested in humans. The aim of this clinical study to investigate the safety and tolerability of a probiotic supplement with these strains was undertaken. The 10-week, double-blind, sex-stratified, randomized, placebo-controlled, single center clinical trial included 50 healthy men and women 20 to 40 years old.

Methods
Participants were randomly allocated to receive capsules once daily with a high ($1x10^9$-$5x10^9$ CFU/bacteria strain; n=18) and low dose ($1x10^8$-$5x10^8$ CFU; n=16) of *D. piger* and *F. praunitzii* or placebo (n=16) for 8 weeks, followed by 2 weeks without supplementation. The primary outcome was tolerability and was defined by discontinuation (yes/no) due to investigational product (IP) during the study. Secondary enpoints were primarlily selected to ensure tolerability (change in gastrointestinal symptoms and frequency of adverse events (AE)) and safety (frequency of AE, serious AEs (SAEs) or change in renal function, liver enzymes, blood cell count, inflammation, total protein) but also efficacy (fasting blood glucose, glycosylated hemoglobin (HbA1c) and feces levels of short chain fatty acids (SCFA); Supplemental Methods). Information regarding compliance and AEs was collected at 6 study visits and by weekly remote monitoring (SFigure 1).

Results
In the intention to treat (ITT) population, 26 women and 24 men, 28.6±5.5 years (mean±standard deviation) were randomised. Baseline characteristics were highly similar between groups, with the exception participants in the placebo group being significantly younger (26.3 ±4.6 vs. 30.5 ± 4.9 years±SD, p=0.02) than those in the low dose group (Extended Data Table 2). During the 8-week treatment period, 4 participants discontinued the study (2 in the placebo group and 2 in the high dose group), none due to adverse events. Both the high and low doses were well tolerated with highly similar AE rates as those observed in the placebo group (Extended Data Table 3). In total, 26 adverse events were distributed on 13 (72.2%) subjects randomized to the high dose, 14 and 7 (43.8%) in the low dose, and 12 and 10 (62.5%) in the placebo group. Change in gastrointestinal symptoms (Extended Data Table 4) were highly similar and reported gastrointestinal AEs were evenly distributed between groups, with 6 subjects with AEs per group (Extended Data Table 3). IP related AEs were evenly distributed among all three groups: 6 (33.3%) in the high dose, 6 (37.5%) in the low dose, and 6 (37.5%) in the placebo group. No serious AEs were observed. There were no group-to-group differences in change (from baseline to 8 weeks) in fasting blood glucose, HbA1c or in any other blood biochemistry end point (STable 5-6). Butyrate, proprionate and

acetate levels increased significantly more in the low dose compared to the placebo group between baseline and week 10 (Extended Data Table 7).

Conclusion and interpretation
Treatment with a probiotic supplement containing *D. piger* and *F. praunitzii* over 8 weeks was found to be safe and well tolerated in healthy young men and women. Due to the small sample size any potential rare adverse events could not be detected. Trial results may not be transferable to other populations. Larger trials in other populations are necessary to determine if this intervention has metabolic benefits and lacks more uncommon side-effects.

Supplement

Extended methods

Study design
Participating volunteers were recruited through advertising in social media (e.g. on Facebook and Instagram) and through posters in public areas (e.g. in Universities, hospitals, and different gyms). In total, 216 men and women were and screened for eligibility and asked to participate. After receiving detailed information about the study, 116 participants declined to participate. Additional participants were ineligible to participate due to inability to speak Swedish (n=4), were diagnosed with any disease (n=8), had recurrent gastrointestinal tract symptoms (n=13), were breast feeding (n=5), were inabel to attend planned study visits (n=5), used prescription medication (n=13), or were ineligible due to other reasons (n=12), leaving 50 participants eligible for inclusion. Participants who met all inclusion criteria, lacked all exclusion criteria (SFigure 2) were invited to participate.

The study was a double-blind, randomized, placebo-controlled, single center trial of 10 weeks in healthy men and women 20 to 40 years old. Eligible participants were randomly allocated to receive capsules once daily with a high ($1x10^9$-$5x10^9$ CFU/bacteria strain; n=18) and low dose ($1x10^8$-$5x10^8$ CFU; n=16) of *D. piger* and *F. praunitzii* or to placebo (n=16) for 8 weeks, followed by a 2 week period without supplementation. Randomisation was performed with varying block size, performed by the sponsor (Metabogen AB, Sweden) using Sealed Envelope Ltd. (2017, https://www.sealedenvelope.com/simple-randomiser/v1/lists). Randomisation was stratified according to sex. Each participant was assigned a study identification number (ID), based on the randomization list, which was kept by the sponsor until study end, completion of the statistical analysis plan, and database lock. Investigational product was labelled with this study ID in order to maintain blinding for participants and study staff.

Information regarding study design, analysis and study objectives was published on Clinicaltrials.gov (NCT03728868) prior to study start.

Primary and secondary end points
The primary outcome was tolerability and was tested using discontinuation (yes/no) during 8 weeks of treatment.
Secondary endpoints including change (between baseline and 8 weeks) in gastrointestinal symptom rating scale (GSRS), fasting blood glucose, glycosylated hemoglobin (HbA1c), renal function (estimated glomerular filtration rate (eGFR) based on serum creatinine), red and white blood cell count, platelet count, serum alanine transaminase (ALT), serum aspartate transaminase (AST), serum alkaline phosphatase (ALP), serum bilirubin, serum C-reactive

protein (CRP), erythrocyte sedimentation rate, serum total protein, feces short chain fatty acid (SCFA) levels (butyrate, acetate, lactate, proprionate, isovalerate, isobutyrate, and succinate), and colonization with F. Prausnitzi in stool, were evaluated between baseline to week 4 and 8 (and after 10 weeks for SCFAs). Analysis of SCFA levels could not be performed in plasma due to methodological difficulties.

Procedures
Six visits to the study clinic (Geriatric Medicine, Sahlgenska University Hospital, Mölndal) were required during the study duration (SFigure 1). Participants received information about the study both in writing and verbally. Eligible participants with no exclusion criteria (SFigure 2) provided a signed informed consent prior to any study procedures and enrollment. Enrollment started on October 10th, 2018 and continued until April 2nd, 2019. The last visit for the most recently included participant was on May 31st, 2019.

Heart rate and blood pressure were measured twice at the screening visit, using a Carescape V100 device (GE Healthcare). Body height, weight, and waist and hip circumferences were measured with a stadiometer as well as a scale and measuring tape. Venous blood was drawn from the cubital vein and used for blood biochemistry analyses. All blood biochemistry was analysed within 4 hours at the Clinical Chemistry laboratory (Sahlgrenska University Hospital Mölndal). All women also completed a pregnancy test (urine human chorionic gonadotropin) which had to be negative for inclusion.

At the randomization visit, feces samples were collected and the Gastrointestinal Symptom Rating Scale (GSRS)[(1)] was completed in order to collect information of any gastrointestinal symptoms the preceeding week. All participants received a diary for recording daily doses taken and to make notes about any potential AEs. During study visits three to five, feces and blood samples as well as data from the GSRS questionnaire form were collected. Two weeks after treatment completion, a last study visit took place to collect data on gastrointestinal symptoms (GSRS) and to collect stool samples.

The first 15 randomised subjects were contacted by telephone daily the first week to inquire about any potential AEs. Thereafter, all participants were contacted by telephone once a week for inquiries about AEs and to collect information about gastrointestinal symptoms (GSRS) the preceeding week.

Intervention
The study product was provided as freeze-dried bacteria packed into capsules designed to disintegrate when reaching the small intestine. Identical capsules and excipient were used for the placebo and IP.

Assessment of gastrointestinal symptoms
Assessment of gastrointestinal symptoms the last week, was performed using the GSRS questionnaire.[(1)] GSRS contains 15 items in total and was analyzed as a total score ranging from 0 to 45. Values 0-9 corresponds to none to minimal gastrointestinal issues, 10-19 minimal gastrointestinal issues, 20-29 moderate gastrointestinal issues, 30-39 moderate to severe gastrointestinal issues, and 40-45 severe gastrointestinal issues.

Blood biochemistry
All blood biochemistry analyses were performed at the Swedac accredited (accreditation number 1240) clinical chemistry laboratory at the Sahlgrenska University Hospital after the

blood draw, without delay. Blood glucose was measured using Glucose HK on a Cobas 6000 instrument (Roche Diagnostics Scandinavia AB). The CV was 3% at concentrations 5 and 15 mmol/L. HbA1c was measured using HPLC (Mono S™, Tricorn™ 50/50 GL (CDP), MonoBeads™ Column (GE Healthcare)). The separated hemoglobin fractions were measured using an UV-detector and absorbance quantified at 417 nm. The CV was 2% at concentrations 42 mmol/mol , 63 mmol/mol and 94 mmol/mol. Erythrocyte sedimentation rate was measured using the Starrsed ST Instrument, Mechatronics (Triolab AB). Erythrocyte count (CV: 3% at 2, 4, and 5 x10^{12}/L) was measured using anti coagulated venous blood with K2-EDTA and measurement of the absorption of light. The instrument used to analyze was the ADVIA 2120i (Siemens Medical Solutions Diagnostics AB). Leukocyte count was measured using anti coagulated venous blood with K2-EDTA and measurement of the absorption of light, using the ADVIA 2120i instrument (Siemens Medical Diagnostics AB), with a CV of 7% at concentrations 3 x 10^9/L to 16 x 10^9/L. Thrombocyte count was measured using anti coagulated venous blood with K2-EDTA and measurement of the absorption of light, with a CV of 9% at 80, 200, and 500 x10^9/L, analyzed an a ADVIA 2120i instrument. Alanine transaminase (ALT) catalyzes the reaction between L-Alanin and 2 oxoglutarat. Further reaction between the produced pyruvate and NADH generates a measure of NADH oxidation, which was directly proportional to the ALT activity, which was measured via the decrease in absorbance. The CV was 6% at 1 µkat/L and 4% at 4 µkat/L and the instrument used was the Cobas 6000. Aspartate transaminase (AST) catalyzes L-Aspartate and 2-oxoglutatrat to oxaloacetate and L-glutamat. The reaction between oxaloacetate and NADH generates a measure of NADH oxidation, which was directly proportional to the AST activity, which was measured via the decrease in absorbance. The CV was 5% at 1 µkat/L and 3% at 3 µkat/L and the instrument used was the Cobas 6000. ALP was analysed using a colorimetric assay using Cobas 6000 with a CV of 4% at 7 µkat/L. Serum total bilirubin was measured using a colometric assay on a Cobas system (Roche Diagnostics Scandinavia AB), with a CV of 5% at concentrations 20 and 130 µmol/l. Serum creatinine was measured using CREP2 on a Cobas 6000 equipment, with a CV of 4% at concentrations 85 and 400 µmol/L. The estimated glomerular filtration rate (eGFR) was calculated using the Lund-Malmö formula based on serum creatinine, age and gender.[(2)] Total protein was measured using on a Cobas 6000 with a CV of 3% at concentrations 50 and 75 g/L.

Statistical analyses
Statistical power was calculated based on anticipated differences in the proportions of study subjects discontinuing due to adverse events. With a discontinuation rate of 0.50 vs. 0.05 due to investigational product in the two treatment groups vs. placebo group (randomized in 2:1, 32 vs. 16 subjects), respectively, with an alpha level of 0.05, using the two-sided Fisher's exact test, a power of 88% was achieved. Comparison of continuous variables between treatment groups (low and high dose) and placebo was performed with Fisher's non-parametric permutation test and the Fisher's exact test (lowest 1-sided p-value multiplied by 2) was used for dichotomous variables. The primary outcome was tolerability and was tested using discontinuation (yes/no) due to investigational product during 8 weeks of treatment. The potential differences in the secondary end point variables, were evaluated by relative change adjusted for placebo and compared with the Fisher's non-parametric permulation test, which also generated the confidence interval for the mean difference. All analyses were performed on complete cases, i.e. no imputations were used. Statistical significance was considered for p-values below 0.05 and all statistics were performed with SAS Software version 9.4 (SAS Institute Inc., Cary, NC, USA).

Extended Results

Participants were randomized to 3 treatment arms, in which 16 (8 (50.0%) men and 8 women) were randomized to the low dose group, 18 (8 (44.4%) men and 10 women) to the high dose group and 16 (8 (50.0%) men and 8 women) to the placebo group (Extended Data Table 2). In the intention to treat (ITT) population, baseline characteristics were similar between groups, with a few exceptions; subjects in the placebo group were significantly younger (26.3 ± 4.6; years) compared to the low (30.5 ± 4.9; p=0.020, years) dose group. Alkaline phosphatase (µkat/L) was significantly lower in the high dose group than in the placebo group (0.92 ± 0.15 vs. 1.15 ± 0.38; p=0.03) and alanine transaminase (µkat/L) was lower in the low dose group than in the placebo group (0.29 ± 0.09 vs. 0.42 ± 0.20; p=0.03). Acetate levels in stool were significantly higher in the placebo than in either treatment group, but no significant differences in other stool SCFAs were observed between groups at baseline (Extended Data Table 2).

Compliance to the treatment, measured as percentage of prescribed doses taken, was generally high with 97.8% in the high dose group, 96.6% in the low dose group, and 97.0% in the placebo group over the course of the 8-week treatment period.

Primary outcome – Tolerability

In the ITT population, two participants in the placeb group discontinued the study within the first week after randomization and 2 participants in the high dose group discontinued the study after the 2nd visit (1 week after randomization). None of the participants discontinued due to an AE. Of those who discontinued, 1 discontinued due to moving outside Sweden and the remaining 3 referred to personal reasons for discontinuing.

Both doses were well tolerated and the incidences of AEs were well distributed between active treatment and placebo. During the study period, a total of 26 AEs distributed on 13 (72.2%) subjects occurred in the participants randomized to the high dose group, 14 and 7 (43.8%) in the low dose group, and 12 and 10 (62.5%) in the placebo group (Extended Data Table 3). The most commonly observed AEs were gastrointestinal events, with 6 subjects within each group, distributed as 33.3% in high dose, 37.5% in low dose, and 37.5% in placebo (Extended Data Table 3). The AEs considered to be related to the IP were also evenly distributed with 6 (33.3%) subjects in the high dose, 6 (37.5%) subjects in the low dose, and 6 (37.5%) subjects in the placebo group (Extended Data Table 3). There were no serious AEs reported in any of the three groups during the study. Highly similar results were observed for the per protocol (PP) population (data not shown).

Secondary outcomes

Both doses of the IP were well tolerated and neither had any negative effects on the the occurrence of gastrointestinal symptoms. There were no differences in placebo adjusted relative change in GSRS at any time point for the both low and high dose groups (Extended Data Table 4). There were no clinically relevant or statistically significant group-to-group differences in change between baseline and 4 or 8 weeks, in any blood biochemistry secondary end points, including eGFR, platelet number, red and white blood cell count, CRP, Hb, erythrocyte sedimentation rate, AST, ALT, ALP, bilirubin, HbA1c or fasting blood glucose (Extended Data Table 5-6).

The relative change between baseline and 10 weeks, in feces butyrate, proprionate and acetate levels in the low dose group were significantly greater than in the placebo group (Extended Data Table 7). Succinate levels increased more in the combined IP groups at 8 weaks, but only the high dose led to significantly higher levels (Extended Data Table 7). Highly similar results were observed for the per protocol (PP) population (data not shown).

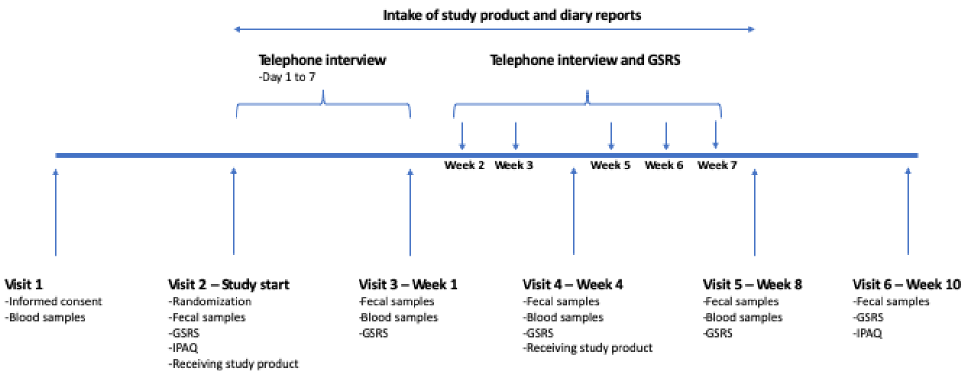

SFigure 1. Study design

Inclusion criteria
- 20-40 years of age.
- Signed informed consent.
- Healthy without any known diseases.
- Willingness and able to participate at planned visits, phone interviews, and to follow study instructions.
- Understand spoken and written Swedish.

Exclusion criteria
- Ongoing treatment with prescribed medication.
- Regular or occasional intake of probiotic supplements.
- Treated with antibiotics within the last three months.
- Pregnancy.
- Have experienced gastrointestinal tract symptoms (during the last month), which could affect study participation, as deemed by study physician.
- Current tobacco use (smoking or snuff)
- Participation in other clinical studies.

Figure 2. Inclusion and exclusion criteria

References

1. Svedlund J, Sjodin I, Dotevall G. GSRS--a clinical rating scale for gastrointestinal symptoms in patients with irritable bowel syndrome and peptic ulcer disease. Dig Dis Sci. Feb 1988;33(2):129-34. Epub 1988/02/01.
2. Bjork J, Back SE, Sterner G, Carlson J, Lindstrom V, Bakoush O, et al. Prediction of relative glomerular filtration rate in adults: new improved equations based on Swedish Caucasians and standardized plasma-creatinine assays. Scand J Clin Lab Invest. 2007;67(7):678-95. Epub 2007/09/14.

Tolerabilitet och biverkningsrisk vid behandling med tarmbakterier hos unga friska frivilliga – en dubbelblind, placebokontrollerad studie

**Datum:**	12 juni 2018
**Sponsor:**	MetaboGen AB
**Projektnummer/identitet:**	META002
**Studiedatum:**	Först deltagaren *Q3 2018*<br>Sista deltagaren fullföljt *Q3 2019*
**Huvudprövare:**	Mattias Lorentzon, professor, överläkare i geriatrik<br>Enheten för geriatrik, Sahlgrenska universitetssjukhuset<br>43180 Mölndal<br>Telefon: 031-3431979/0733-388185<br>E-post: mattias.lorentzon@medic.gu.se
**Deltagande prövare:**	Anna Nilsson, docent, överläkare endokrinologi
**Deltagande forskare:**	Fredrik Bäckhed, Professor<br>Daniel Sundh, Med Dr
**Sponsors representant:**	MetaboGen AB<br>Sara Malcus, CEO<br>Erik Dahlbergsgatan 11A<br>411 26 Göteborg, Sweden<br>Telefon: 031-786 69 36<br>E-post: sara.malcus@metabogen.com

GODKÄNNANDE AV PROTOKOLL

Sponsorns representant

....

Sara Malcus, CEO
MetaboGen AB
Erik Dahlbergsgatan 11A
26 Göteborg

Protokollskrivare

....

Mattias Lorentzon, professor, överläkare i geriatrik
Enheten för geriatrik, Sahlgrenska universitetssjukhuset,
43180 Mölndal

HUVUDPRÖVARENS AVTAL

Jag bekräftar härmed samtycke till att genomföra studien i enlighet med protokollet, god klinisk praxis (GCP), Helsingforsdeklarationen och gällande bestämmelser.

Jag bekräftar att jag ansvarar för det övergripande genomförandet av studien. Jag samtycker till att personligen genomföra eller övervaka den beskrivna studien.

Jag försäkrar att alla medarbetare, kollegor och klinisk personal som hjälper till med genomförandet av studien informeras om sina skyldigheter. Procedurer finns upprättade för att säkerställa att platspersonal får relevant information under hela studien.

[signature]	1/10-2018
Signatur	Datum

Huvudprövarens namn: Mattias Lorentzon

Studieklinik: Sahlgrenska universitetssjukhus
Enheten för geriatrik, Mölndal

Innehållsförteckning

1. Introduktion ... **5**
1.1. Bakgrund ... *5*
1.2. Vetenskaplig frågeställning ... *5*
1.3. Betydelse ... *6*

2. Preliminära resultat ... **6**
2.1. Tolerabilitetsstudie i möss ... *6*
2.2. Virulensfaktorer ... *6*

3. Etiska överväganden ... **7**

4. Studiens utfallsmått ... **7**
4.1. Studiens primära utfallsmått ... *7*
4.2. Studiens sekundära utfallsmått ... *7*

5. Studieplan och tillvägagångssätt ... **8**
5.1. Studiedesign och flödesschema ... *8*
5.2. Rekrytering ... *8*
5.3. Studiebesök 1 ... *8*
5.4. Studiebesök 2 ... *9*
5.5. Studiebesök 3 till 5 ... *9*
5.6. Studiebesök 6 ... *9*
5.7. Telefonintervjuer ... *9*

6. Insamling av biologiska prover och analyser ... **9**
6.1. Blodprover ... *9*
6.2. Avföringsprover ... *10*

7. Inklusions- och exklusionskriterier ... **10**
7.1. Inklusionskriterier ... *10*
7.2. Exklusionskriterier ... *10*

8. Randomisering och blindning ... **11**

9. Studieprodukt ... **11**
9.1. Intag av studieprodukt ... *11*
9.2. Beskrivning av studieprodukt ... *11*

10. Rapportering och hantering av biverkningar ... **11**

11. Avhopp under pågående studien ... **12**

12. Statistiska överväganden ... **12**
12.1. Analyspopulationer ... *12*
12.2. Statistisk styrka ... *12*
12.3. Statistiska analyser ... *12*
12.3.1. Primär analys ... *13*
12.3.2. Sekundära analyser ... *13*

13. Datainsamling och hantering av data ... **13**

14. Referenser ... **13**

1. Introduktion

1.1. Bakgrund

Den humana tarmfloran har under det senaste decenniet blivit erkänd som en miljöfaktor som bidrar till att reglera vår metabolism (1). Studier visar att en förändrad tarmflora är förknippad med ett brett spektrum av sjukdomar som sträcker sig från fetma, typ 2 diabetes och leverförfettning till inflammatorisk tarmsjukdom och autismspektrumtillstånd (2-11). Dessa sjukdomar är associerade med en minskad artmångfald i tarmen och en minskad förekomst av så kallade butyratproducerande bakterier. Den butyratproducerande bakterien *F. prausnitzii* är rikligt förekommande i människans tarm och kan utgöra upp till 5% av tarmfloran hos friska individer. En minskad förekomst av *F. prausnitzii* har kopplats till en obalans av tarmfloran vid metabola syndrom som typ 2-diabetes (5) och leverförfettning (7), och inflammatorisk tarmsjukdom (10-11).

Butyrat är en av flera hälsoassocierade korta fettsyror som butyratproducerande bakterier producerar genom fermentering av kostfiber i tarmen. Detta innebär att en diet med lågt innehåll av kostfiber ger en försämrad kolonisering av butyratproducerande bakterier och därmed en lägre butyratproduktion. Butyrat fungerar som en byggsten för tarmepitelet och har även antiinflammatoriska effekter, vilket kan förklara dess skyddande effekt vid inflammatorisk tarmsjukdom. Flera studier har visat att tarmflorans artdiversitet och förekomsten av *F. prausnitzii* är lägre hos patienter med de inflammatoriska tarmsjukdomarna Crohns sjukdom och ulcerös kolit. Bland annat ses lägre nivåer av antalet butyratproducerande bakterier, inklusive *F. prausnitzii*, och lägre nivåer av korta fettsyror hos personer med ulcerös kolit jämfört med friska personer (10). Liknande resultat har erhållits när patienter med Crohns sjukdom studerats och där personer med en låg förekomst av *F. prausnitzii* löper en högre risk att drabbas av post-operativt återfall av sin sjukdom (11).

Butyrat som produceras i tarmen tas även upp av blodet där den, enligt vår arbetshypotes, kan ha en positiv effekt mot uppkomsten av metabola sjukdomar som typ-2 diabetes och leverförfettning som även de är associerade med en låg förekomst av *F. prausnitzii* (5, 7). Dessa sjukdomar ökar dramatiskt över hela världen och drabbar idag en stor del av befolkningen. Fler än 500 miljoner människor har idag typ-2 diabetes (enligt WHO) och prevalensen av leverförfettning är ca 25 %. Tillsammans utgör dessa sjukdomar stora hot mot världshälsan samt en enorm ekonomisk börda för det globala välfärdssystemet.

Det har blir allt tydligare att bakterier i människans tarmkanal är symbiotiska och beroende av varandras metabolism. *Desulfovibrio piger* är en vanlig sulfatreducerande bakterie som finns närvarande i människans tarm. Hittills har *D. piger* aldrig isolerats utanför matsmältningskanalen, vilket indikerar att bakterien enbart är en naturlig invånare i vårt matsmältningsorgan. Studier utförda av sponsorn (Metabogen AB) visar att butyratproduktionen från *F. prausnitzii* ökar in närvaro av *D. piger* genom att bakterierna samverkar. Det symbiotiska förhållandet mellan *F. prausnitzii* och *D. piger* kan utnyttjas genom att kombinera dessa stammar i ett probiotiskt kosttillskott och därmed upprätthålla butyratproduktionen i tarmen.

1.2. Vetenskaplig frågeställning

Studien avser undersöka hur väl ett intag av ett kosttillskott bestående av kapslar innehållande bakterierna *F. prausnitzii* och *D. piger* tolereras hos unga och friska kvinnor och män. Den primära frågeställningen är hur väl behandling med studieprodukten jämfört med placebo tolereras, vilket definieras som frånvaro av studieavslut på grund av biverkan inom

behandlingstiden. Sekundära utfallsmått inkluderar mag-tarmsymptom (mäts med The Gastrointestinal Symptom Rating Scale-GSRS) och effekter på hematologisk och biokemiska parametrar i blod.

En sekundär frågeställning avser att besvara om *F. prausnitzii* kan kolonisera tarmen i närvaro av *D. piger*. Kolonisering kommer att mätas dels genom kvantitativ PCR där den relativa mängden av *F. prausnitzii* analyseras i avföringsprover och dels genom att mäta plasmanivåerna av korta fettsyror, där butyrat är en av dessa.

1.3. Betydelse

Vår kunskap om tarmfloran och dess betydelse har ökat enormt under det senaste decenniet. Genom att utgå från kunskap om tarmbakteriernas association med positiva och negativa hälsoeffekter, finns goda möjligheter att utveckla nya probiotiska produkter som bygger på att återinföra tarmflorabakterier med positiva hälsoeffekter. Om intag av dessa probiotiska bakteriestammar tolereras väl, så skulle ett kosttillskott där de positiva hälsoeffekterna som är kopplade till *F. prausnitzii* utvecklas och studeras ytterligare.

2. Preliminära resultat

2.1. Tolerabilitetsstudie i möss

En tolerabilitetsstudie har genomförts i 40 stycken Swiss Webster möss under 8 veckor. Djuren var mellan 7 till 8 veckor gamla vid studiestart och tilldelades ett unikt identifieringsnummer. En hög dos av studieprodukten användes och jämfördes med en kontrollprodukt. Tio hanar och 10 honor sondmatades med studieprodukten och 10 hanar och 10 honor sondmatades med kontrollprodukten, tre dagar per vecka under 8 veckor.

Stabiliteten av studieprodukten studerades under studien och baserat på dessa data estimerades förhållandet mellan de två administrerade stammarna till 1:1. Det genomsnittliga antalet cfu (colony forming units) per dos och stam som gavs under studien var 10^{10} cfu. Den genomsnittliga kroppsvikten vid studieavslut var 42 g och baserat på en genomsnittlig kroppsvikt för en kvinna i åldersintervallet 20-39 år på 65 kg (data publicerad av Statistiska centralbyrån) var dosen med avseende på kroppsvikt som gavs i denna musstudie ca 5 000 gånger högre jämfört med den högre dosen som avses att användas i den föreliggande studien.

Under studien undersöktes matintag, kroppsvikt och beteende en gång per vecka under 8 veckor. Mössen var friska och behöll normal vikt, beteende och matkonsumtion under hela studien. Vid studieavslut togs blodprover för hematologiska och biokemiska analyser och en histopatologisk bedömningen genomfördes av insamlade organ (mage, tarm, lever, mjälte, tymus, mesenteriska lymfnoder). Behandlade möss hade ett normalt antal med färre antal vita blodkroppar jämfört med kontrolldjuren, vilket kan vara ett tecken på mindre inflammation, men lika antal röda blodkroppar och trombocyter. Mängden totalprotein i plasma var normal men högre i de behandlade djuren. Dissektion av olika organ visade inte på några synbara förändringar av betydande slag.

2.2. Virulensfaktorer

Bakteriernas antibiotikakänslighet har studerats på ett ackrediterat laboratorium (Utvecklingslaboratoriet för den Europeiska kommittén för testning av antimikrobiell

känslighet, EUCAST, Växjö) och har på så sätt säkerställt att antimikrobiella läkemedel finns att tillgå om oförutsedda biverkningar skulle uppstå.

Bakteriernas genom har sekvenserats och analyserats. Plasmider kunde inte detekteras i samband med denna genomsekvensering vilket indikerar att gener för antibiotikaresistens inte kan överföras från de tillförda stammarna till andra bakterier i tarmen via plasmider. Då genomet analyserades mot en databas för virulensfaktorer, för att identifiera DNA-sekvenser som kan vara associerade med virulens, identifierades en gen för hemolysin och en gen för katalas. Stammarnas hemolyserande förmåga av röda blodkroppar samt dess förmåga att producera katalas har studerats på ett ackrediterat laboratorium (Sahlgrenska bakteriologiska laboratorium). Varken hemolys av röda blodkroppar eller produktion av katalas kunde detekteras.

3. Etiska överväganden

Bakterierna i detta kosttillskott är naturligt förekommande i normalfloran hos friska personer. Av den anledningen anser vi att det inte föreligger några uppenbara säkerhetsrisker för studiedeltagarna. Säkerhetsstudien som genomförts i möss visade på en god tolerabiltet och inga uppenbara säkerhetsrisker kunde detekteras.

Det är idag känt att tarmfloran reglerar vår metabolism och att en förändrad tarmflora kan ses vid metabola syndrom. Tidigare studier har visat att personer med metabola sjukdomar som typ-2 diabetes och leverförfettning, och inflammatorisk tarmsjukdom har en lägre nivå av *F. prausnitzzi* i sin tarm jämfört med friska personer. Genom att upprätthålla en artdivers tarmflora med en hög butyratproduktion är vår hypotes att dessa sjukdomar kan påverkas. Detta skulle kunna ske genom att tillföra *F. prausnitzii* i kombination med *D. piger* som förstärker *F. prausnitziis* koloniseringsförmåga och butyratproduktion.

Sammantaget anser vi att risken för komplikationer för studiedeltagarna är låg samt att antibiotika finns att tillgå om oförutsedda komplikationer skulle uppstå. Vi anser även att de ovan nämnda kunskapsvinster som kan framkomma ur denna studien överväger de eventuellt negativa effekter som studiedeltagarna kan uppleva. Om den föreliggande studien visar att *F. prausnitzii* kan kolonisera tarmen utan komplikationer för studiedeltagarna, så finns en god möjlighet att utveckla ett kosttillskott med förmåga att upprätthålla en gynnsam metabol profil i tarmen med positiva hälsoeffekter.

4. Studiens utfallsmått

4.1. Studiens primära utfallsmått

Studiens primära utfallsmått avser att studera tolerabilitet av studieprodukten hos friska kvinnor och män. Tolerabilitet definieras som fullföljd studie utan avhopp på grund av biverkningar kopplade till studieprodukten.

4.2. Studiens sekundära utfallsmått

Studiens sekundära utfallsmått avser att studera:

- tolerabilitet mätt som mag-tarmsymtom genom skalan gastrointestinal symptom rating scale, GSRS

- förändringar av hematologiska och biokemiska parametrar i blod
- förändringar av blodsockernivåer
- kolonisering av tillförd *F. prausnitzii* i tarmen
- nivåförändring av korta fettsyror i plasma och feces

5. Studieplan och tillvägagångssätt

5.1. Studiedesign och flödesschema

Studien är en randomiserad, placebokontrollerad, dubbelblind 10 veckor lång studie med tre armar där 24 unga friska kvinnor och 24 unga friska män kommer att ingå. Studiedeltagarna som inkluderas i studien kommer sammantaget att besöka studiekliniken vid sex tillfällen (figur 1).

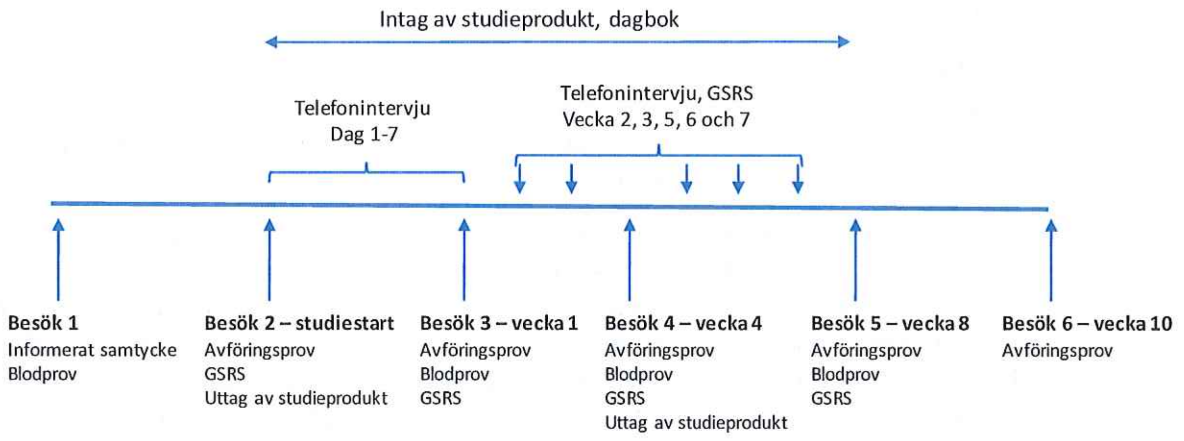

Figur 1. Övergripande flödesschema

5.2. Rekrytering

Genom annonsering i sociala medier, dagstidningar och på allmänna platser på Göteborgs universitet och Sahlgrenska universitetssjukhuset kommer studiedeltagare att rekryteras. Intresserade kvinnor och män ombeds kontakta studiepersonal (via e-post eller telefon) och får ta del av en informationsskrift avsedd för studiedeltagare.

5.3. Studiebesök 1

Under besök 1 får intresserade studiedeltagare träffa studiepersonal som informerar om studien och ställer frågor om inklusions- och exklusionskriterier. De personer som uppfyller kriterierna tillfrågas om deltagande. Deltagandet konfirmeras muntligen och skriftligen genom att det informerat samtycke signeras och kontrasigneras av studiepersonal. Studiedeltagarna får fylla i ett hälsoformulär och svara på frågor gällande hälsa, kost och motionsvanor samt läkemedelsanvändning. Mätning av puls, blodtryck, längd och vikt samt midje- och höftmått sker. Studiedeltagaren ska därefter lämna blodprov och utföra graviditetstest inför en eventuell studiestart. Om analyserade prover (Hb, antal vita, röda blodkroppar och trombocyter, totalprotein, albumin, njur- och leverfunktion, sänkningsreaktion, C-reaktivt protein, fasteglukos, och HbA1c) bedöms vara normala av studieläkare och graviditetstestet är negativt, kan deltagaren fortsätta till randomisering. Studiedeltagaren kommer att uppmanas att undvika graviditet under pågående studie.

5.4. Studiebesök 2

De personer som uppfyller alla inklusionskriterier och inga exklusionskriterier, kallas till studiekliniken inom några dagar för besök 2. Under detta besök lämnas ett avföringsprov och föregående veckas symtom från mag-tarmkanalen besvaras genom GSRS-skalan och utgör deltagarens status innan påbörjat intag av studieprodukt (dag 0). Studiedeltagaren randomiseras till intag av kosttillskott eller placebo. Deltagaren får med sig studieprodukt för fyra veckor samt dagbok för dokumentering av symtom och följsamhet. Intaget av studieprodukt startar nästkommande dag.

5.5. Studiebesök 3 till 5

Under studiebesök 3 till 5 lämnas avföringsprov, blodprov och GSRS-skalan besvaras för vardera föregående vecka. Deltagaren tillfrågas om eventuella biverkningar. Under besök 4 får studiedeltagaren med sig studieprodukt för studiens resterande fyra veckor och gamla förpackningar lämnas in. Gamla förpackningar lämnas även in vid studiebesök 5.

5.6. Studiebesök 6

Två veckor efter avslutat intag av studieprodukt sker ett sista studiebesök där ett avföringsprov lämnas.

5.7. Telefonintervjuer

De första 15 inkluderade studiedeltagarna kommer att kontaktas telefonledes dagligen under den första veckan efter påbörjat intag, för att intervjuas om eventuella biverkningar av studieprodukten (se rubrik 10) och besvara GSRS-skalan. Studiedeltagarna får även telefonnummer till sköterska de kan kontakta vid eventuella biverkningar. Utöver det kommer alla studiedeltagare att telefonintervjuas en gång per vecka och GSRS skalan besvaras med avseende på föregående veckas symtom.

6. Insamling av biologiska prover och analyser

6.1. Blodprover

Blodprover kommer att tas vid besök nummer 1, 3, 4 och 5 (tabell 1). Vid vartdera tillfället kommer maximalt 10 rör med 5 ml blod/rör att tas. Nivåerna av hemoglobin (Hb), antalet leukocyter, erytrocyter och trombocyter, sänkningsreaktion (SR) och långtidsglukos (HbA1c) kommer att studeras i helblod. Fasteglukos mäts i plasma. Lever- och njurfunktion, albumin, samt C-reaktivt protein (CRP) kommer att mätas i serum. Leverproverna inkluderar ALAT, ASAT, ALP och bilirubin. Njurfunktionsproverna inkluderar kreatinin. Blodprover kommer att analyseras vid ett ackrediterat laboratorium vid avdelningen för klinisk kemi, Sahlgrenska universitetssjukhuset eller vid Unilabs. Resterande plasmaprover kommer att förvaras i biobank för senare analys av korta fettsyror, inklusive butyrat, calprotectin, metaboliter, hormoner och cytokiner vid ett externt laboratorium.

Tabell 1. Provtagningsschema och analyser av blod

Analyser	Studiebesök					
	1	2	3	4	5	6
	samtycke	dag 0	v. 1	v. 4	v. 8	v. 10
Helblod: Hematologi, Hb, SR, HbA1c	X		X	X	X	
Serum: CRP, lever- och njurfunktion, albumin	X		X	X	X	
Plasma: Fasteglukos	X		X	X	X	
Plasma till biobank (korta fettsyror, metaboliter, cytokiner, hormoner)	X		X	X	X	

6.2. Avföringsprover

Avföringsprover, inte äldre än 24 timmar, lämnas in i samband med studiebesök 2 till och med besök 6 (tabell 2). Ett prov kan lämnas till studiekliniken upp till två dagar efter inplanerat besök. Studiedeltagarna förses med provtagningsmaterial och provtagningsinstruktion under besök 1 till 5 för insamling av proverna i hemmet. Proverna kommer att förvaras i biobanken för senare analys av kolonisering av *F. prausnitzii* DSM 32379 genom stamspecifik PCR vid externt laboratorium.

Tabell 2. Provtagningsschema och analyser av avföring

Analyser	Studiebesök					
	1	2	3	4	5	6
	samtycke	dag 0	v. 1	v. 4	v. 8	v. 10
Prov till biobank (kolonisering av *F. prausnitzii,* korta fettsyror, calprotectin)		X	X	X	X	X

7. Inklusions- och exklusionskriterier

7.1. Inklusionskriterier

1. 20 till 40 år gammal
2. Signerat samtycke för deltagande
3. Frisk utan några kända sjukdomar
4. Villighet och möjlighet att komma på planerade besök, delta i telefonintervjuer och följa studieinstruktioner
5. Förstå svenska i tal och skrift

7.2. Exklusionskriterier

1. Pågående medicinering med receptbelagt läkemedel
2. Regelbundet eller sporadiskt intag av probiotisk produkt (ej livsmedel med probiotika)
3. Medicinering med antibiotika senaste 3 månaderna
4. Graviditet

5. Upplevda problem från mag-tarmkanalen under den senaste månaden som av studieläkaren bedömas kunna påverka ett studiedeltagande
6. Daglig rökning, snusning
7. Deltagare i annan pågående studie

8. Randomisering och blindning

Studien består av tre armar och randomisering till aktiv studieprodukt eller placebo görs blockvis med varierande blockstorlek och utförs av sponsorn. Studieprodukten ges dubbelblindat, d.v.s. varken studiepersonal eller studiedeltagare har kännedom om huruvida placebo eller aktiv studieprodukt ges. Lika många män och kvinnor kommer att ingå i de tre studiearmarna, dvs randomiseringen kommer att ske stratifierat på kön.

9. Studieprodukt

9.1. Intag av studieprodukt

Studieprodukten tas i form av en kapsel och studeras i två olika doser. Den höga dosen innehåller maximalt 10^9-$5x10^9$ cfu/bakteriestam (colony forming units) och den låga dosen har ett tio gånger lägre innehåll av bakterier. Sexton studiedeltagare randomiseras till placebo under 8 veckor. Sexton deltagare randomiseras till låg dos och sexton deltagare randomiseras till hög dos under 8 veckor. Deltagarna får förpackningar med studieprodukt under besök 2 och vid besök 4 byts använda förpackningar med studieprodukt ut mot nya. Behandlingen pågår under 8 veckor vilket innebär att studiedeltagarna är utan behandling mellan de två sista besöken mellan vecka 8 och 10. Studieprodukten ska tas på fastande mage. Studieproduktens stabilitet kommer att studeras parallellt under hela studien.

9.2. Beskrivning av studieprodukt

F. prausnitzii och *D. piger* isolerades ursprungligen från avföring från en 36 år gammal, frisk, manlig, svensk volontär år 2014. Stammarna som ingår i studieprodukten deponerades under Budapest-fördraget vid DSMZ (Leibniz Institute DSMZ - German Collection of Micro-organisms and Cell Cultures, Braunschweig, Tyskland) och fick deponeringsnummer *F. prausnitzii* DSM 32379 respektive *D. piger* DSM 32187.

För odling och produktutveckling har tillväxtmedierna optimerats för att vara fria från potentiella källor till prioner (bovin spongiform encefalopati). Tillverkningen av de två stammarna utförs på MetaboGens anläggning på Astra Zeneca, Göteborg. Rummet som används för tillverkning har, efter genomgången miljöanalys, bedömts motsvara ett renrum för klass D (EU-GMP).

Den aktiva studieprodukten består av frystorkade bakterier fyllda i enteriska kapslar med en disintegrering i tunntarmen. Produkten kontrolleras före användning av Eurofins (ackrediterat laboratorium för analyser av bl. a. livsmedel) för att utesluta risk för eventuella föroreningar.

Placeboprodukten kommer att innehålla samma excipient som den aktiva studieprodukten.

10. Rapportering och hantering av biverkningar

De första 15 inkluderade studiedeltagarna kommer att kontaktas telefonledes dagligen i en vecka för att intervjuas om eventuella biverkningar av behandlingen. Vid eventuell förekomst

av allvarliga biverkningar bryts koden och säkerhetsanalys genomförs. Om biverkan tros komma från studieprodukten, avslutas studiedeltagandet och antibiotikabehandling med metronidazol kan ges (studieproduktens bakterier är känsliga för metronidazol). Om allvarliga biverkningar saknas under denna tid, kommer efterföljande kontroller (per telefon) göras en gång per vecka under återstoden av studietiden (7 veckor), med undantag för planerade återbesök efter 1, 4, 8 och 10 veckor. Vid varje besök och telefonintervju tillfrågas studiedeltagarna om eventuella biverkningar och fyller i formulär om magbesvär (GSRS) samt lämnar blod och avföringsprov. Försökspersonerna får fylla i studiedagbok och där notera eventuella biverkningar och intag av studieprodukt för följsamhetskontroll. Biverkningar definieras enligt *Common Terminology Criteria for Adverse Events (CTCAE).* Allvarliga biverkningar rapporteras till huvudsponsorn inom 24 timmar.

11. Avhopp under pågående studien

En studiedeltagare har rätt att när som helst avluta studien utan att ange något skäl. Om en deltagare lämnar studien utan att det primära utfallsmåttet uppnåtts inkluderas en ny deltagare för att upprätthålla studiens statistiska styrka. Om en deltagare avslutar studien på grund av biverkningar relaterade till studieprodukten sker ingen ny rekrytering då studiens primära utfallsmått uppnåtts.

12. Statistiska överväganden

En statistisk analysplan med detaljerat beskrivna metoder kommer att skrivas och godkännas av huvudprövaren och sponsorn före studiedatabasen har låsts och analysarbetet har startat.

12.1. Analyspopulationer

Intention to treat (ITT) populationen består av alla patienter som randomiserats till att ta aktiv produkt eller placebo. Per-protokoll-populationen består av alla patienter som tagit >75% av föreskrivna doser och inte konsumerat per oral antibiotika under studietiden eller haft annan signifikant protokollavvikelse. Per-protokoll-populationen kommer att bestämmas blindat på Clean-file mötet som kommer att hållas före studiedatabasen har låsts.

12.2. Statistisk styrka

Eftersom detta är en första studie i människa, planerar vi att undersöka allmän förekomst av biverkningar i behandlade grupper och jämföra med placebo. Om biverkningsrisken (som leder till studieavslut) är 50% i behandlingsgruppen (n=32) och 5% i placebogruppen (n=16), har studien 88% statistisk styrka, förutsatt alpha=0,05 och att tvåsidigt Fisher's exakt test används. Motsvarande styrka för 40% vs. 5% skulle ge en styrka på 70%. Studien kommer att i stora drag ge vägledning om hur pass väl ett intag av studieprodukten tolereras och därmed ge möjlighet att planera studier som med bättre träffsäkerhet kan utvärdera säkerhet och positiv hälsoeffekt.

12.3. Statistiska analyser

12.3.1.Primär analys

Studiens primära analys avser jämförelse mellan *studieprodukten* (båda doserna) och *placebo* vad gäller avhopp på grund av biverkningar kopplade till studieprodukten och kommer att beskrivas med antal och procent för händelsen, samt *event rate* (antal händelser per uppföljd tid i studien) med 95% konfidensintervall baserade på Poisson distribution. Den statistiska jämförelsen kommer att göras på ITT population med två-sidigt Fisher's exakt test. En sensitivitetsanalys på det primära utfallsmåttet kommer att göras med logrank test där uppföljningstiden tas hänsyn till i analysen och avhopp i studien på grund av studiebehandling beskrivs med Kaplan-Meier kurvor och dess 95% konfidensintervall.

12.3.2.Sekundära analyser

Första sekundära analysen i studien kommer jämföra varje dos av *studieprodukten* för sig mot *placebo* med samma metodik som beskriven i 12.3.1 ovan.

Studiens sekundära utfallsmått enligt 4.2 ovan kommer att jämföras både avseende *studieprodukten* totalt vs. *placebo* samt varje dos av *studieprodukten* för sig vs. *placebo* med repeated measures ANOVA om analysantaganden kan visas vara uppfyllda, och i annat fall med Mann-Whitney U-test separat för varje besök. Grafiskt kommer dessa mått att beskrivas med boxplottar.

Den statistiska planen för den relativa kvantifieringen av *F. prausnitzii* DSM 32379 i feces kommer att fastställas då valideringen av analysmetoden har slutförts.

13. Datainsamling och hantering av data

Datainsamling och hantering sker i enlighet med Dataskyddsförordningen (GDPR, General Data Protection Regulation (EU) 2016/679. Loggbok kommer att föras för de olika mätningarna/undersökningarna som möjliggör tydlig spårning vid eventuella frågeställningar vid analys av resultat. Kodning av varje individ kommer att ske vid första mötet, då ett prefix samt nummer kommer att tilldelas personen. Resultat kommer att finnas tillgängligt enbart i dataform, så som grund-, Excel-, samt SPSS-filer. Filerna kommer att vara kodade och tillgängliga på lösenordsbelagda inlåsta datorer som enbart Mattias Lorentzon har tillträde till. Material kommer att sparas i 10 år efter studiens avslutande.

14. Referenser

1. Bäckhed F. et al. The gut microbiota as an environmental factor that regulates fat storage. Proc Natl Acad Sci U S A. 2004 Nov 2;101(44):15718-23

2. Turnbaugh PJ et al. A core gut microbiome in obese and lean twins. Nature. 2009 Jan 22;457(7228):480-4

3. Smith MI et al. Gut microbiomes of Malawian twin pairs discordant for kwashiorkor. Science. 2013 Feb 1;339(6119):548-54

4. Le Chatelier E et al. Richness of human gut microbiome correlates with metabolic markers. Nature. 2013 Aug 29;500(7464):541-6

5. Karlsson FH et al. Gut metagenome in European women with normal, impaired and diabetic glucose control. Nature. 2013 Jun 6;498(7452):99-103.

6. Qin J et al. A metagenome-wide association study of gut microbiota in type 2 diabetes. Nature. 2012 Oct 4;490(7418):55-60

7. Loomba R et al. Gut Microbiome-Based Metagenomic Signature for Non-invasive Detection of Advanced Fibrosis in Human Nonalcoholic Fatty Liver Disease. Cell Metabolism.2017 May 2; 25:1054-62.

8. Ott SJ et al. Reduction in diversity of the colonic mucosa associated bacterial microflora in patients with active inflammatory bowel disease. Gut. 2004 May;53(5):685-93.

9. Hsiao EY et al. Microbiota modulate behavioral and physiological abnormalities associated with neurodevelopmental disorders. Cell. 2013 Dec 19;155(7):1451-63

10. Machiels, K. et al. A decrease of the butyrate-producing species Roseburia hominis and Faecalibacterium prausnitzii defines dysbiosis in patients with ulcerative colitis. Gut 2014 63, 1275–1283

11. Sokol H. et al. Faecalibacterium prausnitzii is an anti-inflammatory commensal bacterium identified by gut microbiota analysis of Crohn disease patients. Proc Natl Acad Sci U S A. 2008 Oct 28;105(43):16731-6.

Tolerability and risk of adverse events in treatment with intestinal bacteria of young, healthy volunteers – a double-blind placebo-controlled study

**Date:**	12 June 2018
**Sponsor:**	MetaboGen AB
**Project number/identity:**	META002
**Study dates:**	First participant: *Q3 2018*<br>Last participant completed: *Q3 2019*
**Principal investigator:**	Mattias Lorentzon, Professor, Senior Geriatrician<br>Geriatrics Unit, Sahlgrenska University Hospital, 431 80 Mölndal [*Sweden*]<br>Telephone: [*+46*] 031-3431979/0733-388185<br>E-mail: mattias.lorentzon@medic.gu.se
**Participating investigator:**	Anna Nilsson, Associate Professor, Senior Endocrinologist
**Participating researchers:**	Fredrik Bäckhed, Professor<br>Daniel Sundh, PhD (Medicine)
**Sponsor's representative:**	MetaboGen AB<br>Sara Malcus, CEO<br>Erik Dahlbergsgatan 11A<br>411 26 Göteborg, Sweden<br>Telephone: [*+46*] 031-786 69 36<br>E-mail: sara.malcus@metabogen.com

AV KAMMARKOLLEGIET AUKTORISERAD TRANSLATOR * NR 686 *

APPROVAL OF PROTOCOL

Sponsor's representative

....

Sara Malcus, CEO
MetaboGen AB
Erik Dahlbergsgatan 11A
[...] [1] 26 Göteborg [*Sweden*]

Author of protocol

....

Mattias Lorentzon, Professor, Senior Geriatrician
Geriatrics Unit, Sahlgrenska University Hospital, 431 80 Mölndal [*Sweden*]

[1] *Part of the postcode is missing in the source text – Translator's Note.*

PRINCIPAL INVESTIGATOR'S AGREEMENT

I hereby confirm that I agree to implement the study in accordance with the protocol, with good clinical practice (GCP), with the Helsiniki Declaration and with the applicable rules.

I confirm that I am responsible for the overall implementation of the study. I agree to personally carry out or monitor the study as described.

I guarantee that all employees, colleagues and clinical staff who will assist in the implementation of the study will be informed of their duties. Procedures are in place to ensure that on-site staff will receive relevant information throughout the study.

______________________________ ______________
Signature Date

Name of the principal investigator: Mattias Lorentzon

Study clinic: Sahlgrenska University Hospital
Geriatrics Unit, Mölndal

Study Protocol: META002 version 0.2
Date: 12 June 2018

Table of contents

1. Introduction **5**
1.1. Background *5*
1.2. Scientific question *5*
1.3. Importance *6*

2. Preliminary findings **6**
2.1. Tolerability study in mice *6*
2.2. Virulence factors *6*

3. Ethical considerations **7**

4. Outcome measures of the study **7**
4.1. Primary outcome measure of the study *7*
4.2. Secondary outcome measures of the study *7*

5. Study plan and approach **8**
5.1. Study design and flow chart *8*
5.2. Recruitment *8*
5.3. Study visit 1 ..... *8*
5.4. Study visit 2 ..... *9*
5.5. Study visits 3 to 5 ..... *9*
5.6. Study visit 6 ..... *9*
5.7. Telephone interviews *9*

6. Collection of biological samples and analyses **9**
6.1. Blood samples *9*
6.2. Faecal samples *10*

7. Inclusion and exclusion criteria **10**
7.1. Inclusion criteria *10*
7.2. Exclusion criteria *10*

8. Randomisation and blinding **11**

9. Study product **11**
9.1. Intake of the study product *11*
9.2. Description of the study product *11*

10. Reporting and management of adverse events **11**

11. Drop-out during the study **12**

12. Statistical considerations **12**
12.1. Populations to be analysed *12*
12.2. Statistical power *12*
12.3. Statistical analyses *12*
12.3.1. Primary analysis *13*
12.3.2. Secondary analyses *13*

13. Data collection and handling **13**

14. References **13**

Study Protocol: META002 version 0.2
Date: 12 June 2018

1. Introduction

1.1. Background

In the past decade, the human intestinal flora has gained recognition as an environmental factor helping to regulate human metabolism (1). Studies have linked changes in the intestinal flora to a wide range of illnesses, ranging from obesity, type 2 diabetes and fatty depositions in the liver to inflammatory bowel disease and autism-spectrum conditions (2–11). These illnesses are associated with a reduced species diversity in the intestine and a reduced presence of butyrate-producing bacteria. *F. prausnitzii* is a butyrate-producing bacterium which is highly prevalent in the human intestine and may account for up to 5% of the intestinal flora in healthy individuals. A reduced presence of *F. prausnitzii* has been linked to an intestinal-flora imbalance in metabolic syndromes such as type 2 diabetes (5) and fatty depositions in the liver (7) as well as inflammatory bowel disease (10–11).

Butyrate is one of several health-associated short fatty acids produced by buturate-producing bacteria through fermentation of roughage in the intestine. This means that a low-fibre diet will cause a deterioration in colonisation by butyrate-producing bacteria and hence a lower butyrate production. Butyrate is an intestinal-epithelium building block and also has an anti-inflammatory effect, which may explain its protective effect in inflammatory bowel disease. Several studies have shown the species diversity of the intestinal flora and the presence of *F. prausnitzii* to be lower in patients with Crohn's disease and ulcerative colitis, which are inflammatory bowel diseases. Among other things, lower levels for the number of butyrate-producing bacteria, including *F. prausnitzii*, and lower levels of short fatty acids are seen in individuals with ulcerative colitis than in healthy individuals (10). Similar findings have been made when studying patients with Crohn's disease, where individuals with a low presence of *F. prausnitzii* are at higher risk of post-operative relapse of their disease (11).

Butyrate produced in the intestine is also absorbed into the blood, where – according to our working hypothesis – it may counteract the development of metabolic diseases such as type 2 diabetes and fatty depositions in the liver, which are also associated with a low presence of *F. prausnitzii* (5, 7). These diseases are increasing dramatically across the world and now affect a large proportion of populations. Today, more than 500 million people have type 2 diabetes (according to the WHO) and the prevalence of fatty depositions in the liver is about 25%. Together, these diseases pose a major threat to global health and place a huge economic burden on the global welfare system.

It has become increasingly clear that the bacteria in the human intestinal tract are symbiotic and dependent on each other's metabolism. *Desulfovibrio piger* is a sulphate-reducing bacterium found in the human intestine. So far, *D. piger* has never been isolated outside the alimentary tract, which indicates that it is a natural inhabitant only of the human alimentary organ. Studies carried out by the sponsor (MetaboGen AB) show that buturate production by *F. prausnitzii* increases in the presence of *D. piger* through interaction between the bacteria. The symbiotic relationship between *F. prausnitzii* and *D. piger* can be exploited by combining these strains in a probiotic dietary supplement with a view to maintaining buturate production in the intestine.

1.2. Scientific question

The study aims to investigate how well the intake of a dietary supplement consisting of capsules containing the *F. prausnitzii* and *D. piger* bacteria is tolerated in young, healthy women and men. The primary question is how well treatment with the study product is tolerated compared with placebo, defined as the absence, within the treatment period, of study termination owing to adverse events. Secondary outcome measures include gastrointestinal symptoms (measured using the Gastrointestinal

Symptom Rating Scale, or GSRS) as well as effects on haematological and biochemical blood parameters.

A secondary question is whether *F. prausnitzii* can colonise the intestine in the presence of *D. piger*. Colonisation will be measured (i) using quantitative PCR, where the relative amount of *F. prausnitzii* will be analysed in faecal samples, and (ii) by measuring levels of short fatty acids, including buturate, in plasma.

1.3. Importance

Our knowledge about the intestinal flora and its importance has grown enormously in the past decade. Taking existing knowledge about the association of intestinal bacteria with positive and negative health effects as a starting point, there are good opportunities to develop new probiotic products based on the reintroduction of intestinal-flora bacteria with positive health effects. If the intake of these probiotic bacterial strains is well tolerated, it could be possible to develop and further investigate a dietary supplement taking advantage of the positive health effects linked to *F. prausnitzii.*[2]

2. Preliminary findings

2.1. Tolerability study in mice

A tolerability study was conducted in 40 Swiss Webster mice over 8 weeks. The animals were 7–8 weeks old when the study started and were assigned unique identification numbers. A high dose of the study product was used and compared with a control product. Using a probe, the study product was fed to 10 males and 10 females while the control product was fed to 10 males and 10 females, 3 days a week for 8 weeks.

In the course of the study, the stability of the study product was investigated; based on the data obtained, the relationship between the two strains administered was estimated to be 1:1. The average number of colony-forming units (cfu) per dose and strain administered during the study was 10^{10}. Average body weight at the end of the study was 42 g; based on the average body weight of a woman aged 20–39 years, i.e. 65 kg (data published by Statistics Sweden), the dose relative to body weight administered in this study on mice was about 5,000 times higher than the higher of the doses intended to be used in the present study.

During the study, food intake, body weight and behaviour were examined once a week for 8 weeks. The mice were in good health and retained normal weight, behaviour and food consumption throughout the study. At the end of the study, blood samples were drawn for haematological and biochemical analyses, and a histopathological assessment was carried out of organs gathered (stomach, intestine, liver, spleen, thymus and mesenteric lymphatic nodes). The treated mice had a normal number of leucocytes but still fewer than the control animals, which could indicate less inflammation, and equal numbers of erythrocytes and platelets. The total amount of protein in plasma was normal but higher in the treated animals. Dissection of various organs did not show any major visible changes.

2.2. Virulence factors

The bacteria's sensitivity to antibiotics was tested at an accredited laboratory (Developmental Laboratory of the European Committee on Antimicrobial Susceptibility Testing, EUCAST, Växjö,

[2] *Literally, the source text reads, "if the intake of these probiotic bacterial strains is well tolerated, a dietary supplement where the positive health effects linked to F. prausnitzii would be developed and studied further", which is not syntactically coherent. This is my interpretation – Translator's Note.*

Sweden) to ensure that antimicrobial drugs will be available in the event of unforeseen adverse events.

The genomes of the bacteria have been sequenced and analysed. No plasmids were detected in genome sequencing, indicating that genes for resistance to antibiotics cannot be transferred via plasmids from the strains added to other intestinal bacteria. Analysis of the genome against a database of virulence factors, to identify DNA sequences that could be associated with virulence, identified a gene for haemolysin and a gene for catalase. The strains' ability to haemolyse erythrocytes and their ability to produce catalase have been studied at an accredited laboratory (the Sahlgrenska Bacteriological Laboratory). Neither erythrocyte haemolysis nor catalase production could be detected.

3. Ethical considerations

The bacteria included in the present dietary supplement occur naturally in the normal flora of healthy individuals. For this reason, we do not consider there to be any obvious safety risks for study participants. The safety study conducted on mice showed good tolerability and no obvious safety risks were detected.

We now know that the intestinal flora regulates human metabolism and that a changed intestinal flora can be seen in metabolic syndromes. Earlier studies have shown than individuals with metabolic conditions such as type 2 diabetes and fatty depositions in the liver or inflammatory bowel disease have a lower level of *F. prausnitzii* in their intestine than healthy individuals. Our hypothesis is that these conditions can be affected by the maintenance of a species-diverse intestinal flora with a high production of butyrates. This could be obtained by adding *F. prausnitzii* in combination with *D. piger*, which reinforces the colonisation ability and butyrate production of *F. prausnitzii.*

Taken together, we consider that the risk of complications for study participants is low and that antibiotics are available in the event of unforeseen adverse events. We also consider that the above-mentioned knowledge gains that the present study may yield outweigh any potential negative effects that study participants may experience. If the present study shows that *F. prausnitzii* can colonise the intestine with no complications for study participants, there are good opportunities to develop a dietary supplement capable of maintaining a favourable metabolic profile in the intestine with positive health effects.

4. Outcome measures of the study

4.1. Primary outcome measure of the study

The primary outcome measure of the study relates to the tolerability of the study product in healthy women and men. Tolerability is defined as completing the study without termination due to adverse events associated with the study product.

4.2. Secondary outcome measures of the study

The secondary outcome measures of the study relate to:

- tolerability measured as gastrointestinal symptoms using the Gastrointestinal Symptom Rating Scale (GSRS)

- changes in haematological and biochemical parameters in blood
- changes in blood sugar levels
- colonisation by added *F. prausnitzii* in the intestine
- changes in the level of short fatty acids in plasma and faeces

5. Study plan and approach

5.1. Study design and flow chart

The study is a randomised, placebo-controlled, double-blind, three-armed, 10-week study in which 24 young, healthy women and 24 young, healthy men will participate. Study participants included in the study will visit the study clinic on a total of 6 occasions (Figure 1).

Intake of study product, journal

Telephone interview days 1–7

Telephone interview, GSRS weeks 2, 3, 5, 6 and 7

**Visit 1**	**Visit 2 – study start**	**Visit 3 – week 1**	**Visit 4 – week 4**	**Visit 5 – week 8**	**Visit 6 – week 10**
Informed consent	Faecal sample	Faecal sample	Faecal sample	Faecal sample	Faecal sample
Blood sample	GSRS	Blood sample	Blood sample	Blood sample	
	Dispensation of study product	GSRS	GSRS	GSRS	
			Dispensation of study product		

Figure 1. Overall flow chart [3]

5.2. Recruitment

Study participants will be recruited through advertising in social media and daily newspapers as well as in public locations at the University of Gothenburg and the Sahlgrenska University Hospital. Women and men who are interested in participating will be asked to contact study staff (via e-mail or telephone) and will be given an informational text intended for study participants.

5.3. Study visit 1

During study visit 1, those interested in participating in the study will meet study staff who will provide information about the study and ask questions pertaining to inclusion and exclusion criteria. Those individuals who meet the criteria will be asked to participate. Participation will be confirmed orally and in writing through the signing, and countersigning by study staff, of an informed-consent form. Study participants will fill in a health questionnaire and answer questions relating to their health, diet, exercise habits and use of medical drugs. Their heart rate, blood pressure, height and weight will be measured and their waist and hip measures taken. Then study participants will leave blood samples and undergo a pregnancy test prior to possibly starting the study. If the samples analysed (Hb, numbers of leucocytes, erythrocytes and platelets, total protein, albumin, kidney and liver function, erythrocyte-sedimentation rate, C-reactive protein, fasting glucose and HbA1c) are deemed to be normal by the study doctor and the pregnancy test is negative, a participant may go on to randomisation. Study participants will be told to avoid pregnancy during the course of the study.

[3] *For a version of the flow chart with more professional layout, including arrows, please refer to the attached source text – Translator's Note*

5.4. Study visit 2

Those individuals who meet all inclusion criteria but none of the exclusion criteria will be given an appointment at the study clinic within a few days for visit 2. During that visit, they will give a faecal sample and answer questions about gastrointestinal symptoms in the previous week using the GSRS scale; this will constitute each participant's baseline status before beginning the intake of the study product (day 0). Study participants will be randomised to either a dietary supplement or placebo. They will receive enough study product for four weeks as well as a journal in which they will document any symptoms and their compliance. The intake of the study product will begin on the next day.

5.5. Study visits 3 to 5

During study visits 3 to 5, participants will give a faecal sample and a blood sample, and they will answer the GSRS scale for the respective previous week. At visit 4, participants will be given enough study product for the remaining four weeks of the study and return used containers. Used containers will also be returned at study visit 5.

5.6. Study visit 6

Two weeks after the intake of the study product has ended, a final study visit will take place during which a faecal sample will be given.

5.7. Telephone interviews

The first 15 study participants included will be contacted over the telephone each day during the first week after starting their intake, to be interviewed about any adverse effects of the study product (see heading 10) and answer the GSRS scale. The study participants will also be given the telephone number of a nurse whom they may contact in case of adverse events. In addition to this, all study participants will be interviewed over the telephone once a week and answer the GSRS scale with regard to symptoms in the previous week.

6. Collection of biological samples and analyses

6.1. Blood samples

Blood samples will be drawn at visits 1, 3, 4 and 5 (Table 1). On each occasion, a maximum of 10 tubes with 5 ml of blood per tube will be drawn. Levels of haemoglobin (Hb), numbers of leucocytes, erythrocytes and platelets, the erythrocyte-sedimentation rate (ESR) and long-term glucose (HbA1c) will be studied in whole blood. Fasting glucose will be measured in plasma. Liver and kidney function, albumin and C-reactive protein (CRP) will be measured in serum. Liver tests include ALAT, ASAT, ALP and bilirubin. Kidney-function tests include creatinine and cystatin C. The blood samples will be analysed at an accredited laboratory at the Department of Clinical Chemistry at the Sahlgrenska University Hospital, or at Unilabs. The remaining plasma samples will be kept in a biobank for later analysis of short fatty acids, including butyrate, calprotectin, metabolites, hormones and cytokines at an external laboratory.

Table 1. Blood-sampling schedule and blood analyses

Analyses	Study visits					
	1	2	3	4	5	6
	consent	day 0	week 1	week 4	week 8	week 10
Whole blood: haematology, Hb, SR, HbA1c	X		X	X	X	
Serum: CRP, liver and kidney function, albumin	X		X	X	X	
Plasma: fasting glucose	X		X	X	X	
Plasma to biobank (short fatty acids, metabolites, cytokines, hormones)	X		X	X	X	

6.2. Faecal samples

Faecal samples, not older than 24 hours, will be submitted in conjunction with study visits 2 to 6 inclusive (Table 2). A sample may be handed in to the study clinic up to two days after a planned visit. At visits 1 to 5, study participants will be given sampling equipment and instructions to collect samples at home. The samples will be kept in the biobank for later analysis of colonisation by *F. prausnitzii* DSM 32379 by means of strain-specific PCR at an external laboratory.

Table 2. Faecal-sampling schedule and faecal analyses

Analyses	Study visits					
	1	2	3	4	5	6
	consent	day 0	week 1	week 4	week 8	week 10
Samples for biobank (colonisation by *F. prausnitzii*, short fatty acids, calprotectin)		X	X	X	X	X

7. Inclusion and exclusion criteria

7.1. Inclusion criteria

1. Age 20 to 40 years
2. Signed participation consent
3. Healthy, with no known illnesses
4. Willingness and ability to come for planned visits, participate in telephone interviews and comply with study instructions
5. Understanding spoken and written Swedish

7.2. Exclusion criteria

1. Ongoing medication with a prescription drug
2. Regular or occasional intake of a probiotic product (not foodstuffs with probiotics)
3. Medication with antibiotics in the past 3 months
4. Pregnancy

5. Gastrointestinal problems experienced in the past month that the study doctor considers might affect study participation
6. Daily smoking or use of *snus* (moist tobacco)
7. Participation in another ongoing study

8. Randomisation and blinding

The study consists of three arms, and randomisation to the active study product or placebo will be performed by the sponsor on the basis of blocks of varying size. The study product will be administered in a double-blind fashion, meaning that neither study staff nor study participants will know whether placebo or the active study product is given. Equal numbers of men and women will be included in the three study arms, meaning that randomisation will be stratified by sex.

9. Study product

9.1. Intake of the study product

The study product will be taken in the form of a capsule and will be studied at two different dosages. The higher dose will contain a maximum of 10^9–$5x10^9$ colony-forming units (cfu) per bacterial strain while the lower dose will have a 10 times lower bacterial content. Sixteen study participants will be randomised to placebo for 8 weeks. Sixteen participants will be randomised to the lower dose and sixteen participants will be randomised to the higher dose for 8 weeks. Participants will be given study-product containers at visit 2, and at visit 4 used study-product containers will be replaced with new ones. Treatment will last for 8 weeks, meaning that study participants will be non-treated between the last two visits, between weeks 8 and 10. The study product is to be taken on an empty stomach. The stability of the study product will be studied in parallel throughout the study.

9.2. Description of the study product

F. prausnitzii and *D. piger* were first isolated from faeces from a 36-year-old healthy male Swedish volunteer in 2014. The strains included in the study product were deposited under the Budapest Treaty with DSMZ (Leibniz Institute DSMZ – German Collection of Micro-organisms and Cell Cultures, Brunswick, Germany) and were assigned deposit numbers *F. prausnitzii* DSM 32379 and *D. piger* DSM 32187, respectively.

For cultivation and product development, the growth media have been optimised to be free of potential sources of prions (bovine spongiform encephalopathy). Manufacturing of the two strains is carried out at MetaboGen's facility at Astra Zeneca, Gothenburg[, *Sweden*]. Following environmental analysis, the room used for manufacturing has been found to correspond to a grade D cleanroom (EU-GMP).

The active study product consists of freeze-dried bacteria filled into enteric capsules for disintegration in the small intestine. Before use, the product is checked by Eurofins (an accredited laboratory for the analysis of, *inter alia*, foodstuffs) to exclude the risk of potential contamination.

The placebo product will contain the same excipient as the active study product.

10.Reporting and management of adverse events

The first 15 study participants included will be contacted over the telephone every day for a week to be interviewed about any adverse effects of the treatment. In the event of serious adverse events, the

code will be broken and a safety analysis will be conducted. If it is believed that the adverse event is due to the study product, study participation will be terminated and antibiotics treatment with metronidazole may be given (the bacteria in the study product are sensitive to metronidazole). If no serious adverse events are reported during this period, subsequent checks (by telephone) will be carried out once a week during the remainder of the study period (7 weeks), with exceptions for the planned visits after 1, 4, 8 and 10 weeks. During each visit and telephone interview, study participants will be asked about any adverse events and will fill in a stomach-troubles form (GSRS) and leave blood and faecal samples. The study participants will fill in a study journal, making notes there about any adverse events and about their intake of the study product, for compliance checks. Adverse events are defined in accordance with the Common Terminology Criteria for Adverse Events (CTCAE). Serious adverse events will be reported to the main sponsor within 24 hours.

11.Drop-out during the study

Study participants may terminate the study at any time without giving a reason. If a participants leaves the study without the primary outcome measure having been obtained, a new participant will be included to maintain the statistical power of the study. If a participant terminates the study because of adverse events associated with the study product, no new recruitment will take place as the primary outcome measure of the study will have been obtained.

12.Statistical considerations

A statistical-analysis plan with detailed descriptions of methods will be developed and approved by the principal investigator and the sponsor before the study database is locked and analysis begins.

12.1. Populations to be analysed

The intention-to-treat (ITT) population consists of all patients randomised to take the active product or placebo. The per-protocol population consists of all patients who have taken >75% of the prescribed doses and who have not consumed any oral antibiotics during the study period or deviated from the protocol in some other significant manner. The per-protocol population will be determined on a blinded basis at the clean-file meeting to be held before the study database is locked.

12.2. Statistical power

Since this is a first study on humans, we plan to investigate the general presence of adverse events in the treated groups and compare with placebo. If the risk of adverse events (causing study termination) is 50% in the treatment group (n=32) and 5% in the placebo group (n=16), the study will have a statistical power of 88%, provided that alpha=0.05 and the two-sided Fisher's exact test is used. The corresponding power for 40% vs. 5% would be 70%. The study will provide general guidance on how well the intake of the study product is tolerated, thus enabling the planning of studies that will be able to evaluate safety and positive health effects with better accuracy.

12.3. Statistical analyses

12.3.1. Primary analysis

The primary analysis of the study pertains to comparison between the *study product* (both dosages) and *placebo* with regard to termination due to adverse events associated with the study product; this will be described in terms of numbers and percentages for this event as well as the event rate (number of events per monitoring time in the study) with a 95% confidence interval based on the Poisson distribution. The statistical comparison will be made with regard to the ITT population using the two-sided Fisher's exact test. Sensitivity analysis of the primary outcome measure will be carried out using the logrank test, where monitoring time will be considered in the analysis and where study termination due to the study treatment will be described using the Kaplan–Meier curve and its 95% confidence interval.

12.3.2. Secondary analyses

The first secondary analysis within the study will compare each dosage of the *study product* separately with *placebo* using the same methods described in 12.3.1 above.

The secondary outcome measures of the study according to 4.2 above will be compared both with respect to the *study product* overall vs. *placebo* and with respect to each dosage of the *study product* separately vs. *placebo*, using repeated-measures ANOVA if the analytical assumptions can be shown to hold and otherwise using the Mann–Whitney U-test separately for each visit. Graphically, these measures will be described using box plots.

The statistical plan for the relative quantification of *F. prausnitzii* DSM 32379 in faeces will be determined when the validation of the analytical method has been completed.

13.Data collection and handling

Data collection and handling will be carried out in accordance with the European Union's General Data Protection Regulation (EU) 2016/679 (GDPR). Logs will be kept for the various measurements/ examinations to enable clear tracing in the event of interrogations during the analysis of results. Coding of each individual will be performed at the first meeting, when a prefix and a number will be assigned to each person. The results will be available only in the form of data such as basic data, Excel files and SPSS files. The files will be coded and available on password-protected and physically locked-up computers to which only Mattias Lorentzon has access. The material will be kept for 10 years after the end of the study.

14.References

1. Bäckhed F. et al. The gut microbiota as an environmental factor that regulates fat storage. Proc Natl Acad Sci U S A. 2004 Nov 2;101(44):15718–23

2. Turnbaugh PJ et al. A core gut microbiome in obese and lean twins. Nature. 2009 Jan 22;457(7228):480–4

3. Smith MI et al. Gut microbiomes of Malawian twin pairs discordant for kwashiorkor. Science. 2013 Feb 1;339(6119):548–54

4. Le Chatelier E et al. Richness of human gut microbiome correlates with metabolic markers. Nature. 2013 Aug 29;500(7464):541–6

5. Karlsson FH et al. Gut metagenome in European women with normal, impaired and diabetic glucose control. Nature. 2013 Jun 6;498(7452):99–103.

Statistical Analysis Plan

Final

META002

Tolerability and Risk of Adverse Events With a Probiotic Supplement: A Randomised and Placebo Controlled Study in Healthy Individuals

2019-11-02

Author

Name/Title:
Aldina Pivodic / Statistician, Statistiska konsultgruppen
Signature: Aldina Pivodic   Date: 2019-11-03

Approvals

Name/Title
Mattias Lorentzon / Principal Investigator, Sahlgrenska University Hospital
Signature: [signature]   Date: 2019-11-02
Name/Title
Sara Malcus / Metabogen AB
Signature: [signature]   Date: 2019-11-02

Revisions

Version	Description of Changes	Date

Table of Contents

1 Study Details 5
1.1 Study Objectives 5
1.2 Study Design 5
1.3 Treatment Groups 5
1.4 Sample Size 6
1.5 Inclusion/Exclusion Criteria 6
1.5.1 Inclusion Criteria 6
1.5.2 Exclusion Criteria 6
2 Study Populations 7
2.1 Definition of Study Populations 7
2.1.1 Intention-to-Treat Population 7
2.1.2 Per-Protocol Population 7
2.1.3 Safety Population 7
3 Study Variables 7
3.1 Baseline Variables 7
3.1.1 Demographics and Baseline Characteristics 7
3.2 Study Variables 8
3.2.1 Primary Tolerability Variable 8
3.2.2 Secondary Variables 8
3.3 Safety Variables 9
3.3.1 Exposure and Compliance of Investigational Product 9
3.3.2 Adverse Events 9
4 Statistical Methodology 10
4.1 General Methodology 10
4.2 Subject Disposition and Data Sets Analyzed 10
4.3 Protocol Violations/Deviations 10
4.4 Demographics and Baseline Characteristics 11
4.5 Primary and Secondary Analyses 11
4.5.1 Primary Tolerability Analysis 11
4.5.2 Secondary Analyses 11
4.5.3 Exploratory Analyses 11
4.5.4 Subgroup Analyses 11
4.6 Safety Analyses 12
4.6.1 Exposure and Compliance of Investigational Product 12
4.6.2 Adverse Events 12

5 Interim Analyses 12
6 Changes of Analysis from Protocol 12
7 Listing of Table, Figures and Listings 13
7.1 Listing of Tables 13
7.2 Listing of Figures 13
7.3 Listing of Listings 13

LIST OF ABBREVIATIONS

Abbreviation	Definition
AE	Adverse events
ALAT	Alanine transaminase
ALP	Alkaline phosphatase
ASAT	Aspartate transaminase
ATC	Anatomical therapeutic classification
BMI	Body mass index
CI	Confidence interval
CRP	C-reactive protein
CTCAE	Common terminology criteria for adverse events
GSRS	Gastrointestinal symptom rating scale
IP	Investigational product
IRR	Incidence rate ratio
ITT	Intention to treat
PP	Per protocol
PT	Preferred term
SAE	Serious adverse events
SD	Standard deviation
SOC	System organ class

1 STUDY DETAILS

1.1 Study Objectives

The primary objective in this study is to evaluate the tolerability of *F. prausnitzii* and *D. piger* in healthy subjects measured by study discontinuation due to investigational product (IP).

The secondary objectives in this study are:

- To evaluate the tolerability of *F. prausnitzii* and *D. piger* measured by gastrointestinal symptom rating scale (GSRS)
- To evaluate the impact on hematologic and biochemical parameters in blood
- To evaluate the impact on glycaemic parameters
- To evaluate the impact on short fatty acids in feces
- To evaluate the colonization of intestine with *F. prausnitzii*

1.2 Study Design

This is a double-blind, placebo-controlled, randomized study, designed to investigate if dietary supplementation with *F. prausnitzii* (combined with *D. piger*) once a day, in two different doses, for 8 consecutive weeks is tolerated compared to placebo and if it can affect the metabolism in a positive way.

Study Flowchart:

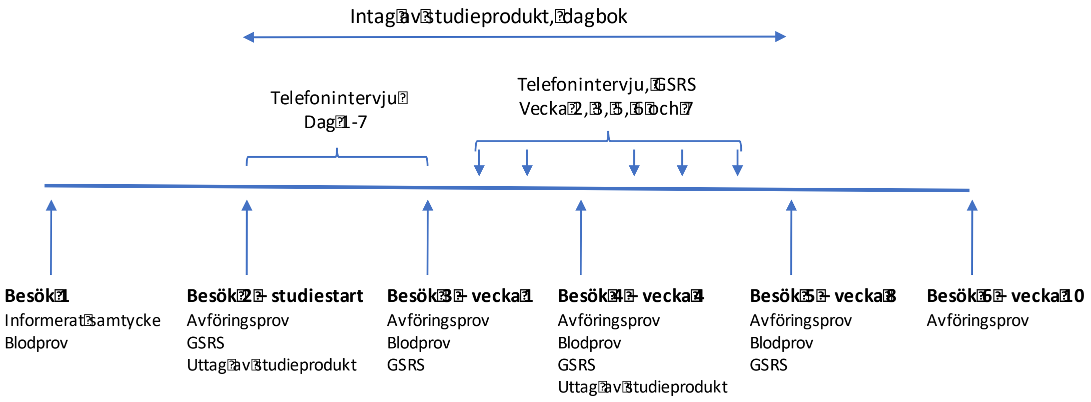

Study visit 1 (informed consent), visit 2 (study start, randomization, study treatment starts on following day), visit 3 (week 1), visit 4 (week 4), visit 5 (week 8, study treatment ends) and visit 6 (week 10, follow-up visit) are planned to be performed.

1.3 Treatment Groups

Following treatment groups are studied:

- Placebo Comparator: Placebo

One capsule containing placebo (identical to the capsule with active product (*F. prausnitzii* and *D. piger*) in taste and appearance but without the active component) taken orally once a day (morning), one hour before breakfast on empty stomach, for 8 consecutive weeks.

- Active Comparator: High dose *F. prausnitzii* and *D. piger*

  One capsule (containing *F. prausnitzii* and *D. piger* at a dose of 10E9-5x10E9 colony forming units per bacterial strain) taken orally once a day (morning), one hour before breakfast on empty stomach, for 8 consecutive weeks.

- Active Comparator: Low dose *F. prausnitzii* and *D. piger*

  One capsule (containing *F. prausnitzii* and *D. piger* at a dose of 10E8-5x10E8 colony forming units per bacterial strain) taken orally once a day (morning), one hour before breakfast on empty stomach, for 8 consecutive weeks.

1.4 Sample Size

Since this is a first-in-man study, tolerability and general occurrence of side effects will be studied in the treated groups and compared to placebo. Assuming a discontinuation rate of 0.50 vs 0.05 due to IP in the two treatment groups vs placebo group (randomized in 2:1, 32 vs 16 subjects), respectively, alpha 0.05, and using two-sided Fisher's exact test, a power of 88% could be achieved. Corresponding calculation of a discontinuation rate of 0.40 vs 0.05 would result in 70% power.

1.5 Inclusion/Exclusion Criteria

1.5.1 Inclusion Criteria

1. 20 to 40 years old
2. Signed consent for participation
3. Healthy individuals without any known diseases
4. Willingness and ability to attend to planned visits, participate in telephone interviews and follow study instructions
5. Understanding the Swedish language in spoken and written terms

1.5.2 Exclusion Criteria

1. Ongoing treatment with prescription drugs
2. Regular or sporadic intake of probiotic products (foods with probiotics are allowed)
3. Treated with antibiotics during the last 3 months
4. Pregnancy
5. Have experienced gastrointestinal tract symptoms (during the last month), which could affect study participation, as deemed by study physician.
6. Current tobacco use (smoking or snuff)
7. Participation in another clinical study

2 STUDY POPULATIONS

2.1 Definition of Study Populations

2.1.1 Intention-to-Treat Population

All randomized subjects will be included in the Intention-to-Treat (ITT) population.

2.1.2 Per-Protocol Population

All randomized subjects that have complied to >75% of the received doses, and have not used oral antibiotics during the study period, and did not have any other major protocol violations will be included in the Per Protocol (PP) population. The final decisions regarding the PP population will be taken, in blinded manner, at the Clean File meeting before the database lock.

2.1.3 Safety Population

All enrolled subjects who received at least one dose of the randomised IP will be included in the safety population.

3 STUDY VARIABLES

3.1 Baseline Variables

3.1.1 Demographics and Baseline Characteristics

Following baseline variables will be described:

- Age (years)
- Sex (female/male)
- Body Mass Index (BMI; kg/m2)
- Waist hip ratio
- Systolic blood pressure (mean from 2 measurements, mm/hg)
- Diastolic blood pressure (mean from 2 measurements, mm/hg)
- Renal function (based on serum creatinine, calculated eGFR; ml/min)
- Blood haemoglobin (Hb; g/l)
- Blood total protein (g/l)
- Blood fasting blood glucose (mmol/L)
- Blood HBA1C (mmol/mol)
- GSRS (score 0-45)
- Blood erythrocyte sedimentation rate (mm/hour)

- Blood C-reactive protein (CRP) (mg/L)
- Blood red blood cell count (number, 10^9/L)
- Blood white blood cell count (number, 10^9/L)
- Blood platelet count (number, 10^9/L)
- Blood alanine transaminase (ALAT) (µkat/L)
- Blood aspartate transaminase (ASAT) (µkat/L)
- Blood alkaline phosphatase (ALP) (µkat/L)
- Blood bilirubin (µmol/L)
- Colonization with *F. prausnitzii* in stool
- Stool short chain fatty acids (nmol/mg dry weight)
    - Butyrate
    - Propionate
    - Acetate
    - Lactate
    - Isobutyrate
    - Isovalerate
    - Succinate

3.2 Study Variables

3.2.1 Primary Tolerability Variable

Primary variable is study discontinuation due to IP during 8 weeks of treatment, and will be measured as No/Yes at the end of subjects' follow-up. Early discontinuations due to other reasons will be handled as No discontinuation due to IP. No imputations will be made.

3.2.2 Secondary Variables

Following secondary variables will be analysed:

- Change in GSRS from baseline to week 4 and 8. GSRS includes 15 items and will be analysed as total score. The total score ranges from 0 to 45 and is derived by summing up the scores for the included items. Values 0-9 means *None to minimal gastrointestinal issues*, 10-19 *Minimal gastrointestinal issues*, 20-29 *Moderate gastrointestinal issues*, 30-39 *Moderate to severe gastrointestinal issues* and 40-45 *Severe gastrointestinal issues*.
- Change in hematologic and biochemical parameters in blood from baseline to week 4 and 8. Following parameters will be analysed:
    - Erythrocyte sedimentation rate (mm/hour)
    - C-reactive protein (CRP) (mg/L)
    - Red blood cell count (number, 10^9/L)

- White blood cell count (number, 10^9/L)
- Platelet count (number, 10^9/L)
- Alanine transaminase (ALAT) (µkat/L)
- Aspartate transaminase (ASAT) (µkat/L)
- Alkaline phosphatase (ALP) (µkat/L)
- Bilirubin (µmol/L)
- Renal function, eGFR (ml/min)
- Blood total protein (g/l)

- Change in glycaemic levels in blood from baseline to week 4 and 8. Following parameters will be analysed:
    - Fasting blood glucose (mmol/l)
    - HBA1c (mmol/mol)
- Relative change in short-chain fatty acids in stool from baseline to week 4, 8 and 10. Following parameters will be analysed:
    - Butyrate
    - Propionate
    - Acetate
    - Lactate
    - Isobutyrate
    - Isovalerate
    - Succinate
- Colonization with *F. prausnitzii* in stool from baseline to week 8.

3.3 Safety Variables

3.3.1 Exposure and Compliance of Investigational Product

Exposure duration in days and compliance, both as continuous and ≤75% vs >75% of planned time, will be summarized. The intake of study medication starts at Visit 2 + 1 day.

3.3.2 Adverse Events

Adverse events (AE) are collected continuously during the study and defined and coded according to Common Terminology Criteria for Adverse Events (CTCAE).

4 STATISTICAL METHODOLOGY

4.1 General Methodology

Categorical variables will be described by number and percentage and 95% confidence interval (CI) for percentages based on binomial distribution, and for continuous variables mean, standard deviation (SD), median, minimum and maximum, and 95% CI for the mean based on normal distribution or bootstrapped, in case data is not normally distributed.

Main comparisons will be made for both doses of *F. prausnitzii* and *D. piger* vs Placebo and secondary for high and low dose separately vs Placebo.

For test between two groups with respect to dichotomous variables Fisher's exact test will be used, and for continuous variables Fisher's non-parametric permutation test will be used.

Primary variable will be described by event rates, number of events divided by follow-up time, and 95% CI will be computed using exact Poisson limits. Time to event data will be described by Kaplan-Meier curves, and primary test between the groups will be performed using Fisher's exact test. The sensitivity analysis will be performed using time-to-event methodology and tested by non-parametric log-rank test.

Secondary variables are all continuous and are not guaranteed to be normally distributed. For this reason the change from baseline to week 4 and week 8 will be tested by applying Fisher's non-parametric permutation test. Mean difference between treatments will be presented along with the 95% CI based on the same permutation test.

Primary and secondary analyses will be performed both for ITT and PP population.

No adjustments for multiple comparisons will be made due to the study being a tolerability and safety study. The differences in parameter estimates between the IP and the placebo will be evaluated for its clinical relevance.

No imputation of data will be made.

All tests will be two-tailed and conducted at 0.05 significance level. All analyses will be performed by using SAS software version 9.4 (SAS Institute Inc., Cary, NC, USA).

4.2 Subject Disposition and Data Sets Analyzed

The number of subjects included in each of the ITT, PP and safety populations will be summarized for each treatment group and *F. prausnitzii* and *D. piger* group in total. The number and percentage of subjects randomized and treated will be presented. Subjects who completed the study and subjects who withdrew from study prematurely will also be presented with a breakdown of the reasons for withdrawal by treatment group.

4.3 Protocol Violations/Deviations

Major protocol deviations are those that are considered to have an effect on the analysis. A list of potential major protocol deviations will be generated programmatically from the data captured before the clean file meeting. The finalisation of the major protocol deviations will be done at the clean file meeting.

The number of subjects with major protocol deviations will be summarized per treatment group.

4.4 Demographics and Baseline Characteristics

Demographics and baseline characteristics will be summarized by treatment group for the ITT and PP populations and analyzed according to the methods described in General Methodology, section 4.1, above.

4.5 Primary and Secondary Analyses

4.5.1 Primary Tolerability Analysis

Primary tolerability analysis is the description of discontinuation rate due to IP and test between both *F. prausnitzii* and *D. piger* treatment groups vs Placebo, performed on ITT population using Fisher's exact test. Percentage of subjects and 95% CI for the percentage using binomial distribution will be described, along with the event rates and 95% CI computed by exact Poisson limits, and incidence rate ratio (IRR) between the groups using Poisson distribution.

Sensitivity analysis will be performed by log-rank test and time to discontinuation will be described using Kaplan-Meier technique.

4.5.2 Secondary Analyses

Secondary analyses include repeated measures data and will be performed using the methodology described in General Methodology, section 4.1, above, by using Fisher's non-parametric permutation test.

Continuous variables will be presented by boxplots and individual spaghettograms.

4.5.3 Exploratory Analyses

The effects of the IP on the gut microbiome will be studied. The gut microbiome will be profiled by whole genome shotgun sequencing of total genomic DNA extracted from stool samples and analysed after removal of human as well as low quality sequences by mapping the sequence data to known microbial genomes. The abundance of microbial taxonomic groups will be analysed prospectively in samples obtained from the subjects at 3 different time points, namely at baseline and then at 8 and 10 weeks during and after the probiotic treatment.

The association between estimated baseline dietary intake and physical activity (both assessed using questionnaires) and effect of IP on all predefined outcomes will be investigated.

4.5.4 Subgroup Analyses

A post-hoc defined subgroup analyses of relative change in short-chain fatty acids in stool from baseline to 4, 8, and 10 weeks, as well as the colonization with *F. prausnitzii* in stool from baseline to week 8 will be performed. The analyses will be performed on all feces samples that are collected exactly according to the protocol. The list of excluded feces samples will be provided along with the reason for exclusion.

4.6 Safety Analyses

4.6.1 Exposure and Compliance of Investigational Product

Duration of IP will be summarized for each treatment group. Compliance will be summarized both as continuous and dichotomous variable (≤75% and >75%).

The summaries will be provided for safety population.

4.6.2 Adverse Events

Only treatment-emergent AEs, i.e. AEs that have occurred after first dose of IP has been taken, will be included in the summaries for safety population.

A summary of subjects reporting at least one of the following AEs will be presented in an overview table:

- Any AE
- Any SAE
- Any treatment-related AE
- Any treatment-related SAE
- Any AE leading to discontinuation

Summaries per SOC and PT presenting n (%) of AEs and n (%) of subjects with at least one AE will be provided for:

- All AEs (includes all serious and non-serious AEs)
- All AEs by maximum reported intensity
- All AEs by causality
- All SAEs
- All AEs leading to discontinuation

5 INTERIM ANALYSES

No interim analysis is defined.

6 CHANGES OF ANALYSIS FROM PROTOCOL

The study protocol stated that secondary analyses will be analysed using either ANOVA in case data is normally distributed or Mann-Whitney U-test for not normally distributed variables. In order to have an exact statistical plan to avoid subjective future decisions, and a method that is suitable for not normally and normally distributed variables, Fisher's non-parametric permutation test will be used. This decision was made by the author of this document and the change was made prior to database lock and prior to data delivery to the statistician.

The protocol specified that species specific analysis of colonisation would be done for *F. prausnitzii* DSM 32379 at study visits from week 0 to 10, but due to methodological limitations

and unforeseen analysis difficulties, only the total amount of *F. prausnitzii* will be analysed for study visits from week 0 and 8.

The post-hoc subgroup analyses of short-chain fatty acids and colonization with *F. prausnitzii* was added to this SAP not previously defined in the protocol.

7 LISTING OF TABLE, FIGURES AND LISTINGS

7.1 Listing of Tables

Table Number	Table Title
14.1.1	Subject Disposition and Data Sets Analyzed (ITT Population)
14.1.2	Protocol Deviations Leading to Exclusion from PP Population (ITT Population)
14.1.3	Protocol Deviations Leading to Exclusion from the Post-hoc Subgroup Analysis (ITT Population)
14.1.3.1	Demographics and Baseline Characteristics (ITT Population)
14.1.3.2	Demographics and Baseline Characteristics (PP Population)
14.2.1.1	Primary Tolerability Analysis (ITT Population)
14.2.1.2	Primary Tolerability Analysis (PP Population)
14.2.x	*Other efficacy variables/analyses*
14.2.x	*Exploratory Analysis – xxx*
14.2.x	*Post-hoc Subgroup Analysis - xxx*
14.3.1	Duration of Exposure and Compliance (Safety Population)
14.3.2.1	Summary of Adverse Events (Safety Population)
14.3.2.2	Adverse Events, by System Organ Class and Preferred Term (Safety Population)
14.3.2.3	Adverse Events, by System Organ Class, Preferred Term and Maximum Reported Intensity (Safety Population)
14.3.2.4	Adverse Events, by System Organ Class, Preferred Term and Causality Assessment (Safety Population)
14.3.2.5	Serious Adverse Events, by System Organ Class and Preferred Term (Safety Population)
14.3.3	Adverse Events Leading to Discontinuation, by System Organ Class and Preferred Term (Safety Population)

7.2 Listing of Figures

Figure Number	Figure Title
14.2.x	*For Primary/Secondary Analyses*

7.3 Listing of Listings

Listing Number	Listing Title
16.2.1	Discontinued Subjects
16.2.2	Subjects with Important Protocol Deviations
16.2.3	Subjects Excluded from the PP Analysis
16.2.4	Demographics and Baseline Characteristics
16.2.5	Compliance and Therapy Exposure
16.2.6	Primary and Secondary Variables
16.2.7	Adverse Events

2019-11-06

Database lock

Documentation regarding META002 - Tolerability and Risk of Adverse Events With a Probiotic Supplement: A Randomised and Placebo Controlled Study in Healthy Individuals

The database Meta002 Complete DB SAP Nov6th has been finalized for analysis according to the statistical analysis plan (SAP) dated 2019-11-03. No further changes in the dataset will be done before unblindning and performing the analysis according to the SAP.

Mattias Lorentzon, 2019-11-06, 4.50 pm
Principal Investigator

Sahlgrenska University Hospital
Mölndal

Tables and Figures to Statistical Report

Study META002

Tolerability and Risk of Adverse Events With a Probiotic Supplement: A Randomised and Placebo Controlled Study in Healthy Individuals

20200102

Authors:
Aldina Pivodic, Statistiska konsultgruppen

Revisions

Version	Description of Changes	Date
1	Draft version 1: Analyses acc to SAP except for PP population. Some data issues needed to be resolved	20191208
2	Updated database regarding couple of AE cases, one compliance value, subgroup population for correctly treated feces samples (the complete patient excluded rather than specific visits).<br>All PP tables added.<br>Additional Post hoc analyses were defined in an amendment to SAP (adjusted for baseline value and age using MMRM) and implemented for short chain fatty acids, tables 14.2.5.4-14.2.5.10<br>Corrected to count only treatment emergent AEs in Table 14.3.2.1	20200102

LIST OF TABLES

Table 14.1.1 Subject Disposition and Data Sets Analyzed (ITT Population) 4
Table 14.1.2 Protocol Deviations Leading to Exclusion from PP Population (ITT Population) 5
Table 14.1.3 Protocol Deviations Leading to Exclusion from the Post-hoc Subgroup Analysis (ITT Population) 6
Table 14.1.4.1 Demographics and Baseline Characteristics (ITT Population) 7
Table 14.1.4.2 Demographics and Baseline Characteristics (PP Population) 10
Table 14.2.1.1 Primary Tolerability Analysis (ITT Population) 13
Table 14.2.1.2 Primary Tolerability Analysis (PP Population) 14
Table 14.2.2.1 Secondary Analysis - Change in GSRS (ITT Population) 15
Table 14.2.2.2 Secondary Analysis - Change in GSRS (PP Population) 16
Table 14.2.3.1 Secondary Analysis - Change in hematologic and biochemical parameters (ITT Population) 17
Table 14.2.3.2 Secondary Analysis - Change in hematologic and biochemical parameters (PP Population) 25
Table 14.2.4.1 Secondary Analysis - Change in glycaemic levels (ITT Population) 33
Table 14.2.4.2 Secondary Analysis - Change in glycaemic levels (PP Population) 35
Table 14.2.5.1 Secondary Analysis - Relative change in short-chain fatty acids in stool (ITT Population) 37
Table 14.2.5.2 Secondary Analysis - Relative change in short-chain fatty acids in stool (PP Population) 43
Table 14.2.5.3 Post-hoc Subgroup Analysis - Relative change in short-chain fatty acids in stool (Subgroup population according to the protocol for feces analyses) 49
Table 14.2.5.4 Posthoc Analyses - Butyrate - MMRM on various populations 55
Table 14.2.5.5 Posthoc Analyses - Propionate - MMRM on various populations 58
Table 14.2.5.6 Posthoc Analyses - Acetate - MMRM on various populations 61
Table 14.2.5.7 Posthoc Analyses - Lactate - MMRM on various populations 64
Table 14.2.5.8 Posthoc Analyses - Isobutyrate - MMRM on various populations 67
Table 14.2.5.9 Posthoc Analyses - Isovalerate - MMRM on various populations 70
Table 14.2.5.10 Posthoc Analyses - Succinate - MMRM on various populations 73
Table 14.2.6.1 Secondary Analysis - Colonization with F. prausnitzii and D. Piger in stool (ITT Population) 76
Table 14.2.6.2 Secondary Analysis - Colonization with F. prausnitzii and D. Piger in stool (PP Population) 78
Table 14.2.6.3 Post-hoc Subgroup Analysis - Colonization with F. prausnitzii and D. Piger in stool (Subgroup population according to the protocol for feces analyses) 80
Table 14.3.1 Duration of Exposure and Compliance (Safety Population) 83
Table 14.3.2.1 Summary of Adverse Events (Safety Population) 84
Table 14.3.2.2 Adverse Events, by System Organ Class and Preferred Term (Safety Population) 85
Table 14.3.2.3 Adverse Events, by System Organ Class, Preferred Term and Maximum Reported Intensity (Safety Population) 86
Table 14.3.2.4 Adverse Events, by System Organ Class, Preferred Term and Causality Assessment (Safety Population) 87

LIST OF FIGURES

Figure 14.2.2.1.1 Boxplots for GSRS (score 0-45) over time 88
Figure 14.2.2.1.2 Individual values for GSRS (score 0-45) over time 88
Figure 14.2.3.1.1 Boxplots for Erythrocyte sedimentation rate (mm/hour) over time 89
Figure 14.2.3.1.2 Individual values for Erythrocyte sedimentation rate (mm/hour) over time 89
Figure 14.2.3.2.1 Boxplots for C-reactive protein (mg/L) over time 90
Figure 14.2.3.2.2 Individual values for C-reactive protein (mg/L) over time 90
Figure 14.2.3.3.1 Boxplots for Red blood cell count (10^9/L) over time 91
Figure 14.2.3.3.2 Individual values for Red blood cell count (10^9/L) over time 91
Figure 14.2.3.4.1 Boxplots for White blood cell count (10^9/L) over time 92
Figure 14.2.3.4.2 Individual values for White blood cell count (10^9/L) over time 92

Figure 14.2.3.5.1 Boxplots for Platelet count (10^9/L) over time 93
Figure 14.2.3.5.2 Individual values for Platelet count (10^9/L) over time 93
Figure 14.2.3.6.1 Boxplots for Alanine transaminase (µkat/L) over time 94
Figure 14.2.3.6.2 Individual values for Alanine transaminase (µkat/L) over time 94
Figure 14.2.3.7.1 Boxplots for Aspartate transaminase (µkat/L) over time 95
Figure 14.2.3.7.2 Individual values for Aspartate transaminase (µkat/L) over time 95
Figure 14.2.3.8.1 Boxplots for Alkaline phosphatase (µkat/L) over time 96
Figure 14.2.3.8.2 Individual values for Alkaline phosphatase (µkat/L) over time 96
Figure 14.2.3.9.1 Boxplots for Bilirubin (µmol/L) over time 97
Figure 14.2.3.9.2 Individual values for Bilirubin (µmol/L) over time 97
Figure 14.2.3.10.1 Boxplots for Renal function eGFR (ml/min) over time 98
Figure 14.2.3.10.2 Individual values for Renal function eGFR (ml/min) over time 98
Figure 14.2.3.11.1 Boxplots for Blood total protein (g/l) over time 99
Figure 14.2.3.11.2 Individual values for Blood total protein (g/l) over time 99
Figure 14.2.3.12.1 Boxplots for Blood haemoglobin (g/l) over time 100
Figure 14.2.3.12.2 Individual values for Blood haemoglobin (g/l) over time 100
Figure 14.2.4.1.1 Boxplots for Fasting blood glucose (mmol/L) over time 101
Figure 14.2.4.1.2 Individual values for Fasting blood glucose (mmol/L) over time 101
Figure 14.2.4.2.1 Boxplots for Blood HBA1C (mmol/mol) over time 102
Figure 14.2.4.2.2 Individual values for Blood HBA1C (mmol/mol) over time 102
Figure 14.2.5.1.1 Boxplots for Butyrate over time 103
Figure 14.2.5.1.2 Individual values for Butyrate over time 103
Figure 14.2.5.2.1 Boxplots for Propionate over time 104
Figure 14.2.5.2.2 Individual values for Propionate over time 104
Figure 14.2.5.3.1 Boxplots for Acetate over time 105
Figure 14.2.5.3.2 Individual values for Acetate over time 105
Figure 14.2.5.4.1 Boxplots for Lactate over time 106
Figure 14.2.5.4.2 Individual values for Lactate over time 106
Figure 14.2.5.5.1 Boxplots for Isobutyrate over time 107
Figure 14.2.5.5.2 Individual values for Isobutyrate over time 107
Figure 14.2.5.6.1 Boxplots for Isovalerate over time 108
Figure 14.2.5.6.2 Individual values for Isovalerate over time 108
Figure 14.2.5.7.1 Boxplots for Succinate over time 109
Figure 14.2.5.7.2 Individual values for Succinate over time 109
Figure 14.2.6.1.1 Boxplots for Colonization with F. prausnitzii in stool SL3.3 over time 110
Figure 14.2.6.1.2 Individual values for Colonization with F. prausnitzii in stool SL3.3 over time 110
Figure 14.2.6.2.1 Boxplots for Colonization with F. prausnitzii in stool L2.6 over time 111
Figure 14.2.6.2.2 Individual values for Colonization with F. prausnitzii in stool L2.6 over time 111
Figure 14.2.6.3.1 Boxplots for Colonization with F. prausnitzii in stool KLE1255 over time 112
Figure 14.2.6.3.2 Individual values for Colonization with F. prausnitzii in stool KLE1255 over time 112
Figure 14.2.6.4.1 Boxplots for Colonization with F. prausnitzii in stool A2.165 over time 113
Figure 14.2.6.4.2 Individual values for Colonization with F. prausnitzii in stool A2.165 over time 113
Figure 14.2.6.5.1 Boxplots for Colonization with F. prausnitzii in stool Total over time 114
Figure 14.2.6.5.2 Individual values for Colonization with F. prausnitzii in stool Total over time 114
Figure 14.2.6.6.1 Boxplots for Colonization with D. Piger in stool over time 115
Figure 14.2.6.6.2 Individual values for Colonization with D. Piger in stool over time 115

Table 14.1.1 Subject Disposition and Data Sets Analyzed (ITT Population)

	Total (n=50)	High dose (n=18)	Low dose (n=16)	Placebo (n=16)
**ITT population**	50 (100.0%)	18 (100.0%)	16 (100.0%)	16 (100.0%)
**PP population**	49 (98.0%)	18 (100.0%)	15 (93.8%)	16 (100.0%)
**Safety population**	50 (100.0%)	18 (100.0%)	16 (100.0%)	16 (100.0%)
**Subgroup population according to the protocol for feces analyses**	46 (92.0%)	16 (88.9%)	14 (87.5%)	16 (100.0%)
**Early termination**				
**No**	46 (92.0%)	16 (88.9%)	16 (100.0%)	14 (87.5%)
**Yes**	4 (8.0%)	2 (11.1%)	0 (0.0%)	2 (12.5%)
**Reason for early termination**				
**subject initiated**	4	2	0	2

For categorical variables n (%) is presented.

2020-01-01 Analys.sas

Table 14.1.2 Protocol Deviations Leading to Exclusion from PP Population (ITT Population)

Subject ID	Enrolment number	Treatment group	Reason for exclusion from PP
HP4	E242	Low dose	studiedeltagaren har ej noterat när provet är taget
2020-01-01 Analys.sas			

Table 14.1.3 Protocol Deviations Leading to Exclusion from the Post-hoc Subgroup Analysis (ITT Population)

Subject ID	Enrolment number	Treatment group	Reason for exclusion from the subgroup analysis of feces samples handled as per protocol
HP4	E242	Low dose	Datum saknas för fecesprover V2 och V3
IO5	E152	High dose	Datum saknas för fecesprov V2
JV5	E246	High dose	För gammalt (2.5 dgr) fecesprov vid V
RW5	E226	Low dose	För gammalt fecesprov (4dgr) vid V6
2020-01-01 Analys.sas			

Table 14.1.4.1 Demographics and Baseline Characteristics (ITT Population)

					Test between groups		
Variable	High+Low dose (n=34)	High dose (n=18)	Low dose (n=16)	Placebo (n=16)	High+Low dose vs Placebo	High dose vs Placebo	Low dose vs Placebo
**Age (years)**	29.8 (5.7)<br>30 (20; 40)<br>n=34	29.1 (6.3)<br>28.5 (20; 39)<br>n=18	30.5 (4.9)<br>31 (21; 40)<br>n=16	26.3 (4.6)<br>25.5 (20; 38)<br>n=16	0.035	0.15	0.020
**Sex**							
**Female**	18 (52.9%)	10 (55.6%)	8 (50.0%)	8 (50.0%)			
**Male**	16 (47.1%)	8 (44.4%)	8 (50.0%)	8 (50.0%)	1.00	1.00	1.00
**Body Mass Index (kg/m2)**	24.8 (3.8)<br>24.4 (18.1; 35.7)<br>n=34	25.3 (4.6)<br>24.4 (18.1; 35.7)<br>n=18	24.3 (2.8)<br>24.1 (19.6; 28.1)<br>n=16	23.8 (3.3)<br>23.5 (20.3; 34)<br>n=16	0.37	0.30	0.64
**Waist to hip ratio**	0.798 (0.078)<br>0.798 (0.63; 0.98)<br>n=34	0.808 (0.093)<br>0.803 (0.63; 0.98)<br>n=18	0.787 (0.059)<br>0.796 (0.692; 0.896)<br>n=16	0.799 (0.087)<br>0.8 (0.688; 1.009)<br>n=16	0.97	0.78	0.65
**Systolic blood pressure (mmHg)**	117.9 (12.4)<br>116.8 (92; 157)<br>n=34	121.9 (11.9)<br>120 (101.5; 157)<br>n=18	113.4 (11.7)<br>113.5 (92; 138.5)<br>n=16	117.2 (8.5)<br>116.8 (102; 139)<br>n=16	0.85	0.20	0.31
**Diastolic blood pressure (mmHg)**	69.9 (9.0)<br>69.3 (55; 94.5)<br>n=34	71.6 (9.3)<br>70.3 (57.5; 94.5)<br>n=18	68.0 (8.4)<br>68.3 (55; 85)<br>n=16	67.2 (7.1)<br>66.5 (55.5; 83)<br>n=16	0.29	0.13	0.78
**GSRS (score 0-45) at randomization**	3.74 (3.23)<br>3 (0; 12)<br>n=34	3.69 (3.44)<br>2.75 (0; 12)<br>n=18	3.78 (3.09)<br>3 (0; 11)<br>n=16	2.69 (1.59)<br>2.5 (1; 7.5)<br>n=16	0.23	0.31	0.24
**Blood samples**							
**Renal function eGFR (ml/min) at screening**	90.6 (10.0)<br>91 (71; 115)<br>n=34	90.1 (8.8)<br>91 (71; 104)<br>n=18	91.3 (11.4)<br>90 (71; 115)<br>n=16	89.6 (9.3)<br>87.5 (76; 117)<br>n=16	0.73	0.88	0.66
**Blood haemoglobin (g/l) at screening**	140.2 (13.2)<br>139.5 (118; 164)<br>n=34	140.0 (13.9)<br>139.5 (118; 164)<br>n=18	140.4 (13.0)<br>141 (124; 159)<br>n=16	141.8 (14.6)<br>140 (123; 169)<br>n=16	0.71	0.72	0.79
**Blood total protein (g/l) at screening**	70.5 (4.6)<br>70 (62; 80)<br>n=33	70.9 (4.9)<br>72.5 (62; 80)<br>n=18	70.1 (4.2)<br>70 (64; 78)<br>n=15	72.9 (5.1)<br>73 (62; 81)<br>n=16	0.12	0.27	0.12
**Fasting blood glucose (mmol/L) at screening**	5.15 (0.40)<br>5.15 (4.5; 6)<br>n=34	5.29 (0.44)<br>5.25 (4.5; 6)<br>n=18	5.00 (0.28)<br>5 (4.5; 5.5)<br>n=16	5.21 (0.35)<br>5.25 (4.6; 5.7)<br>n=16	0.68	0.58	0.082

					Test between groups		
**Variable**	**High+Low dose (n=34)**	**High dose (n=18)**	**Low dose (n=16)**	**Placebo (n=16)**	**High+Low dose vs Placebo**	**High dose vs Placebo**	**Low dose vs Placebo**
**Blood HBA1C (mmol/mol) at screening**	30.4 (2.6)<br>30 (26; 39)<br>n=34	30.6 (3.2)<br>30 (26; 39)<br>n=18	30.2 (1.8)<br>30.5 (26; 33)<br>n=16	30.4 (2.0)<br>31 (26; 33)<br>n=16	1.00	0.91	0.84
**Erythrocyte sedimentation rate (mm/hour) at screening**	4.66 (0.45)<br>4.6 (3.7; 5.4)<br>n=34	4.67 (0.47)<br>4.6 (3.7; 5.4)<br>n=18	4.65 (0.45)<br>4.65 (3.8; 5.4)<br>n=16	4.70 (0.57)<br>4.65 (3.9; 5.6)<br>n=16	0.83	0.90	0.80
**C-reactive protein (mg/L) at screening**	0.912 (3.630)<br>0 (0; 21)<br>n=34	1.50 (4.94)<br>0 (0; 21)<br>n=18	0.250 (0.683)<br>0 (0; 2)<br>n=16	0.938 (2.265)<br>0 (0; 9)<br>n=16	0.82	0.87	0.36
**Red blood cell count (10^9/L) at screening**	241.0 (44.1)<br>242 (172; 331)<br>n=34	253.6 (49.9)<br>261 (173; 331)<br>n=18	226.9 (32.5)<br>229 (172; 295)<br>n=16	238.1 (41.8)<br>235 (175; 324)<br>n=16	0.82	0.34	0.41
**White blood cell count (10^9/L) at screening**	4.73 (1.16)<br>4.5 (2.9; 7.1)<br>n=34	4.83 (1.28)<br>4.65 (2.9; 7.1)<br>n=18	4.61 (1.03)<br>4.15 (3.6; 6.8)<br>n=16	4.91 (0.87)<br>5.05 (3.6; 6.7)<br>n=16	0.59	0.86	0.39
**Platelet count (10^9/L) at screening**	6.03 (6.74)<br>3 (1; 29)<br>n=34	7.33 (8.35)<br>3 (1; 29)<br>n=18	4.56 (4.10)<br>3 (1; 15)<br>n=16	6.69 (4.29)<br>5 (2; 16)<br>n=16	0.73	0.79	0.17
**Alanine transaminase (µkat/L) at screening**	0.352 (0.151)<br>0.315 (0.13; 0.77)<br>n=34	0.407 (0.175)<br>0.345 (0.21; 0.77)<br>n=18	0.290 (0.090)<br>0.3 (0.13; 0.47)<br>n=16	0.418 (0.202)<br>0.35 (0.19; 0.83)<br>n=16	0.21	0.86	0.028
**Aspartate transaminase (µkat/L) at screening**	0.417 (0.157)<br>0.37 (0.28; 1.2)<br>n=34	0.429 (0.207)<br>0.355 (0.28; 1.2)<br>n=18	0.403 (0.073)<br>0.38 (0.29; 0.55)<br>n=16	0.472 (0.123)<br>0.425 (0.26; 0.69)<br>n=16	0.28	0.53	0.069
**Alkaline phosphatase (µkat/L) at screening**	0.987 (0.228)<br>0.94 (0.65; 1.6)<br>n=34	0.923 (0.150)<br>0.915 (0.68; 1.3)<br>n=18	1.06 (0.28)<br>0.99 (0.65; 1.6)<br>n=16	1.15 (0.38)<br>1.1 (0.44; 1.6)<br>n=16	0.076	0.030	0.45
**Bilirubin (µmol/L) at screening**	11.4 (8.0)<br>9 (3.8; 37)<br>n=34	10.4 (7.7)<br>8.1 (3.8; 37)<br>n=18	12.5 (8.5)<br>9.5 (5.3; 37)<br>n=16	10.5 (5.0)<br>9.5 (3.7; 23)<br>n=16	0.73	0.95	0.45
**Colonization with F. prausnitzii in stool**							
**Colonization with F. prausnitzii in stool SL3.3 at randomization**	399113 (300581)<br>261943 (64423; 1322383)<br>n=30	486085 (381409)<br>386948 (87115; 1322383)<br>n=14	323012 (188047)<br>250282 (64423; 648169)<br>n=16	447790 (427354)<br>344404 (65739; 1495280)<br>n=13	0.65	0.82	0.32
**Colonization with F. prausnitzii in stool L2.6 at randomization**	445500 (455431)<br>296051 (97458; 2395226)<br>n=30	473748 (604906)<br>282870 (97458; 2395226)<br>n=14	420783 (287205)<br>298545 (155219; 1110434)<br>n=16	407073 (270661)<br>334194 (105446; 910201)<br>n=13	0.88	0.82	0.90

					Test between groups		
Variable	High+Low dose (n=34)	High dose (n=18)	Low dose (n=16)	Placebo (n=16)	High+Low dose vs Placebo	High dose vs Placebo	Low dose vs Placebo
**Colonization with F. prausnitzii in stool KLE1255 at randomization**	421180 (455384)<br>317422 (56316; 2104040)<br>n=30	388569 (440720)<br>298151 (56316; 1795932)<br>n=14	449714 (480340)<br>325233 (64786; 2104040)<br>n=16	462245 (308874)<br>349653 (147476; 1214343)<br>n=13	0.74	0.65	0.92
**Colonization with F. prausnitzii in stool A2.165 at randomization**	475369 (558059)<br>282274 (68859; 2981157)<br>n=30	406020 (320415)<br>275428 (117096; 1027944)<br>n=14	536050 (710426)<br>292417 (68859; 2981157)<br>n=16	447652 (240108)<br>515762 (108885; 882020)<br>n=13	0.98	0.72	0.86
**Colonization with F. prausnitzii in stool Total at randomization**	1741162 (1032122)<br>1492610 (503848; 4691391)<br>n=30	1754422 (1020550)<br>1492610 (532972; 4691391)<br>n=14	1729559 (1075441)<br>1500193 (503848; 4531826)<br>n=16	1764760 (783823)<br>1791817 (533480; 3106899)<br>n=13	0.91	0.98	0.91
**Stool short chain fatty acids (nmol/mg dry weight)**							
**Butyrate at randomization**	54.0 (43.2)<br>40.1 (6.2; 221.3)<br>n=32	56.7 (52.5)<br>33.2 (6.2; 221.3)<br>n=16	51.4 (33.0)<br>41.6 (11.7; 122.7)<br>n=16	85.3 (60.9)<br>70.1 (23.8; 241.4)<br>n=14	0.064	0.18	0.064
**Propionate at randomization**	83.0 (75.1)<br>58.3 (10.7; 393.5)<br>n=32	76.2 (53.2)<br>64.5 (10.7; 190)<br>n=16	89.7 (93.5)<br>51.1 (17.2; 393.5)<br>n=16	155.2 (174.8)<br>79.5 (27.9; 651.5)<br>n=14	0.074	0.090	0.22
**Acetate at randomization**	222.4 (120.5)<br>207.7 (56.8; 597.7)<br>n=32	222.0 (130.2)<br>203.4 (56.8; 597.7)<br>n=16	222.9 (114.1)<br>207.7 (71.2; 485.5)<br>n=16	352.6 (213.3)<br>293.3 (98; 719.3)<br>n=14	0.015	0.047	0.044
**Lactate at randomization**	0.458 (0.418)<br>0.262 (0.106; 1.419)<br>n=32	0.454 (0.429)<br>0.22 (0.118; 1.367)<br>n=16	0.461 (0.421)<br>0.308 (0.106; 1.419)<br>n=16	0.980 (1.446)<br>0.494 (0.098; 5.712)<br>n=14	0.086	0.16	0.18
**Isobutyrate at randomization**	10.5 (4.7)<br>9.8 (2.8; 20.6)<br>n=32	10.0 (4.8)<br>9.7 (2.8; 20.6)<br>n=16	11.1 (4.7)<br>9.8 (4.6; 19)<br>n=16	10.4 (6.0)<br>9.3 (2.8; 27.7)<br>n=14	0.96	0.83	0.75
**Isovalerate at randomization**	8.26 (3.50)<br>8.37 (2.51; 14.79)<br>n=32	7.96 (3.65)<br>8.12 (2.51; 14.27)<br>n=16	8.56 (3.43)<br>8.47 (3.65; 14.79)<br>n=16	7.61 (4.98)<br>6.88 (1.61; 23)<br>n=14	0.64	0.85	0.58
**Succinate at randomization**	3.39 (14.19)<br>0.78 (0.15; 81.05)<br>n=32	0.911 (0.871)<br>0.681 (0.189; 3.104)<br>n=16	5.86 (20.06)<br>0.91 (0.15; 81.05)<br>n=16	28.4 (89.8)<br>0.9 (0.2; 337.3)<br>n=14	0.21	0.17	0.49

For categorical variables n (%) is presented.
For continuous variables Mean (SD) / Median (Min; Max) / n= is presented.
For comparison between groups Fisher´s Exact test (lowest 1-sided p-value multiplied by 2) was used for dichotomous variables and the Fisher´s Non Parametric Permutation Test was used for continuous variables.
2020-01-01 Analys.sas

Table 14.1.4.2 Demographics and Baseline Characteristics (PP Population)

					Test between groups		
**Variable**	**High+Low dose (n=33)**	**High dose (n=18)**	**Low dose (n=15)**	**Placebo (n=16)**	**High+Low dose vs Placebo**	**High dose vs Placebo**	**Low dose vs Placebo**
**Age (years)**	29.8 (5.8)<br>30 (20; 40)<br>n=33	29.1 (6.3)<br>28.5 (20; 39)<br>n=18	30.5 (5.1)<br>31 (21; 40)<br>n=15	26.3 (4.6)<br>25.5 (20; 38)<br>n=16	0.040	0.15	0.023
**Sex**							
**Female**	18 (54.5%)	10 (55.6%)	8 (53.3%)	8 (50.0%)			
**Male**	15 (45.5%)	8 (44.4%)	7 (46.7%)	8 (50.0%)	1.00	1.00	1.00
**Body Mass Index (kg/m2)**	24.8 (3.9)<br>24.3 (18.1; 35.7)<br>n=33	25.3 (4.6)<br>24.4 (18.1; 35.7)<br>n=18	24.2 (2.8)<br>23.5 (19.6; 28.1)<br>n=15	23.8 (3.3)<br>23.5 (20.3; 34)<br>n=16	0.40	0.30	0.73
**Waist to hip ratio**	0.796 (0.079)<br>0.794 (0.63; 0.98)<br>n=33	0.808 (0.093)<br>0.803 (0.63; 0.98)<br>n=18	0.783 (0.058)<br>0.789 (0.692; 0.896)<br>n=15	0.799 (0.087)<br>0.8 (0.688; 1.009)<br>n=16	0.90	0.78	0.53
**Systolic blood pressure (mmHg)**	117.3 (12.0)<br>116.5 (92; 157)<br>n=33	121.9 (11.9)<br>120 (101.5; 157)<br>n=18	111.8 (9.9)<br>112.5 (92; 131)<br>n=15	117.2 (8.5)<br>116.8 (102; 139)<br>n=16	1.00	0.20	0.11
**Diastolic blood pressure (mmHg)**	69.5 (8.9)<br>69 (55; 94.5)<br>n=33	71.6 (9.3)<br>70.3 (57.5; 94.5)<br>n=18	67.1 (7.8)<br>67.5 (55; 85)<br>n=15	67.2 (7.1)<br>66.5 (55.5; 83)<br>n=16	0.36	0.13	0.98
**GSRS (score 0-45) at randomization**	3.64 (3.23)<br>3 (0; 12)<br>n=33	3.69 (3.44)<br>2.75 (0; 12)<br>n=18	3.57 (3.07)<br>3 (0; 11)<br>n=15	2.69 (1.59)<br>2.5 (1; 7.5)<br>n=16	0.29	0.31	0.35
**Blood samples**							
**Renal function eGFR (ml/min) at screening**	90.4 (10.0)<br>91 (71; 115)<br>n=33	90.1 (8.8)<br>91 (71; 104)<br>n=18	90.7 (11.5)<br>90 (71; 115)<br>n=15	89.6 (9.3)<br>87.5 (76; 117)<br>n=16	0.80	0.88	0.78
**Blood haemoglobin (g/l) at screening**	140.1 (13.4)<br>139 (118; 164)<br>n=33	140.0 (13.9)<br>139.5 (118; 164)<br>n=18	140.3 (13.4)<br>139 (124; 159)<br>n=15	141.8 (14.6)<br>140 (123; 169)<br>n=16	0.70	0.72	0.77
**Blood total protein (g/l) at screening**	70.6 (4.6)<br>70.5 (62; 80)<br>n=32	70.9 (4.9)<br>72.5 (62; 80)<br>n=18	70.1 (4.3)<br>69.5 (64; 78)<br>n=14	72.9 (5.1)<br>73 (62; 81)<br>n=16	0.13	0.27	0.14
**Fasting blood glucose (mmol/L) at screening**	5.16 (0.40)<br>5.2 (4.5; 6)<br>n=33	5.29 (0.44)<br>5.25 (4.5; 6)<br>n=18	5.00 (0.29)<br>5 (4.5; 5.5)<br>n=15	5.21 (0.35)<br>5.25 (4.6; 5.7)<br>n=16	0.71	0.58	0.095

					Test between groups		
Variable	High+Low dose (n=33)	High dose (n=18)	Low dose (n=15)	Placebo (n=16)	High+Low dose vs Placebo	High dose vs Placebo	Low dose vs Placebo
**Blood HBA1C (mmol/mol) at screening**	30.5 (2.5)<br>30 (26; 39)<br>n=33	30.6 (3.2)<br>30 (26; 39)<br>n=18	30.5 (1.4)<br>31 (28; 33)<br>n=15	30.4 (2.0)<br>31 (26; 33)<br>n=16	0.92	0.91	0.96
**Erythrocyte sedimentation rate (mm/hour) at screening**	4.65 (0.46)<br>4.6 (3.7; 5.4)<br>n=33	4.67 (0.47)<br>4.6 (3.7; 5.4)<br>n=18	4.63 (0.46)<br>4.6 (3.8; 5.4)<br>n=15	4.70 (0.57)<br>4.65 (3.9; 5.6)<br>n=16	0.79	0.90	0.75
**C-reactive protein (mg/L) at screening**	0.939 (3.682)<br>0 (0; 21)<br>n=33	1.50 (4.94)<br>0 (0; 21)<br>n=18	0.267 (0.704)<br>0 (0; 2)<br>n=15	0.938 (2.265)<br>0 (0; 9)<br>n=16	0.84	0.87	0.42
**Red blood cell count (10^9/L) at screening**	240.8 (44.8)<br>242 (172; 331)<br>n=33	253.6 (49.9)<br>261 (173; 331)<br>n=18	225.5 (33.2)<br>228 (172; 295)<br>n=15	238.1 (41.8)<br>235 (175; 324)<br>n=16	0.84	0.34	0.37
**White blood cell count (10^9/L) at screening**	4.72 (1.18)<br>4.4 (2.9; 7.1)<br>n=33	4.83 (1.28)<br>4.65 (2.9; 7.1)<br>n=18	4.59 (1.07)<br>4.1 (3.6; 6.8)<br>n=15	4.91 (0.87)<br>5.05 (3.6; 6.7)<br>n=16	0.59	0.86	0.40
**Platelet count (10^9/L) at screening**	6.00 (6.85)<br>3 (1; 29)<br>n=33	7.33 (8.35)<br>3 (1; 29)<br>n=18	4.40 (4.19)<br>3 (1; 15)<br>n=15	6.69 (4.29)<br>5 (2; 16)<br>n=16	0.73	0.79	0.16
**Alanine transaminase (µkat/L) at screening**	0.352 (0.154)<br>0.31 (0.13; 0.77)<br>n=33	0.407 (0.175)<br>0.345 (0.21; 0.77)<br>n=18	0.286 (0.092)<br>0.29 (0.13; 0.47)<br>n=15	0.418 (0.202)<br>0.35 (0.19; 0.83)<br>n=16	0.22	0.86	0.025
**Aspartate transaminase (µkat/L) at screening**	0.414 (0.158)<br>0.37 (0.28; 1.2)<br>n=33	0.429 (0.207)<br>0.355 (0.28; 1.2)<br>n=18	0.395 (0.069)<br>0.37 (0.29; 0.55)<br>n=15	0.472 (0.123)<br>0.425 (0.26; 0.69)<br>n=16	0.25	0.53	0.045
**Alkaline phosphatase (µkat/L) at screening**	0.978 (0.224)<br>0.94 (0.65; 1.6)<br>n=33	0.923 (0.150)<br>0.915 (0.68; 1.3)<br>n=18	1.04 (0.28)<br>0.97 (0.65; 1.6)<br>n=15	1.15 (0.38)<br>1.1 (0.44; 1.6)<br>n=16	0.064	0.030	0.39
**Bilirubin (µmol/L) at screening**	11.5 (8.1)<br>9 (3.8; 37)<br>n=33	10.4 (7.7)<br>8.1 (3.8; 37)<br>n=18	12.9 (8.6)<br>10 (5.3; 37)<br>n=15	10.5 (5.0)<br>9.5 (3.7; 23)<br>n=16	0.68	0.95	0.37
**Colonization with F. prausnitzii in stool**							
**Colonization with F. prausnitzii in stool SL3.3 at randomization**	404008 (304682)<br>266737 (64423; 1322383)<br>n=29	486085 (381409)<br>386948 (87115; 1322383)<br>n=14	327403 (193796)<br>243415 (64423; 648169)<br>n=15	447790 (427354)<br>344404 (65739; 1495280)<br>n=13	0.68	0.82	0.36
**Colonization with F. prausnitzii in stool L2.6 at randomization**	450817 (462543)<br>300793 (97458; 2395226)<br>n=29	473748 (604906)<br>282870 (97458; 2395226)<br>n=14	429414 (295129)<br>305780 (155219; 1110434)<br>n=15	407073 (270661)<br>334194 (105446; 910201)<br>n=13	0.86	0.82	0.84

					Test between groups		
Variable	High+Low dose (n=33)	High dose (n=18)	Low dose (n=15)	Placebo (n=16)	High+Low dose vs Placebo	High dose vs Placebo	Low dose vs Placebo
**Colonization with F. prausnitzii in stool KLE1255 at randomization**	430738 (460372)<br>327602 (56316; 2104040)<br>n=29	388569 (440720)<br>298151 (56316; 1795932)<br>n=14	470096 (489986)<br>343225 (64786; 2104040)<br>n=15	462245 (308874)<br>349653 (147476; 1214343)<br>n=13	0.79	0.65	1.00
**Colonization with F. prausnitzii in stool A2.165 at randomization**	487816 (563683)<br>283563 (68859; 2981157)<br>n=29	406020 (320415)<br>275428 (117096; 1027944)<br>n=14	564160 (726092)<br>303848 (68859; 2981157)<br>n=15	447652 (240108)<br>515762 (108885; 882020)<br>n=13	0.97	0.72	0.76
**Colonization with F. prausnitzii in stool Total at randomization**	1773379 (1034925)<br>1596111 (503848; 4691391)<br>n=29	1754422 (1020550)<br>1492610 (532972; 4691391)<br>n=14	1791073 (1083659)<br>1675642 (503848; 4531826)<br>n=15	1764760 (783823)<br>1791817 (533480; 3106899)<br>n=13	0.98	0.98	0.95
**Stool short chain fatty acids (nmol/mg dry weight)**							
**Butyrate at randomization**	54.6 (43.8)<br>40.7 (6.2; 221.3)<br>n=31	56.7 (52.5)<br>33.2 (6.2; 221.3)<br>n=16	52.4 (33.9)<br>42.5 (11.7; 122.7)<br>n=15	85.3 (60.9)<br>70.1 (23.8; 241.4)<br>n=14	0.075	0.18	0.083
**Propionate at randomization**	84.4 (75.9)<br>63.9 (10.7; 393.5)<br>n=31	76.2 (53.2)<br>64.5 (10.7; 190)<br>n=16	93.2 (95.7)<br>52.7 (17.2; 393.5)<br>n=15	155.2 (174.8)<br>79.5 (27.9; 651.5)<br>n=14	0.081	0.090	0.26
**Acetate at randomization**	225.8 (120.9)<br>212.7 (56.8; 597.7)<br>n=31	222.0 (130.2)<br>203.4 (56.8; 597.7)<br>n=16	229.9 (114.5)<br>212.7 (71.2; 485.5)<br>n=15	352.6 (213.3)<br>293.3 (98; 719.3)<br>n=14	0.021	0.047	0.066
**Lactate at randomization**	0.465 (0.423)<br>0.299 (0.106; 1.419)<br>n=31	0.454 (0.429)<br>0.22 (0.118; 1.367)<br>n=16	0.477 (0.430)<br>0.316 (0.106; 1.419)<br>n=15	0.980 (1.446)<br>0.494 (0.098; 5.712)<br>n=14	0.10	0.16	0.21
**Isobutyrate at randomization**	10.6 (4.8)<br>10.1 (2.8; 20.6)<br>n=31	10.0 (4.8)<br>9.7 (2.8; 20.6)<br>n=16	11.2 (4.8)<br>10.1 (4.6; 19)<br>n=15	10.4 (6.0)<br>9.3 (2.8; 27.7)<br>n=14	0.94	0.83	0.71
**Isovalerate at randomization**	8.25 (3.56)<br>8.34 (2.51; 14.79)<br>n=31	7.96 (3.65)<br>8.12 (2.51; 14.27)<br>n=16	8.55 (3.55)<br>8.4 (3.65; 14.79)<br>n=15	7.61 (4.98)<br>6.88 (1.61; 23)<br>n=14	0.66	0.85	0.59
**Succinate at randomization**	3.47 (14.42)<br>0.8 (0.15; 81.05)<br>n=31	0.911 (0.871)<br>0.681 (0.189; 3.104)<br>n=16	6.21 (20.71)<br>0.91 (0.15; 81.05)<br>n=15	28.4 (89.8)<br>0.9 (0.2; 337.3)<br>n=14	0.22	0.17	0.52

For categorical variables n (%) is presented.
For continuous variables Mean (SD) / Median (Min; Max) / n= is presented.
For comparison between groups Fisher´s Exact test (lowest 1-sided p-value multiplied by 2) was used for dichotomous variables and the Fisher´s Non Parametric Permutation Test was used for continuous variables.
2020-01-01 Analys.sas

Table 14.2.1.1 Primary Tolerability Analysis (ITT Population)

					Difference in percentages and test between groups		
**Variable**	**High+Low dose (n=34)**	**High dose (n=18)**	**Low dose (n=16)**	**Placebo (n=16)**	**High+Low dose vs Placebo**	**High dose vs Placebo**	**Low dose vs Placebo**
**Early termination due to IP**							
**No**	34 (100.0%)	18 (100.0%)	16 (100.0%)	16 (100.0%)	0.0% (-4.6%;4.6%) p=1.00	0.0% (-5.9%;5.9%) p=1.00	0.0% (-6.3%;6.3%) p=1.00

For categorical variables n (%) is presented.
For comparison between groups Fisher´s Exact test (lowest 1-sided p-value multiplied by 2) was used for dichotomous variables.
2020-01-01 Analys.sas

Table 14.2.1.2 Primary Tolerability Analysis (PP Population)

Variable	High+Low dose (n=33)	High dose (n=18)	Low dose (n=15)	Placebo (n=16)	Difference in percentages and test between groups: High+Low dose vs Placebo	High dose vs Placebo	Low dose vs Placebo
**Early termination due to IP**							
**No**	33 (100.0%)	18 (100.0%)	15 (100.0%)	16 (100.0%)	0.0% (-4.6%;4.6%) p=1.00	0.0% (-5.9%;5.9%) p=1.00	0.0% (-6.5%;6.5%) p=1.00

For categorical variables n (%) is presented.
For comparison between groups Fisher´s Exact test (lowest 1-sided p-value multiplied by 2) was used for dichotomous variables.
2020-01-01 Analys.sas

Table 14.2.2.1 Secondary Analysis - Change in GSRS (ITT Population)

					Difference and test between groups		
**Variable**	**High+Low dose (n=34)**	**High dose (n=18)**	**Low dose (n=16)**	**Placebo (n=16)**	**High+Low dose vs Placebo**	**High dose vs Placebo**	**Low dose vs Placebo**
**GSRS (score 0-45) at randomization**	3.74 (3.23)<br>3 (0; 12)<br>n=34	3.69 (3.44)<br>2.75 (0; 12)<br>n=18	3.78 (3.09)<br>3 (0; 11)<br>n=16	2.69 (1.59)<br>2.5 (1; 7.5)<br>n=16			
**GSRS (score 0-45) at week 1**	4.19 (3.64)<br>3.75 (0; 13.5)<br>n=34	4.00 (3.09)<br>4 (0; 13)<br>n=18	4.41 (4.27)<br>3 (0; 13.5)<br>n=16	1.96 (1.71)<br>1.5 (0; 5)<br>n=14			
**GSRS (score 0-45) at week 4**	3.88 (3.61)<br>3 (0; 15)<br>n=32	3.88 (2.85)<br>3.75 (0; 10)<br>n=16	3.88 (4.34)<br>2.5 (0; 15)<br>n=16	3.71 (3.48)<br>3 (0; 12.5)<br>n=14			
**GSRS (score 0-45) at week 8**	3.67 (3.86)<br>2.75 (0; 14.5)<br>n=32	3.66 (3.52)<br>3 (0.5; 13.5)<br>n=16	3.69 (4.28)<br>2.5 (0; 14.5)<br>n=16	2.18 (2.30)<br>1.75 (0; 7.5)<br>n=14			
**GSRS (score 0-45) at week 10**	3.55 (3.50)<br>3 (0; 11.5)<br>n=32	3.78 (3.50)<br>3.25 (0; 11.5)<br>n=16	3.31 (3.59)<br>2 (0; 11.5)<br>n=16	2.21 (2.78)<br>0.5 (0; 7)<br>n=12			
**Change in GSRS (score 0-45) from randomization to week 1**	0.456 (2.706)<br>0.25 (-5; 5.5)<br>n=34	0.306 (2.971)<br>0 (-5; 4.5)<br>n=18	0.625 (2.460)<br>0.5 (-3; 5.5)<br>n=16	-0.821 (1.422)<br>-1 (-2.5; 1.5)<br>n=14	1.28 (-0.25; 2.82)	1.13 (-0.58; 2.87)	1.45 (-0.07; 3.00)
**Change in GSRS (score 0-45) from randomization to week 4**	0.047 (2.525)<br>0 (-5; 4.5)<br>n=32	0.000 (2.751)<br>-0.25 (-5; 4.5)<br>n=16	0.094 (2.368)<br>0 (-4; 4)<br>n=16	0.929 (3.710)<br>-0.25 (-2.5; 11)<br>n=14	-0.882 (-2.667; 1.111)<br>p=0.37	-0.929 (-3.300; 1.500)<br>p=0.48	-0.835 (-3.071; 1.437)<br>p=0.50
**Change in GSRS (score 0-45) from randomization to week 8**	-0.156 (3.298)<br>-0.25 (-5.5; 9)<br>n=32	-0.219 (3.728)<br>-0.25 (-5.5; 9)<br>n=16	-0.094 (2.928)<br>-0.25 (-4; 7.5)<br>n=16	-0.607 (2.816)<br>-1 (-4.5; 6)<br>n=14	0.451 (-1.500; 2.545)<br>p=0.68	0.388 (-2.083; 2.917)<br>p=0.77	0.513 (-1.611; 2.667)<br>p=0.65
**Change in GSRS (score 0-45) from randomization to week 10**	-0.281 (3.175)<br>-0.25 (-6.5; 7)<br>n=32	-0.094 (3.494)<br>0 (-6.5; 7)<br>n=16	-0.469 (2.924)<br>-0.5 (-4.5; 5)<br>n=16	-0.667 (3.003)<br>-1.25 (-4; 5.5)<br>n=12	0.385 (-1.714; 2.571)	0.573 (-2.000; 3.187)	0.198 (-2.143; 2.583)

For continuous variables Mean (SD) / Median (Min; Max) / n= is presented.
For comparison between groups the Fisher´s Non Parametric Permutation Test was used for continuous variables. The confidence interval for then mean difference between groups is based on Fishers non-parametric permutation test.
2020-01-01 Analys.sas

Table 14.2.2.2 Secondary Analysis - Change in GSRS (PP Population)

					Difference and test between groups		
**Variable**	**High+Low dose (n=33)**	**High dose (n=18)**	**Low dose (n=15)**	**Placebo (n=16)**	**High+Low dose vs Placebo**	**High dose vs Placebo**	**Low dose vs Placebo**
**GSRS (score 0-45) at randomization**	3.64 (3.23)<br>3 (0; 12)<br>n=33	3.69 (3.44)<br>2.75 (0; 12)<br>n=18	3.57 (3.07)<br>3 (0; 11)<br>n=15	2.69 (1.59)<br>2.5 (1; 7.5)<br>n=16			
**GSRS (score 0-45) at week 1**	4.08 (3.63)<br>3.5 (0; 13.5)<br>n=33	4.00 (3.09)<br>4 (0; 13)<br>n=18	4.17 (4.31)<br>3 (0; 13.5)<br>n=15	1.96 (1.71)<br>1.5 (0; 5)<br>n=14			
**GSRS (score 0-45) at week 4**	3.90 (3.67)<br>3 (0; 15)<br>n=31	3.88 (2.85)<br>3.75 (0; 10)<br>n=16	3.93 (4.49)<br>2 (0; 15)<br>n=15	3.71 (3.48)<br>3 (0; 12.5)<br>n=14			
**GSRS (score 0-45) at week 8**	3.69 (3.92)<br>2.5 (0; 14.5)<br>n=31	3.66 (3.52)<br>3 (0.5; 13.5)<br>n=16	3.73 (4.43)<br>2.5 (0; 14.5)<br>n=15	2.18 (2.30)<br>1.75 (0; 7.5)<br>n=14			
**GSRS (score 0-45) at week 10**	3.58 (3.55)<br>3 (0; 11.5)<br>n=31	3.78 (3.50)<br>3.25 (0; 11.5)<br>n=16	3.37 (3.71)<br>1.5 (0; 11.5)<br>n=15	2.21 (2.78)<br>0.5 (0; 7)<br>n=12			
**Change in GSRS (score 0-45) from randomization to week 1**	0.439 (2.746)<br>0 (-5; 5.5)<br>n=33	0.306 (2.971)<br>0 (-5; 4.5)<br>n=18	0.600 (2.544)<br>0.5 (-3; 5.5)<br>n=15	-0.821 (1.422)<br>-1 (-2.5; 1.5)<br>n=14	1.26 (-0.28; 2.83)	1.13 (-0.58; 2.87)	1.42 (-0.12; 3.00)
**Change in GSRS (score 0-45) from randomization to week 4**	0.177 (2.455)<br>0 (-5; 4.5)<br>n=31	0.000 (2.751)<br>-0.25 (-5; 4.5)<br>n=16	0.367 (2.175)<br>0 (-2.5; 4)<br>n=15	0.929 (3.710)<br>-0.25 (-2.5; 11)<br>n=14	-0.751 (-2.556; 1.250)<br>p=0.44	-0.929 (-3.300; 1.500)<br>p=0.48	-0.562 (-2.857; 1.687)<br>p=0.66
**Change in GSRS (score 0-45) from randomization to week 8**	-0.032 (3.276)<br>0 (-5.5; 9)<br>n=31	-0.219 (3.728)<br>-0.25 (-5.5; 9)<br>n=16	0.167 (2.833)<br>0 (-4; 7.5)<br>n=15	-0.607 (2.816)<br>-1 (-4.5; 6)<br>n=14	0.575 (-1.400; 2.650)<br>p=0.60	0.388 (-2.083; 2.917)<br>p=0.77	0.774 (-1.400; 2.917)<br>p=0.49
**Change in GSRS (score 0-45) from randomization to week 10**	-0.145 (3.131)<br>0 (-6.5; 7)<br>n=31	-0.094 (3.494)<br>0 (-6.5; 7)<br>n=16	-0.200 (2.815)<br>-0.5 (-4; 5)<br>n=15	-0.667 (3.003)<br>-1.25 (-4; 5.5)<br>n=12	0.522 (-1.556; 2.667)	0.573 (-2.000; 3.187)	0.467 (-1.875; 2.833)

For continuous variables Mean (SD) / Median (Min; Max) / n= is presented.
For comparison between groups the Fisher´s Non Parametric Permutation Test was used for continuous variables. The confidence interval for then mean difference between groups is based on Fishers non-parametric permutation test.
2020-01-01 Analys.sas

Table 14.2.3.1 Secondary Analysis - Change in hematologic and biochemical parameters (ITT Population)

					Difference and test between groups		
**Variable**	**High+Low dose (n=34)**	**High dose (n=18)**	**Low dose (n=16)**	**Placebo (n=16)**	**High+Low dose vs Placebo**	**High dose vs Placebo**	**Low dose vs Placebo**
**Erythrocyte sedimentation rate (mm/hour) at screening**	4.66 (0.45)<br>4.6 (3.7; 5.4)<br>n=34	4.67 (0.47)<br>4.6 (3.7; 5.4)<br>n=18	4.65 (0.45)<br>4.65 (3.8; 5.4)<br>n=16	4.70 (0.57)<br>4.65 (3.9; 5.6)<br>n=16			
**Erythrocyte sedimentation rate (mm/hour) at week 1**	4.65 (0.38)<br>4.65 (4; 5.3)<br>n=34	4.69 (0.39)<br>4.6 (4.2; 5.3)<br>n=18	4.61 (0.39)<br>4.7 (4; 5.3)<br>n=16	4.62 (0.59)<br>4.75 (3.7; 5.5)<br>n=14			
**Erythrocyte sedimentation rate (mm/hour) at week 4**	4.68 (0.39)<br>4.8 (4; 5.3)<br>n=32	4.71 (0.42)<br>4.85 (4.1; 5.3)<br>n=16	4.64 (0.36)<br>4.8 (4; 5.2)<br>n=16	4.61 (0.58)<br>4.7 (3.8; 5.6)<br>n=14			
**Erythrocyte sedimentation rate (mm/hour) at week 8**	4.72 (0.38)<br>4.8 (3.9; 5.4)<br>n=32	4.76 (0.41)<br>4.8 (3.9; 5.4)<br>n=16	4.68 (0.34)<br>4.75 (4; 5.3)<br>n=16	4.66 (0.55)<br>4.8 (3.7; 5.3)<br>n=14			
**Change in Erythrocyte sedimentation rate (mm/hour) from screening to week 1**	-0.009 (0.193)<br>0 (-0.4; 0.6)<br>n=34	0.022 (0.224)<br>0.05 (-0.3; 0.6)<br>n=18	-0.044 (0.150)<br>0 (-0.4; 0.2)<br>n=16	-0.064 (0.231)<br>-0.1 (-0.4; 0.3)<br>n=14	0.055 (-0.073; 0.189)	0.087 (-0.075; 0.256)	0.021 (-0.129; 0.167)
**Change in Erythrocyte sedimentation rate (mm/hour) from screening to week 4**	0.009 (0.212)<br>0 (-0.5; 0.4)<br>n=32	0.025 (0.205)<br>-0.05 (-0.2; 0.4)<br>n=16	-0.006 (0.224)<br>0 (-0.5; 0.4)<br>n=16	-0.079 (0.219)<br>0 (-0.5; 0.2)<br>n=14	0.088 (-0.050; 0.225)<br>p=0.23	0.104 (-0.057; 0.262)<br>p=0.22	0.072 (-0.100; 0.237)<br>p=0.43
**Change in Erythrocyte sedimentation rate (mm/hour) from screening to week 8**	0.056 (0.203)<br>0.1 (-0.4; 0.5)<br>n=32	0.081 (0.201)<br>0.1 (-0.3; 0.5)<br>n=16	0.031 (0.209)<br>0.1 (-0.4; 0.3)<br>n=16	-0.021 (0.249)<br>0 (-0.4; 0.3)<br>n=14	0.078 (-0.064; 0.212)<br>p=0.30	0.103 (-0.067; 0.271)<br>p=0.25	0.053 (-0.125; 0.225)<br>p=0.59
**C-reactive protein (mg/L) at screening**	0.912 (3.630)<br>0 (0; 21)<br>n=34	1.50 (4.94)<br>0 (0; 21)<br>n=18	0.250 (0.683)<br>0 (0; 2)<br>n=16	0.938 (2.265)<br>0 (0; 9)<br>n=16			
**C-reactive protein (mg/L) at week 1**	0.500 (1.420)<br>0 (0; 7)<br>n=34	0.833 (1.855)<br>0 (0; 7)<br>n=18	0.125 (0.500)<br>0 (0; 2)<br>n=16	1.57 (2.59)<br>0 (0; 8)<br>n=14			
**C-reactive protein (mg/L) at week 4**	1.19 (3.34)<br>0 (0; 18)<br>n=32	2.13 (4.56)<br>0 (0; 18)<br>n=16	0.250 (0.577)<br>0 (0; 2)<br>n=16	0.857 (1.916)<br>0 (0; 7)<br>n=14			
**C-reactive protein (mg/L) at week 8**	0.719 (1.464)<br>0 (0; 6)<br>n=32	1.38 (1.86)<br>0.5 (0; 6)<br>n=16	0.063 (0.250)<br>0 (0; 1)<br>n=16	0.857 (1.406)<br>0 (0; 4)<br>n=14			

					Difference and test between groups		
Variable	High+Low dose (n=34)	High dose (n=18)	Low dose (n=16)	Placebo (n=16)	High+Low dose vs Placebo	High dose vs Placebo	Low dose vs Placebo
**Change in C-reactive protein (mg/L) from screening to week 1**	-0.412 (2.451)<br>0 (-14; 1)<br>n=34	-0.667 (3.361)<br>0 (-14; 1)<br>n=18	-0.125 (0.500)<br>0 (-2; 0)<br>n=16	0.643 (1.550)<br>0 (-1; 5)<br>n=14	-1.05 (-2.56; -0.08)	-1.31 (-3.17; 0.17)	-0.768 (-1.600; -0.000)
**Change in C-reactive protein (mg/L) from screening to week 4**	0.250 (4.280)<br>0 (-15; 18)<br>n=32	0.500 (6.088)<br>0 (-15; 18)<br>n=16	0.000 (0.816)<br>0 (-2; 2)<br>n=16	-0.071 (0.917)<br>0 (-2; 2)<br>n=14	0.321 (-1.778; 2.571)<br>p=0.68	0.571 (-2.333; 3.667)<br>p=0.59	0.071 (-0.571; 0.714)<br>p=1.00
**Change in C-reactive protein (mg/L) from screening to week 8**	-0.219 (2.871)<br>0 (-15; 3)<br>n=32	-0.250 (4.058)<br>0 (-15; 3)<br>n=16	-0.188 (0.750)<br>0 (-2; 1)<br>n=16	-0.071 (2.235)<br>0 (-6; 4)<br>n=14	-0.147 (-2.000; 1.222)<br>p=1.00	-0.179 (-2.600; 1.875)<br>p=0.99	-0.116 (-1.286; 1.000)<br>p=0.94
**Red blood cell count (10^9/L) at screening**	241.0 (44.1)<br>242 (172; 331)<br>n=34	253.6 (49.9)<br>261 (173; 331)<br>n=18	226.9 (32.5)<br>229 (172; 295)<br>n=16	238.1 (41.8)<br>235 (175; 324)<br>n=16			
**Red blood cell count (10^9/L) at week 1**	249.0 (39.4)<br>246 (153; 346)<br>n=34	261.9 (40.7)<br>259.5 (204; 346)<br>n=18	234.5 (33.4)<br>241.5 (153; 297)<br>n=16	247.9 (32.5)<br>242 (198; 290)<br>n=14			
**Red blood cell count (10^9/L) at week 4**	255.5 (48.1)<br>250 (175; 385)<br>n=32	272.3 (49.2)<br>265 (198; 385)<br>n=16	238.8 (41.9)<br>236.5 (175; 316)<br>n=16	250.5 (33.7)<br>252 (194; 302)<br>n=14			
**Red blood cell count (10^9/L) at week 8**	249.2 (45.1)<br>249.5 (169; 340)<br>n=32	264.2 (43.1)<br>261.5 (194; 340)<br>n=16	234.2 (43.1)<br>232.5 (169; 310)<br>n=16	243.6 (34.5)<br>231.5 (201; 319)<br>n=14			
**Change in Red blood cell count (10^9/L) from screening to week 1**	8.03 (27.09)<br>8 (-48; 57)<br>n=34	8.39 (32.27)<br>10.5 (-48; 57)<br>n=18	7.63 (20.82)<br>7 (-24; 55)<br>n=16	10.1 (25.7)<br>20.5 (-38; 44)<br>n=14	-2.04 (-19.15; 14.67)	-1.68 (-23.20; 19.87)	-2.45 (-20.00; 15.00)
**Change in Red blood cell count (10^9/L) from screening to week 4**	19.5 (31.3)<br>11.5 (-20; 100)<br>n=32	27.1 (37.5)<br>23.5 (-20; 100)<br>n=16	11.9 (22.3)<br>8 (-14; 69)<br>n=16	12.6 (25.5)<br>9.5 (-36; 71)<br>n=14	6.83 (-11.42; 26.50)<br>p=0.50	14.4 (-9.4; 38.6)<br>p=0.24	-0.768 (-18.667; 17.167)<br>p=0.93
**Change in Red blood cell count (10^9/L) from screening to week 8**	13.1 (25.8)<br>7 (-41; 66)<br>n=32	18.9 (29.0)<br>16 (-41; 66)<br>n=16	7.31 (21.56)<br>0 (-22; 46)<br>n=16	5.79 (39.50)<br>15 (-90; 88)<br>n=14	7.34 (-12.87; 26.92)<br>p=0.46	13.2 (-12.5; 38.6)<br>p=0.32	1.53 (-22.00; 24.67)<br>p=0.90
**White blood cell count (10^9/L) at screening**	4.73 (1.16)<br>4.5 (2.9; 7.1)<br>n=34	4.83 (1.28)<br>4.65 (2.9; 7.1)<br>n=18	4.61 (1.03)<br>4.15 (3.6; 6.8)<br>n=16	4.91 (0.87)<br>5.05 (3.6; 6.7)<br>n=16			
**White blood cell count (10^9/L) at week 1**	5.18 (1.38)<br>4.85 (3.4; 10)<br>n=34	5.49 (1.67)<br>5.25 (3.4; 10)<br>n=18	4.84 (0.90)<br>4.6 (3.7; 7)<br>n=16	5.06 (1.04)<br>4.85 (4; 7.1)<br>n=14			

					Difference and test between groups		
**Variable**	**High+Low dose (n=34)**	**High dose (n=18)**	**Low dose (n=16)**	**Placebo (n=16)**	**High+Low dose vs Placebo**	**High dose vs Placebo**	**Low dose vs Placebo**
**White blood cell count (10^9/L) at week 4**	5.28 (1.27)<br>5.05 (3.5; 7.9)<br>n=32	5.37 (1.35)<br>5.15 (3.6; 7.9)<br>n=16	5.20 (1.23)<br>4.9 (3.5; 7.1)<br>n=16	5.45 (1.06)<br>5.3 (3.8; 7.9)<br>n=14			
**White blood cell count (10^9/L) at week 8**	5.37 (1.62)<br>5.15 (3.1; 8.8)<br>n=32	5.73 (1.87)<br>5.75 (3.1; 8.8)<br>n=16	5.01 (1.28)<br>4.8 (3.3; 7.4)<br>n=16	5.44 (1.42)<br>4.85 (3.6; 8.2)<br>n=14			
**Change in White blood cell count (10^9/L) from screening to week 1**	0.456 (0.882)<br>0.3 (-1; 3.6)<br>n=34	0.656 (1.031)<br>0.45 (-0.5; 3.6)<br>n=18	0.231 (0.637)<br>0.3 (-1; 1.5)<br>n=16	0.121 (1.090)<br>0.25 (-1.6; 2.2)<br>n=14	0.334 (-0.256; 0.950)	0.534 (-0.217; 1.314)	0.110 (-0.550; 0.771)
**Change in White blood cell count (10^9/L) from screening to week 4**	0.609 (0.798)<br>0.45 (-0.5; 3)<br>n=32	0.625 (0.770)<br>0.45 (-0.5; 2.2)<br>n=16	0.594 (0.850)<br>0.45 (-0.5; 3)<br>n=16	0.514 (1.156)<br>0.4 (-1.4; 2.8)<br>n=14	0.095 (-0.475; 0.700)<br>p=0.76	0.111 (-0.617; 0.833)<br>p=0.78	0.079 (-0.662; 0.837)<br>p=0.85
**Change in White blood cell count (10^9/L) from screening to week 8**	0.694 (0.998)<br>0.6 (-1.1; 3)<br>n=32	0.988 (1.110)<br>0.8 (-0.8; 3)<br>n=16	0.400 (0.802)<br>0.4 (-1.1; 1.8)<br>n=16	0.500 (1.173)<br>0.1 (-0.9; 2.8)<br>n=14	0.194 (-0.487; 0.891)<br>p=0.59	0.487 (-0.360; 1.350)<br>p=0.26	-0.100 (-0.850; 0.650)<br>p=0.80
**Platelet count (10^9/L) at screening**	6.03 (6.74)<br>3 (1; 29)<br>n=34	7.33 (8.35)<br>3 (1; 29)<br>n=18	4.56 (4.10)<br>3 (1; 15)<br>n=16	6.69 (4.29)<br>5 (2; 16)<br>n=16			
**Platelet count (10^9/L) at week 1**	5.32 (4.82)<br>3 (1; 18)<br>n=34	6.50 (5.99)<br>3.5 (1; 18)<br>n=18	4.00 (2.63)<br>3 (1; 10)<br>n=16	6.21 (3.66)<br>5.5 (1; 13)<br>n=14			
**Platelet count (10^9/L) at week 4**	6.19 (5.87)<br>4 (1; 23)<br>n=32	7.38 (6.95)<br>4.5 (1; 23)<br>n=16	5.00 (4.47)<br>3 (1; 18)<br>n=16	5.50 (3.18)<br>4 (2; 12)<br>n=14			
**Platelet count (10^9/L) at week 8**	4.94 (4.79)<br>3.5 (1; 22)<br>n=32	6.19 (6.08)<br>4 (1; 22)<br>n=16	3.69 (2.68)<br>3 (1; 10)<br>n=16	6.07 (3.85)<br>5 (2; 15)<br>n=14			
**Change in Platelet count (10^9/L) from screening to week 1**	-0.706 (2.553)<br>0 (-11; 3)<br>n=34	-0.833 (2.995)<br>0 (-11; 3)<br>n=18	-0.563 (2.032)<br>0 (-5; 2)<br>n=16	-0.714 (2.972)<br>-0.5 (-5; 4)<br>n=14	0.008 (-1.800; 1.600)	-0.119 (-2.333; 2.000)	0.152 (-1.750; 2.000)
**Change in Platelet count (10^9/L) from screening to week 4**	1.000 (4.853)<br>0 (-6; 20)<br>n=32	1.56 (5.81)<br>0 (-6; 20)<br>n=16	0.438 (3.777)<br>0 (-6; 11)<br>n=16	-1.43 (2.24)<br>-1 (-7; 1)<br>n=14	2.43 (0.00; 5.25)<br>p=0.053	2.99 (0.00; 6.33)<br>p=0.059	1.87 (-0.43; 4.25)<br>p=0.13
**Change in Platelet count (10^9/L) from screening to week 8**	-0.250 (2.185)<br>0 (-7; 3)<br>n=32	0.375 (1.455)<br>0 (-3; 3)<br>n=16	-0.875 (2.630)<br>0 (-7; 3)<br>n=16	-0.857 (3.655)<br>-0.5 (-7; 5)<br>n=14	0.607 (-1.200; 2.375)<br>p=0.52	1.23 (-0.83; 3.29)<br>p=0.26	-0.018 (-2.429; 2.333)<br>p=1.00

					Difference and test between groups		
Variable	High+Low dose (n=34)	High dose (n=18)	Low dose (n=16)	Placebo (n=16)	High+Low dose vs Placebo	High dose vs Placebo	Low dose vs Placebo
**Alanine transaminase (µkat/L) at screening**	0.352 (0.151)<br>0.315 (0.13; 0.77)<br>n=34	0.407 (0.175)<br>0.345 (0.21; 0.77)<br>n=18	0.290 (0.090)<br>0.3 (0.13; 0.47)<br>n=16	0.418 (0.202)<br>0.35 (0.19; 0.83)<br>n=16			
**Alanine transaminase (µkat/L) at week 1**	0.343 (0.162)<br>0.335 (0; 0.97)<br>n=34	0.392 (0.181)<br>0.335 (0.2; 0.97)<br>n=18	0.287 (0.119)<br>0.335 (0; 0.44)<br>n=16	0.509 (0.453)<br>0.335 (0.19; 1.8)<br>n=14			
**Alanine transaminase (µkat/L) at week 4**	0.330 (0.140)<br>0.32 (0.12; 0.84)<br>n=32	0.382 (0.166)<br>0.33 (0.2; 0.84)<br>n=16	0.278 (0.086)<br>0.265 (0.12; 0.41)<br>n=16	0.364 (0.224)<br>0.285 (0.23; 1.1)<br>n=14			
**Alanine transaminase (µkat/L) at week 8**	0.360 (0.184)<br>0.34 (0.12; 1.1)<br>n=32	0.427 (0.225)<br>0.395 (0.18; 1.1)<br>n=16	0.294 (0.098)<br>0.265 (0.12; 0.44)<br>n=16	0.366 (0.281)<br>0.295 (0.17; 1.3)<br>n=14			
**Change in Alanine transaminase (µkat/L) from screening to week 1**	-0.009 (0.098)<br>0.005 (-0.2; 0.2)<br>n=34	-0.015 (0.118)<br>0.005 (-0.2; 0.2)<br>n=18	-0.003 (0.072)<br>-0.005 (-0.13; 0.15)<br>n=16	0.114 (0.416)<br>0.01 (-0.38; 1.42)<br>n=14	-0.123 (-0.256; 0.037)	-0.129 (-0.324; 0.063)	-0.117 (-0.315; 0.069)
**Change in Alanine transaminase (µkat/L) from screening to week 4**	-0.026 (0.084)<br>-0.01 (-0.28; 0.11)<br>n=32	-0.039 (0.110)<br>-0.015 (-0.28; 0.11)<br>n=16	-0.013 (0.046)<br>-0.01 (-0.15; 0.05)<br>n=16	-0.031 (0.148)<br>-0.025 (-0.39; 0.31)<br>n=14	0.006 (-0.065; 0.076)<br>p=0.87	-0.007 (-0.104; 0.090)<br>p=0.89	0.019 (-0.058; 0.097)<br>p=0.65
**Change in Alanine transaminase (µkat/L) from screening to week 8**	0.005 (0.102)<br>-0.01 (-0.23; 0.33)<br>n=32	0.006 (0.137)<br>-0.01 (-0.23; 0.33)<br>n=16	0.004 (0.054)<br>-0.015 (-0.07; 0.12)<br>n=16	-0.029 (0.203)<br>-0.025 (-0.44; 0.51)<br>n=14	0.034 (-0.057; 0.129)<br>p=0.46	0.036 (-0.092; 0.162)<br>p=0.60	0.033 (-0.071; 0.137)<br>p=0.57
**Aspartate transaminase (µkat/L) at screening**	0.417 (0.157)<br>0.37 (0.28; 1.2)<br>n=34	0.429 (0.207)<br>0.355 (0.28; 1.2)<br>n=18	0.403 (0.073)<br>0.38 (0.29; 0.55)<br>n=16	0.472 (0.123)<br>0.425 (0.26; 0.69)<br>n=16			
**Aspartate transaminase (µkat/L) at week 1**	0.391 (0.103)<br>0.385 (0.23; 0.74)<br>n=34	0.401 (0.116)<br>0.38 (0.23; 0.74)<br>n=18	0.380 (0.088)<br>0.395 (0.26; 0.54)<br>n=16	0.827 (1.579)<br>0.385 (0.23; 6.3)<br>n=14			
**Aspartate transaminase (µkat/L) at week 4**	0.388 (0.082)<br>0.37 (0.25; 0.65)<br>n=32	0.389 (0.070)<br>0.38 (0.25; 0.53)<br>n=16	0.388 (0.095)<br>0.355 (0.29; 0.65)<br>n=16	0.401 (0.103)<br>0.36 (0.28; 0.61)<br>n=14			
**Aspartate transaminase (µkat/L) at week 8**	0.395 (0.087)<br>0.405 (0.19; 0.58)<br>n=32	0.416 (0.080)<br>0.435 (0.29; 0.58)<br>n=16	0.374 (0.092)<br>0.36 (0.19; 0.55)<br>n=16	0.409 (0.103)<br>0.395 (0.25; 0.69)<br>n=14			
**Change in Aspartate transaminase (µkat/L) from screening to week 1**	-0.026 (0.098)<br>0 (-0.46; 0.1)<br>n=34	-0.029 (0.129)<br>0.02 (-0.46; 0.1)<br>n=18	-0.023 (0.047)<br>-0.01 (-0.11; 0.03)<br>n=16	0.376 (1.592)<br>-0.03 (-0.22; 5.9)<br>n=14	-0.402 (-0.779; 0.064)	-0.405 (-1.013; 0.084)	-0.399 (-1.025; 0.067)

					Difference and test between groups		
**Variable**	**High+Low dose (n=34)**	**High dose (n=18)**	**Low dose (n=16)**	**Placebo (n=16)**	**High+Low dose vs Placebo**	**High dose vs Placebo**	**Low dose vs Placebo**
**Change in Aspartate transaminase (µkat/L) from screening to week 4**	-0.034 (0.158)<br>-0.015 (-0.8; 0.17)<br>n=32	-0.053 (0.215)<br>-0.015 (-0.8; 0.17)<br>n=16	-0.016 (0.068)<br>-0.01 (-0.12; 0.13)<br>n=16	-0.051 (0.083)<br>-0.055 (-0.21; 0.11)<br>n=14	0.017 (-0.078; 0.087)<br>p=0.66	-0.002 (-0.120; 0.101)<br>p=0.98	0.035 (-0.021; 0.092)<br>p=0.22
**Change in Aspartate transaminase (µkat/L) from screening to week 8**	-0.028 (0.149)<br>-0.005 (-0.73; 0.12)<br>n=32	-0.026 (0.202)<br>0.02 (-0.73; 0.12)<br>n=16	-0.029 (0.070)<br>-0.03 (-0.18; 0.07)<br>n=16	-0.042 (0.107)<br>-0.025 (-0.22; 0.12)<br>n=14	0.015 (-0.081; 0.090)<br>p=0.69	0.016 (-0.103; 0.123)<br>p=0.83	0.013 (-0.053; 0.080)<br>p=0.69
**Alkaline phosphatase (µkat/L) at screening**	0.987 (0.228)<br>0.94 (0.65; 1.6)<br>n=34	0.923 (0.150)<br>0.915 (0.68; 1.3)<br>n=18	1.06 (0.28)<br>0.99 (0.65; 1.6)<br>n=16	1.15 (0.38)<br>1.1 (0.44; 1.6)<br>n=16			
**Alkaline phosphatase (µkat/L) at week 1**	0.995 (0.252)<br>0.92 (0.68; 1.5)<br>n=34	0.922 (0.181)<br>0.87 (0.7; 1.4)<br>n=18	1.08 (0.30)<br>1.03 (0.68; 1.5)<br>n=16	1.15 (0.38)<br>1.15 (0.52; 1.8)<br>n=14			
**Alkaline phosphatase (µkat/L) at week 4**	1.00 (0.24)<br>0.92 (0.74; 1.5)<br>n=32	0.930 (0.190)<br>0.86 (0.74; 1.5)<br>n=16	1.08 (0.27)<br>0.97 (0.78; 1.5)<br>n=16	1.12 (0.34)<br>1.2 (0.44; 1.6)<br>n=14			
**Alkaline phosphatase (µkat/L) at week 8**	1.01 (0.25)<br>0.97 (0.69; 1.6)<br>n=32	0.944 (0.171)<br>0.94 (0.73; 1.4)<br>n=16	1.07 (0.30)<br>1.04 (0.69; 1.6)<br>n=16	1.11 (0.33)<br>1.1 (0.58; 1.6)<br>n=14			
**Change in Alkaline phosphatase (µkat/L) from screening to week 1**	0.008 (0.074)<br>0 (-0.1; 0.2)<br>n=34	-0.002 (0.068)<br>0 (-0.1; 0.1)<br>n=18	0.018 (0.081)<br>0 (-0.1; 0.2)<br>n=16	-0.011 (0.139)<br>0 (-0.4; 0.2)<br>n=14	0.018 (-0.046; 0.079)	0.009 (-0.066; 0.085)	0.029 (-0.053; 0.112)
**Change in Alkaline phosphatase (µkat/L) from screening to week 4**	0.015 (0.113)<br>0.005 (-0.3; 0.2)<br>n=32	0.013 (0.107)<br>0.045 (-0.2; 0.2)<br>n=16	0.016 (0.122)<br>0 (-0.3; 0.2)<br>n=16	-0.047 (0.159)<br>0 (-0.4; 0.28)<br>n=14	0.062 (-0.021; 0.143)<br>p=0.14	0.060 (-0.040; 0.161)<br>p=0.24	0.063 (-0.041; 0.167)<br>p=0.23
**Change in Alkaline phosphatase (µkat/L) from screening to week 8**	0.021 (0.109)<br>0.015 (-0.22; 0.21)<br>n=32	0.027 (0.089)<br>0.03 (-0.22; 0.2)<br>n=16	0.014 (0.130)<br>0 (-0.2; 0.21)<br>n=16	-0.056 (0.147)<br>-0.01 (-0.4; 0.14)<br>n=14	0.077 (-0.002; 0.155)<br>p=0.060	0.083 (-0.006; 0.174)<br>p=0.069	0.071 (-0.034; 0.176)<br>p=0.18
**Bilirubin (µmol/L) at screening**	11.4 (8.0)<br>9 (3.8; 37)<br>n=34	10.4 (7.7)<br>8.1 (3.8; 37)<br>n=18	12.5 (8.5)<br>9.5 (5.3; 37)<br>n=16	10.5 (5.0)<br>9.5 (3.7; 23)<br>n=16			
**Bilirubin (µmol/L) at week 1**	9.93 (5.15)<br>7.95 (4; 25)<br>n=34	9.87 (5.50)<br>7.5 (4; 25)<br>n=18	10.00 (4.90)<br>8.5 (4; 19)<br>n=16	9.59 (4.06)<br>9.35 (4.5; 18)<br>n=14			
**Bilirubin (µmol/L) at week 4**	10.8 (5.9)<br>9.3 (3.2; 30)<br>n=32	9.13 (3.52)<br>8.45 (3.2; 15)<br>n=16	12.5 (7.3)<br>9.6 (3.4; 30)<br>n=16	9.21 (4.32)<br>9 (0; 17)<br>n=14			

Variable	High+Low dose (n=34)	High dose (n=18)	Low dose (n=16)	Placebo (n=16)	Difference and test between groups<br>High+Low dose vs Placebo	High dose vs Placebo	Low dose vs Placebo
**Bilirubin (µmol/L) at week 8**	10.5 (5.2)<br>9.4 (0; 23)<br>n=32	9.73 (5.10)<br>9.65 (0; 23)<br>n=16	11.3 (5.4)<br>8.9 (3.3; 20)<br>n=16	9.64 (4.35)<br>7.8 (5.7; 19)<br>n=14			
**Change in Bilirubin (µmol/L) from screening to week 1**	-1.44 (4.94)<br>-0.7 (-22; 5.4)<br>n=34	-0.528 (3.615)<br>-0.45 (-12; 5.4)<br>n=18	-2.48 (6.06)<br>-1.65 (-22; 5)<br>n=16	-0.286 (4.141)<br>-0.5 (-9.9; 8.4)<br>n=14	-1.16 (-4.32; 1.57)	-0.242 (-3.040; 2.489)	-2.19 (-6.10; 1.48)
**Change in Bilirubin (µmol/L) from screening to week 4**	-0.925 (5.923)<br>0 (-24; 11)<br>n=32	-1.84 (6.49)<br>-0.15 (-24; 4)<br>n=16	-0.006 (5.351)<br>0.1 (-12; 11)<br>n=16	-0.657 (3.922)<br>-1.35 (-5.8; 9.3)<br>n=14	-0.268 (-3.910; 2.933)<br>p=0.92	-1.19 (-5.22; 2.49)<br>p=0.63	0.651 (-2.925; 4.114)<br>p=0.72
**Change in Bilirubin (µmol/L) from screening to week 8**	-1.20 (5.15)<br>0 (-19; 5.9)<br>n=32	-1.25 (5.18)<br>0.4 (-14; 5.9)<br>n=16	-1.16 (5.30)<br>0 (-19; 5)<br>n=16	-0.236 (5.372)<br>-1 (-7.8; 11.3)<br>n=14	-0.967 (-4.470; 2.262)<br>p=0.57	-1.01 (-5.08; 2.90)<br>p=0.61	-0.921 (-4.983; 2.920)<br>p=0.67
**Renal function eGFR (ml/min) at screening**	90.6 (10.0)<br>91 (71; 115)<br>n=34	90.1 (8.8)<br>91 (71; 104)<br>n=18	91.3 (11.4)<br>90 (71; 115)<br>n=16	89.6 (9.3)<br>87.5 (76; 117)<br>n=16			
**Renal function eGFR (ml/min) at week 1**	91.3 (10.3)<br>90 (76; 117)<br>n=34	90.4 (10.7)<br>88 (79; 117)<br>n=18	92.4 (10.0)<br>91 (76; 111)<br>n=16	91.0 (9.1)<br>92 (75; 109)<br>n=14			
**Renal function eGFR (ml/min) at week 4**	91.1 (9.4)<br>88.5 (76; 111)<br>n=32	89.1 (9.0)<br>87.5 (76; 111)<br>n=16	93.0 (9.6)<br>89.5 (80; 107)<br>n=16	93.0 (9.5)<br>95.5 (72; 108)<br>n=14			
**Renal function eGFR (ml/min) at week 8**	90.7 (8.7)<br>90.5 (73; 110)<br>n=32	90.5 (9.4)<br>89.5 (75; 110)<br>n=16	90.8 (8.3)<br>91 (73; 101)<br>n=16	92.6 (8.5)<br>92 (76; 112)<br>n=14			
**Change in Renal function eGFR (ml/min) from screening to week 1**	0.676 (5.819)<br>0.5 (-10; 13)<br>n=34	0.278 (7.028)<br>-2 (-10; 13)<br>n=18	1.13 (4.26)<br>1.5 (-6; 7)<br>n=16	0.214 (4.726)<br>0 (-8; 7)<br>n=14	0.462 (-3.000; 4.000)	0.063 (-4.333; 4.625)	0.911 (-2.500; 4.286)
**Change in Renal function eGFR (ml/min) from screening to week 4**	0.688 (6.332)<br>0.5 (-10; 14)<br>n=32	-0.375 (6.270)<br>0 (-10; 11)<br>n=16	1.75 (6.41)<br>2 (-8; 14)<br>n=16	2.21 (6.99)<br>4.5 (-12; 12)<br>n=14	-1.53 (-5.80; 2.70)<br>p=0.49	-2.59 (-7.50; 2.33)<br>p=0.30	-0.464 (-5.500; 4.500)<br>p=0.88
**Change in Renal function eGFR (ml/min) from screening to week 8**	0.281 (5.687)<br>0.5 (-14; 13)<br>n=32	1.000 (5.610)<br>1 (-9; 13)<br>n=16	-0.438 (5.853)<br>-1 (-14; 9)<br>n=16	1.86 (5.32)<br>2 (-8; 10)<br>n=14	-1.58 (-5.22; 2.00)<br>p=0.40	-0.857 (-4.875; 3.250)<br>p=0.69	-2.29 (-6.50; 1.87)<br>p=0.29
**Blood total protein (g/l) at screening**	70.5 (4.6)<br>70 (62; 80)<br>n=33	70.9 (4.9)<br>72.5 (62; 80)<br>n=18	70.1 (4.2)<br>70 (64; 78)<br>n=15	72.9 (5.1)<br>73 (62; 81)<br>n=16			

Variable	High+Low dose (n=34)	High dose (n=18)	Low dose (n=16)	Placebo (n=16)	Difference and test between groups: High+Low dose vs Placebo	High dose vs Placebo	Low dose vs Placebo
**Blood total protein (g/l) at week 1**	71.3 (3.5)<br>72 (65; 78)<br>n=34	71.6 (3.9)<br>72 (65; 78)<br>n=18	70.9 (3.1)<br>71 (66; 76)<br>n=16	71.7 (2.6)<br>72.5 (65; 75)<br>n=14			
**Blood total protein (g/l) at week 4**	71.4 (4.0)<br>71 (62; 81)<br>n=32	71.9 (4.9)<br>71 (62; 81)<br>n=16	70.9 (2.8)<br>71 (66; 75)<br>n=16	71.6 (3.9)<br>72.5 (60; 76)<br>n=14			
**Blood total protein (g/l) at week 8**	71.0 (3.6)<br>71 (64; 79)<br>n=32	72.3 (4.1)<br>72.5 (64; 79)<br>n=16	69.8 (2.6)<br>70 (65; 75)<br>n=16	71.9 (3.0)<br>72.5 (65; 75)<br>n=14			
**Change in Blood total protein (g/l) from screening to week 1**	0.636 (3.707)<br>1 (-7; 7)<br>n=33	0.722 (4.084)<br>0.5 (-7; 7)<br>n=18	0.533 (3.335)<br>1 (-6; 5)<br>n=15	-1.14 (3.76)<br>-1 (-7; 3)<br>n=14	1.78 (-0.60; 4.12)	1.87 (-1.00; 4.75)	1.68 (-1.00; 4.40)
**Change in Blood total protein (g/l) from screening to week 4**	1.000 (4.457)<br>1 (-8; 13)<br>n=31	1.38 (5.28)<br>1 (-8; 13)<br>n=16	0.600 (3.521)<br>0 (-4; 8)<br>n=15	-1.21 (5.55)<br>-1 (-10; 6)<br>n=14	2.21 (-0.92; 5.33)<br>p=0.17	2.59 (-1.43; 6.67)<br>p=0.22	1.81 (-1.67; 5.33)<br>p=0.31
**Change in Blood total protein (g/l) from screening to week 8**	0.742 (3.473)<br>1 (-9; 6)<br>n=31	1.81 (2.93)<br>2 (-3; 6)<br>n=16	-0.400 (3.738)<br>-1 (-9; 5)<br>n=15	-1.000 (4.574)<br>-0.5 (-8; 4)<br>n=14	1.74 (-0.86; 4.22)<br>p=0.19	2.81 (0.00; 5.71)<br>p=0.061	0.600 (-2.571; 3.833)<br>p=0.72
**Blood haemoglobin (g/l) at screening**	140.2 (13.2)<br>139.5 (118; 164)<br>n=34	140.0 (13.9)<br>139.5 (118; 164)<br>n=18	140.4 (13.0)<br>141 (124; 159)<br>n=16	141.8 (14.6)<br>140 (123; 169)<br>n=16			
**Blood haemoglobin (g/l) at week 1**	139.9 (12.2)<br>140.5 (119; 165)<br>n=34	140.4 (12.5)<br>142 (120; 162)<br>n=18	139.4 (12.1)<br>138.5 (119; 165)<br>n=16	138.1 (12.5)<br>139.5 (118; 161)<br>n=14			
**Blood haemoglobin (g/l) at week 4**	140.0 (12.1)<br>141.5 (117; 162)<br>n=32	139.8 (13.5)<br>144 (117; 160)<br>n=16	140.1 (10.9)<br>140.5 (124; 162)<br>n=16	138.9 (14.2)<br>137.5 (118; 171)<br>n=14			
**Blood haemoglobin (g/l) at week 8**	141.1 (11.1)<br>142.5 (120; 161)<br>n=32	141.8 (12.6)<br>144.5 (120; 161)<br>n=16	140.4 (9.7)<br>141.5 (127; 161)<br>n=16	138.7 (12.3)<br>141.5 (117; 161)<br>n=14			
**Change in Blood haemoglobin (g/l) from screening to week 1**	-0.265 (5.822)<br>-0.5 (-12; 10)<br>n=34	0.444 (6.157)<br>1.5 (-12; 10)<br>n=18	-1.06 (5.51)<br>-1.5 (-11; 7)<br>n=16	-2.21 (6.07)<br>-3 (-12; 9)<br>n=14	1.95 (-1.87; 5.67)	2.66 (-1.78; 7.14)	1.15 (-3.25; 5.43)
**Change in Blood haemoglobin (g/l) from screening to week 4**	-0.125 (5.988)<br>-1 (-13; 14)<br>n=32	0.063 (6.060)<br>-1.5 (-8; 12)<br>n=16	-0.313 (6.107)<br>-0.5 (-13; 14)<br>n=16	-1.43 (5.33)<br>-0.5 (-12; 6)<br>n=14	1.30 (-2.44; 5.00)<br>p=0.50	1.49 (-2.80; 5.71)<br>p=0.51	1.12 (-3.17; 5.43)<br>p=0.63

					Difference and test between groups		
**Variable**	**High+Low dose (n=34)**	**High dose (n=18)**	**Low dose (n=16)**	**Placebo (n=16)**	**High+Low dose vs Placebo**	**High dose vs Placebo**	**Low dose vs Placebo**
**Change in Blood haemoglobin (g/l) from screening to week 8**	0.969 (6.498)<br>1.5 (-14; 17)<br>n=32	2.00 (6.35)<br>0 (-10; 17)<br>n=16	-0.063 (6.688)<br>2.5 (-14; 7)<br>n=16	-1.57 (7.08)<br>-0.5 (-16; 8)<br>n=14	2.54 (-1.80; 6.78)<br>p=0.25	3.57 (-1.43; 8.62)<br>p=0.16	1.51 (-3.78; 6.67)<br>p=0.57

For continuous variables Mean (SD) / Median (Min; Max) / n= is presented.
For comparison between groups the Fisher´s Non Parametric Permutation Test was used for continuous variables. The confidence interval for then mean difference between groups is based on Fishers non-parametric permutation test.
2020-01-01 Analys.sas

Table 14.2.3.2 Secondary Analysis - Change in hematologic and biochemical parameters (PP Population)

					Difference and test between groups		
**Variable**	**High+Low dose (n=33)**	**High dose (n=18)**	**Low dose (n=15)**	**Placebo (n=16)**	**High+Low dose vs Placebo**	**High dose vs Placebo**	**Low dose vs Placebo**
**Erythrocyte sedimentation rate (mm/hour) at screening**	4.65 (0.46)<br>4.6 (3.7; 5.4)<br>n=33	4.67 (0.47)<br>4.6 (3.7; 5.4)<br>n=18	4.63 (0.46)<br>4.6 (3.8; 5.4)<br>n=15	4.70 (0.57)<br>4.65 (3.9; 5.6)<br>n=16			
**Erythrocyte sedimentation rate (mm/hour) at week 1**	4.65 (0.39)<br>4.6 (4; 5.3)<br>n=33	4.69 (0.39)<br>4.6 (4.2; 5.3)<br>n=18	4.60 (0.40)<br>4.7 (4; 5.3)<br>n=15	4.62 (0.59)<br>4.75 (3.7; 5.5)<br>n=14			
**Erythrocyte sedimentation rate (mm/hour) at week 4**	4.67 (0.39)<br>4.8 (4; 5.3)<br>n=31	4.71 (0.42)<br>4.85 (4.1; 5.3)<br>n=16	4.63 (0.37)<br>4.8 (4; 5.2)<br>n=15	4.61 (0.58)<br>4.7 (3.8; 5.6)<br>n=14			
**Erythrocyte sedimentation rate (mm/hour) at week 8**	4.72 (0.38)<br>4.8 (3.9; 5.4)<br>n=31	4.76 (0.41)<br>4.8 (3.9; 5.4)<br>n=16	4.67 (0.35)<br>4.7 (4; 5.3)<br>n=15	4.66 (0.55)<br>4.8 (3.7; 5.3)<br>n=14			
**Change in Erythrocyte sedimentation rate (mm/hour) from screening to week 1**	-0.003 (0.193)<br>0 (-0.4; 0.6)<br>n=33	0.022 (0.224)<br>0.05 (-0.3; 0.6)<br>n=18	-0.033 (0.150)<br>0 (-0.4; 0.2)<br>n=15	-0.064 (0.231)<br>-0.1 (-0.4; 0.3)<br>n=14	0.061 (-0.070; 0.200)	0.087 (-0.075; 0.256)	0.031 (-0.114; 0.180)
**Change in Erythrocyte sedimentation rate (mm/hour) from screening to week 4**	0.013 (0.214)<br>0 (-0.5; 0.4)<br>n=31	0.025 (0.205)<br>-0.05 (-0.2; 0.4)<br>n=16	0.000 (0.230)<br>0 (-0.5; 0.4)<br>n=15	-0.079 (0.219)<br>0 (-0.5; 0.2)<br>n=14	0.091 (-0.050; 0.233)<br>p=0.22	0.104 (-0.057; 0.262)<br>p=0.22	0.079 (-0.100; 0.250)<br>p=0.40
**Change in Erythrocyte sedimentation rate (mm/hour) from screening to week 8**	0.058 (0.206)<br>0.1 (-0.4; 0.5)<br>n=31	0.081 (0.201)<br>0.1 (-0.3; 0.5)<br>n=16	0.033 (0.216)<br>0.1 (-0.4; 0.3)<br>n=15	-0.021 (0.249)<br>0 (-0.4; 0.3)<br>n=14	0.079 (-0.064; 0.220)<br>p=0.30	0.103 (-0.067; 0.271)<br>p=0.25	0.055 (-0.125; 0.233)<br>p=0.57
**C-reactive protein (mg/L) at screening**	0.939 (3.682)<br>0 (0; 21)<br>n=33	1.50 (4.94)<br>0 (0; 21)<br>n=18	0.267 (0.704)<br>0 (0; 2)<br>n=15	0.938 (2.265)<br>0 (0; 9)<br>n=16			
**C-reactive protein (mg/L) at week 1**	0.515 (1.439)<br>0 (0; 7)<br>n=33	0.833 (1.855)<br>0 (0; 7)<br>n=18	0.133 (0.516)<br>0 (0; 2)<br>n=15	1.57 (2.59)<br>0 (0; 8)<br>n=14			
**C-reactive protein (mg/L) at week 4**	1.16 (3.39)<br>0 (0; 18)<br>n=31	2.13 (4.56)<br>0 (0; 18)<br>n=16	0.133 (0.352)<br>0 (0; 1)<br>n=15	0.857 (1.916)<br>0 (0; 7)<br>n=14			
**C-reactive protein (mg/L) at week 8**	0.742 (1.483)<br>0 (0; 6)<br>n=31	1.38 (1.86)<br>0.5 (0; 6)<br>n=16	0.067 (0.258)<br>0 (0; 1)<br>n=15	0.857 (1.406)<br>0 (0; 4)<br>n=14			

Variable	High+Low dose (n=33)	High dose (n=18)	Low dose (n=15)	Placebo (n=16)	Difference and test between groups: High+Low dose vs Placebo	High dose vs Placebo	Low dose vs Placebo
**Change in C-reactive protein (mg/L) from screening to week 1**	-0.424 (2.488)<br>0 (-14; 1)<br>n=33	-0.667 (3.361)<br>0 (-14; 1)<br>n=18	-0.133 (0.516)<br>0 (-2; 0)<br>n=15	0.643 (1.550)<br>0 (-1; 5)<br>n=14	-1.07 (-2.57; -0.00)	-1.31 (-3.17; 0.17)	-0.776 (-1.625; -0.000)
**Change in C-reactive protein (mg/L) from screening to week 4**	0.194 (4.339)<br>0 (-15; 18)<br>n=31	0.500 (6.088)<br>0 (-15; 18)<br>n=16	-0.133 (0.640)<br>0 (-2; 1)<br>n=15	-0.071 (0.917)<br>0 (-2; 2)<br>n=14	0.265 (-1.857; 2.556)<br>p=0.76	0.571 (-2.333; 3.667)<br>p=0.59	-0.062 (-0.667; 0.500)<br>p=1.00
**Change in C-reactive protein (mg/L) from screening to week 8**	-0.226 (2.918)<br>0 (-15; 3)<br>n=31	-0.250 (4.058)<br>0 (-15; 3)<br>n=16	-0.200 (0.775)<br>0 (-2; 1)<br>n=15	-0.071 (2.235)<br>0 (-6; 4)<br>n=14	-0.154 (-2.000; 1.250)<br>p=1.00	-0.179 (-2.600; 1.875)<br>p=0.99	-0.129 (-1.333; 1.143)<br>p=0.93
**Red blood cell count (10^9/L) at screening**	240.8 (44.8)<br>242 (172; 331)<br>n=33	253.6 (49.9)<br>261 (173; 331)<br>n=18	225.5 (33.2)<br>228 (172; 295)<br>n=15	238.1 (41.8)<br>235 (175; 324)<br>n=16			
**Red blood cell count (10^9/L) at week 1**	249.3 (40.0)<br>246 (153; 346)<br>n=33	261.9 (40.7)<br>259.5 (204; 346)<br>n=18	234.1 (34.6)<br>242 (153; 297)<br>n=15	247.9 (32.5)<br>242 (198; 290)<br>n=14			
**Red blood cell count (10^9/L) at week 4**	253.6 (47.6)<br>248 (175; 385)<br>n=31	272.3 (49.2)<br>265 (198; 385)<br>n=16	233.6 (37.8)<br>231 (175; 302)<br>n=15	250.5 (33.7)<br>252 (194; 302)<br>n=14			
**Red blood cell count (10^9/L) at week 8**	247.8 (45.1)<br>246 (169; 340)<br>n=31	264.2 (43.1)<br>261.5 (194; 340)<br>n=16	230.3 (41.6)<br>225 (169; 310)<br>n=15	243.6 (34.5)<br>231.5 (201; 319)<br>n=14			
**Change in Red blood cell count (10^9/L) from screening to week 1**	8.45 (27.39)<br>9 (-48; 57)<br>n=33	8.39 (32.27)<br>10.5 (-48; 57)<br>n=18	8.53 (21.22)<br>9 (-24; 55)<br>n=15	10.1 (25.7)<br>20.5 (-38; 44)<br>n=14	-1.62 (-18.90; 15.25)	-1.68 (-23.20; 19.87)	-1.54 (-19.29; 16.50)
**Change in Red blood cell count (10^9/L) from screening to week 4**	17.9 (30.5)<br>10 (-20; 100)<br>n=31	27.1 (37.5)<br>23.5 (-20; 100)<br>n=16	8.07 (16.88)<br>7 (-14; 41)<br>n=15	12.6 (25.5)<br>9.5 (-36; 71)<br>n=14	5.23 (-13.00; 24.78)<br>p=0.60	14.4 (-9.4; 38.6)<br>p=0.24	-4.58 (-20.86; 11.80)<br>p=0.59
**Change in Red blood cell count (10^9/L) from screening to week 8**	12.1 (25.5)<br>4 (-41; 66)<br>n=31	18.9 (29.0)<br>16 (-41; 66)<br>n=16	4.73 (19.59)<br>-1 (-22; 39)<br>n=15	5.79 (39.50)<br>15 (-90; 88)<br>n=14	6.28 (-14.09; 26.60)<br>p=0.52	13.2 (-12.5; 38.6)<br>p=0.32	-1.05 (-24.20; 22.29)<br>p=0.93
**White blood cell count (10^9/L) at screening**	4.72 (1.18)<br>4.4 (2.9; 7.1)<br>n=33	4.83 (1.28)<br>4.65 (2.9; 7.1)<br>n=18	4.59 (1.07)<br>4.1 (3.6; 6.8)<br>n=15	4.91 (0.87)<br>5.05 (3.6; 6.7)<br>n=16			
**White blood cell count (10^9/L) at week 1**	5.20 (1.40)<br>4.9 (3.4; 10)<br>n=33	5.49 (1.67)<br>5.25 (3.4; 10)<br>n=18	4.85 (0.93)<br>4.6 (3.7; 7)<br>n=15	5.06 (1.04)<br>4.85 (4; 7.1)<br>n=14			

					Difference and test between groups		
**Variable**	**High+Low dose (n=33)**	**High dose (n=18)**	**Low dose (n=15)**	**Placebo (n=16)**	**High+Low dose vs Placebo**	**High dose vs Placebo**	**Low dose vs Placebo**
**White blood cell count (10^9/L) at week 4**	5.25 (1.28)<br>5 (3.5; 7.9)<br>n=31	5.37 (1.35)<br>5.15 (3.6; 7.9)<br>n=16	5.13 (1.24)<br>4.7 (3.5; 7.1)<br>n=15	5.45 (1.06)<br>5.3 (3.8; 7.9)<br>n=14			
**White blood cell count (10^9/L) at week 8**	5.35 (1.64)<br>5 (3.1; 8.8)<br>n=31	5.73 (1.87)<br>5.75 (3.1; 8.8)<br>n=16	4.94 (1.30)<br>4.6 (3.3; 7.4)<br>n=15	5.44 (1.42)<br>4.85 (3.6; 8.2)<br>n=14			
**Change in White blood cell count (10^9/L) from screening to week 1**	0.476 (0.888)<br>0.3 (-1; 3.6)<br>n=33	0.656 (1.031)<br>0.45 (-0.5; 3.6)<br>n=18	0.260 (0.649)<br>0.3 (-1; 1.5)<br>n=15	0.121 (1.090)<br>0.25 (-1.6; 2.2)<br>n=14	0.354 (-0.242; 0.986)	0.534 (-0.217; 1.314)	0.139 (-0.533; 0.817)
**Change in White blood cell count (10^9/L) from screening to week 4**	0.584 (0.798)<br>0.4 (-0.5; 3)<br>n=31	0.625 (0.770)<br>0.45 (-0.5; 2.2)<br>n=16	0.540 (0.852)<br>0.4 (-0.5; 3)<br>n=15	0.514 (1.156)<br>0.4 (-1.4; 2.8)<br>n=14	0.070 (-0.512; 0.690)<br>p=0.84	0.111 (-0.617; 0.833)<br>p=0.78	0.026 (-0.750; 0.800)<br>p=0.96
**Change in White blood cell count (10^9/L) from screening to week 8**	0.677 (1.011)<br>0.6 (-1.1; 3)<br>n=31	0.988 (1.110)<br>0.8 (-0.8; 3)<br>n=16	0.347 (0.801)<br>0.4 (-1.1; 1.8)<br>n=15	0.500 (1.173)<br>0.1 (-0.9; 2.8)<br>n=14	0.177 (-0.510; 0.886)<br>p=0.62	0.487 (-0.360; 1.350)<br>p=0.26	-0.153 (-0.914; 0.614)<br>p=0.70
**Platelet count (10^9/L) at screening**	6.00 (6.85)<br>3 (1; 29)<br>n=33	7.33 (8.35)<br>3 (1; 29)<br>n=18	4.40 (4.19)<br>3 (1; 15)<br>n=15	6.69 (4.29)<br>5 (2; 16)<br>n=16			
**Platelet count (10^9/L) at week 1**	5.30 (4.90)<br>3 (1; 18)<br>n=33	6.50 (5.99)<br>3.5 (1; 18)<br>n=18	3.87 (2.67)<br>3 (1; 10)<br>n=15	6.21 (3.66)<br>5.5 (1; 13)<br>n=14			
**Platelet count (10^9/L) at week 4**	5.81 (5.55)<br>4 (1; 23)<br>n=31	7.38 (6.95)<br>4.5 (1; 23)<br>n=16	4.13 (2.92)<br>3 (1; 10)<br>n=15	5.50 (3.18)<br>4 (2; 12)<br>n=14			
**Platelet count (10^9/L) at week 8**	4.77 (4.78)<br>3 (1; 22)<br>n=31	6.19 (6.08)<br>4 (1; 22)<br>n=16	3.27 (2.15)<br>3 (1; 9)<br>n=15	6.07 (3.85)<br>5 (2; 15)<br>n=14			
**Change in Platelet count (10^9/L) from screening to week 1**	-0.697 (2.592)<br>0 (-11; 3)<br>n=33	-0.833 (2.995)<br>0 (-11; 3)<br>n=18	-0.533 (2.100)<br>0 (-5; 2)<br>n=15	-0.714 (2.972)<br>-0.5 (-5; 4)<br>n=14	0.017 (-1.800; 1.667)	-0.119 (-2.333; 2.000)	0.181 (-1.800; 2.143)
**Change in Platelet count (10^9/L) from screening to week 4**	0.677 (4.571)<br>0 (-6; 20)<br>n=31	1.56 (5.81)<br>0 (-6; 20)<br>n=16	-0.267 (2.604)<br>0 (-6; 5)<br>n=15	-1.43 (2.24)<br>-1 (-7; 1)<br>n=14	2.11 (-0.12; 4.86)<br>p=0.077	2.99 (0.00; 6.33)<br>p=0.059	1.16 (-0.71; 3.00)<br>p=0.24
**Change in Platelet count (10^9/L) from screening to week 8**	-0.355 (2.138)<br>0 (-7; 3)<br>n=31	0.375 (1.455)<br>0 (-3; 3)<br>n=16	-1.13 (2.50)<br>0 (-7; 2)<br>n=15	-0.857 (3.655)<br>-0.5 (-7; 5)<br>n=14	0.502 (-1.300; 2.222)<br>p=0.60	1.23 (-0.83; 3.29)<br>p=0.26	-0.276 (-2.667; 2.143)<br>p=0.86

					Difference and test between groups		
**Variable**	**High+Low dose (n=33)**	**High dose (n=18)**	**Low dose (n=15)**	**Placebo (n=16)**	**High+Low dose vs Placebo**	**High dose vs Placebo**	**Low dose vs Placebo**
**Alanine transaminase (µkat/L) at screening**	0.352 (0.154)<br>0.31 (0.13; 0.77)<br>n=33	0.407 (0.175)<br>0.345 (0.21; 0.77)<br>n=18	0.286 (0.092)<br>0.29 (0.13; 0.47)<br>n=15	0.418 (0.202)<br>0.35 (0.19; 0.83)<br>n=16			
**Alanine transaminase (µkat/L) at week 1**	0.340 (0.163)<br>0.33 (0; 0.97)<br>n=33	0.392 (0.181)<br>0.335 (0.2; 0.97)<br>n=18	0.277 (0.116)<br>0.33 (0; 0.42)<br>n=15	0.509 (0.453)<br>0.335 (0.19; 1.8)<br>n=14			
**Alanine transaminase (µkat/L) at week 4**	0.327 (0.142)<br>0.32 (0.12; 0.84)<br>n=31	0.382 (0.166)<br>0.33 (0.2; 0.84)<br>n=16	0.269 (0.082)<br>0.26 (0.12; 0.41)<br>n=15	0.364 (0.224)<br>0.285 (0.23; 1.1)<br>n=14			
**Alanine transaminase (µkat/L) at week 8**	0.358 (0.186)<br>0.33 (0.12; 1.1)<br>n=31	0.427 (0.225)<br>0.395 (0.18; 1.1)<br>n=16	0.285 (0.094)<br>0.26 (0.12; 0.44)<br>n=15	0.366 (0.281)<br>0.295 (0.17; 1.3)<br>n=14			
**Change in Alanine transaminase (µkat/L) from screening to week 1**	-0.012 (0.098)<br>0 (-0.2; 0.2)<br>n=33	-0.015 (0.118)<br>0.005 (-0.2; 0.2)<br>n=18	-0.009 (0.070)<br>-0.02 (-0.13; 0.15)<br>n=15	0.114 (0.416)<br>0.01 (-0.38; 1.42)<br>n=14	-0.126 (-0.262; 0.035)	-0.129 (-0.324; 0.063)	-0.123 (-0.330; 0.064)
**Change in Alanine transaminase (µkat/L) from screening to week 4**	-0.028 (0.084)<br>-0.01 (-0.28; 0.11)<br>n=31	-0.039 (0.110)<br>-0.015 (-0.28; 0.11)<br>n=16	-0.017 (0.045)<br>-0.01 (-0.15; 0.04)<br>n=15	-0.031 (0.148)<br>-0.025 (-0.39; 0.31)<br>n=14	0.003 (-0.069; 0.073)<br>p=0.93	-0.007 (-0.104; 0.090)<br>p=0.89	0.015 (-0.065; 0.094)<br>p=0.72
**Change in Alanine transaminase (µkat/L) from screening to week 8**	0.003 (0.103)<br>-0.01 (-0.23; 0.33)<br>n=31	0.006 (0.137)<br>-0.01 (-0.23; 0.33)<br>n=16	-0.001 (0.052)<br>-0.02 (-0.07; 0.12)<br>n=15	-0.029 (0.203)<br>-0.025 (-0.44; 0.51)<br>n=14	0.032 (-0.060; 0.127)<br>p=0.50	0.036 (-0.092; 0.162)<br>p=0.60	0.028 (-0.080; 0.133)<br>p=0.62
**Aspartate transaminase (µkat/L) at screening**	0.414 (0.158)<br>0.37 (0.28; 1.2)<br>n=33	0.429 (0.207)<br>0.355 (0.28; 1.2)<br>n=18	0.395 (0.069)<br>0.37 (0.29; 0.55)<br>n=15	0.472 (0.123)<br>0.425 (0.26; 0.69)<br>n=16			
**Aspartate transaminase (µkat/L) at week 1**	0.386 (0.101)<br>0.38 (0.23; 0.74)<br>n=33	0.401 (0.116)<br>0.38 (0.23; 0.74)<br>n=18	0.369 (0.080)<br>0.39 (0.26; 0.51)<br>n=15	0.827 (1.579)<br>0.385 (0.23; 6.3)<br>n=14			
**Aspartate transaminase (µkat/L) at week 4**	0.388 (0.083)<br>0.37 (0.25; 0.65)<br>n=31	0.389 (0.070)<br>0.38 (0.25; 0.53)<br>n=16	0.387 (0.098)<br>0.35 (0.29; 0.65)<br>n=15	0.401 (0.103)<br>0.36 (0.28; 0.61)<br>n=14			
**Aspartate transaminase (µkat/L) at week 8**	0.395 (0.089)<br>0.41 (0.19; 0.58)<br>n=31	0.416 (0.080)<br>0.435 (0.29; 0.58)<br>n=16	0.373 (0.095)<br>0.34 (0.19; 0.55)<br>n=15	0.409 (0.103)<br>0.395 (0.25; 0.69)<br>n=14			
**Change in Aspartate transaminase (µkat/L) from screening to week 1**	-0.028 (0.099)<br>0 (-0.46; 0.1)<br>n=33	-0.029 (0.129)<br>0.02 (-0.46; 0.1)<br>n=18	-0.026 (0.047)<br>-0.01 (-0.11; 0.03)<br>n=15	0.376 (1.592)<br>-0.03 (-0.22; 5.9)<br>n=14	-0.403 (-0.791; 0.062)	-0.405 (-1.013; 0.084)	-0.402 (-1.150; 0.066)

					Difference and test between groups		
Variable	High+Low dose (n=33)	High dose (n=18)	Low dose (n=15)	Placebo (n=16)	High+Low dose vs Placebo	High dose vs Placebo	Low dose vs Placebo
**Change in Aspartate transaminase (µkat/L) from screening to week 4**	-0.031 (0.160)<br>-0.01 (-0.8; 0.17)<br>n=31	-0.053 (0.215)<br>-0.015 (-0.8; 0.17)<br>n=16	-0.009 (0.064)<br>0 (-0.11; 0.13)<br>n=15	-0.051 (0.083)<br>-0.055 (-0.21; 0.11)<br>n=14	0.019 (-0.077; 0.090)<br>p=0.63	-0.002 (-0.120; 0.101)<br>p=0.98	0.042 (-0.014; 0.099)<br>p=0.15
**Change in Aspartate transaminase (µkat/L) from screening to week 8**	-0.024 (0.150)<br>0 (-0.73; 0.12)<br>n=31	-0.026 (0.202)<br>0.02 (-0.73; 0.12)<br>n=16	-0.022 (0.066)<br>-0.03 (-0.18; 0.07)<br>n=15	-0.042 (0.107)<br>-0.025 (-0.22; 0.12)<br>n=14	0.018 (-0.078; 0.092)<br>p=0.65	0.016 (-0.103; 0.123)<br>p=0.83	0.020 (-0.047; 0.087)<br>p=0.56
**Alkaline phosphatase (µkat/L) at screening**	0.978 (0.224)<br>0.94 (0.65; 1.6)<br>n=33	0.923 (0.150)<br>0.915 (0.68; 1.3)<br>n=18	1.04 (0.28)<br>0.97 (0.65; 1.6)<br>n=15	1.15 (0.38)<br>1.1 (0.44; 1.6)<br>n=16			
**Alkaline phosphatase (µkat/L) at week 1**	0.982 (0.245)<br>0.9 (0.68; 1.5)<br>n=33	0.922 (0.181)<br>0.87 (0.7; 1.4)<br>n=18	1.06 (0.30)<br>0.95 (0.68; 1.5)<br>n=15	1.15 (0.38)<br>1.15 (0.52; 1.8)<br>n=14			
**Alkaline phosphatase (µkat/L) at week 4**	0.986 (0.226)<br>0.91 (0.74; 1.5)<br>n=31	0.930 (0.190)<br>0.86 (0.74; 1.5)<br>n=16	1.05 (0.25)<br>0.94 (0.78; 1.5)<br>n=15	1.12 (0.34)<br>1.2 (0.44; 1.6)<br>n=14			
**Alkaline phosphatase (µkat/L) at week 8**	0.993 (0.236)<br>0.96 (0.69; 1.6)<br>n=31	0.944 (0.171)<br>0.94 (0.73; 1.4)<br>n=16	1.04 (0.29)<br>0.97 (0.69; 1.6)<br>n=15	1.11 (0.33)<br>1.1 (0.58; 1.6)<br>n=14			
**Change in Alkaline phosphatase (µkat/L) from screening to week 1**	0.005 (0.073)<br>0 (-0.1; 0.2)<br>n=33	-0.002 (0.068)<br>0 (-0.1; 0.1)<br>n=18	0.013 (0.081)<br>0 (-0.1; 0.2)<br>n=15	-0.011 (0.139)<br>0 (-0.4; 0.2)<br>n=14	0.016 (-0.049; 0.077)	0.009 (-0.066; 0.085)	0.023 (-0.060; 0.108)
**Change in Alkaline phosphatase (µkat/L) from screening to week 4**	0.009 (0.109)<br>0 (-0.3; 0.2)<br>n=31	0.013 (0.107)<br>0.045 (-0.2; 0.2)<br>n=16	0.004 (0.115)<br>0 (-0.3; 0.2)<br>n=15	-0.047 (0.159)<br>0 (-0.4; 0.28)<br>n=14	0.056 (-0.029; 0.137)<br>p=0.19	0.060 (-0.040; 0.161)<br>p=0.24	0.051 (-0.052; 0.158)<br>p=0.33
**Change in Alkaline phosphatase (µkat/L) from screening to week 8**	0.015 (0.106)<br>0 (-0.22; 0.21)<br>n=31	0.027 (0.089)<br>0.03 (-0.22; 0.2)<br>n=16	0.002 (0.124)<br>0 (-0.2; 0.21)<br>n=15	-0.056 (0.147)<br>-0.01 (-0.4; 0.14)<br>n=14	0.071 (-0.009; 0.149)<br>p=0.082	0.083 (-0.006; 0.174)<br>p=0.069	0.058 (-0.044; 0.164)<br>p=0.26
**Bilirubin (µmol/L) at screening**	11.5 (8.1)<br>9 (3.8; 37)<br>n=33	10.4 (7.7)<br>8.1 (3.8; 37)<br>n=18	12.9 (8.6)<br>10 (5.3; 37)<br>n=15	10.5 (5.0)<br>9.5 (3.7; 23)<br>n=16			
**Bilirubin (µmol/L) at week 1**	10.1 (5.1)<br>8.2 (4; 25)<br>n=33	9.87 (5.50)<br>7.5 (4; 25)<br>n=18	10.4 (4.8)<br>9.3 (4; 19)<br>n=15	9.59 (4.06)<br>9.35 (4.5; 18)<br>n=14			
**Bilirubin (µmol/L) at week 4**	10.9 (6.0)<br>9.3 (3.2; 30)<br>n=31	9.13 (3.52)<br>8.45 (3.2; 15)<br>n=16	12.7 (7.5)<br>9.9 (3.4; 30)<br>n=15	9.21 (4.32)<br>9 (0; 17)<br>n=14			

					Difference and test between groups		
**Variable**	**High+Low dose (n=33)**	**High dose (n=18)**	**Low dose (n=15)**	**Placebo (n=16)**	**High+Low dose vs Placebo**	**High dose vs Placebo**	**Low dose vs Placebo**
**Bilirubin (µmol/L) at week 8**	10.6 (5.3)<br>9.4 (0; 23)<br>n=31	9.73 (5.10)<br>9.65 (0; 23)<br>n=16	11.5 (5.5)<br>9.3 (3.3; 20)<br>n=15	9.64 (4.35)<br>7.8 (5.7; 19)<br>n=14			
**Change in Bilirubin (µmol/L) from screening to week 1**	-1.42 (5.01)<br>-0.6 (-22; 5.4)<br>n=33	-0.528 (3.615)<br>-0.45 (-12; 5.4)<br>n=18	-2.50 (6.27)<br>-1.3 (-22; 5)<br>n=15	-0.286 (4.141)<br>-0.5 (-9.9; 8.4)<br>n=14	-1.14 (-4.35; 1.63)	-0.242 (-3.040; 2.489)	-2.21 (-6.30; 1.68)
**Change in Bilirubin (µmol/L) from screening to week 4**	-1.04 (5.99)<br>0 (-24; 11)<br>n=31	-1.84 (6.49)<br>-0.15 (-24; 4)<br>n=16	-0.180 (5.492)<br>0 (-12; 11)<br>n=15	-0.657 (3.922)<br>-1.35 (-5.8; 9.3)<br>n=14	-0.382 (-4.080; 2.911)<br>p=0.88	-1.19 (-5.22; 2.49)<br>p=0.63	0.477 (-3.187; 4.062)<br>p=0.79
**Change in Bilirubin (µmol/L) from screening to week 8**	-1.30 (5.21)<br>0 (-19; 5.9)<br>n=31	-1.25 (5.18)<br>0.4 (-14; 5.9)<br>n=16	-1.36 (5.42)<br>0 (-19; 5)<br>n=15	-0.236 (5.372)<br>-1 (-7.8; 11.3)<br>n=14	-1.07 (-4.65; 2.21)<br>p=0.54	-1.01 (-5.08; 2.90)<br>p=0.61	-1.12 (-5.24; 2.90)<br>p=0.60
**Renal function eGFR (ml/min) at screening**	90.4 (10.0)<br>91 (71; 115)<br>n=33	90.1 (8.8)<br>91 (71; 104)<br>n=18	90.7 (11.5)<br>90 (71; 115)<br>n=15	89.6 (9.3)<br>87.5 (76; 117)<br>n=16			
**Renal function eGFR (ml/min) at week 1**	90.9 (10.1)<br>89 (76; 117)<br>n=33	90.4 (10.7)<br>88 (79; 117)<br>n=18	91.5 (9.7)<br>91 (76; 111)<br>n=15	91.0 (9.1)<br>92 (75; 109)<br>n=14			
**Renal function eGFR (ml/min) at week 4**	90.7 (9.3)<br>88 (76; 111)<br>n=31	89.1 (9.0)<br>87.5 (76; 111)<br>n=16	92.4 (9.7)<br>89 (80; 107)<br>n=15	93.0 (9.5)<br>95.5 (72; 108)<br>n=14			
**Renal function eGFR (ml/min) at week 8**	90.4 (8.7)<br>90 (73; 110)<br>n=31	90.5 (9.4)<br>89.5 (75; 110)<br>n=16	90.3 (8.3)<br>90 (73; 101)<br>n=15	92.6 (8.5)<br>92 (76; 112)<br>n=14			
**Change in Renal function eGFR (ml/min) from screening to week 1**	0.545 (5.858)<br>0 (-10; 13)<br>n=33	0.278 (7.028)<br>-2 (-10; 13)<br>n=18	0.867 (4.274)<br>1 (-6; 7)<br>n=15	0.214 (4.726)<br>0 (-8; 7)<br>n=14	0.331 (-3.222; 3.889)	0.063 (-4.333; 4.625)	0.652 (-2.800; 4.125)
**Change in Renal function eGFR (ml/min) from screening to week 4**	0.645 (6.432)<br>0 (-10; 14)<br>n=31	-0.375 (6.270)<br>0 (-10; 11)<br>n=16	1.73 (6.64)<br>2 (-8; 14)<br>n=15	2.21 (6.99)<br>4.5 (-12; 12)<br>n=14	-1.57 (-5.89; 2.71)<br>p=0.49	-2.59 (-7.50; 2.33)<br>p=0.30	-0.481 (-5.667; 4.714)<br>p=0.87
**Change in Renal function eGFR (ml/min) from screening to week 8**	0.323 (5.776)<br>1 (-14; 13)<br>n=31	1.000 (5.610)<br>1 (-9; 13)<br>n=16	-0.400 (6.057)<br>-1 (-14; 9)<br>n=15	1.86 (5.32)<br>2 (-8; 10)<br>n=14	-1.53 (-5.22; 2.11)<br>p=0.42	-0.857 (-4.875; 3.250)<br>p=0.69	-2.26 (-6.60; 2.00)<br>p=0.31
**Blood total protein (g/l) at screening**	70.6 (4.6)<br>70.5 (62; 80)<br>n=32	70.9 (4.9)<br>72.5 (62; 80)<br>n=18	70.1 (4.3)<br>69.5 (64; 78)<br>n=14	72.9 (5.1)<br>73 (62; 81)<br>n=16			

					Difference and test between groups		
Variable	High+Low dose<br>(n=33)	High dose<br>(n=18)	Low dose<br>(n=15)	Placebo<br>(n=16)	High+Low dose vs<br>Placebo	High dose vs Placebo	Low dose vs Placebo
**Blood total protein (g/l) at week 1**	71.4 (3.5)<br>72 (65; 78)<br>n=33	71.6 (3.9)<br>72 (65; 78)<br>n=18	71.1 (3.1)<br>71 (66; 76)<br>n=15	71.7 (2.6)<br>72.5 (65; 75)<br>n=14			
**Blood total protein (g/l) at week 4**	71.4 (4.0)<br>71 (62; 81)<br>n=31	71.9 (4.9)<br>71 (62; 81)<br>n=16	70.9 (2.9)<br>71 (66; 75)<br>n=15	71.6 (3.9)<br>72.5 (60; 76)<br>n=14			
**Blood total protein (g/l) at week 8**	71.1 (3.7)<br>71 (64; 79)<br>n=31	72.3 (4.1)<br>72.5 (64; 79)<br>n=16	69.7 (2.7)<br>70 (65; 75)<br>n=15	71.9 (3.0)<br>72.5 (65; 75)<br>n=14			
**Change in Blood total protein (g/l) from screening to week 1**	0.719 (3.735)<br>1 (-7; 7)<br>n=32	0.722 (4.084)<br>0.5 (-7; 7)<br>n=18	0.714 (3.384)<br>1 (-6; 5)<br>n=14	-1.14 (3.76)<br>-1 (-7; 3)<br>n=14	1.86 (-0.56; 4.25)	1.87 (-1.00; 4.75)	1.86 (-1.00; 4.67)
**Change in Blood total protein (g/l) from screening to week 4**	0.967 (4.529)<br>0.5 (-8; 13)<br>n=30	1.38 (5.28)<br>1 (-8; 13)<br>n=16	0.500 (3.632)<br>0 (-4; 8)<br>n=14	-1.21 (5.55)<br>-1 (-10; 6)<br>n=14	2.18 (-1.00; 5.37)<br>p=0.19	2.59 (-1.43; 6.67)<br>p=0.22	1.71 (-2.00; 5.37)<br>p=0.37
**Change in Blood total protein (g/l) from screening to week 8**	0.767 (3.530)<br>1 (-9; 6)<br>n=30	1.81 (2.93)<br>2 (-3; 6)<br>n=16	-0.429 (3.877)<br>-1 (-9; 5)<br>n=14	-1.000 (4.574)<br>-0.5 (-8; 4)<br>n=14	1.77 (-0.86; 4.30)<br>p=0.19	2.81 (0.00; 5.71)<br>p=0.061	0.571 (-2.714; 3.857)<br>p=0.77
**Blood haemoglobin (g/l) at screening**	140.1 (13.4)<br>139 (118; 164)<br>n=33	140.0 (13.9)<br>139.5 (118; 164)<br>n=18	140.3 (13.4)<br>139 (124; 159)<br>n=15	141.8 (14.6)<br>140 (123; 169)<br>n=16			
**Blood haemoglobin (g/l) at week 1**	140.0 (12.3)<br>141 (119; 165)<br>n=33	140.4 (12.5)<br>142 (120; 162)<br>n=18	139.5 (12.5)<br>139 (119; 165)<br>n=15	138.1 (12.5)<br>139.5 (118; 161)<br>n=14			
**Blood haemoglobin (g/l) at week 4**	140.0 (12.3)<br>142 (117; 162)<br>n=31	139.8 (13.5)<br>144 (117; 160)<br>n=16	140.1 (11.3)<br>141 (124; 162)<br>n=15	138.9 (14.2)<br>137.5 (118; 171)<br>n=14			
**Blood haemoglobin (g/l) at week 8**	141.1 (11.3)<br>143 (120; 161)<br>n=31	141.8 (12.6)<br>144.5 (120; 161)<br>n=16	140.3 (10.1)<br>142 (127; 161)<br>n=15	138.7 (12.3)<br>141.5 (117; 161)<br>n=14			
**Change in Blood haemoglobin (g/l) from screening to week 1**	-0.121 (5.851)<br>0 (-12; 10)<br>n=33	0.444 (6.157)<br>1.5 (-12; 10)<br>n=18	-0.800 (5.596)<br>-1 (-11; 7)<br>n=15	-2.21 (6.07)<br>-3 (-12; 9)<br>n=14	2.09 (-1.73; 5.87)	2.66 (-1.78; 7.14)	1.41 (-3.00; 5.83)
**Change in Blood haemoglobin (g/l) from screening to week 4**	-0.032 (6.063)<br>-1 (-13; 14)<br>n=31	0.063 (6.060)<br>-1.5 (-8; 12)<br>n=16	-0.133 (6.278)<br>0 (-13; 14)<br>n=15	-1.43 (5.33)<br>-0.5 (-12; 6)<br>n=14	1.40 (-2.37; 5.25)<br>p=0.48	1.49 (-2.80; 5.71)<br>p=0.51	1.30 (-3.17; 5.71)<br>p=0.58

Variable	High+Low dose (n=33)	High dose (n=18)	Low dose (n=15)	Placebo (n=16)	Difference and test between groups: High+Low dose vs Placebo	High dose vs Placebo	Low dose vs Placebo
**Change in Blood haemoglobin (g/l) from screening to week 8**	1.06 (6.58)<br>2 (-14; 17)<br>n=31	2.00 (6.35)<br>0 (-10; 17)<br>n=16	0.067 (6.902)<br>3 (-14; 7)<br>n=15	-1.57 (7.08)<br>-0.5 (-16; 8)<br>n=14	2.64 (-1.78; 7.00)<br>p=0.24	3.57 (-1.43; 8.62)<br>p=0.16	1.64 (-3.67; 7.00)<br>p=0.55

For continuous variables Mean (SD) / Median (Min; Max) / n= is presented.
For comparison between groups the Fisher´s Non Parametric Permutation Test was used for continuous variables. The confidence interval for then mean difference between groups is based on Fishers non-parametric permutation test.
2020-01-01 Analys.sas

Table 14.2.4.1 Secondary Analysis - Change in glycaemic levels (ITT Population)

					Difference and test between groups		
**Variable**	**High+Low dose (n=34)**	**High dose (n=18)**	**Low dose (n=16)**	**Placebo (n=16)**	**High+Low dose vs Placebo**	**High dose vs Placebo**	**Low dose vs Placebo**
**Fasting blood glucose (mmol/L) at screening**	5.15 (0.40)<br>5.15 (4.5; 6)<br>n=34	5.29 (0.44)<br>5.25 (4.5; 6)<br>n=18	5.00 (0.28)<br>5 (4.5; 5.5)<br>n=16	5.21 (0.35)<br>5.25 (4.6; 5.7)<br>n=16			
**Fasting blood glucose (mmol/L) at week 1**	5.26 (0.37)<br>5.2 (4.7; 6.1)<br>n=34	5.37 (0.43)<br>5.3 (4.8; 6.1)<br>n=18	5.14 (0.26)<br>5.2 (4.7; 5.8)<br>n=16	5.14 (0.39)<br>5.2 (4.5; 5.7)<br>n=14			
**Fasting blood glucose (mmol/L) at week 4**	5.25 (0.38)<br>5.15 (4.5; 6)<br>n=32	5.33 (0.46)<br>5.3 (4.5; 6)<br>n=16	5.18 (0.27)<br>5.1 (4.8; 5.7)<br>n=16	5.26 (0.40)<br>5.35 (4.6; 6)<br>n=14			
**Fasting blood glucose (mmol/L) at week 8**	5.16 (0.42)<br>5.25 (4.4; 5.9)<br>n=32	5.23 (0.40)<br>5.3 (4.6; 5.9)<br>n=16	5.10 (0.45)<br>5 (4.4; 5.9)<br>n=16	5.18 (0.29)<br>5.1 (4.7; 5.6)<br>n=14			
**Change in Fasting blood glucose (mmol/L) from screening to week 1**	0.109 (0.223)<br>0.1 (-0.4; 0.5)<br>n=34	0.078 (0.262)<br>0.1 (-0.4; 0.4)<br>n=18	0.144 (0.171)<br>0.15 (-0.1; 0.5)<br>n=16	-0.021 (0.229)<br>-0.05 (-0.4; 0.5)<br>n=14	0.130 (-0.014; 0.273)	0.099 (-0.080; 0.280)	0.165 (0.017; 0.314)
**Change in Fasting blood glucose (mmol/L) from screening to week 4**	0.116 (0.336)<br>0.05 (-0.4; 1)<br>n=32	0.056 (0.333)<br>-0.05 (-0.4; 0.6)<br>n=16	0.175 (0.340)<br>0.15 (-0.2; 1)<br>n=16	0.100 (0.306)<br>0.1 (-0.6; 0.6)<br>n=14	0.016 (-0.189; 0.229)<br>p=0.93	-0.044 (-0.283; 0.200)<br>p=0.76	0.075 (-0.167; 0.320)<br>p=0.58
**Change in Fasting blood glucose (mmol/L) from screening to week 8**	0.028 (0.368)<br>0 (-0.7; 0.7)<br>n=32	-0.044 (0.379)<br>-0.1 (-0.7; 0.7)<br>n=16	0.100 (0.354)<br>0 (-0.5; 0.7)<br>n=16	0.021 (0.340)<br>0.15 (-0.7; 0.5)<br>n=14	0.007 (-0.222; 0.240)<br>p=0.98	-0.065 (-0.333; 0.200)<br>p=0.66	0.079 (-0.180; 0.340)<br>p=0.58
**Blood HBA1C (mmol/mol) at screening**	30.4 (2.6)<br>30 (26; 39)<br>n=34	30.6 (3.2)<br>30 (26; 39)<br>n=18	30.2 (1.8)<br>30.5 (26; 33)<br>n=16	30.4 (2.0)<br>31 (26; 33)<br>n=16			
**Blood HBA1C (mmol/mol) at week 1**	30.2 (2.5)<br>30 (25; 36)<br>n=33	30.5 (3.0)<br>30 (25; 36)<br>n=17	29.8 (2.0)<br>30.5 (25; 32)<br>n=16	30.1 (2.3)<br>30 (26; 34)<br>n=14			
**Blood HBA1C (mmol/mol) at week 4**	30.7 (2.5)<br>30.5 (25; 38)<br>n=32	31.6 (2.8)<br>31 (27; 38)<br>n=16	29.8 (1.9)<br>30 (25; 32)<br>n=16	30.0 (2.7)<br>30 (25; 35)<br>n=14			
**Blood HBA1C (mmol/mol) at week 8**	30.3 (2.5)<br>30 (25; 37)<br>n=32	31.1 (2.5)<br>31 (26; 37)<br>n=16	29.6 (2.3)<br>30 (25; 34)<br>n=16	29.9 (2.8)<br>29.5 (26; 36)<br>n=14			

					Difference and test between groups		
**Variable**	**High+Low dose (n=34)**	**High dose (n=18)**	**Low dose (n=16)**	**Placebo (n=16)**	**High+Low dose vs Placebo**	**High dose vs Placebo**	**Low dose vs Placebo**
**Change in Blood HBA1C (mmol/mol) from screening to week 1**	-0.242 (1.324)<br>0 (-3; 2)<br>n=33	-0.118 (1.219)<br>0 (-3; 2)<br>n=17	-0.375 (1.455)<br>-0.5 (-3; 2)<br>n=16	-0.214 (1.251)<br>0 (-3; 1)<br>n=14	-0.028 (-0.889; 0.800)	0.097 (-0.833; 1.000)	-0.161 (-1.167; 0.857)
**Change in Blood HBA1C (mmol/mol) from screening to week 4**	0.063 (1.523)<br>0 (-3; 3)<br>n=32	0.500 (1.211)<br>1 (-2; 3)<br>n=16	-0.375 (1.708)<br>0 (-3; 2)<br>n=16	-0.357 (1.646)<br>0 (-3; 2)<br>n=14	0.420 (-0.625; 1.400)<br>p=0.46	0.857 (-0.200; 2.000)<br>p=0.15	-0.018 (-1.286; 1.250)<br>p=1.00
**Change in Blood HBA1C (mmol/mol) from screening to week 8**	-0.344 (1.359)<br>0 (-3; 2)<br>n=32	-0.063 (1.124)<br>0 (-2; 2)<br>n=16	-0.625 (1.544)<br>-1 (-3; 2)<br>n=16	-0.500 (1.871)<br>-0.5 (-3; 3)<br>n=14	0.156 (-0.818; 1.143)<br>p=0.84	0.438 (-0.714; 1.600)<br>p=0.51	-0.125 (-1.429; 1.167)<br>p=0.92

For continuous variables Mean (SD) / Median (Min; Max) / n= is presented.
For comparison between groups the Fisher´s Non Parametric Permutation Test was used for continuous variables. The confidence interval for then mean difference between groups is based on Fishers non-parametric permutation test.
2020-01-01 Analys.sas

Table 14.2.4.2 Secondary Analysis - Change in glycaemic levels (PP Population)

					Difference and test between groups		
**Variable**	**High+Low dose (n=33)**	**High dose (n=18)**	**Low dose (n=15)**	**Placebo (n=16)**	**High+Low dose vs Placebo**	**High dose vs Placebo**	**Low dose vs Placebo**
**Fasting blood glucose (mmol/L) at screening**	5.16 (0.40)<br>5.2 (4.5; 6)<br>n=33	5.29 (0.44)<br>5.25 (4.5; 6)<br>n=18	5.00 (0.29)<br>5 (4.5; 5.5)<br>n=15	5.21 (0.35)<br>5.25 (4.6; 5.7)<br>n=16			
**Fasting blood glucose (mmol/L) at week 1**	5.26 (0.37)<br>5.2 (4.7; 6.1)<br>n=33	5.37 (0.43)<br>5.3 (4.8; 6.1)<br>n=18	5.14 (0.27)<br>5.2 (4.7; 5.8)<br>n=15	5.14 (0.39)<br>5.2 (4.5; 5.7)<br>n=14			
**Fasting blood glucose (mmol/L) at week 4**	5.26 (0.38)<br>5.2 (4.5; 6)<br>n=31	5.33 (0.46)<br>5.3 (4.5; 6)<br>n=16	5.20 (0.26)<br>5.1 (4.9; 5.7)<br>n=15	5.26 (0.40)<br>5.35 (4.6; 6)<br>n=14			
**Fasting blood glucose (mmol/L) at week 8**	5.17 (0.43)<br>5.3 (4.4; 5.9)<br>n=31	5.23 (0.40)<br>5.3 (4.6; 5.9)<br>n=16	5.11 (0.47)<br>5 (4.4; 5.9)<br>n=15	5.18 (0.29)<br>5.1 (4.7; 5.6)<br>n=14			
**Change in Fasting blood glucose (mmol/L) from screening to week 1**	0.106 (0.226)<br>0.1 (-0.4; 0.5)<br>n=33	0.078 (0.262)<br>0.1 (-0.4; 0.4)<br>n=18	0.140 (0.176)<br>0.1 (-0.1; 0.5)<br>n=15	-0.021 (0.229)<br>-0.05 (-0.4; 0.5)<br>n=14	0.127 (-0.020; 0.275)	0.099 (-0.080; 0.280)	0.161 (0.000; 0.317)
**Change in Fasting blood glucose (mmol/L) from screening to week 4**	0.126 (0.337)<br>0.1 (-0.4; 1)<br>n=31	0.056 (0.333)<br>-0.05 (-0.4; 0.6)<br>n=16	0.200 (0.336)<br>0.2 (-0.2; 1)<br>n=15	0.100 (0.306)<br>0.1 (-0.6; 0.6)<br>n=14	0.026 (-0.186; 0.244)<br>p=0.86	-0.044 (-0.283; 0.200)<br>p=0.76	0.100 (-0.143; 0.350)<br>p=0.45
**Change in Fasting blood glucose (mmol/L) from screening to week 8**	0.029 (0.374)<br>0 (-0.7; 0.7)<br>n=31	-0.044 (0.379)<br>-0.1 (-0.7; 0.7)<br>n=16	0.107 (0.365)<br>0 (-0.5; 0.7)<br>n=15	0.021 (0.340)<br>0.15 (-0.7; 0.5)<br>n=14	0.008 (-0.225; 0.243)<br>p=0.98	-0.065 (-0.333; 0.200)<br>p=0.66	0.085 (-0.183; 0.357)<br>p=0.56
**Blood HBA1C (mmol/mol) at screening**	30.5 (2.5)<br>30 (26; 39)<br>n=33	30.6 (3.2)<br>30 (26; 39)<br>n=18	30.5 (1.4)<br>31 (28; 33)<br>n=15	30.4 (2.0)<br>31 (26; 33)<br>n=16			
**Blood HBA1C (mmol/mol) at week 1**	30.3 (2.5)<br>30.5 (25; 36)<br>n=32	30.5 (3.0)<br>30 (25; 36)<br>n=17	30.0 (1.9)<br>31 (25; 32)<br>n=15	30.1 (2.3)<br>30 (26; 34)<br>n=14			
**Blood HBA1C (mmol/mol) at week 4**	30.8 (2.5)<br>31 (25; 38)<br>n=31	31.6 (2.8)<br>31 (27; 38)<br>n=16	29.9 (1.9)<br>30 (25; 32)<br>n=15	30.0 (2.7)<br>30 (25; 35)<br>n=14			
**Blood HBA1C (mmol/mol) at week 8**	30.4 (2.4)<br>30 (25; 37)<br>n=31	31.1 (2.5)<br>31 (26; 37)<br>n=16	29.7 (2.3)<br>30 (25; 34)<br>n=15	29.9 (2.8)<br>29.5 (26; 36)<br>n=14			

Variable	High+Low dose (n=33)	High dose (n=18)	Low dose (n=15)	Placebo (n=16)	Difference and test between groups: High+Low dose vs Placebo	High dose vs Placebo	Low dose vs Placebo
**Change in Blood HBA1C (mmol/mol) from screening to week 1**	-0.281 (1.326)<br>0 (-3; 2)<br>n=32	-0.118 (1.219)<br>0 (-3; 2)<br>n=17	-0.467 (1.457)<br>-1 (-3; 2)<br>n=15	-0.214 (1.251)<br>0 (-3; 1)<br>n=14	-0.067 (-0.900; 0.750)	0.097 (-0.833; 1.000)	-0.252 (-1.333; 0.800)
**Change in Blood HBA1C (mmol/mol) from screening to week 4**	0.000 (1.506)<br>0 (-3; 3)<br>n=31	0.500 (1.211)<br>1 (-2; 3)<br>n=16	-0.533 (1.642)<br>0 (-3; 2)<br>n=15	-0.357 (1.646)<br>0 (-3; 2)<br>n=14	0.357 (-0.700; 1.364)<br>p=0.55	0.857 (-0.200; 2.000)<br>p=0.15	-0.176 (-1.429; 1.125)<br>p=0.86
**Change in Blood HBA1C (mmol/mol) from screening to week 8**	-0.387 (1.358)<br>0 (-3; 2)<br>n=31	-0.063 (1.124)<br>0 (-2; 2)<br>n=16	-0.733 (1.534)<br>-1 (-3; 2)<br>n=15	-0.500 (1.871)<br>-0.5 (-3; 3)<br>n=14	0.113 (-0.889; 1.111)<br>p=0.91	0.438 (-0.714; 1.600)<br>p=0.51	-0.233 (-1.556; 1.111)<br>p=0.80

For continuous variables Mean (SD) / Median (Min; Max) / n= is presented.
For comparison between groups the Fisher´s Non Parametric Permutation Test was used for continuous variables. The confidence interval for then mean difference between groups is based on Fishers non-parametric permutation test.
2020-01-01 Analys.sas

Table 14.2.5.1 Secondary Analysis - Relative change in short-chain fatty acids in stool (ITT Population)

					Difference and test between groups		
Variable	High+Low dose (n=34)	High dose (n=18)	Low dose (n=16)	Placebo (n=16)	High+Low dose vs Placebo	High dose vs Placebo	Low dose vs Placebo
**Butyrate at randomization**	54.0 (43.2)<br>40.1 (6.2; 221.3)<br>n=32	56.7 (52.5)<br>33.2 (6.2; 221.3)<br>n=16	51.4 (33.0)<br>41.6 (11.7; 122.7)<br>n=16	85.3 (60.9)<br>70.1 (23.8; 241.4)<br>n=14			
**Butyrate at week 1**	77.9 (67.2)<br>68.8 (5.8; 363.2)<br>n=31	62.2 (39.1)<br>62.8 (11.1; 166.5)<br>n=15	92.6 (84.4)<br>84.6 (5.8; 363.2)<br>n=16	111.0 (84.5)<br>80.1 (8.5; 280.5)<br>n=13			
**Butyrate at week 4**	77.8 (66.4)<br>63 (5; 305.4)<br>n=31	62.1 (44.4)<br>54 (5; 152.4)<br>n=15	92.6 (80.6)<br>66.5 (14.7; 305.4)<br>n=16	112.1 (91.5)<br>96.9 (13.5; 354.7)<br>n=14			
**Butyrate at week 8**	64.7 (56.0)<br>48.3 (14.7; 273.6)<br>n=30	42.8 (29.7)<br>32.9 (14.7; 113.5)<br>n=14	83.8 (66.8)<br>60.2 (30.5; 273.6)<br>n=16	119.9 (141.9)<br>66.6 (13.2; 488.4)<br>n=13			
**Butyrate at week 10**	83.0 (72.5)<br>69.9 (9.7; 389.5)<br>n=32	62.4 (48.6)<br>46.1 (9.7; 185.5)<br>n=16	103.5 (87.1)<br>94.4 (12.7; 389.5)<br>n=16	90.0 (80.5)<br>46 (6; 245)<br>n=14			
**Relative change in Butyrate from randomization to week 1**	83.0 (130.4)<br>16.4 (-74.2; 428.1)<br>n=31	70.5 (144.9)<br>12.8 (-74.2; 428.1)<br>n=15	94.6 (118.8)<br>103.1 (-64.9; 290.2)<br>n=16	66.7 (128.3)<br>51.8 (-77.5; 433.6)<br>n=13	16.3 (-68.7; 105.7)	3.84 (-101.11; 111.03)	27.9 (-64.9; 121.2)
**Relative change in Butyrate from randomization to week 4**	85.1 (160.3)<br>22 (-83.8; 522.3)<br>n=31	53.3 (144.2)<br>18 (-83.8; 396.5)<br>n=15	114.9 (173.2)<br>81.4 (-58.2; 522.3)<br>n=16	71.0 (144.7)<br>20.9 (-75.6; 433)<br>n=14	14.0 (-83.1; 119.6)<br>p=0.80	-17.7 (-127.7; 93.4)<br>p=0.75	43.8 (-75.0; 165.9)<br>p=0.45
**Relative change in Butyrate from randomization to week 8**	128.2 (429.8)<br>1.5 (-75.3; 2239.4)<br>n=30	32.8 (127.1)<br>-22.2 (-75.3; 309.2)<br>n=14	211.7 (572.0)<br>14.9 (-58.6; 2239.4)<br>n=16	53.4 (131.3)<br>-0.1 (-81.8; 351.8)<br>n=13	74.8 (-82.7; 335.7)<br>p=0.74	-20.6 (-123.7; 81.1)<br>p=0.68	158.3 (-79.2; 472.6)<br>p=0.43
**Relative change in Butyrate from randomization to week 10**	177.2 (575.9)<br>39.5 (-86.3; 3229.9)<br>n=32	68.7 (173.7)<br>12.2 (-70; 605.3)<br>n=16	285.7 (793.8)<br>72.5 (-86.3; 3229.9)<br>n=16	2.84 (58.79)<br>-3.07 (-84.25; 120.27)<br>n=14	174.4 (6.7; 495.5)<br>p=0.030	65.9 (-23.3; 163.9)<br>p=0.18	282.9 (25.2; 665.6)<br>p=0.0093
**Propionate at randomization**	83.0 (75.1)<br>58.3 (10.7; 393.5)<br>n=32	76.2 (53.2)<br>64.5 (10.7; 190)<br>n=16	89.7 (93.5)<br>51.1 (17.2; 393.5)<br>n=16	155.2 (174.8)<br>79.5 (27.9; 651.5)<br>n=14			
**Propionate at week 1**	105.2 (94.4)<br>85.1 (22; 458.2)<br>n=31	98.9 (85.7)<br>79.2 (22; 381.9)<br>n=15	111.1 (104.3)<br>91.4 (22.9; 458.2)<br>n=16	119.8 (90.4)<br>97.4 (22.4; 343.5)<br>n=13			

					Difference and test between groups		
Variable	High+Low dose (n=34)	High dose (n=18)	Low dose (n=16)	Placebo (n=16)	High+Low dose vs Placebo	High dose vs Placebo	Low dose vs Placebo
**Propionate at week 4**	96.0 (79.6)<br>77.3 (13.9; 368.6)<br>n=31	80.4 (44.0)<br>77.3 (18.3; 161.9)<br>n=15	110.7 (102.0)<br>78.6 (13.9; 368.6)<br>n=16	125.1 (112.5)<br>77.4 (11.3; 462.8)<br>n=14			
**Propionate at week 8**	77.3 (51.3)<br>52.3 (25.9; 210.4)<br>n=30	70.3 (47.0)<br>48.5 (25.9; 175.7)<br>n=14	83.5 (55.7)<br>68.3 (30.3; 210.4)<br>n=16	134.6 (133.1)<br>79 (15.3; 447.2)<br>n=13			
**Propionate at week 10**	93.2 (55.4)<br>88.8 (12.5; 246.5)<br>n=32	82.6 (48.3)<br>76.9 (12.5; 199.2)<br>n=16	103.9 (61.5)<br>93 (16.7; 246.5)<br>n=16	112.3 (103.2)<br>57.5 (23.7; 323.7)<br>n=14			
**Relative change in Propionate from randomization to week 1**	61.6 (122.4)<br>4.4 (-66.9; 486.2)<br>n=31	73.1 (152.9)<br>1.9 (-44.5; 486.2)<br>n=15	50.9 (88.9)<br>31.6 (-66.9; 257.5)<br>n=16	24.7 (70.5)<br>0.9 (-66.4; 143.3)<br>n=13	36.9 (-30.0; 114.2)	48.4 (-41.6; 143.4)	26.2 (-35.0; 89.1)
**Relative change in Propionate from randomization to week 4**	51.5 (143.7)<br>0.3 (-61.3; 677.5)<br>n=31	39.6 (100.5)<br>2.5 (-61.3; 250)<br>n=15	62.7 (177.7)<br>-3.5 (-43.5; 677.5)<br>n=16	21.1 (85.0)<br>-15.8 (-80.6; 184.1)<br>n=14	30.4 (-40.5; 120.0)<br>p=0.51	18.5 (-51.9; 91.2)<br>p=0.60	41.6 (-49.9; 146.4)<br>p=0.52
**Relative change in Propionate from randomization to week 8**	33.2 (111.0)<br>0.8 (-75.9; 413)<br>n=30	36.7 (138.1)<br>-21.1 (-74.3; 413)<br>n=14	30.2 (85.3)<br>2.4 (-75.9; 228.3)<br>n=16	41.0 (118.3)<br>33.1 (-87.9; 375.5)<br>n=13	-7.82 (-78.45; 72.01)<br>p=0.81	-4.32 (-104.65; 97.32)<br>p=0.94	-10.9 (-86.0; 65.0)<br>p=0.77
**Relative change in Propionate from randomization to week 10**	59.8 (132.5)<br>22.7 (-83.7; 615.3)<br>n=32	34.9 (81.2)<br>21 (-61.7; 256.6)<br>n=16	84.7 (168.5)<br>46.9 (-83.7; 615.3)<br>n=16	-12.6 (38.7)<br>-13.7 (-64.5; 57.5)<br>n=14	72.4 (11.0; 149.6)<br>p=0.016	47.5 (1.2; 95.9)<br>p=0.044	97.3 (14.1; 188.8)<br>p=0.017
**Acetate at randomization**	222.4 (120.5)<br>207.7 (56.8; 597.7)<br>n=32	222.0 (130.2)<br>203.4 (56.8; 597.7)<br>n=16	222.9 (114.1)<br>207.7 (71.2; 485.5)<br>n=16	352.6 (213.3)<br>293.3 (98; 719.3)<br>n=14			
**Acetate at week 1**	279.3 (202.9)<br>239.9 (20.5; 1101.6)<br>n=31	245.4 (156.6)<br>231.1 (20.5; 683.1)<br>n=15	311.0 (239.2)<br>252.3 (88.1; 1101.6)<br>n=16	347.5 (190.4)<br>314 (79.4; 655.3)<br>n=13			
**Acetate at week 4**	267.8 (176.6)<br>222.9 (48.9; 904.9)<br>n=31	252.8 (121.9)<br>256.9 (57; 525)<br>n=15	281.8 (219.3)<br>212.4 (48.9; 904.9)<br>n=16	343.4 (207.7)<br>317.7 (38.9; 773.6)<br>n=14			
**Acetate at week 8**	254.6 (180.2)<br>192.3 (87.2; 771.9)<br>n=30	223.0 (165.0)<br>142.3 (92.1; 655)<br>n=14	282.3 (193.4)<br>215.3 (87.2; 771.9)<br>n=16	369.5 (288.9)<br>256.4 (79; 899.5)<br>n=13			
**Acetate at week 10**	303.0 (217.5)<br>235.7 (48.2; 1117.7)<br>n=32	252.9 (160.7)<br>195.3 (48.2; 574.1)<br>n=16	353.2 (258.0)<br>313.3 (50.9; 1117.7)<br>n=16	303.2 (215.0)<br>215.2 (46.6; 750.5)<br>n=14			

					Difference and test between groups		
Variable	High+Low dose (n=34)	High dose (n=18)	Low dose (n=16)	Placebo (n=16)	High+Low dose vs Placebo	High dose vs Placebo	Low dose vs Placebo
**Relative change in Acetate from randomization to week 1**	49.5 (109.1)<br>5.7 (-81.5; 344.6)<br>n=31	44.8 (123.7)<br>-6 (-81.5; 339.6)<br>n=15	53.9 (97.3)<br>37.8 (-57.8; 344.6)<br>n=16	24.4 (68.5)<br>4.4 (-64.6; 188.6)<br>n=13	25.1 (-36.2; 93.3)	20.5 (-56.3; 101.3)	29.5 (-33.4; 96.8)
**Relative change in Acetate from randomization to week 4**	45.4 (117.3)<br>25.7 (-54.8; 522.3)<br>n=31	40.6 (92.5)<br>12.7 (-54.8; 268.4)<br>n=15	50.0 (139.6)<br>31.8 (-54.3; 522.3)<br>n=16	16.1 (77.6)<br>-6.4 (-83.4; 224.3)<br>n=14	29.3 (-31.5; 103.2)<br>p=0.43	24.5 (-39.6; 90.9)<br>p=0.45	33.9 (-42.7; 120.2)<br>p=0.49
**Relative change in Acetate from randomization to week 8**	36.6 (94.4)<br>19.3 (-69.2; 296.7)<br>n=30	25.0 (100.1)<br>-7 (-69.2; 296.7)<br>n=14	46.8 (91.1)<br>26.9 (-62.9; 262.9)<br>n=16	29.6 (87.9)<br>21.3 (-83; 248)<br>n=13	7.00 (-52.71; 70.18)<br>p=0.85	-4.64 (-78.46; 69.71)<br>p=0.90	17.2 (-50.2; 86.1)<br>p=0.61
**Relative change in Acetate from randomization to week 10**	54.0 (105.5)<br>35.7 (-84.3; 380.3)<br>n=32	25.1 (66.3)<br>14.9 (-56.5; 144.8)<br>n=16	83.0 (129.8)<br>45.6 (-84.3; 380.3)<br>n=16	-12.0 (38.2)<br>-17.8 (-62.9; 38.6)<br>n=14	66.0 (12.8; 127.6)<br>p=0.0094	37.1 (-3.6; 78.7)<br>p=0.072	95.0 (25.4; 169.2)<br>p=0.0042
**Lactate at randomization**	0.458 (0.418)<br>0.262 (0.106; 1.419)<br>n=32	0.454 (0.429)<br>0.22 (0.118; 1.367)<br>n=16	0.461 (0.421)<br>0.308 (0.106; 1.419)<br>n=16	0.980 (1.446)<br>0.494 (0.098; 5.712)<br>n=14			
**Lactate at week 1**	1.01 (0.88)<br>0.66 (0.17; 3.91)<br>n=31	0.880 (0.602)<br>0.811 (0.201; 2.239)<br>n=15	1.12 (1.09)<br>0.65 (0.17; 3.91)<br>n=16	2.24 (4.43)<br>0.95 (0.16; 16.67)<br>n=13			
**Lactate at week 4**	1.38 (1.77)<br>0.97 (0.1; 9.74)<br>n=30	0.986 (0.775)<br>0.853 (0.101; 3.039)<br>n=15	1.77 (2.36)<br>1.03 (0.1; 9.74)<br>n=15	1.48 (2.78)<br>0.65 (0.22; 11.03)<br>n=14			
**Lactate at week 8**	0.937 (1.314)<br>0.327 (0.127; 6.35)<br>n=30	0.846 (1.092)<br>0.293 (0.127; 3.997)<br>n=14	1.02 (1.51)<br>0.46 (0.18; 6.35)<br>n=16	1.87 (4.33)<br>0.75 (0.1; 16.17)<br>n=13			
**Lactate at week 10**	0.976 (0.679)<br>0.782 (0.081; 2.667)<br>n=31	0.983 (0.681)<br>0.929 (0.081; 2.18)<br>n=16	0.969 (0.701)<br>0.73 (0.247; 2.667)<br>n=15	1.92 (2.85)<br>1.22 (0.14; 11.51)<br>n=14			
**Relative change in Lactate from randomization to week 1**	333.6 (449.3)<br>182.2 (-85.5; 1656.8)<br>n=31	337.3 (487.5)<br>182.2 (-71.3; 1656.8)<br>n=15	330.1 (426.5)<br>180.8 (-85.5; 1295.9)<br>n=16	248.8 (354.2)<br>172.8 (-85.5; 910.4)<br>n=13	84.8 (-181.2; 376.4)	88.4 (-235.0; 422.0)	81.3 (-217.8; 391.5)
**Relative change in Lactate from randomization to week 4**	447.5 (773.3)<br>252.5 (-87.2; 3082.5)<br>n=30	297.5 (404.6)<br>272.8 (-87.2; 1426.7)<br>n=15	598 (1013)<br>232 (-83; 3083)<br>n=15	198.5 (371.8)<br>74.1 (-73.9; 1352.2)<br>n=14	249.0 (-114.4; 718.5)<br>p=0.28	98.9 (-194.6; 392.4)<br>p=0.51	399.1 (-145.0; 991.8)<br>p=0.23

Variable	High+Low dose (n=34)	High dose (n=18)	Low dose (n=16)	Placebo (n=16)	Difference and test between groups: High+Low dose vs Placebo	High dose vs Placebo	Low dose vs Placebo
**Relative change in Lactate from randomization to week 8**	124.0 (353.6)<br>36.5 (-47.6; 1908.3)<br>n=30	53.1 (124.7)<br>20.3 (-47.6; 378.1)<br>n=14	186.0 (468.5)<br>48 (-31.2; 1908.3)<br>n=16	137.1 (364.7)<br>-2.8 (-97.3; 1301.3)<br>n=13	-13.1 (-192.0; 257.7)<br>p=0.91	-83.9 (-281.3; 93.9)<br>p=0.59	48.9 (-225.2; 375.2)<br>p=0.71
**Relative change in Lactate from randomization to week 10**	338.4 (388.5)<br>235.1 (-93.9; 1395.9)<br>n=31	326.9 (343.4)<br>321.1 (-93.9; 881.7)<br>n=16	350.6 (443.6)<br>173.3 (-79.3; 1395.9)<br>n=15	342.6 (447.8)<br>185.6 (-79.3; 1346.3)<br>n=14	-4.22 (-264.06; 266.87)<br>p=0.96	-15.7 (-314.3; 281.7)<br>p=0.90	8.04 (-326.28; 350.10)<br>p=0.96
**Isobutyrate at randomization**	10.5 (4.7)<br>9.8 (2.8; 20.6)<br>n=32	10.0 (4.8)<br>9.7 (2.8; 20.6)<br>n=16	11.1 (4.7)<br>9.8 (4.6; 19)<br>n=16	10.4 (6.0)<br>9.3 (2.8; 27.7)<br>n=14			
**Isobutyrate at week 1**	12.8 (8.3)<br>9.6 (3.8; 38.4)<br>n=31	12.9 (8.9)<br>9.7 (4.4; 38.4)<br>n=15	12.7 (8.0)<br>9.6 (3.8; 32.8)<br>n=16	10.5 (4.1)<br>9.5 (3.6; 18.8)<br>n=13			
**Isobutyrate at week 4**	12.6 (5.8)<br>12 (2.8; 24.2)<br>n=31	13.3 (6.0)<br>12.5 (2.8; 22.9)<br>n=15	12.0 (5.7)<br>10.5 (3.4; 24.2)<br>n=16	11.7 (5.1)<br>11.7 (1.9; 21.7)<br>n=14			
**Isobutyrate at week 8**	11.0 (5.0)<br>10.3 (3.3; 23)<br>n=30	11.4 (6.2)<br>9.4 (3.3; 23)<br>n=14	10.6 (3.7)<br>10.8 (4.9; 17.4)<br>n=16	10.5 (5.7)<br>6.8 (3.4; 19.6)<br>n=13			
**Isobutyrate at week 10**	11.6 (5.9)<br>10.5 (2.6; 28.1)<br>n=32	11.0 (5.1)<br>10.8 (4.2; 22.5)<br>n=16	12.3 (6.7)<br>10.1 (2.6; 28.1)<br>n=16	9.07 (3.58)<br>9.17 (3.24; 16.5)<br>n=14			
**Relative change in Isobutyrate from randomization to week 1**	32.1 (81.8)<br>17 (-52.6; 320.8)<br>n=31	47.8 (100.2)<br>22.6 (-52.6; 320.8)<br>n=15	17.5 (59.3)<br>7.5 (-49.7; 143.5)<br>n=16	22.5 (57.7)<br>18.9 (-63.3; 135.7)<br>n=13	9.63 (-37.21; 61.72)	25.3 (-37.2; 89.9)	-5.05 (-49.79; 40.77)
**Relative change in Isobutyrate from randomization to week 4**	30.7 (60.0)<br>14.7 (-44.1; 188)<br>n=31	48.8 (67.9)<br>32.3 (-41.1; 188)<br>n=15	13.7 (47.4)<br>-6.3 (-44.1; 110.1)<br>n=16	55.1 (133.4)<br>18.9 (-78.7; 439)<br>n=14	-24.4 (-79.0; 37.2)<br>p=0.42	-6.29 (-85.34; 69.62)<br>p=0.88	-41.4 (-111.8; 28.2)<br>p=0.30
**Relative change in Isobutyrate from randomization to week 8**	21.3 (70.2)<br>-3.9 (-60.1; 189.2)<br>n=30	36.6 (86.9)<br>17.1 (-55.1; 189.2)<br>n=14	7.95 (50.78)<br>-4.9 (-60.11; 100.62)<br>n=16	17.4 (61.1)<br>-4.1 (-71.7; 142.8)<br>n=13	3.89 (-39.85; 50.09)<br>p=0.89	19.2 (-40.5; 79.4)<br>p=0.51	-9.50 (-51.91; 32.82)<br>p=0.64
**Relative change in Isobutyrate from randomization to week 10**	32.2 (83.4)<br>19.6 (-85; 268.1)<br>n=32	33.9 (85.0)<br>18.6 (-62.1; 250.6)<br>n=16	30.4 (84.6)<br>29.9 (-85; 268.1)<br>n=16	19.5 (116.3)<br>-13.6 (-66.9; 399.4)<br>n=14	12.6 (-44.0; 77.2)<br>p=0.72	14.4 (-59.5; 86.6)<br>p=0.72	10.9 (-61.9; 83.3)<br>p=0.78
**Isovalerate at randomization**	8.26 (3.50)<br>8.37 (2.51; 14.79)<br>n=32	7.96 (3.65)<br>8.12 (2.51; 14.27)<br>n=16	8.56 (3.43)<br>8.47 (3.65; 14.79)<br>n=16	7.61 (4.98)<br>6.88 (1.61; 23)<br>n=14			

					Difference and test between groups		
Variable	High+Low dose (n=34)	High dose (n=18)	Low dose (n=16)	Placebo (n=16)	High+Low dose vs Placebo	High dose vs Placebo	Low dose vs Placebo
**Isovalerate at week 1**	9.77 (6.32)<br>7.21 (2.78; 30.61)<br>n=31	10.1 (7.4)<br>7 (3; 30.6)<br>n=15	9.47 (5.40)<br>8 (2.78; 20.58)<br>n=16	8.00 (3.01)<br>7.4 (3.05; 13.62)<br>n=13			
**Isovalerate at week 4**	9.96 (4.61)<br>8.39 (2.46; 18.32)<br>n=31	10.7 (4.9)<br>9.7 (2.5; 18.2)<br>n=15	9.23 (4.35)<br>8.23 (3.29; 18.32)<br>n=16	8.98 (3.61)<br>9.06 (1.83; 16.35)<br>n=14			
**Isovalerate at week 8**	8.46 (3.83)<br>8.19 (2.09; 17.95)<br>n=30	9.04 (4.62)<br>7.98 (2.09; 17.95)<br>n=14	7.95 (3.04)<br>8.19 (3.04; 12.93)<br>n=16	7.78 (3.86)<br>6.37 (2.7; 13.56)<br>n=13			
**Isovalerate at week 10**	9.05 (4.83)<br>7.75 (2.46; 20.18)<br>n=32	8.70 (4.42)<br>8.08 (2.99; 18.59)<br>n=16	9.40 (5.34)<br>7.55 (2.46; 20.18)<br>n=16	7.16 (3.08)<br>6.65 (2.52; 13.48)<br>n=14			
**Relative change in Isovalerate from randomization to week 1**	27.5 (76.9)<br>10.1 (-54.5; 292.5)<br>n=31	41.6 (94.2)<br>13.4 (-54.5; 292.5)<br>n=15	14.3 (56.4)<br>-11.9 (-40.6; 140.9)<br>n=16	28.7 (64.1)<br>20.5 (-62.7; 172)<br>n=13	-1.23 (-46.99; 49.60)	12.9 (-49.0; 76.4)	-14.5 (-60.5; 32.1)
**Relative change in Isovalerate from randomization to week 4**	31.4 (61.3)<br>15.5 (-44.1; 194)<br>n=31	50.6 (69.3)<br>20.2 (-38.7; 194)<br>n=15	13.3 (48.0)<br>-5.4 (-44.1; 125)<br>n=16	79.0 (176.9)<br>37 (-74.7; 601.5)<br>n=14	-47.6 (-113.4; 29.0)<br>p=0.23	-28.4 (-126.5; 65.3)<br>p=0.62	-65.7 (-156.2; 21.2)<br>p=0.19
**Relative change in Isovalerate from randomization to week 8**	17.9 (66.3)<br>-7.3 (-57.9; 190)<br>n=30	33.3 (76.3)<br>19.6 (-57.9; 190)<br>n=14	4.34 (55.13)<br>-15.9 (-57.63; 143.87)<br>n=16	28.9 (92.8)<br>14.2 (-70.4; 295.3)<br>n=13	-11.1 (-59.1; 40.8)<br>p=0.64	4.39 (-61.43; 69.54)<br>p=0.90	-24.6 (-80.9; 30.5)<br>p=0.41
**Relative change in Isovalerate from randomization to week 10**	29.0 (84.0)<br>10.7 (-81.8; 280.2)<br>n=32	31.3 (90.3)<br>-0.8 (-63.4; 280.2)<br>n=16	26.8 (80.2)<br>17.9 (-81.8; 228.7)<br>n=16	47.9 (184.0)<br>-7.4 (-71.5; 660)<br>n=14	-18.9 (-89.3; 64.2)<br>p=0.61	-16.6 (-115.9; 78.8)<br>p=0.81	-21.2 (-117.8; 70.0)<br>p=0.79
**Succinate at randomization**	3.39 (14.19)<br>0.78 (0.15; 81.05)<br>n=32	0.911 (0.871)<br>0.681 (0.189; 3.104)<br>n=16	5.86 (20.06)<br>0.91 (0.15; 81.05)<br>n=16	28.4 (89.8)<br>0.9 (0.2; 337.3)<br>n=14			
**Succinate at week 1**	0.902 (0.587)<br>0.803 (0.159; 2.455)<br>n=31	0.912 (0.569)<br>0.814 (0.159; 2.455)<br>n=15	0.894 (0.621)<br>0.619 (0.218; 2.153)<br>n=16	1.55 (3.01)<br>0.67 (0.18; 11.36)<br>n=13			
**Succinate at week 4**	0.790 (0.881)<br>0.496 (0.099; 4.782)<br>n=31	0.869 (1.150)<br>0.496 (0.244; 4.782)<br>n=15	0.716 (0.554)<br>0.484 (0.099; 2.06)<br>n=16	0.519 (0.267)<br>0.441 (0.196; 1.067)<br>n=14			

					Difference and test between groups		
**Variable**	**High+Low dose (n=34)**	**High dose (n=18)**	**Low dose (n=16)**	**Placebo (n=16)**	**High+Low dose vs Placebo**	**High dose vs Placebo**	**Low dose vs Placebo**
**Succinate at week 8**	4.73 (17.48)<br>0.85 (0.16; 95.34)<br>n=30	2.29 (5.13)<br>0.85 (0.3; 20.04)<br>n=14	6.87 (23.61)<br>0.84 (0.16; 95.34)<br>n=16	0.717 (0.347)<br>0.735 (0.225; 1.286)<br>n=13			
**Succinate at week 10**	0.803 (0.615)<br>0.627 (0.146; 2.726)<br>n=32	0.787 (0.585)<br>0.644 (0.159; 2.361)<br>n=16	0.819 (0.663)<br>0.627 (0.146; 2.726)<br>n=16	1.06 (1.49)<br>0.66 (0.1; 6.01)<br>n=14			
**Relative change in Succinate from randomization to week 1**	72.2 (195.3)<br>4.5 (-99.3; 856)<br>n=31	58.5 (116.1)<br>18.3 (-66.4; 322.6)<br>n=15	85.0 (251.7)<br>-25.3 (-99.3; 856)<br>n=16	1.09 (99.25)<br>-25.94 (-96.63; 258.02)<br>n=13	71.1 (-26.5; 193.9)	57.5 (-29.1; 141.6)	84.0 (-51.7; 235.8)
**Relative change in Succinate from randomization to week 4**	11.8 (103.9)<br>-29.9 (-99; 384.3)<br>n=31	33.7 (130.9)<br>-29.9 (-77.6; 384.3)<br>n=15	-8.76 (68.37)<br>-26.06 (-98.95; 121.1)<br>n=16	-34.6 (77.8)<br>-59.8 (-99.8; 209.1)<br>n=14	46.4 (-11.1; 112.2)<br>p=0.12	68.3 (-12.4; 150.7)<br>p=0.099	25.8 (-28.5; 79.3)<br>p=0.35
**Relative change in Succinate from randomization to week 8**	260.4 (962.3)<br>15 (-99.4; 4988.2)<br>n=30	164.0 (512.6)<br>13.2 (-51.5; 1928.7)<br>n=14	344.8 (1243.5)<br>15 (-99.4; 4988.2)<br>n=16	-19.6 (42.1)<br>-11.2 (-99.8; 40.5)<br>n=13	280.1 (8.2; 854.5)<br>p=0.023	183.6 (7.9; 443.2)<br>p=0.026	364.4 (-1.2; 1016.1)<br>p=0.055
**Relative change in Succinate from randomization to week 10**	26.1 (152.9)<br>-15.2 (-98.3; 720.2)<br>n=32	24.6 (105.3)<br>-15.2 (-75.3; 334.4)<br>n=16	27.6 (193.0)<br>-23.8 (-98.3; 720.2)<br>n=16	-9.19 (99.21)<br>-57.04 (-99.86; 216.73)<br>n=14	35.3 (-37.7; 130.4)<br>p=0.48	33.8 (-40.2; 109.3)<br>p=0.38	36.8 (-60.9; 149.9)<br>p=0.66

For continuous variables Mean (SD) / Median (Min; Max) / n= is presented.
For comparison between groups the Fisher´s Non Parametric Permutation Test was used for continuous variables. The confidence interval for then mean difference between groups is based on Fishers non-parametric permutation test.
2020-01-01 Analys.sas

Table 14.2.5.2 Secondary Analysis - Relative change in short-chain fatty acids in stool (PP Population)

					Difference and test between groups		
**Variable**	**High+Low dose (n=33)**	**High dose (n=18)**	**Low dose (n=15)**	**Placebo (n=16)**	**High+Low dose vs Placebo**	**High dose vs Placebo**	**Low dose vs Placebo**
**Butyrate at randomization**	54.6 (43.8)<br>40.7 (6.2; 221.3)<br>n=31	56.7 (52.5)<br>33.2 (6.2; 221.3)<br>n=16	52.4 (33.9)<br>42.5 (11.7; 122.7)<br>n=15	85.3 (60.9)<br>70.1 (23.8; 241.4)<br>n=14			
**Butyrate at week 1**	77.0 (68.1)<br>65.8 (5.8; 363.2)<br>n=30	62.2 (39.1)<br>62.8 (11.1; 166.5)<br>n=15	91.8 (87.2)<br>82.8 (5.8; 363.2)<br>n=15	111.0 (84.5)<br>80.1 (8.5; 280.5)<br>n=13			
**Butyrate at week 4**	77.8 (67.5)<br>61.3 (5; 305.4)<br>n=30	62.1 (44.4)<br>54 (5; 152.4)<br>n=15	93.5 (83.3)<br>63 (14.7; 305.4)<br>n=15	112.1 (91.5)<br>96.9 (13.5; 354.7)<br>n=14			
**Butyrate at week 8**	65.8 (56.6)<br>52.8 (14.7; 273.6)<br>n=29	42.8 (29.7)<br>32.9 (14.7; 113.5)<br>n=14	87.3 (67.6)<br>61.7 (36.1; 273.6)<br>n=15	119.9 (141.9)<br>66.6 (13.2; 488.4)<br>n=13			
**Butyrate at week 10**	81.7 (73.3)<br>68.7 (9.7; 389.5)<br>n=31	62.4 (48.6)<br>46.1 (9.7; 185.5)<br>n=16	102.2 (90.0)<br>87.1 (12.7; 389.5)<br>n=15	90.0 (80.5)<br>46 (6; 245)<br>n=14			
**Relative change in Butyrate from randomization to week 1**	79.4 (131.1)<br>16.3 (-74.2; 428.1)<br>n=30	70.5 (144.9)<br>12.8 (-74.2; 428.1)<br>n=15	88.2 (120.1)<br>92 (-64.9; 290.2)<br>n=15	66.7 (128.3)<br>51.8 (-77.5; 433.6)<br>n=13	12.7 (-70.1; 102.5)	3.84 (-101.11; 111.03)	21.6 (-73.5; 115.8)
**Relative change in Butyrate from randomization to week 4**	84.0 (162.9)<br>20.7 (-83.8; 522.3)<br>n=30	53.3 (144.2)<br>18 (-83.8; 396.5)<br>n=15	114.7 (179.3)<br>76.6 (-58.2; 522.3)<br>n=15	71.0 (144.7)<br>20.9 (-75.6; 433)<br>n=14	13.0 (-85.9; 121.2)<br>p=0.82	-17.7 (-127.7; 93.4)<br>p=0.75	43.7 (-80.8; 170.2)<br>p=0.48
**Relative change in Butyrate from randomization to week 8**	133.2 (436.5)<br>2 (-75.3; 2239.4)<br>n=29	32.8 (127.1)<br>-22.2 (-75.3; 309.2)<br>n=14	226.9 (588.7)<br>19 (-58.6; 2239.4)<br>n=15	53.4 (131.3)<br>-0.1 (-81.8; 351.8)<br>n=13	79.8 (-82.0; 341.4)<br>p=0.70	-20.6 (-123.7; 81.1)<br>p=0.68	173.5 (-76.1; 500.3)<br>p=0.38
**Relative change in Butyrate from randomization to week 10**	175.2 (585.3)<br>35.6 (-86.3; 3229.9)<br>n=31	68.7 (173.7)<br>12.2 (-70; 605.3)<br>n=16	288.8 (821.5)<br>67.5 (-86.3; 3229.9)<br>n=15	2.84 (58.79)<br>-3.07 (-84.25; 120.27)<br>n=14	172.4 (1.6; 495.6)<br>p=0.046	65.9 (-23.3; 163.9)<br>p=0.18	285.9 (14.2; 680.5)<br>p=0.019
**Propionate at randomization**	84.4 (75.9)<br>63.9 (10.7; 393.5)<br>n=31	76.2 (53.2)<br>64.5 (10.7; 190)<br>n=16	93.2 (95.7)<br>52.7 (17.2; 393.5)<br>n=15	155.2 (174.8)<br>79.5 (27.9; 651.5)<br>n=14			
**Propionate at week 1**	105.6 (96.0)<br>82.8 (22; 458.2)<br>n=30	98.9 (85.7)<br>79.2 (22; 381.9)<br>n=15	112.3 (107.8)<br>90.5 (22.9; 458.2)<br>n=15	119.8 (90.4)<br>97.4 (22.4; 343.5)<br>n=13			

					Difference and test between groups		
**Variable**	**High+Low dose (n=33)**	**High dose (n=18)**	**Low dose (n=15)**	**Placebo (n=16)**	**High+Low dose vs Placebo**	**High dose vs Placebo**	**Low dose vs Placebo**
**Propionate at week 4**	96.5 (80.9)<br>76.8 (13.9; 368.6)<br>n=30	80.4 (44.0)<br>77.3 (18.3; 161.9)<br>n=15	112.6 (105.2)<br>76.4 (13.9; 368.6)<br>n=15	125.1 (112.5)<br>77.4 (11.3; 462.8)<br>n=14			
**Propionate at week 8**	78.7 (51.7)<br>56 (25.9; 210.4)<br>n=29	70.3 (47.0)<br>48.5 (25.9; 175.7)<br>n=14	86.6 (56.2)<br>75.7 (30.3; 210.4)<br>n=15	134.6 (133.1)<br>79 (15.3; 447.2)<br>n=13			
**Propionate at week 10**	93.0 (56.3)<br>88.1 (12.5; 246.5)<br>n=31	82.6 (48.3)<br>76.9 (12.5; 199.2)<br>n=16	104.2 (63.6)<br>92.2 (16.7; 246.5)<br>n=15	112.3 (103.2)<br>57.5 (23.7; 323.7)<br>n=14			
**Relative change in Propionate from randomization to week 1**	58.8 (123.5)<br>3.2 (-66.9; 486.2)<br>n=30	73.1 (152.9)<br>1.9 (-44.5; 486.2)<br>n=15	44.5 (88.2)<br>16.5 (-66.9; 257.5)<br>n=15	24.7 (70.5)<br>0.9 (-66.4; 143.3)<br>n=13	34.1 (-32.8; 111.5)	48.4 (-41.6; 143.4)	19.8 (-41.8; 83.0)
**Relative change in Propionate from randomization to week 4**	49.4 (145.6)<br>-3.5 (-61.3; 677.5)<br>n=30	39.6 (100.5)<br>2.5 (-61.3; 250)<br>n=15	59.2 (183.3)<br>-7.2 (-43.5; 677.5)<br>n=15	21.1 (85.0)<br>-15.8 (-80.6; 184.1)<br>n=14	28.3 (-43.3; 118.5)<br>p=0.56	18.5 (-51.9; 91.2)<br>p=0.60	38.1 (-57.6; 145.4)<br>p=0.60
**Relative change in Propionate from randomization to week 8**	34.4 (112.8)<br>1 (-75.9; 413)<br>n=29	36.7 (138.1)<br>-21.1 (-74.3; 413)<br>n=14	32.3 (87.8)<br>3.8 (-75.9; 228.3)<br>n=15	41.0 (118.3)<br>33.1 (-87.9; 375.5)<br>n=13	-6.61 (-79.31; 75.36)<br>p=0.83	-4.32 (-104.65; 97.32)<br>p=0.94	-8.75 (-87.31; 70.61)<br>p=0.82
**Relative change in Propionate from randomization to week 10**	56.4 (133.3)<br>12.5 (-83.7; 615.3)<br>n=31	34.9 (81.2)<br>21 (-61.7; 256.6)<br>n=16	79.3 (173.0)<br>12.5 (-83.7; 615.3)<br>n=15	-12.6 (38.7)<br>-13.7 (-64.5; 57.5)<br>n=14	69.0 (8.3; 146.7)<br>p=0.020	47.5 (1.2; 95.9)<br>p=0.044	91.9 (6.4; 185.9)<br>p=0.032
**Acetate at randomization**	225.8 (120.9)<br>212.7 (56.8; 597.7)<br>n=31	222.0 (130.2)<br>203.4 (56.8; 597.7)<br>n=16	229.9 (114.5)<br>212.7 (71.2; 485.5)<br>n=15	352.6 (213.3)<br>293.3 (98; 719.3)<br>n=14			
**Acetate at week 1**	281.1 (206.1)<br>251.8 (20.5; 1101.6)<br>n=30	245.4 (156.6)<br>231.1 (20.5; 683.1)<br>n=15	316.9 (246.4)<br>264.7 (88.1; 1101.6)<br>n=15	347.5 (190.4)<br>314 (79.4; 655.3)<br>n=13			
**Acetate at week 4**	268.4 (179.6)<br>219.9 (48.9; 904.9)<br>n=30	252.8 (121.9)<br>256.9 (57; 525)<br>n=15	283.9 (226.9)<br>207.7 (48.9; 904.9)<br>n=15	343.4 (207.7)<br>317.7 (38.9; 773.6)<br>n=14			
**Acetate at week 8**	256.3 (183.1)<br>180.3 (87.2; 771.9)<br>n=29	223.0 (165.0)<br>142.3 (92.1; 655)<br>n=14	287.3 (199.1)<br>223.7 (87.2; 771.9)<br>n=15	369.5 (288.9)<br>256.4 (79; 899.5)<br>n=13			
**Acetate at week 10**	306.6 (220.1)<br>241.6 (48.2; 1117.7)<br>n=31	252.9 (160.7)<br>195.3 (48.2; 574.1)<br>n=16	363.8 (263.4)<br>330 (50.9; 1117.7)<br>n=15	303.2 (215.0)<br>215.2 (46.6; 750.5)<br>n=14			

					Difference and test between groups		
Variable	High+Low dose (n=33)	High dose (n=18)	Low dose (n=15)	Placebo (n=16)	High+Low dose vs Placebo	High dose vs Placebo	Low dose vs Placebo
**Relative change in Acetate from randomization to week 1**	48.1 (110.7)<br>4.3 (-81.5; 344.6)<br>n=30	44.8 (123.7)<br>-6 (-81.5; 339.6)<br>n=15	51.4 (100.3)<br>26.1 (-57.8; 344.6)<br>n=15	24.4 (68.5)<br>4.4 (-64.6; 188.6)<br>n=13	23.8 (-38.4; 92.5)	20.5 (-56.3; 101.3)	27.1 (-37.0; 95.1)
**Relative change in Acetate from randomization to week 4**	43.2 (118.6)<br>19.2 (-54.8; 522.3)<br>n=30	40.6 (92.5)<br>12.7 (-54.8; 268.4)<br>n=15	45.7 (143.4)<br>28.8 (-54.3; 522.3)<br>n=15	16.1 (77.6)<br>-6.4 (-83.4; 224.3)<br>n=14	27.1 (-34.5; 102.3)<br>p=0.47	24.5 (-39.6; 90.9)<br>p=0.45	29.6 (-50.0; 116.9)<br>p=0.58
**Relative change in Acetate from randomization to week 8**	35.2 (95.8)<br>18.8 (-69.2; 296.7)<br>n=29	25.0 (100.1)<br>-7 (-69.2; 296.7)<br>n=14	44.8 (94.0)<br>21 (-62.9; 262.9)<br>n=15	29.6 (87.9)<br>21.3 (-83; 248)<br>n=13	5.63 (-54.68; 70.66)<br>p=0.88	-4.64 (-78.46; 69.71)<br>p=0.90	15.2 (-55.4; 87.4)<br>p=0.68
**Relative change in Acetate from randomization to week 10**	53.7 (107.3)<br>32.5 (-84.3; 380.3)<br>n=31	25.1 (66.3)<br>14.9 (-56.5; 144.8)<br>n=16	84.2 (134.2)<br>41.4 (-84.3; 380.3)<br>n=15	-12.0 (38.2)<br>-17.8 (-62.9; 38.6)<br>n=14	65.7 (12.2; 128.4)<br>p=0.011	37.1 (-3.6; 78.7)<br>p=0.072	96.2 (22.7; 172.3)<br>p=0.0062
**Lactate at randomization**	0.465 (0.423)<br>0.299 (0.106; 1.419)<br>n=31	0.454 (0.429)<br>0.22 (0.118; 1.367)<br>n=16	0.477 (0.430)<br>0.316 (0.106; 1.419)<br>n=15	0.980 (1.446)<br>0.494 (0.098; 5.712)<br>n=14			
**Lactate at week 1**	1.02 (0.90)<br>0.73 (0.17; 3.91)<br>n=30	0.880 (0.602)<br>0.811 (0.201; 2.239)<br>n=15	1.15 (1.12)<br>0.66 (0.17; 3.91)<br>n=15	2.24 (4.43)<br>0.95 (0.16; 16.67)<br>n=13			
**Lactate at week 4**	1.40 (1.80)<br>1.03 (0.1; 9.74)<br>n=29	0.986 (0.775)<br>0.853 (0.101; 3.039)<br>n=15	1.85 (2.43)<br>1.19 (0.1; 9.74)<br>n=14	1.48 (2.78)<br>0.65 (0.22; 11.03)<br>n=14			
**Lactate at week 8**	0.958 (1.332)<br>0.32 (0.127; 6.35)<br>n=29	0.846 (1.092)<br>0.293 (0.127; 3.997)<br>n=14	1.06 (1.55)<br>0.59 (0.18; 6.35)<br>n=15	1.87 (4.33)<br>0.75 (0.1; 16.17)<br>n=13			
**Lactate at week 10**	0.983 (0.690)<br>0.785 (0.081; 2.667)<br>n=30	0.983 (0.681)<br>0.929 (0.081; 2.18)<br>n=16	0.982 (0.725)<br>0.719 (0.247; 2.667)<br>n=14	1.92 (2.85)<br>1.22 (0.14; 11.51)<br>n=14			
**Relative change in Lactate from randomization to week 1**	338.2 (456.2)<br>174.1 (-85.5; 1656.8)<br>n=30	337.3 (487.5)<br>182.2 (-71.3; 1656.8)<br>n=15	339.1 (439.9)<br>165.9 (-85.5; 1295.9)<br>n=15	248.8 (354.2)<br>172.8 (-85.5; 910.4)<br>n=13	89.3 (-185.1; 385.2)	88.4 (-235.0; 422.0)	90.3 (-217.9; 407.8)
**Relative change in Lactate from randomization to week 4**	455.0 (785.9)<br>272.8 (-87.2; 3082.5)<br>n=29	297.5 (404.6)<br>272.8 (-87.2; 1426.7)<br>n=15	624 (1046)<br>243 (-83; 3083)<br>n=14	198.5 (371.8)<br>74.1 (-73.9; 1352.2)<br>n=14	256.4 (-114.3; 729.7)<br>p=0.28	98.9 (-194.6; 392.4)<br>p=0.51	425.2 (-142.9; 1031.7)<br>p=0.21

					Difference and test between groups		
**Variable**	**High+Low dose (n=33)**	**High dose (n=18)**	**Low dose (n=15)**	**Placebo (n=16)**	**High+Low dose vs Placebo**	**High dose vs Placebo**	**Low dose vs Placebo**
**Relative change in Lactate from randomization to week 8**	126.4 (359.6)<br>35.6 (-47.6; 1908.3)<br>n=29	53.1 (124.7)<br>20.3 (-47.6; 378.1)<br>n=14	194.8 (483.5)<br>42.8 (-31.2; 1908.3)<br>n=15	137.1 (364.7)<br>-2.8 (-97.3; 1301.3)<br>n=13	-10.7 (-196.0; 262.6)<br>p=0.93	-83.9 (-281.3; 93.9)<br>p=0.59	57.7 (-231.8; 386.7)<br>p=0.67
**Relative change in Lactate from randomization to week 10**	341.0 (394.9)<br>232.7 (-93.9; 1395.9)<br>n=30	326.9 (343.4)<br>321.1 (-93.9; 881.7)<br>n=16	357.2 (459.6)<br>140.9 (-79.3; 1395.9)<br>n=14	342.6 (447.8)<br>185.6 (-79.3; 1346.3)<br>n=14	-1.56 (-266.92; 272.76)<br>p=0.98	-15.7 (-314.3; 281.7)<br>p=0.90	14.6 (-339.9; 373.2)<br>p=0.93
**Isobutyrate at randomization**	10.6 (4.8)<br>10.1 (2.8; 20.6)<br>n=31	10.0 (4.8)<br>9.7 (2.8; 20.6)<br>n=16	11.2 (4.8)<br>10.1 (4.6; 19)<br>n=15	10.4 (6.0)<br>9.3 (2.8; 27.7)<br>n=14			
**Isobutyrate at week 1**	12.5 (8.4)<br>9.6 (3.8; 38.4)<br>n=30	12.9 (8.9)<br>9.7 (4.4; 38.4)<br>n=15	12.2 (8.1)<br>9.6 (3.8; 32.8)<br>n=15	10.5 (4.1)<br>9.5 (3.6; 18.8)<br>n=13			
**Isobutyrate at week 4**	12.5 (5.9)<br>12 (2.8; 24.2)<br>n=30	13.3 (6.0)<br>12.5 (2.8; 22.9)<br>n=15	11.7 (5.8)<br>10.1 (3.4; 24.2)<br>n=15	11.7 (5.1)<br>11.7 (1.9; 21.7)<br>n=14			
**Isobutyrate at week 8**	11.1 (5.0)<br>10.6 (3.3; 23)<br>n=29	11.4 (6.2)<br>9.4 (3.3; 23)<br>n=14	10.9 (3.7)<br>11.6 (4.9; 17.4)<br>n=15	10.5 (5.7)<br>6.8 (3.4; 19.6)<br>n=13			
**Isobutyrate at week 10**	11.4 (5.9)<br>10.3 (2.6; 28.1)<br>n=31	11.0 (5.1)<br>10.8 (4.2; 22.5)<br>n=16	11.9 (6.8)<br>9.9 (2.6; 28.1)<br>n=15	9.07 (3.58)<br>9.17 (3.24; 16.5)<br>n=14			
**Relative change in Isobutyrate from randomization to week 1**	29.5 (81.8)<br>14 (-52.6; 320.8)<br>n=30	47.8 (100.2)<br>22.6 (-52.6; 320.8)<br>n=15	11.2 (55.6)<br>3.9 (-49.7; 143.5)<br>n=15	22.5 (57.7)<br>18.9 (-63.3; 135.7)<br>n=13	6.96 (-38.98; 59.71)	25.3 (-37.2; 89.9)	-11.4 (-55.1; 33.1)
**Relative change in Isobutyrate from randomization to week 4**	29.4 (60.6)<br>13.1 (-44.1; 188)<br>n=30	48.8 (67.9)<br>32.3 (-41.1; 188)<br>n=15	10.1 (46.7)<br>-6.5 (-44.1; 110.1)<br>n=15	55.1 (133.4)<br>18.9 (-78.7; 439)<br>n=14	-25.6 (-81.2; 35.9)<br>p=0.40	-6.29 (-85.34; 69.62)<br>p=0.88	-45.0 (-117.8; 25.0)<br>p=0.26
**Relative change in Isobutyrate from randomization to week 8**	23.1 (70.8)<br>-3.8 (-60.1; 189.2)<br>n=29	36.6 (86.9)<br>17.1 (-55.1; 189.2)<br>n=14	10.5 (51.5)<br>-3.9 (-60.1; 100.6)<br>n=15	17.4 (61.1)<br>-4.1 (-71.7; 142.8)<br>n=13	5.64 (-39.21; 52.83)<br>p=0.83	19.2 (-40.5; 79.4)<br>p=0.51	-7.00 (-50.98; 36.37)<br>p=0.75
**Relative change in Isobutyrate from randomization to week 10**	30.1 (83.9)<br>19.4 (-85; 268.1)<br>n=31	33.9 (85.0)<br>18.6 (-62.1; 250.6)<br>n=16	26.0 (85.6)<br>19.4 (-85; 268.1)<br>n=15	19.5 (116.3)<br>-13.6 (-66.9; 399.4)<br>n=14	10.5 (-46.6; 77.3)<br>p=0.78	14.4 (-59.5; 86.6)<br>p=0.72	6.45 (-69.39; 80.21)<br>p=0.87
**Isovalerate at randomization**	8.25 (3.56)<br>8.34 (2.51; 14.79)<br>n=31	7.96 (3.65)<br>8.12 (2.51; 14.27)<br>n=16	8.55 (3.55)<br>8.4 (3.65; 14.79)<br>n=15	7.61 (4.98)<br>6.88 (1.61; 23)<br>n=14			

					Difference and test between groups		
Variable	High+Low dose (n=33)	High dose (n=18)	Low dose (n=15)	Placebo (n=16)	High+Low dose vs Placebo	High dose vs Placebo	Low dose vs Placebo
**Isovalerate at week 1**	9.54 (6.30)<br>7.1 (2.78; 30.61)<br>n=30	10.1 (7.4)<br>7 (3; 30.6)<br>n=15	9.01 (5.25)<br>7.21 (2.78; 20.58)<br>n=15	8.00 (3.01)<br>7.4 (3.05; 13.62)<br>n=13			
**Isovalerate at week 4**	9.85 (4.64)<br>8.38 (2.46; 18.32)<br>n=30	10.7 (4.9)<br>9.7 (2.5; 18.2)<br>n=15	8.96 (4.36)<br>8.21 (3.29; 18.32)<br>n=15	8.98 (3.61)<br>9.06 (1.83; 16.35)<br>n=14			
**Isovalerate at week 8**	8.55 (3.86)<br>8.41 (2.09; 17.95)<br>n=29	9.04 (4.62)<br>7.98 (2.09; 17.95)<br>n=14	8.10 (3.08)<br>8.41 (3.04; 12.93)<br>n=15	7.78 (3.86)<br>6.37 (2.7; 13.56)<br>n=13			
**Isovalerate at week 10**	8.83 (4.75)<br>7.56 (2.46; 20.18)<br>n=31	8.70 (4.42)<br>8.08 (2.99; 18.59)<br>n=16	8.97 (5.22)<br>7.55 (2.46; 20.18)<br>n=15	7.16 (3.08)<br>6.65 (2.52; 13.48)<br>n=14			
**Relative change in Isovalerate from randomization to week 1**	25.4 (77.4)<br>6.9 (-54.5; 292.5)<br>n=30	41.6 (94.2)<br>13.4 (-54.5; 292.5)<br>n=15	9.25 (54.55)<br>-13.51 (-40.59; 140.85)<br>n=15	28.7 (64.1)<br>20.5 (-62.7; 172)<br>n=13	-3.29 (-49.46; 48.27)	12.9 (-49.0; 76.4)	-19.5 (-65.6; 27.0)
**Relative change in Isovalerate from randomization to week 4**	30.6 (62.2)<br>14.2 (-44.1; 194)<br>n=30	50.6 (69.3)<br>20.2 (-38.7; 194)<br>n=15	10.6 (48.4)<br>-10.6 (-44.1; 125)<br>n=15	79.0 (176.9)<br>37 (-74.7; 601.5)<br>n=14	-48.4 (-115.2; 28.7)<br>p=0.24	-28.4 (-126.5; 65.3)<br>p=0.62	-68.4 (-162.2; 19.3)<br>p=0.17
**Relative change in Isovalerate from randomization to week 8**	19.7 (66.7)<br>-1.7 (-57.9; 190)<br>n=29	33.3 (76.3)<br>19.6 (-57.9; 190)<br>n=14	6.95 (56.03)<br>-13.41 (-57.63; 143.87)<br>n=15	28.9 (92.8)<br>14.2 (-70.4; 295.3)<br>n=13	-9.26 (-57.78; 44.56)<br>p=0.69	4.39 (-61.43; 69.54)<br>p=0.90	-22.0 (-79.6; 34.2)<br>p=0.48
**Relative change in Isovalerate from randomization to week 10**	27.3 (84.8)<br>8.7 (-81.8; 280.2)<br>n=31	31.3 (90.3)<br>-0.8 (-63.4; 280.2)<br>n=16	23.0 (81.5)<br>12.6 (-81.8; 228.7)<br>n=15	47.9 (184.0)<br>-7.4 (-71.5; 660)<br>n=14	-20.7 (-92.2; 65.3)<br>p=0.60	-16.6 (-115.9; 78.8)<br>p=0.81	-25.0 (-125.9; 67.6)<br>p=0.76
**Succinate at randomization**	3.47 (14.42)<br>0.8 (0.15; 81.05)<br>n=31	0.911 (0.871)<br>0.681 (0.189; 3.104)<br>n=16	6.21 (20.71)<br>0.91 (0.15; 81.05)<br>n=15	28.4 (89.8)<br>0.9 (0.2; 337.3)<br>n=14			
**Succinate at week 1**	0.922 (0.586)<br>0.809 (0.159; 2.455)<br>n=30	0.912 (0.569)<br>0.814 (0.159; 2.455)<br>n=15	0.933 (0.622)<br>0.637 (0.218; 2.153)<br>n=15	1.55 (3.01)<br>0.67 (0.18; 11.36)<br>n=13			
**Succinate at week 4**	0.807 (0.891)<br>0.499 (0.099; 4.782)<br>n=30	0.869 (1.150)<br>0.496 (0.244; 4.782)<br>n=15	0.745 (0.561)<br>0.503 (0.099; 2.06)<br>n=15	0.519 (0.267)<br>0.441 (0.196; 1.067)<br>n=14			

					Difference and test between groups		
**Variable**	**High+Low dose (n=33)**	**High dose (n=18)**	**Low dose (n=15)**	**Placebo (n=16)**	**High+Low dose vs Placebo**	**High dose vs Placebo**	**Low dose vs Placebo**
**Succinate at week 8**	4.89 (17.77)<br>0.86 (0.16; 95.34)<br>n=29	2.29 (5.13)<br>0.85 (0.3; 20.04)<br>n=14	7.31 (24.37)<br>0.89 (0.16; 95.34)<br>n=15	0.717 (0.347)<br>0.735 (0.225; 1.286)<br>n=13			
**Succinate at week 10**	0.818 (0.619)<br>0.654 (0.146; 2.726)<br>n=31	0.787 (0.585)<br>0.644 (0.159; 2.361)<br>n=16	0.852 (0.673)<br>0.654 (0.146; 2.726)<br>n=15	1.06 (1.49)<br>0.66 (0.1; 6.01)<br>n=14			
**Relative change in Succinate from randomization to week 1**	76.5 (197.1)<br>7 (-99.3; 856)<br>n=30	58.5 (116.1)<br>18.3 (-66.4; 322.6)<br>n=15	94.5 (257.5)<br>-20.7 (-99.3; 856)<br>n=15	1.09 (99.25)<br>-25.94 (-96.63; 258.02)<br>n=13	75.4 (-24.7; 197.0)	57.5 (-29.1; 141.6)	93.4 (-48.1; 246.6)
**Relative change in Succinate from randomization to week 4**	14.2 (104.8)<br>-27.3 (-99; 384.3)<br>n=30	33.7 (130.9)<br>-29.9 (-77.6; 384.3)<br>n=15	-5.30 (69.31)<br>-16.97 (-98.95; 121.1)<br>n=15	-34.6 (77.8)<br>-59.8 (-99.8; 209.1)<br>n=14	48.8 (-10.3; 114.8)<br>p=0.11	68.3 (-12.4; 150.7)<br>p=0.099	29.3 (-26.9; 83.7)<br>p=0.30
**Relative change in Succinate from randomization to week 8**	271.6 (977.4)<br>17 (-99.4; 4988.2)<br>n=29	164.0 (512.6)<br>13.2 (-51.5; 1928.7)<br>n=14	372.0 (1282.2)<br>17 (-99.4; 4988.2)<br>n=15	-19.6 (42.1)<br>-11.2 (-99.8; 40.5)<br>n=13	291.2 (11.6; 872.8)<br>p=0.016	183.6 (7.9; 443.2)<br>p=0.026	391.6 (4.2; 1047.0)<br>p=0.034
**Relative change in Succinate from randomization to week 10**	28.7 (154.7)<br>-14.8 (-98.3; 720.2)<br>n=31	24.6 (105.3)<br>-15.2 (-75.3; 334.4)<br>n=16	33.0 (198.5)<br>-11.4 (-98.3; 720.2)<br>n=15	-9.19 (99.21)<br>-57.04 (-99.86; 216.73)<br>n=14	37.9 (-37.2; 135.9)<br>p=0.45	33.8 (-40.2; 109.3)<br>p=0.38	42.2 (-59.4; 158.3)<br>p=0.60

For continuous variables Mean (SD) / Median (Min; Max) / n= is presented.
For comparison between groups the Fisher´s Non Parametric Permutation Test was used for continuous variables. The confidence interval for then mean difference between groups is based on Fishers non-parametric permutation test.
2020-01-01 Analys.sas

Table 14.2.5.3 Post-hoc Subgroup Analysis - Relative change in short-chain fatty acids in stool (Subgroup population according to the protocol for feces analyses)

					Difference and test between groups		
Variable	High+Low dose (n=30)	High dose (n=16)	Low dose (n=14)	Placebo (n=16)	High+Low dose vs Placebo	High dose vs Placebo	Low dose vs Placebo
**Butyrate at randomization**	57.2 (44.1)<br>42.5 (6.2; 221.3)<br>n=29	59.0 (53.5)<br>34.9 (6.2; 221.3)<br>n=15	55.3 (33.2)<br>43.4 (20.4; 122.7)<br>n=14	85.3 (60.9)<br>70.1 (23.8; 241.4)<br>n=14			
**Butyrate at week 1**	79.8 (69.2)<br>65.8 (11.1; 363.2)<br>n=28	61.7 (40.6)<br>62.7 (11.1; 166.5)<br>n=14	97.9 (87.1)<br>84.6 (23.7; 363.2)<br>n=14	111.0 (84.5)<br>80.1 (8.5; 280.5)<br>n=13			
**Butyrate at week 4**	78.9 (68.9)<br>61.3 (5; 305.4)<br>n=28	59.2 (44.5)<br>50.7 (5; 152.4)<br>n=14	98.6 (84.0)<br>66.5 (14.7; 305.4)<br>n=14	112.1 (91.5)<br>96.9 (13.5; 354.7)<br>n=14			
**Butyrate at week 8**	57.3 (41.2)<br>43.8 (14.7; 183.4)<br>n=27	39.3 (27.8)<br>32.2 (14.7; 113.5)<br>n=13	74.0 (45.3)<br>60.2 (36.1; 183.4)<br>n=14	119.9 (141.9)<br>66.6 (13.2; 488.4)<br>n=13			
**Butyrate at week 10**	70.9 (47.4)<br>60.2 (9.7; 185.5)<br>n=29	60.9 (49.8)<br>43.8 (9.7; 185.5)<br>n=15	81.6 (43.8)<br>79.1 (12.7; 154.3)<br>n=14	90.0 (80.5)<br>46 (6; 245)<br>n=14			
**Relative change in Butyrate from randomization to week 1**	79.0 (130.7)<br>16.3 (-74.2; 428.1)<br>n=28	59.8 (144.0)<br>5.6 (-74.2; 428.1)<br>n=14	98.2 (118.1)<br>103.1 (-64.9; 290.2)<br>n=14	66.7 (128.3)<br>51.8 (-77.5; 433.6)<br>n=13	12.3 (-72.5; 104.8)	-6.92 (-114.30; 101.19)	31.5 (-65.4; 125.8)
**Relative change in Butyrate from randomization to week 4**	73.4 (158.5)<br>18.7 (-83.8; 522.3)<br>n=28	29.9 (116.6)<br>5.8 (-83.8; 396.5)<br>n=14	116.8 (185.9)<br>48 (-58.2; 522.3)<br>n=14	71.0 (144.7)<br>20.9 (-75.6; 433)<br>n=14	2.33 (-95.26; 109.36)<br>p=0.99	-41.1 (-142.1; 60.3)<br>p=0.43	45.8 (-83.8; 176.0)<br>p=0.47
**Relative change in Butyrate from randomization to week 8**	48.7 (161.2)<br>1.1 (-75.3; 728.9)<br>n=27	11.5 (103.1)<br>-28.7 (-75.3; 270)<br>n=13	83.2 (198.7)<br>14.9 (-58.6; 728.9)<br>n=14	53.4 (131.3)<br>-0.1 (-81.8; 351.8)<br>n=13	-4.71 (-93.60; 105.44)<br>p=0.86	-41.9 (-137.5; 54.0)<br>p=0.38	29.8 (-93.2; 161.4)<br>p=0.71
**Relative change in Butyrate from randomization to week 10**	65.6 (144.2)<br>32.2 (-86.3; 605.3)<br>n=29	53.4 (168.3)<br>6.5 (-70; 605.3)<br>n=15	78.7 (117.9)<br>51.5 (-86.3; 390.7)<br>n=14	2.84 (58.79)<br>-3.07 (-84.25; 120.27)<br>n=14	62.8 (-7.9; 146.9)<br>p=0.088	50.6 (-33.0; 143.2)<br>p=0.35	75.8 (5.9; 147.4)<br>p=0.033
**Propionate at randomization**	87.9 (77.3)<br>65.1 (10.7; 393.5)<br>n=29	79.0 (53.9)<br>65.1 (10.7; 190)<br>n=15	97.4 (97.8)<br>60.6 (17.2; 393.5)<br>n=14	155.2 (174.8)<br>79.5 (27.9; 651.5)<br>n=14			
**Propionate at week 1**	109.3 (98.0)<br>85.5 (22; 458.2)<br>n=28	99.9 (88.9)<br>76 (22; 381.9)<br>n=14	118.7 (108.9)<br>92.2 (25.3; 458.2)<br>n=14	119.8 (90.4)<br>97.4 (22.4; 343.5)<br>n=13			

					Difference and test between groups		
**Variable**	**High+Low dose (n=30)**	**High dose (n=16)**	**Low dose (n=14)**	**Placebo (n=16)**	**High+Low dose vs Placebo**	**High dose vs Placebo**	**Low dose vs Placebo**
**Propionate at week 4**	98.8 (83.0)<br>76.8 (13.9; 368.6)<br>n=28	79.4 (45.5)<br>73 (18.3; 161.9)<br>n=14	118.2 (106.9)<br>78.7 (13.9; 368.6)<br>n=14	125.1 (112.5)<br>77.4 (11.3; 462.8)<br>n=14			
**Propionate at week 8**	74.3 (49.7)<br>48.6 (25.9; 210.4)<br>n=27	62.2 (37.3)<br>48.3 (25.9; 142.2)<br>n=13	85.5 (58.1)<br>68.3 (30.3; 210.4)<br>n=14	134.6 (133.1)<br>79 (15.3; 447.2)<br>n=13			
**Propionate at week 10**	87.8 (50.3)<br>85 (12.5; 199.2)<br>n=29	82.1 (49.9)<br>76.5 (12.5; 199.2)<br>n=15	94.0 (51.9)<br>91 (16.7; 194.3)<br>n=14	112.3 (103.2)<br>57.5 (23.7; 323.7)<br>n=14			
**Relative change in Propionate from randomization to week 1**	58.9 (125.6)<br>3.2 (-66.9; 486.2)<br>n=28	67.7 (157.1)<br>-5.7 (-44.5; 486.2)<br>n=14	50.1 (88.8)<br>31.6 (-66.9; 257.5)<br>n=14	24.7 (70.5)<br>0.9 (-66.4; 143.3)<br>n=13	34.2 (-34.4; 113.3)	43.0 (-50.5; 141.0)	25.4 (-38.1; 89.4)
**Relative change in Propionate from randomization to week 4**	46.6 (148.6)<br>-7.8 (-61.3; 677.5)<br>n=28	29.8 (96.7)<br>-3.6 (-61.3; 250)<br>n=14	63.4 (189.5)<br>-7.8 (-43.5; 677.5)<br>n=14	21.1 (85.0)<br>-15.8 (-80.6; 184.1)<br>n=14	25.5 (-47.5; 116.7)<br>p=0.64	8.75 (-60.85; 80.65)<br>p=0.81	42.3 (-56.7; 151.5)<br>p=0.57
**Relative change in Propionate from randomization to week 8**	14.4 (82.2)<br>0.6 (-75.9; 235.2)<br>n=27	7.77 (89.15)<br>-24.93 (-74.25; 235.15)<br>n=13	20.6 (78.1)<br>2.4 (-75.9; 228.3)<br>n=14	41.0 (118.3)<br>33.1 (-87.9; 375.5)<br>n=13	-26.6 (-87.8; 41.2)<br>p=0.41	-33.3 (-116.9; 49.3)<br>p=0.45	-20.4 (-100.5; 55.2)<br>p=0.62
**Relative change in Propionate from randomization to week 10**	33.5 (83.3)<br>9.4 (-83.7; 256.6)<br>n=29	26.5 (76.4)<br>9 (-61.7; 256.6)<br>n=15	41.0 (92.4)<br>10.9 (-83.7; 219.6)<br>n=14	-12.6 (38.7)<br>-13.7 (-64.5; 57.5)<br>n=14	46.1 (1.1; 93.9)<br>p=0.044	39.1 (-5.0; 84.9)<br>p=0.085	53.6 (-2.3; 108.3)<br>p=0.059
**Acetate at randomization**	227.6 (124.6)<br>212.7 (56.8; 597.7)<br>n=29	225.8 (133.9)<br>215.7 (56.8; 597.7)<br>n=15	229.5 (118.8)<br>207.7 (71.2; 485.5)<br>n=14	352.6 (213.3)<br>293.3 (98; 719.3)<br>n=14			
**Acetate at week 1**	284.1 (211.2)<br>251.8 (20.5; 1101.6)<br>n=28	238.0 (159.7)<br>217.3 (20.5; 683.1)<br>n=14	330.2 (250.0)<br>290.6 (88.1; 1101.6)<br>n=14	347.5 (190.4)<br>314 (79.4; 655.3)<br>n=13			
**Acetate at week 4**	273.3 (184.5)<br>219.9 (48.9; 904.9)<br>n=28	252.6 (126.5)<br>245.3 (57; 525)<br>n=14	294.0 (231.9)<br>212.4 (48.9; 904.9)<br>n=14	343.4 (207.7)<br>317.7 (38.9; 773.6)<br>n=14			
**Acetate at week 8**	225.1 (146.4)<br>180.2 (87.2; 771.9)<br>n=27	189.7 (112.9)<br>136.7 (92.1; 422.5)<br>n=13	257.9 (169.4)<br>214 (87.2; 771.9)<br>n=14	369.5 (288.9)<br>256.4 (79; 899.5)<br>n=13			
**Acetate at week 10**	277.0 (165.7)<br>229.8 (48.2; 673.9)<br>n=29	246.3 (164.1)<br>190.9 (48.2; 574.1)<br>n=15	310.0 (166.9)<br>313.3 (50.9; 673.9)<br>n=14	303.2 (215.0)<br>215.2 (46.6; 750.5)<br>n=14			

					Difference and test between groups		
**Variable**	**High+Low dose (n=30)**	**High dose (n=16)**	**Low dose (n=14)**	**Placebo (n=16)**	**High+Low dose vs Placebo**	**High dose vs Placebo**	**Low dose vs Placebo**
**Relative change in Acetate from randomization to week 1**	49.2 (112.6)<br>4.3 (-81.5; 344.6)<br>n=28	40.0 (126.9)<br>-9.9 (-81.5; 339.6)<br>n=14	58.3 (100.3)<br>37.8 (-57.8; 344.6)<br>n=14	24.4 (68.5)<br>4.4 (-64.6; 188.6)<br>n=13	24.8 (-38.6; 95.7)	15.7 (-64.0; 96.8)	34.0 (-32.4; 102.4)
**Relative change in Acetate from randomization to week 4**	45.7 (121.8)<br>19.2 (-54.8; 522.3)<br>n=28	39.5 (95.9)<br>6.2 (-54.8; 268.4)<br>n=14	51.8 (146.7)<br>31.8 (-54.3; 522.3)<br>n=14	16.1 (77.6)<br>-6.4 (-83.4; 224.3)<br>n=14	29.6 (-35.6; 105.4)<br>p=0.45	23.4 (-43.3; 91.9)<br>p=0.49	35.8 (-45.9; 124.7)<br>p=0.51
**Relative change in Acetate from randomization to week 8**	19.6 (77.4)<br>2.7 (-69.2; 262.9)<br>n=27	4.07 (65.05)<br>-16.67 (-69.2; 130.58)<br>n=13	34.0 (87.2)<br>20.4 (-62.9; 262.9)<br>n=14	29.6 (87.9)<br>21.3 (-83; 248)<br>n=13	-10.0 (-62.6; 46.5)<br>p=0.69	-25.5 (-87.7; 37.4)<br>p=0.42	4.36 (-65.67; 72.64)<br>p=0.91
**Relative change in Acetate from randomization to week 10**	40.5 (91.4)<br>32.1 (-84.3; 380.3)<br>n=29	19.2 (64.1)<br>6.9 (-56.5; 144.8)<br>n=15	63.5 (111.7)<br>40.1 (-84.3; 380.3)<br>n=14	-12.0 (38.2)<br>-17.8 (-62.9; 38.6)<br>n=14	52.5 (6.6; 105.4)<br>p=0.022	31.1 (-9.5; 72.0)<br>p=0.13	75.5 (15.2; 138.2)<br>p=0.011
**Lactate at randomization**	0.452 (0.423)<br>0.224 (0.106; 1.419)<br>n=29	0.416 (0.416)<br>0.217 (0.118; 1.367)<br>n=15	0.490 (0.444)<br>0.32 (0.106; 1.419)<br>n=14	0.980 (1.446)<br>0.494 (0.098; 5.712)<br>n=14			
**Lactate at week 1**	1.06 (0.91)<br>0.91 (0.17; 3.91)<br>n=28	0.908 (0.614)<br>0.95 (0.201; 2.239)<br>n=14	1.21 (1.14)<br>0.83 (0.17; 3.91)<br>n=14	2.24 (4.43)<br>0.95 (0.16; 16.67)<br>n=13			
**Lactate at week 4**	1.41 (1.87)<br>0.91 (0.1; 9.74)<br>n=27	0.959 (0.797)<br>0.828 (0.101; 3.039)<br>n=14	1.89 (2.53)<br>1.03 (0.1; 9.74)<br>n=13	1.48 (2.78)<br>0.65 (0.22; 11.03)<br>n=14			
**Lactate at week 8**	0.871 (1.237)<br>0.32 (0.127; 6.35)<br>n=27	0.603 (0.633)<br>0.292 (0.127; 1.822)<br>n=13	1.12 (1.60)<br>0.64 (0.18; 6.35)<br>n=14	1.87 (4.33)<br>0.75 (0.1; 16.17)<br>n=13			
**Lactate at week 10**	0.952 (0.672)<br>0.785 (0.081; 2.667)<br>n=28	1.01 (0.70)<br>1.02 (0.08; 2.18)<br>n=15	0.889 (0.662)<br>0.709 (0.247; 2.667)<br>n=13	1.92 (2.85)<br>1.22 (0.14; 11.51)<br>n=14			
**Relative change in Lactate from randomization to week 1**	363.9 (461.7)<br>197.8 (-85.5; 1656.8)<br>n=28	365.1 (493.4)<br>204.2 (-71.3; 1656.8)<br>n=14	362.7 (446.5)<br>189.6 (-85.5; 1295.9)<br>n=14	248.8 (354.2)<br>172.8 (-85.5; 910.4)<br>n=13	115.1 (-163.8; 412.7)	116.3 (-225.2; 462.8)	113.9 (-209.2; 443.7)
**Relative change in Lactate from randomization to week 4**	474.6 (810.8)<br>272.8 (-87.2; 3082.5)<br>n=27	316.4 (413.0)<br>292.8 (-87.2; 1426.7)<br>n=14	645 (1086)<br>193 (-83; 3083)<br>n=13	198.5 (371.8)<br>74.1 (-73.9; 1352.2)<br>n=14	276.0 (-115.8; 768.6)<br>p=0.25	117.9 (-185.1; 421.8)<br>p=0.44	446.4 (-155.1; 1069.4)<br>p=0.20

					Difference and test between groups		
**Variable**	**High+Low dose (n=30)**	**High dose (n=16)**	**Low dose (n=14)**	**Placebo (n=16)**	**High+Low dose vs Placebo**	**High dose vs Placebo**	**Low dose vs Placebo**
**Relative change in Lactate from randomization to week 8**	125.3 (370.8)<br>35.6 (-47.6; 1908.3)<br>n=27	34.8 (108.6)<br>14.9 (-47.6; 378.1)<br>n=13	209.3 (498.4)<br>54.7 (-31.2; 1908.3)<br>n=14	137.1 (364.7)<br>-2.8 (-97.3; 1301.3)<br>n=13	-11.7 (-204.2; 264.7)<br>p=0.95	-102.2 (-303.7; 70.0)<br>p=0.45	72.3 (-230.0; 406.2)<br>p=0.61
**Relative change in Lactate from randomization to week 10**	344.1 (398.7)<br>232.7 (-93.9; 1395.9)<br>n=28	351.1 (340.9)<br>407 (-93.9; 881.7)<br>n=15	335.9 (471.2)<br>108.5 (-79.3; 1395.9)<br>n=13	342.6 (447.8)<br>185.6 (-79.3; 1346.3)<br>n=14	1.50 (-267.08; 283.33)<br>p=1.00	8.56 (-295.52; 310.03)<br>p=0.96	-6.65 (-377.09; 361.63)<br>p=0.97
**Isobutyrate at randomization**	10.8 (4.9)<br>10.2 (2.8; 20.6)<br>n=29	10.1 (4.9)<br>10.2 (2.8; 20.6)<br>n=15	11.5 (4.9)<br>10.6 (4.6; 19)<br>n=14	10.4 (6.0)<br>9.3 (2.8; 27.7)<br>n=14			
**Isobutyrate at week 1**	12.4 (8.3)<br>9.6 (3.8; 38.4)<br>n=28	12.2 (8.8)<br>9.5 (4.4; 38.4)<br>n=14	12.7 (8.1)<br>9.6 (3.8; 32.8)<br>n=14	10.5 (4.1)<br>9.5 (3.6; 18.8)<br>n=13			
**Isobutyrate at week 4**	12.7 (6.0)<br>12 (2.8; 24.2)<br>n=28	13.3 (6.2)<br>12.3 (2.8; 22.9)<br>n=14	12.1 (5.9)<br>10.5 (3.4; 24.2)<br>n=14	11.7 (5.1)<br>11.7 (1.9; 21.7)<br>n=14			
**Isobutyrate at week 8**	10.6 (4.6)<br>10 (3.3; 23)<br>n=27	10.5 (5.5)<br>8.3 (3.3; 23)<br>n=13	10.7 (3.8)<br>10.8 (4.9; 17.4)<br>n=14	10.5 (5.7)<br>6.8 (3.4; 19.6)<br>n=13			
**Isobutyrate at week 10**	10.5 (4.6)<br>10.2 (2.6; 19.6)<br>n=29	10.2 (4.2)<br>10.7 (4.2; 18.5)<br>n=15	10.7 (5.2)<br>9.5 (2.6; 19.6)<br>n=14	9.07 (3.58)<br>9.17 (3.24; 16.5)<br>n=14			
**Relative change in Isobutyrate from randomization to week 1**	26.3 (78.3)<br>14 (-52.6; 320.8)<br>n=28	38.1 (96.4)<br>19.8 (-52.6; 320.8)<br>n=14	14.5 (56.1)<br>7.5 (-49.7; 143.5)<br>n=14	22.5 (57.7)<br>18.9 (-63.3; 135.7)<br>n=13	3.76 (-40.31; 55.80)	15.6 (-44.5; 79.8)	-8.03 (-53.12; 37.16)
**Relative change in Isobutyrate from randomization to week 4**	29.9 (62.2)<br>13.1 (-44.1; 188)<br>n=28	48.4 (70.5)<br>25.4 (-41.1; 188)<br>n=14	11.3 (48.2)<br>-6.3 (-44.1; 110.1)<br>n=14	55.1 (133.4)<br>18.9 (-78.7; 439)<br>n=14	-25.2 (-83.5; 37.6)<br>p=0.42	-6.70 (-88.78; 72.52)<br>p=0.89	-43.7 (-120.6; 28.5)<br>p=0.29
**Relative change in Isobutyrate from randomization to week 8**	15.6 (65.8)<br>-3.9 (-60.1; 189.2)<br>n=27	25.8 (79.9)<br>16.7 (-55.1; 189.2)<br>n=13	6.16 (50.61)<br>-4.9 (-60.11; 100.62)<br>n=14	17.4 (61.1)<br>-4.1 (-71.7; 142.8)<br>n=13	-1.84 (-44.89; 42.79)<br>p=0.92	8.34 (-48.78; 66.25)<br>p=0.77	-11.3 (-56.4; 33.1)<br>p=0.60
**Relative change in Isobutyrate from randomization to week 10**	16.7 (67.4)<br>17.3 (-85; 250.6)<br>n=29	24.2 (78.2)<br>17.3 (-62.1; 250.6)<br>n=15	8.68 (55.32)<br>12.34 (-84.98; 105.2)<br>n=14	19.5 (116.3)<br>-13.6 (-66.9; 399.4)<br>n=14	-2.82 (-54.48; 56.70)<br>p=0.87	4.67 (-69.14; 74.05)<br>p=0.91	-10.8 (-79.0; 50.1)<br>p=0.85

					Difference and test between groups		
Variable	High+Low dose (n=30)	High dose (n=16)	Low dose (n=14)	Placebo (n=16)	High+Low dose vs Placebo	High dose vs Placebo	Low dose vs Placebo
**Isovalerate at randomization**	8.37 (3.65)<br>8.4 (2.51; 14.79)<br>n=29	8.03 (3.77)<br>8.34 (2.51; 14.27)<br>n=15	8.73 (3.62)<br>8.47 (3.65; 14.79)<br>n=14	7.61 (4.98)<br>6.88 (1.61; 23)<br>n=14			
**Isovalerate at week 1**	9.39 (6.14)<br>7.1 (2.78; 30.61)<br>n=28	9.40 (7.12)<br>6.74 (2.99; 30.61)<br>n=14	9.37 (5.25)<br>8 (2.78; 20.58)<br>n=14	8.00 (3.01)<br>7.4 (3.05; 13.62)<br>n=13			
**Isovalerate at week 4**	10.1 (4.7)<br>8.6 (2.5; 18.3)<br>n=28	10.9 (5.0)<br>10.6 (2.5; 18.2)<br>n=14	9.21 (4.41)<br>8.23 (3.29; 18.32)<br>n=14	8.98 (3.61)<br>9.06 (1.83; 16.35)<br>n=14			
**Isovalerate at week 8**	8.36 (3.83)<br>8.41 (2.09; 17.95)<br>n=27	8.63 (4.54)<br>7.06 (2.09; 17.95)<br>n=13	8.11 (3.20)<br>8.44 (3.04; 12.93)<br>n=14	7.78 (3.86)<br>6.37 (2.7; 13.56)<br>n=13			
**Isovalerate at week 10**	8.10 (3.95)<br>7.56 (2.46; 16.19)<br>n=29	8.04 (3.67)<br>7.94 (2.99; 13.44)<br>n=15	8.17 (4.36)<br>7.22 (2.46; 16.19)<br>n=14	7.16 (3.08)<br>6.65 (2.52; 13.48)<br>n=14			
**Relative change in Isovalerate from randomization to week 1**	22.1 (73.3)<br>6.9 (-54.5; 292.5)<br>n=28	31.7 (89.1)<br>11.7 (-54.5; 292.5)<br>n=14	12.5 (55.1)<br>-11.9 (-40.6; 140.9)<br>n=14	28.7 (64.1)<br>20.5 (-62.7; 172)<br>n=13	-6.65 (-50.96; 43.84)	2.93 (-56.42; 65.56)	-16.2 (-63.4; 30.9)
**Relative change in Isovalerate from randomization to week 4**	32.5 (63.9)<br>14.2 (-44.1; 194)<br>n=28	52.8 (71.4)<br>25.9 (-38.7; 194)<br>n=14	12.1 (49.9)<br>-6.3 (-44.1; 125)<br>n=14	79.0 (176.9)<br>37 (-74.7; 601.5)<br>n=14	-46.5 (-116.4; 31.8)<br>p=0.26	-26.2 (-128.6; 70.2)<br>p=0.67	-66.9 (-165.5; 23.2)<br>p=0.20
**Relative change in Isovalerate from randomization to week 8**	16.2 (67.1)<br>-12.9 (-57.9; 190)<br>n=27	27.9 (76.5)<br>14.2 (-57.9; 190)<br>n=13	5.32 (57.78)<br>-15.9 (-57.63; 143.87)<br>n=14	28.9 (92.8)<br>14.2 (-70.4; 295.3)<br>n=13	-12.8 (-63.0; 41.1)<br>p=0.62	-1.10 (-69.52; 65.79)<br>p=0.97	-23.6 (-83.9; 34.3)<br>p=0.47
**Relative change in Isovalerate from randomization to week 10**	15.5 (73.6)<br>2.2 (-81.8; 280.2)<br>n=29	22.3 (85.7)<br>-3.9 (-63.4; 280.2)<br>n=15	8.27 (60.46)<br>10.66 (-81.77; 114.99)<br>n=14	47.9 (184.0)<br>-7.4 (-71.5; 660)<br>n=14	-32.4 (-101.3; 49.1)<br>p=0.48	-25.6 (-129.2; 68.1)<br>p=0.73	-39.7 (-139.1; 45.8)<br>p=0.63
**Succinate at randomization**	3.67 (14.90)<br>0.8 (0.15; 81.05)<br>n=29	0.948 (0.888)<br>0.744 (0.189; 3.104)<br>n=15	6.58 (21.44)<br>0.91 (0.15; 81.05)<br>n=14	28.4 (89.8)<br>0.9 (0.2; 337.3)<br>n=14			
**Succinate at week 1**	0.906 (0.539)<br>0.809 (0.218; 2.455)<br>n=28	0.966 (0.550)<br>0.818 (0.41; 2.455)<br>n=14	0.845 (0.543)<br>0.619 (0.218; 2.081)<br>n=14	1.55 (3.01)<br>0.67 (0.18; 11.36)<br>n=13			

					Difference and test between groups		
**Variable**	**High+Low dose (n=30)**	**High dose (n=16)**	**Low dose (n=14)**	**Placebo (n=16)**	**High+Low dose vs Placebo**	**High dose vs Placebo**	**Low dose vs Placebo**
**Succinate at week 4**	0.759 (0.890)<br>0.487 (0.099; 4.782)<br>n=28	0.867 (1.193)<br>0.487 (0.244; 4.782)<br>n=14	0.651 (0.443)<br>0.484 (0.099; 1.556)<br>n=14	0.519 (0.267)<br>0.441 (0.196; 1.067)<br>n=14			
**Succinate at week 8**	5.19 (18.40)<br>0.86 (0.16; 95.34)<br>n=27	2.41 (5.32)<br>0.86 (0.3; 20.04)<br>n=13	7.77 (25.22)<br>0.93 (0.16; 95.34)<br>n=14	0.717 (0.347)<br>0.735 (0.225; 1.286)<br>n=13			
**Succinate at week 10**	0.856 (0.622)<br>0.694 (0.146; 2.726)<br>n=29	0.828 (0.580)<br>0.694 (0.209; 2.361)<br>n=15	0.885 (0.686)<br>0.675 (0.146; 2.726)<br>n=14	1.06 (1.49)<br>0.66 (0.1; 6.01)<br>n=14			
**Relative change in Succinate from randomization to week 1**	79.2 (202.5)<br>7 (-99.3; 856)<br>n=28	66.6 (116.1)<br>23.3 (-66.4; 322.6)<br>n=14	91.9 (267.1)<br>-25.3 (-99.3; 856)<br>n=14	1.09 (99.25)<br>-25.94 (-96.63; 258.02)<br>n=13	78.1 (-26.2; 201.8)	65.5 (-19.2; 150.9)	90.8 (-56.8; 251.0)
**Relative change in Succinate from randomization to week 4**	5.19 (102.55)<br>-32.52 (-98.95; 384.25)<br>n=28	24.7 (131.0)<br>-35 (-77.6; 384.3)<br>n=14	-14.3 (62.1)<br>-26.1 (-99; 93.6)<br>n=14	-34.6 (77.8)<br>-59.8 (-99.8; 209.1)<br>n=14	39.8 (-17.0; 105.4)<br>p=0.19	59.3 (-21.6; 143.2)<br>p=0.16	20.3 (-33.5; 72.8)<br>p=0.47
**Relative change in Succinate from randomization to week 8**	286.6 (1012.4)<br>17 (-99.4; 4988.2)<br>n=27	165.7 (533.5)<br>7.6 (-51.5; 1928.7)<br>n=13	398.8 (1326.2)<br>25 (-99.4; 4988.2)<br>n=14	-19.6 (42.1)<br>-11.2 (-99.8; 40.5)<br>n=13	306.2 (8.5; 893.1)<br>p=0.022	185.3 (0.4; 451.5)<br>p=0.048	418.5 (6.3; 1075.5)<br>p=0.031
**Relative change in Succinate from randomization to week 10**	34.5 (158.5)<br>-11.4 (-98.3; 720.2)<br>n=29	29.9 (106.8)<br>-14.8 (-75.3; 334.4)<br>n=15	39.6 (204.3)<br>-7.6 (-98.3; 720.2)<br>n=14	-9.19 (99.21)<br>-57.04 (-99.86; 216.73)<br>n=14	43.7 (-33.7; 142.5)<br>p=0.37	39.0 (-38.5; 119.2)<br>p=0.33	48.8 (-57.0; 166.9)<br>p=0.54

For continuous variables Mean (SD) / Median (Min; Max) / n= is presented.
For comparison between groups the Fisher´s Non Parametric Permutation Test was used for continuous variables. The confidence interval for then mean difference between groups is based on Fishers non-parametric permutation test.
2020-01-01 Analys.sas

Table 14.2.5.4 Posthoc Analyses - Butyrate - MMRM on various populations

		MMRM comparing High+Low dose vs Placebo					MMRM comparing High dose vs Placebo and Low dose vs Placebo							
		High+Low dose		Placebo		High+Low dose vs Placebo	High dose		Low dose		Placebo		High dose vs Placebo	Low dose vs Placebo
Population	Time Point	n	LS mean<br>(95% CI)<br>p-value	n	LS mean<br>(95% CI)<br>p-value	Difference in LS mean<br>(95% CI)<br>p-value	n	LS mean<br>(95% CI)<br>p-value	n	LS mean<br>(95% CI)<br>p-value	n	LS mean<br>(95% CI)<br>p-value	Difference in LS mean<br>(95% CI)<br>p-value	Difference in LS mean<br>(95% CI)<br>p-value
ITT Population with non-missing value at any time point	W4	31	73.4<br>(20.7; 126.0)<br>p=0.0075	14	103.2<br>(25.5; 180.9)<br>p=0.010	-29.8<br>(-125.4; 65.8)<br>p=0.53	15	47.2<br>(-18.7; 113.1)<br>p=0.16	16	99.3<br>(18.5; 180.2)<br>p=0.017	14	101.6<br>(23.0; 180.2)<br>p=0.013	-54.4<br>(-156.4; 47.7)<br>p=0.29	-2.3<br>(-119.1; 114.5)<br>p=0.97
	W8	30	106.1<br>(-36.0; 248.1)<br>p=0.14	13	79.3<br>(-0.5; 159.2)<br>p=0.051	26.7<br>(-133.9; 187.4)<br>p=0.74	14	14.8<br>(-40.0; 69.6)<br>p=0.59	16	196.2<br>(-75.7; 468.1)<br>p=0.15	13	77.6<br>(-1.8; 157.1)<br>p=0.055	-62.8<br>(-156.8; 31.1)<br>p=0.18	118.5<br>(-163.5; 400.6)<br>p=0.40
	W10	32	164.8<br>(-33.0; 362.7)<br>p=0.10	14	35.0<br>(-15.7; 85.7)<br>p=0.17	129.8<br>(-72.7; 332.3)<br>p=0.20	16	60.8<br>(-19.2; 140.7)<br>p=0.13	16	270.2<br>(-111.1; 651.5)<br>p=0.16	14	33.4<br>(-17.2; 84.0)<br>p=0.19	27.4<br>(-66.1; 120.8)<br>p=0.56	236.8<br>(-147.0; 620.6)<br>p=0.22
ITT Population excl outliers with non-missing value at any time point	W4	27	46.1<br>(2.1; 90.1)<br>p=0.041	13	60.8<br>(2.0; 119.6)<br>p=0.043	-14.7<br>(-85.6; 56.1)<br>p=0.68	14	21.6<br>(-26.6; 69.7)<br>p=0.37	13	73.5<br>(-3.8; 150.8)<br>p=0.062	13	60.3<br>(1.0; 119.6)<br>p=0.046	-38.8<br>(-107.1; 29.5)<br>p=0.26	13.2<br>(-87.1; 113.5)<br>p=0.79
	W8	26	14.9<br>(-20.1; 50.0)<br>p=0.39	12	73.7<br>(-5.7; 153.0)<br>p=0.068	-58.7<br>(-145.6; 28.1)<br>p=0.18	13	9.5<br>(-48.2; 67.2)<br>p=0.74	13	22.0<br>(-18.0; 62.0)<br>p=0.27	12	73.3<br>(-6.4; 153.0)<br>p=0.070	-63.8<br>(-159.5; 32.0)<br>p=0.19	-51.3<br>(-143.6; 41.1)<br>p=0.27
	W10	28	41.5<br>(8.4; 74.5)<br>p=0.015	13	23.0<br>(-20.6; 66.5)<br>p=0.29	18.5<br>(-35.7; 72.7)<br>p=0.49	15	27.1<br>(-19.0; 73.3)<br>p=0.24	13	58.5<br>(8.5; 108.5)<br>p=0.023	13	22.5<br>(-21.6; 66.7)<br>p=0.31	4.6<br>(-53.4; 62.6)<br>p=0.87	36.0<br>(-36.9; 108.8)<br>p=0.32
ITT Population with complete cases at all time points	W4	30	78.9<br>(24.6; 133.1)<br>p=0.0055	13	101.0<br>(21.8; 180.2)<br>p=0.014	-22.2<br>(-120.2; 75.9)<br>p=0.65	14	54.5<br>(-16.4; 125.4)<br>p=0.13	16	101.3<br>(20.7; 181.8)<br>p=0.015	13	99.7<br>(19.7; 179.7)<br>p=0.016	-45.3<br>(-152.3; 61.8)<br>p=0.40	1.5<br>(-115.4; 118.5)<br>p=0.98
	W8	30	118.1<br>(-31.9; 268.1)<br>p=0.12	13	76.8<br>(-5.7; 159.2)<br>p=0.067	41.3<br>(-127.7; 210.3)<br>p=0.62	14	27.8<br>(-29.8; 85.3)<br>p=0.33	16	198.1<br>(-73.4; 469.7)<br>p=0.15	13	75.5<br>(-6.6; 157.5)<br>p=0.070	-47.7<br>(-145.5; 50.1)<br>p=0.33	122.7<br>(-159.7; 405.0)<br>p=0.38
	W10	30	181.5<br>(-28.6; 391.6)<br>p=0.089	13	28.0<br>(-23.9; 79.9)<br>p=0.28	153.5<br>(-61.9; 368.9)<br>p=0.16	14	79.0<br>(-9.7; 167.8)<br>p=0.079	16	272.1<br>(-109.1; 653.4)<br>p=0.16	13	26.7<br>(-24.9; 78.3)<br>p=0.30	52.3<br>(-50.1; 154.8)<br>p=0.31	245.4<br>(-138.8; 629.6)<br>p=0.20
ITT Population excl outliers with complete cases at all time points	W4	26	52.0<br>(6.1; 97.9)<br>p=0.027	12	60.7<br>(-0.5; 122.0)<br>p=0.052	-8.7<br>(-82.9; 65.5)<br>p=0.81	13	29.4<br>(-24.0; 82.7)<br>p=0.27	13	74.8<br>(-2.1; 151.8)<br>p=0.056	12	60.6<br>(-1.0; 122.2)<br>p=0.054	-31.2<br>(-105.3; 42.8)<br>p=0.40	14.3<br>(-86.6; 115.1)<br>p=0.78
	W8	26	20.0<br>(-14.7; 54.7)<br>p=0.25	12	73.2<br>(-7.4; 153.7)<br>p=0.074	-53.2<br>(-140.5; 34.2)<br>p=0.22	13	16.8<br>(-42.5; 76.1)<br>p=0.57	13	23.3<br>(-16.0; 62.7)<br>p=0.24	12	73.0<br>(-7.8; 153.9)<br>p=0.075	-56.2<br>(-153.2; 40.7)<br>p=0.25	-49.7<br>(-142.4; 43.0)<br>p=0.28

		MMRM comparing High+Low dose vs Placebo					MMRM comparing High dose vs Placebo and Low dose vs Placebo							
		High+Low dose		Placebo		High+Low dose vs Placebo	High dose		Low dose		Placebo		High dose vs Placebo	Low dose vs Placebo
**Population**	**Time Point**	**n**	**LS mean (95% CI) p-value**	**n**	**LS mean (95% CI) p-value**	**Difference in LS mean (95% CI) p-value**	**n**	**LS mean (95% CI) p-value**	**n**	**LS mean (95% CI) p-value**	**n**	**LS mean (95% CI) p-value**	**Difference in LS mean (95% CI) p-value**	**Difference in LS mean (95% CI) p-value**
	**W10**	26	49.7<br>(15.4; 84.0)<br>p=0.0058	12	20.2<br>(-25.4; 65.8)<br>p=0.37	29.5<br>(-27.0; 86.0)<br>p=0.30	13	39.7<br>(-11.2; 90.7)<br>p=0.12	13	59.8<br>(10.2; 109.3)<br>p=0.020	12	20.1<br>(-25.9; 66.0)<br>p=0.38	19.7<br>(-42.9; 82.3)<br>p=0.53	39.7<br>(-33.2; 112.6)<br>p=0.28
**Subgroup population according to the protocol for feces analyses with non-missing value at any time point**	**W4**	28	68.7<br>(10.4; 127.0)<br>p=0.022	14	81.2<br>(4.5; 157.9)<br>p=0.039	-12.5<br>(-112.0; 87.1)<br>p=0.80	14	24.6<br>(-32.0; 81.1)<br>p=0.38	14	114.3<br>(19.5; 209.0)<br>p=0.019	14	80.1<br>(2.2; 158.1)<br>p=0.044	-55.6<br>(-154.9; 43.8)<br>p=0.26	34.1<br>(-92.9; 161.2)<br>p=0.59
	**W8**	27	40.2<br>(-15.8; 96.2)<br>p=0.15	13	58.8<br>(-13.6; 131.3)<br>p=0.11	-18.6<br>(-110.1; 72.8)<br>p=0.68	13	-1.1<br>(-45.2; 42.9)<br>p=0.96	14	80.6<br>(-18.0; 179.3)<br>p=0.11	13	57.8<br>(-14.3; 129.9)<br>p=0.11	-58.9<br>(-144.9; 27.1)<br>p=0.17	22.9<br>(-99.8; 145.5)<br>p=0.71
	**W10**	29	61.7<br>(11.5; 112.0)<br>p=0.017	14	13.0<br>(-27.5; 53.4)<br>p=0.52	48.8<br>(-18.1; 115.6)<br>p=0.15	15	49.2<br>(-25.9; 124.3)<br>p=0.19	14	76.2<br>(15.0; 137.3)<br>p=0.016	14	11.9<br>(-28.6; 52.5)<br>p=0.55	37.3<br>(-50.9; 125.4)<br>p=0.40	64.2<br>(-12.0; 140.4)<br>p=0.096
**Subgroup population according to the protocol for feces analyses excl outliers with non-missing value at any time point**	**W4**	25	35.0<br>(-10.3; 80.3)<br>p=0.13	13	51.7<br>(-4.7; 108.1)<br>p=0.071	-16.7<br>(-89.6; 56.1)<br>p=0.64	13	-0.6<br>(-29.3; 28.1)<br>p=0.97	12	75.5<br>(-10.2; 161.2)<br>p=0.082	13	50.5<br>(-6.1; 107.1)<br>p=0.078	-51.1<br>(-115.6; 13.5)<br>p=0.12	25.0<br>(-80.1; 130.0)<br>p=0.63
	**W8**	24	9.7<br>(-25.1; 44.5)<br>p=0.58	12	65.2<br>(-11.3; 141.7)<br>p=0.093	-55.5<br>(-142.0; 31.0)<br>p=0.20	12	-8.6<br>(-57.8; 40.5)<br>p=0.72	12	30.7<br>(-12.0; 73.4)<br>p=0.15	12	64.0<br>(-12.5; 140.5)<br>p=0.098	-72.6<br>(-166.8; 21.6)<br>p=0.13	-33.3<br>(-123.4; 56.8)<br>p=0.46
	**W10**	26	28.2<br>(-2.4; 58.7)<br>p=0.069	13	13.9<br>(-24.8; 52.6)<br>p=0.47	14.3<br>(-37.4; 66.0)<br>p=0.58	14	11.6<br>(-26.0; 49.1)<br>p=0.54	12	48.8<br>(0.8; 96.9)<br>p=0.046	13	12.7<br>(-26.2; 51.5)<br>p=0.51	-1.1<br>(-57.4; 55.1)<br>p=0.97	36.2<br>(-30.9; 103.2)<br>p=0.28
**Subgroup population according to the protocol for feces analyses with complete cases at all time points**	**W4**	27	75.3<br>(14.9; 135.6)<br>p=0.016	13	81.9<br>(1.4; 162.3)<br>p=0.046	-6.6<br>(-110.7; 97.5)<br>p=0.90	13	32.9<br>(-29.9; 95.6)<br>p=0.30	14	115.3<br>(21.3; 209.3)<br>p=0.018	13	81.1<br>(-0.2; 162.5)<br>p=0.051	-48.3<br>(-154.8; 58.2)<br>p=0.36	34.2<br>(-93.9; 162.3)<br>p=0.59
	**W8**	27	46.6<br>(-10.0; 103.2)<br>p=0.10	13	57.6<br>(-17.1; 132.3)<br>p=0.13	-11.0<br>(-104.7; 82.7)<br>p=0.81	13	9.5<br>(-35.7; 54.8)<br>p=0.67	14	81.7<br>(-16.1; 179.5)<br>p=0.099	13	56.9<br>(-17.5; 131.3)<br>p=0.13	-47.3<br>(-136.1; 41.4)<br>p=0.29	24.8<br>(-98.2; 147.9)<br>p=0.68
	**W10**	27	71.3<br>(18.6; 123.9)<br>p=0.0094	13	8.9<br>(-34.4; 52.1)<br>p=0.68	62.4<br>(-9.2; 134.1)<br>p=0.086	13	65.6<br>(-18.7; 149.9)<br>p=0.12	14	77.2<br>(17.0; 137.4)<br>p=0.013	13	8.1<br>(-35.0; 51.2)<br>p=0.70	57.5<br>(-41.1; 156.1)<br>p=0.24	69.1<br>(-7.6; 145.8)<br>p=0.076
**Subgroup population according to the protocol for feces analyses excl outliers with complete cases at all time points**	**W4**	24	39.7<br>(-7.5; 86.9)<br>p=0.096	12	52.7<br>(-6.8; 112.1)<br>p=0.081	-12.9<br>(-89.6; 63.7)<br>p=0.73	12	3.9<br>(-27.7; 35.4)<br>p=0.80	12	76.4<br>(-9.1; 162.0)<br>p=0.078	12	51.8<br>(-7.8; 111.4)<br>p=0.086	-47.9<br>(-116.4; 20.5)<br>p=0.16	24.7<br>(-81.7; 131.0)<br>p=0.64

		MMRM comparing High+Low dose vs Placebo					MMRM comparing High dose vs Placebo and Low dose vs Placebo							
		High+Low dose		Placebo		High+Low dose vs Placebo	High dose		Low dose		Placebo		High dose vs Placebo	Low dose vs Placebo
Population	Time Point	n	LS mean (95% CI) p-value	n	LS mean (95% CI) p-value	Difference in LS mean (95% CI) p-value	n	LS mean (95% CI) p-value	n	LS mean (95% CI) p-value	n	LS mean (95% CI) p-value	Difference in LS mean (95% CI) p-value	Difference in LS mean (95% CI) p-value
	**W8**	24	13.5 (-20.9; 47.9) p=0.43	12	65.1 (-12.5; 142.7) p=0.097	-51.6 (-138.7; 35.4) p=0.24	12	-3.8 (-53.2; 45.5) p=0.88	12	31.7 (-10.6; 73.9) p=0.14	12	64.2 (-13.4; 141.8) p=0.10	-68.0 (-162.8; 26.7) p=0.15	-32.5 (-123.1; 58.0) p=0.47
	**W10**	24	35.4 (3.6; 67.2) p=0.030	12	12.1 (-29.0; 53.3) p=0.55	23.3 (-31.0; 77.6) p=0.39	12	21.9 (-19.5; 63.4) p=0.29	12	49.8 (2.0; 97.6) p=0.042	12	11.3 (-29.9; 52.5) p=0.58	10.7 (-49.6; 71.0) p=0.72	38.5 (-29.2; 106.3) p=0.25

2020-01-02 Analys.sas

Table 14.2.5.5 Posthoc Analyses - Propionate - MMRM on various populations

		MMRM comparing High+Low dose vs Placebo					MMRM comparing High dose vs Placebo and Low dose vs Placebo							
		High+Low dose		Placebo		High+Low dose vs Placebo	High dose		Low dose		Placebo		High dose vs Placebo	Low dose vs Placebo
Population	Time Point	n	LS mean<br>(95% CI)<br>p-value	n	LS mean<br>(95% CI)<br>p-value	Difference in LS mean<br>(95% CI)<br>p-value	n	LS mean<br>(95% CI)<br>p-value	n	LS mean<br>(95% CI)<br>p-value	n	LS mean<br>(95% CI)<br>p-value	Difference in LS mean<br>(95% CI)<br>p-value	Difference in LS mean<br>(95% CI)<br>p-value
**ITT Population with non-missing value at any time point**	**W4**	31	44.6<br>(-5.9; 95.1)<br>p=0.082	14	38.3<br>(-2.4; 78.9)<br>p=0.064	6.4<br>(-60.7; 73.4)<br>p=0.85	15	31.0<br>(-14.6; 76.7)<br>p=0.18	16	57.7<br>(-29.3; 144.7)<br>p=0.19	14	38.0<br>(-2.8; 78.9)<br>p=0.067	-7.0<br>(-68.5; 54.5)<br>p=0.82	19.6<br>(-79.1; 118.4)<br>p=0.69
	**W8**	30	26.0<br>(-9.5; 61.4)<br>p=0.15	13	53.9<br>(-6.8; 114.5)<br>p=0.080	-27.9<br>(-97.0; 41.2)<br>p=0.42	14	29.0<br>(-35.2; 93.3)<br>p=0.37	16	25.2<br>(-13.5; 63.9)<br>p=0.20	13	53.7<br>(-6.9; 114.4)<br>p=0.081	-24.7<br>(-110.9; 61.6)<br>p=0.57	-28.5<br>(-100.6; 43.5)<br>p=0.43
	**W10**	32	52.7<br>(8.7; 96.7)<br>p=0.020	14	4.6<br>(-23.1; 32.2)<br>p=0.74	48.1<br>(-4.1; 100.3)<br>p=0.070	16	25.9<br>(-10.3; 62.0)<br>p=0.16	16	79.7<br>(1.4; 158.0)<br>p=0.046	14	4.3<br>(-23.5; 32.2)<br>p=0.75	21.5<br>(-24.2; 67.2)<br>p=0.35	75.4<br>(-8.6; 159.3)<br>p=0.077
**ITT Population excl outliers with non-missing value at any time point**	**W4**	27	12.9<br>(-10.6; 36.4)<br>p=0.27	12	32.6<br>(-8.6; 73.7)<br>p=0.12	-19.6<br>(-66.9; 27.6)<br>p=0.40	13	8.2<br>(-23.5; 39.8)<br>p=0.60	14	17.5<br>(-18.2; 53.2)<br>p=0.33	12	32.1<br>(-9.1; 73.4)<br>p=0.12	-24.0<br>(-75.6; 27.7)<br>p=0.35	-14.6<br>(-69.2; 40.0)<br>p=0.59
	**W8**	26	4.0<br>(-26.3; 34.2)<br>p=0.79	11	32.2<br>(-8.5; 72.8)<br>p=0.12	-28.2<br>(-79.9; 23.4)<br>p=0.28	12	-3.9<br>(-49.3; 41.5)<br>p=0.86	14	12.7<br>(-25.7; 51.1)<br>p=0.51	11	31.7<br>(-9.1; 72.5)<br>p=0.12	-35.6<br>(-98.0; 26.8)<br>p=0.25	-19.0<br>(-75.6; 37.5)<br>p=0.50
	**W10**	28	22.6<br>(-3.8; 48.9)<br>p=0.091	12	2.1<br>(-24.6; 28.8)<br>p=0.87	20.5<br>(-17.4; 58.4)<br>p=0.28	14	5.1<br>(-22.3; 32.5)<br>p=0.71	14	40.4<br>(-2.6; 83.4)<br>p=0.065	12	1.7<br>(-25.1; 28.4)<br>p=0.90	3.4<br>(-36.4; 43.2)<br>p=0.86	38.7<br>(-12.3; 89.8)<br>p=0.13
**ITT Population with complete cases at all time points**	**W4**	30	46.7<br>(-5.5; 98.9)<br>p=0.078	13	40.1<br>(-3.2; 83.4)<br>p=0.069	6.6<br>(-63.3; 76.5)<br>p=0.85	14	33.3<br>(-15.7; 82.2)<br>p=0.18	16	58.6<br>(-28.5; 145.6)<br>p=0.18	13	39.9<br>(-3.7; 83.4)<br>p=0.072	-6.6<br>(-72.1; 58.9)<br>p=0.84	18.7<br>(-81.2; 118.6)<br>p=0.71
	**W8**	30	27.6<br>(-8.6; 63.9)<br>p=0.13	13	53.9<br>(-6.9; 114.7)<br>p=0.081	-26.3<br>(-95.8; 43.2)<br>p=0.45	14	29.6<br>(-36.4; 95.7)<br>p=0.37	16	26.0<br>(-12.6; 64.7)<br>p=0.18	13	53.7<br>(-7.2; 114.6)<br>p=0.082	-24.1<br>(-111.5; 63.4)<br>p=0.58	-27.7<br>(-99.8; 44.5)<br>p=0.44
	**W10**	30	55.0<br>(8.0; 102.1)<br>p=0.023	13	3.1<br>(-26.5; 32.7)<br>p=0.83	51.9<br>(-4.1; 108.0)<br>p=0.068	14	26.0<br>(-15.4; 67.5)<br>p=0.21	16	80.6<br>(2.2; 159.0)<br>p=0.044	13	2.9<br>(-26.9; 32.7)<br>p=0.84	23.1<br>(-27.9; 74.2)<br>p=0.36	77.7<br>(-7.1; 162.5)<br>p=0.071
**ITT Population excl outliers with complete cases at all time points**	**W4**	26	14.1<br>(-10.5; 38.7)<br>p=0.25	11	33.9<br>(-9.6; 77.3)<br>p=0.12	-19.8<br>(-69.4; 29.9)<br>p=0.42	12	9.0<br>(-25.3; 43.4)<br>p=0.60	14	18.9<br>(-17.1; 54.8)<br>p=0.29	11	33.3<br>(-10.3; 77.0)<br>p=0.13	-24.3<br>(-79.2; 30.6)<br>p=0.37	-14.5<br>(-71.1; 42.2)<br>p=0.61
	**W8**	26	4.6<br>(-26.6; 35.7)<br>p=0.77	11	31.2<br>(-11.3; 73.7)<br>p=0.14	-26.7<br>(-80.6; 27.2)<br>p=0.32	12	-6.0<br>(-53.0; 41.0)<br>p=0.80	14	14.1<br>(-24.5; 52.6)<br>p=0.46	11	30.7<br>(-12.0; 73.3)<br>p=0.15	-36.7<br>(-102.0; 28.6)<br>p=0.26	-16.6<br>(-74.8; 41.6)<br>p=0.56

		MMRM comparing High+Low dose vs Placebo					MMRM comparing High dose vs Placebo and Low dose vs Placebo							
		High+Low dose		Placebo		High+Low dose vs Placebo	High dose		Low dose		Placebo		High dose vs Placebo	Low dose vs Placebo
Population	Time Point	n	LS mean<br>(95% CI)<br>p-value	n	LS mean<br>(95% CI)<br>p-value	Difference in LS mean<br>(95% CI)<br>p-value	n	LS mean<br>(95% CI)<br>p-value	n	LS mean<br>(95% CI)<br>p-value	n	LS mean<br>(95% CI)<br>p-value	Difference in LS mean<br>(95% CI)<br>p-value	Difference in LS mean<br>(95% CI)<br>p-value
	**W10**	26	22.8<br>(-5.8; 51.4)<br>p=0.11	11	-0.2<br>(-29.0; 28.6)<br>p=0.99	23.0<br>(-18.6; 64.6)<br>p=0.27	12	1.2<br>(-30.3; 32.8)<br>p=0.94	14	41.7<br>(-1.2; 84.6)<br>p=0.056	11	-0.7<br>(-29.5; 28.1)<br>p=0.96	2.0<br>(-42.7; 46.6)<br>p=0.93	42.5<br>(-10.3; 95.2)<br>p=0.11
**Subgroup population according to the protocol for feces analyses with non-missing value at any time point**	**W4**	28	42.6<br>(-13.2; 98.4)<br>p=0.13	14	30.5<br>(-10.2; 71.2)<br>p=0.14	12.1<br>(-59.8; 83.9)<br>p=0.74	14	23.0<br>(-24.8; 70.9)<br>p=0.34	14	62.7<br>(-36.3; 161.6)<br>p=0.21	14	30.3<br>(-10.7; 71.2)<br>p=0.14	-7.2<br>(-72.3; 57.8)<br>p=0.82	32.4<br>(-77.2; 142.1)<br>p=0.55
	**W8**	27	11.0<br>(-17.9; 39.9)<br>p=0.44	13	47.2<br>(-11.9; 106.3)<br>p=0.11	-36.2<br>(-103.2; 30.9)<br>p=0.28	13	2.7<br>(-38.0; 43.4)<br>p=0.89	14	19.9<br>(-18.7; 58.5)<br>p=0.30	13	46.9<br>(-12.0; 105.8)<br>p=0.12	-44.1<br>(-118.5; 30.2)<br>p=0.24	-27.0<br>(-97.2; 43.2)<br>p=0.44
	**W10**	29	29.1<br>(0.6; 57.6)<br>p=0.045	14	-3.2<br>(-27.1; 20.8)<br>p=0.79	32.3<br>(-5.6; 70.2)<br>p=0.093	15	19.0<br>(-14.5; 52.5)<br>p=0.26	14	40.3<br>(-5.0; 85.5)<br>p=0.080	14	-3.4<br>(-27.5; 20.6)<br>p=0.77	22.4<br>(-21.1; 65.9)<br>p=0.30	43.7<br>(-7.8; 95.2)<br>p=0.094
**Subgroup population according to the protocol for feces analyses excl outliers with non-missing value at any time point**	**W4**	26	8.8<br>(-14.4; 32.0)<br>p=0.45	12	32.7<br>(-8.3; 73.7)<br>p=0.11	-23.9<br>(-70.9; 23.1)<br>p=0.31	13	7.7<br>(-23.2; 38.5)<br>p=0.62	13	10.0<br>(-25.8; 45.9)<br>p=0.57	12	32.4<br>(-8.7; 73.4)<br>p=0.12	-24.7<br>(-76.0; 26.6)<br>p=0.33	-22.3<br>(-76.7; 32.0)<br>p=0.41
	**W8**	25	4.6<br>(-27.0; 36.2)<br>p=0.77	11	32.9<br>(-7.6; 73.4)<br>p=0.11	-28.3<br>(-80.5; 23.9)<br>p=0.28	12	-3.5<br>(-49.6; 42.6)<br>p=0.88	13	13.9<br>(-27.8; 55.5)<br>p=0.50	11	32.6<br>(-8.0; 73.1)<br>p=0.11	-36.0<br>(-98.6; 26.5)<br>p=0.25	-18.7<br>(-77.4; 40.1)<br>p=0.52
	**W10**	27	17.2<br>(-8.5; 42.8)<br>p=0.18	12	2.2<br>(-24.5; 29.0)<br>p=0.87	14.9<br>(-22.4; 52.3)<br>p=0.42	14	4.8<br>(-23.7; 33.4)<br>p=0.73	13	30.8<br>(-11.5; 73.0)<br>p=0.15	12	1.9<br>(-24.8; 28.6)<br>p=0.89	3.0<br>(-37.6; 43.5)<br>p=0.88	28.9<br>(-21.3; 79.0)<br>p=0.25
**Subgroup population according to the protocol for feces analyses with complete cases at all time points**	**W4**	27	44.3<br>(-13.6; 102.3)<br>p=0.13	13	33.3<br>(-10.2; 76.7)<br>p=0.13	11.1<br>(-64.0; 86.1)<br>p=0.77	13	24.2<br>(-27.7; 76.1)<br>p=0.35	14	63.4<br>(-35.7; 162.5)<br>p=0.20	13	32.9<br>(-10.8; 76.6)<br>p=0.14	-8.7<br>(-78.5; 61.0)<br>p=0.80	30.5<br>(-80.4; 141.3)<br>p=0.58
	**W8**	27	11.5<br>(-18.2; 41.2)<br>p=0.44	13	47.1<br>(-12.1; 106.4)<br>p=0.12	-35.7<br>(-103.0; 31.7)<br>p=0.29	13	2.0<br>(-40.2; 44.2)<br>p=0.92	14	20.6<br>(-18.0; 59.2)<br>p=0.29	13	46.8<br>(-12.2; 105.8)<br>p=0.12	-44.7<br>(-120.0; 30.5)<br>p=0.24	-26.2<br>(-96.4; 44.0)<br>p=0.45
	**W10**	27	29.5<br>(-1.3; 60.3)<br>p=0.060	13	-3.7<br>(-29.3; 22.0)<br>p=0.77	33.2<br>(-7.7; 74.0)<br>p=0.11	13	17.5<br>(-21.1; 56.1)<br>p=0.36	14	41.0<br>(-4.4; 86.4)<br>p=0.075	13	-4.0<br>(-29.9; 21.8)<br>p=0.75	21.6<br>(-27.2; 70.3)<br>p=0.38	45.0<br>(-7.6; 97.6)<br>p=0.091
**Subgroup population according to the protocol for feces analyses excl outliers with complete cases at all time points**	**W4**	25	9.8<br>(-14.5; 34.2)<br>p=0.42	11	34.3<br>(-8.9; 77.6)<br>p=0.12	-24.5<br>(-73.8; 24.9)<br>p=0.32	12	8.6<br>(-25.0; 42.2)<br>p=0.61	13	11.4<br>(-24.8; 47.6)<br>p=0.52	11	33.8<br>(-9.6; 77.3)<br>p=0.12	-25.3<br>(-79.8; 29.3)<br>p=0.35	-22.4<br>(-78.8; 33.9)<br>p=0.42

		MMRM comparing High+Low dose vs Placebo					MMRM comparing High dose vs Placebo and Low dose vs Placebo							
		High+Low dose		Placebo		High+Low dose vs Placebo	High dose		Low dose		Placebo		High dose vs Placebo	Low dose vs Placebo
Population	Time Point	n	LS mean (95% CI) p-value	n	LS mean (95% CI) p-value	Difference in LS mean (95% CI) p-value	n	LS mean (95% CI) p-value	n	LS mean (95% CI) p-value	n	LS mean (95% CI) p-value	Difference in LS mean (95% CI) p-value	Difference in LS mean (95% CI) p-value
	**W8**	25	4.6 (-28.2; 37.4) p=0.78	11	31.7 (-10.6; 74.0) p=0.14	-27.1 (-81.9; 27.7) p=0.32	12	-6.5 (-54.6; 41.7) p=0.79	13	15.2 (-26.5; 57.0) p=0.46	11	31.2 (-11.2; 73.7) p=0.14	-37.7 (-103.5; 28.1) p=0.25	-16.0 (-76.5; 44.6) p=0.59
	**W10**	25	16.9 (-11.1; 44.8) p=0.23	11	0.3 (-28.5; 29.1) p=0.98	16.6 (-24.3; 57.6) p=0.42	12	0.8 (-32.1; 33.7) p=0.96	13	32.1 (-10.2; 74.5) p=0.13	11	-0.2 (-29.0; 28.6) p=0.99	1.0 (-44.5; 46.5) p=0.96	32.3 (-19.6; 84.3) p=0.21
2020-01-02 Analys.sas														

Table 14.2.5.6 Posthoc Analyses - Acetate - MMRM on various populations

		MMRM comparing High+Low dose vs Placebo					MMRM comparing High dose vs Placebo and Low dose vs Placebo							
		High+Low dose		Placebo		High+Low dose vs Placebo	High dose		Low dose		Placebo		High dose vs Placebo	Low dose vs Placebo
Population	Time Point	n	LS mean<br>(95% CI)<br>p-value	n	LS mean<br>(95% CI)<br>p-value	Difference in LS mean<br>(95% CI)<br>p-value	n	LS mean<br>(95% CI)<br>p-value	n	LS mean<br>(95% CI)<br>p-value	n	LS mean<br>(95% CI)<br>p-value	Difference in LS mean<br>(95% CI)<br>p-value	Difference in LS mean<br>(95% CI)<br>p-value
**ITT Population with non-missing value at any time point**	**W4**	31	35.9<br>(-3.6; 75.3)<br>p=0.074	14	38.6<br>(-0.0; 77.1)<br>p=0.051	-2.7<br>(-59.3; 53.9)<br>p=0.92	15	31.5<br>(-8.6; 71.6)<br>p=0.12	16	40.6<br>(-25.8; 107.0)<br>p=0.22	14	38.0<br>(-0.7; 76.7)<br>p=0.054	-6.5<br>(-63.2; 50.1)<br>p=0.82	2.6<br>(-75.7; 80.9)<br>p=0.95
	**W8**	30	25.9<br>(-3.6; 55.5)<br>p=0.084	13	48.2<br>(1.6; 94.8)<br>p=0.043	-22.2<br>(-76.8; 32.3)<br>p=0.42	14	15.2<br>(-29.4; 59.8)<br>p=0.50	16	37.5<br>(-3.6; 78.5)<br>p=0.073	13	47.7<br>(1.2; 94.3)<br>p=0.045	-32.5<br>(-95.0; 29.9)<br>p=0.30	-10.3<br>(-72.9; 52.4)<br>p=0.74
	**W10**	32	44.8<br>(10.5; 79.2)<br>p=0.012	14	10.5<br>(-19.8; 40.7)<br>p=0.49	34.4<br>(-10.5; 79.2)<br>p=0.13	16	16.5<br>(-14.8; 47.7)<br>p=0.29	16	73.6<br>(14.9; 132.4)<br>p=0.015	14	9.9<br>(-20.2; 40.1)<br>p=0.51	6.5<br>(-36.3; 49.4)<br>p=0.76	63.7<br>(-2.3; 129.6)<br>p=0.058
**ITT Population excl outliers with non-missing value at any time point**	**W4**	24	8.2<br>(-11.3; 27.7)<br>p=0.40	12	9.1<br>(-18.8; 36.9)<br>p=0.51	-0.9<br>(-34.5; 32.7)<br>p=0.96	12	1.4<br>(-21.9; 24.7)<br>p=0.90	12	15.1<br>(-17.8; 47.9)<br>p=0.36	12	8.9<br>(-19.1; 37.0)<br>p=0.52	-7.5<br>(-43.7; 28.7)<br>p=0.68	6.2<br>(-36.9; 49.2)<br>p=0.77
	**W8**	23	-4.7<br>(-27.2; 17.9)<br>p=0.68	11	24.3<br>(-12.9; 61.5)<br>p=0.19	-28.9<br>(-72.3; 14.5)<br>p=0.18	11	-10.0<br>(-45.2; 25.2)<br>p=0.57	12	0.5<br>(-28.6; 29.6)<br>p=0.97	11	24.1<br>(-13.1; 61.3)<br>p=0.20	-34.1<br>(-84.6; 16.4)<br>p=0.18	-23.6<br>(-71.3; 24.2)<br>p=0.32
	**W10**	25	12.9<br>(-6.0; 31.9)<br>p=0.17	12	-2.0<br>(-28.5; 24.5)<br>p=0.88	15.0<br>(-17.8; 47.8)<br>p=0.36	13	11.1<br>(-18.3; 40.5)<br>p=0.45	12	15.1<br>(-8.8; 39.0)<br>p=0.21	12	-2.2<br>(-28.7; 24.3)<br>p=0.87	13.3<br>(-25.9; 52.4)<br>p=0.50	17.3<br>(-19.4; 53.9)<br>p=0.34
**ITT Population with complete cases at all time points**	**W4**	30	39.7<br>(-0.6; 80.0)<br>p=0.053	13	38.0<br>(-2.4; 78.4)<br>p=0.064	1.6<br>(-56.8; 60.1)<br>p=0.96	14	38.5<br>(-2.6; 79.6)<br>p=0.066	16	41.0<br>(-25.1; 107.2)<br>p=0.22	13	37.7<br>(-2.8; 78.2)<br>p=0.067	0.8<br>(-57.9; 59.5)<br>p=0.98	3.4<br>(-75.6; 82.3)<br>p=0.93
	**W8**	30	28.4<br>(-1.4; 58.2)<br>p=0.061	13	48.6<br>(1.4; 95.7)<br>p=0.044	-20.1<br>(-75.1; 34.8)<br>p=0.46	14	17.9<br>(-27.1; 63.0)<br>p=0.42	16	37.9<br>(-2.9; 78.7)<br>p=0.068	13	48.2<br>(1.1; 95.3)<br>p=0.045	-30.2<br>(-93.3; 32.8)<br>p=0.34	-10.3<br>(-73.0; 52.4)<br>p=0.74
	**W10**	30	49.8<br>(13.7; 85.9)<br>p=0.0081	13	8.0<br>(-24.1; 40.0)<br>p=0.62	41.8<br>(-5.6; 89.2)<br>p=0.082	14	22.4<br>(-12.2; 56.9)<br>p=0.20	16	74.1<br>(15.4; 132.7)<br>p=0.015	13	7.6<br>(-24.3; 39.5)<br>p=0.63	14.8<br>(-31.6; 61.1)<br>p=0.52	66.5<br>(-0.2; 133.1)<br>p=0.051
**ITT Population excl outliers with complete cases at all time points**	**W4**	23	11.4<br>(-8.6; 31.3)<br>p=0.25	11	9.2<br>(-20.8; 39.3)<br>p=0.53	2.1<br>(-33.4; 37.7)<br>p=0.90	11	7.5<br>(-16.7; 31.6)<br>p=0.53	12	15.0<br>(-17.5; 47.6)<br>p=0.35	11	9.2<br>(-21.0; 39.3)<br>p=0.54	-1.7<br>(-39.8; 36.4)<br>p=0.93	5.9<br>(-38.4; 50.1)<br>p=0.79
	**W8**	23	-3.5<br>(-25.7; 18.8)<br>p=0.75	11	25.0<br>(-13.0; 63.0)<br>p=0.19	-28.5<br>(-72.3; 15.3)<br>p=0.19	11	-7.7<br>(-43.2; 27.7)<br>p=0.66	12	0.5<br>(-28.1; 29.1)<br>p=0.97	11	24.9<br>(-13.1; 63.0)<br>p=0.19	-32.7<br>(-83.7; 18.3)<br>p=0.20	-24.4<br>(-72.5; 23.6)<br>p=0.31

		MMRM comparing High+Low dose vs Placebo					MMRM comparing High dose vs Placebo and Low dose vs Placebo							
		High+Low dose		Placebo		High+Low dose vs Placebo	High dose		Low dose		Placebo		High dose vs Placebo	Low dose vs Placebo
**Population**	**Time Point**	**n**	**LS mean (95% CI) p-value**	**n**	**LS mean (95% CI) p-value**	**Difference in LS mean (95% CI) p-value**	**n**	**LS mean (95% CI) p-value**	**n**	**LS mean (95% CI) p-value**	**n**	**LS mean (95% CI) p-value**	**Difference in LS mean (95% CI) p-value**	**Difference in LS mean (95% CI) p-value**
	**W10**	23	15.5<br>(-4.3; 35.3)<br>p=0.12	11	-2.7<br>(-31.5; 26.2)<br>p=0.85	18.2<br>(-16.8; 53.1)<br>p=0.30	11	16.1<br>(-16.7; 48.9)<br>p=0.32	12	15.0<br>(-8.8; 38.9)<br>p=0.21	11	-2.7<br>(-31.6; 26.1)<br>p=0.85	18.8<br>(-24.1; 61.7)<br>p=0.38	17.8<br>(-20.3; 55.9)<br>p=0.35
**Subgroup population according to the protocol for feces analyses with non-missing value at any time point**	**W4**	28	36.8<br>(-6.5; 80.2)<br>p=0.094	14	33.8<br>(-4.6; 72.3)<br>p=0.083	3.0<br>(-56.2; 62.2)<br>p=0.92	14	30.1<br>(-13.6; 73.9)<br>p=0.17	14	44.6<br>(-30.4; 119.6)<br>p=0.24	14	33.2<br>(-5.5; 71.8)<br>p=0.090	-3.1<br>(-61.8; 55.7)<br>p=0.92	11.4<br>(-74.5; 97.4)<br>p=0.79
	**W8**	27	10.7<br>(-14.4; 35.8)<br>p=0.39	13	43.5<br>(-2.1; 89.1)<br>p=0.061	-32.8<br>(-84.9; 19.3)<br>p=0.21	13	-4.4<br>(-32.4; 23.5)<br>p=0.75	14	26.7<br>(-13.9; 67.4)<br>p=0.19	13	42.8<br>(-2.5; 88.2)<br>p=0.064	-47.2<br>(-101.0; 6.5)<br>p=0.083	-16.1<br>(-76.9; 44.8)<br>p=0.60
	**W10**	29	32.5<br>(3.0; 62.1)<br>p=0.032	14	5.7<br>(-23.5; 35.0)<br>p=0.69	26.8<br>(-12.3; 65.9)<br>p=0.17	15	11.0<br>(-20.0; 42.0)<br>p=0.48	14	56.2<br>(5.0; 107.5)<br>p=0.032	14	5.1<br>(-24.1; 34.3)<br>p=0.73	5.9<br>(-36.8; 48.6)<br>p=0.78	51.1<br>(-5.8; 108.1)<br>p=0.077
**Subgroup population according to the protocol for feces analyses excl outliers with non-missing value at any time point**	**W4**	23	4.3<br>(-14.6; 23.1)<br>p=0.65	12	7.9<br>(-19.6; 35.4)<br>p=0.56	-3.7<br>(-36.7; 29.4)<br>p=0.82	12	1.6<br>(-21.4; 24.6)<br>p=0.89	11	7.2<br>(-24.9; 39.3)<br>p=0.65	12	7.9<br>(-19.7; 35.5)<br>p=0.56	-6.3<br>(-42.1; 29.5)<br>p=0.72	-0.7<br>(-42.8; 41.5)<br>p=0.97
	**W8**	22	-7.6<br>(-31.0; 15.8)<br>p=0.51	11	23.2<br>(-13.9; 60.3)<br>p=0.21	-30.8<br>(-74.6; 13.0)<br>p=0.16	11	-9.9<br>(-45.6; 25.9)<br>p=0.58	11	-5.3<br>(-35.8; 25.2)<br>p=0.73	11	23.2<br>(-13.9; 60.2)<br>p=0.21	-33.0<br>(-83.9; 17.8)<br>p=0.20	-28.4<br>(-77.1; 20.2)<br>p=0.24
	**W10**	24	11.3<br>(-8.3; 30.9)<br>p=0.25	12	-3.2<br>(-29.0; 22.7)<br>p=0.80	14.5<br>(-18.0; 47.0)<br>p=0.37	13	11.1<br>(-18.5; 40.7)<br>p=0.45	11	11.6<br>(-13.3; 36.6)<br>p=0.35	12	-3.2<br>(-29.1; 22.6)<br>p=0.80	14.3<br>(-24.6; 53.2)<br>p=0.46	14.9<br>(-21.7; 51.5)<br>p=0.41
**Subgroup population according to the protocol for feces analyses with complete cases at all time points**	**W4**	27	41.5<br>(-2.9; 85.9)<br>p=0.066	13	33.4<br>(-7.1; 74.0)<br>p=0.10	8.1<br>(-53.3; 69.5)<br>p=0.79	13	38.3<br>(-6.9; 83.5)<br>p=0.094	14	45.0<br>(-29.8; 119.9)<br>p=0.23	13	32.8<br>(-7.8; 73.5)<br>p=0.11	5.5<br>(-55.7; 66.7)<br>p=0.86	12.2<br>(-74.5; 98.9)<br>p=0.78
	**W8**	27	12.7<br>(-12.5; 37.9)<br>p=0.31	13	44.0<br>(-2.1; 90.0)<br>p=0.061	-31.3<br>(-83.6; 21.0)<br>p=0.23	13	-2.3<br>(-30.4; 25.7)<br>p=0.87	14	27.2<br>(-13.2; 67.5)<br>p=0.18	13	43.4<br>(-2.5; 89.2)<br>p=0.063	-45.7<br>(-99.7; 8.3)<br>p=0.095	-16.2<br>(-77.1; 44.7)<br>p=0.59
	**W10**	27	37.0<br>(5.7; 68.4)<br>p=0.022	13	3.4<br>(-27.6; 34.3)<br>p=0.83	33.7<br>(-8.2; 75.6)<br>p=0.11	13	16.5<br>(-18.1; 51.1)<br>p=0.34	14	56.6<br>(5.5; 107.8)<br>p=0.031	13	2.8<br>(-28.1; 33.6)<br>p=0.86	13.7<br>(-32.4; 59.9)<br>p=0.55	53.9<br>(-4.1; 111.9)<br>p=0.068
**Subgroup population according to the protocol for feces analyses excl outliers with complete cases at all time points**	**W4**	22	7.3<br>(-12.1; 26.6)<br>p=0.45	11	8.1<br>(-21.5; 37.8)<br>p=0.58	-0.9<br>(-35.8; 34.1)<br>p=0.96	11	7.2<br>(-16.6; 31.1)<br>p=0.54	11	7.3<br>(-24.7; 39.3)<br>p=0.64	11	8.1<br>(-21.5; 37.8)<br>p=0.58	-0.9<br>(-38.5; 36.7)<br>p=0.96	-0.8<br>(-44.3; 42.6)<br>p=0.97

		MMRM comparing High+Low dose vs Placebo					MMRM comparing High dose vs Placebo and Low dose vs Placebo							
		High+Low dose		Placebo		High+Low dose vs Placebo	High dose		Low dose		Placebo		High dose vs Placebo	Low dose vs Placebo
Population	Time Point	n	LS mean (95% CI) p-value	n	LS mean (95% CI) p-value	Difference in LS mean (95% CI) p-value	n	LS mean (95% CI) p-value	n	LS mean (95% CI) p-value	n	LS mean (95% CI) p-value	Difference in LS mean (95% CI) p-value	Difference in LS mean (95% CI) p-value
	**W8**	22	-6.6 (-29.7; 16.6) p=0.57	11	23.9 (-14.0; 61.8) p=0.21	-30.5 (-74.7; 13.8) p=0.17	11	-8.0 (-43.9; 28.0) p=0.65	11	-5.2 (-35.3; 24.9) p=0.73	11	23.9 (-14.1; 61.9) p=0.21	-31.9 (-83.3; 19.6) p=0.21	-29.1 (-78.1; 19.9) p=0.23
	**W10**	22	13.8 (-6.7; 34.3) p=0.18	11	-3.8 (-32.0; 24.4) p=0.79	17.6 (-17.2; 52.3) p=0.31	11	15.9 (-17.2; 48.9) p=0.33	11	11.7 (-13.2; 36.7) p=0.34	11	-3.8 (-32.0; 24.5) p=0.79	19.6 (-23.1; 62.4) p=0.36	15.5 (-22.7; 53.7) p=0.41
2020-01-02 Analys.sas														

Table 14.2.5.7 Posthoc Analyses - Lactate - MMRM on various populations

		MMRM comparing High+Low dose vs Placebo					MMRM comparing High dose vs Placebo and Low dose vs Placebo							
		High+Low dose		Placebo		High+Low dose vs Placebo	High dose		Low dose		Placebo		High dose vs Placebo	Low dose vs Placebo
Population	Time Point	n	LS mean (95% CI) p-value	n	LS mean (95% CI) p-value	Difference in LS mean (95% CI) p-value	n	LS mean (95% CI) p-value	n	LS mean (95% CI) p-value	n	LS mean (95% CI) p-value	Difference in LS mean (95% CI) p-value	Difference in LS mean (95% CI) p-value
**ITT Population with non-missing value at any time point**	**W4**	30	436.6<br>(158.6; 714.7)<br>p=0.0029	14	216.2<br>(22.3; 410.1)<br>p=0.030	220.4<br>(-125.9; 566.7)<br>p=0.21	15	279.9<br>(80.5; 479.4)<br>p=0.0071	15	591.0<br>(89.2; 1092.8)<br>p=0.022	14	215.7<br>(21.6; 409.8)<br>p=0.030	64.2<br>(-220.1; 348.5)<br>p=0.65	375.3<br>(-169.0; 919.6)<br>p=0.17
	**W8**	30	114.3<br>(-13.5; 242.2)<br>p=0.078	13	155.3<br>(-32.1; 342.7)<br>p=0.10	-40.9<br>(-263.7; 181.9)<br>p=0.71	14	43.6<br>(-37.8; 125.0)<br>p=0.29	16	179.5<br>(-48.4; 407.5)<br>p=0.12	13	154.9<br>(-32.3; 342.2)<br>p=0.10	-111.4<br>(-312.1; 89.4)<br>p=0.27	24.6<br>(-267.2; 316.4)<br>p=0.87
	**W10**	31	327.3<br>(195.8; 458.8)<br>p=<.0001	14	360.3<br>(137.4; 583.2)<br>p=0.0022	-32.9<br>(-298.5; 232.6)<br>p=0.80	16	313.5<br>(157.9; 469.0)<br>p=0.0002	15	343.5<br>(132.1; 554.9)<br>p=0.0021	14	359.8<br>(136.7; 582.9)<br>p=0.0023	-46.3<br>(-326.2; 233.6)<br>p=0.74	-16.3<br>(-328.1; 295.5)<br>p=0.92
**ITT Population excl outliers with non-missing value at any time point**	**W4**	26	195.0<br>(119.9; 270.2)<br>p=<.0001	10	145.0<br>(67.3; 222.7)<br>p=0.0006	50.1<br>(-58.2; 158.3)<br>p=0.35	14	205.7<br>(92.6; 318.7)<br>p=0.0008	12	181.6<br>(85.5; 277.7)<br>p=0.0005	10	145.0<br>(67.2; 222.8)<br>p=0.0006	60.7<br>(-77.6; 198.9)<br>p=0.38	36.6<br>(-86.5; 159.7)<br>p=0.55
	**W8**	26	60.3<br>(11.2; 109.4)<br>p=0.018	9	67.2<br>(2.4; 131.9)<br>p=0.043	-6.9<br>(-86.9; 73.1)<br>p=0.86	13	55.4<br>(-26.9; 137.7)<br>p=0.18	13	66.4<br>(10.9; 121.9)<br>p=0.020	9	67.2<br>(2.3; 132.0)<br>p=0.043	-11.8<br>(-114.7; 91.2)<br>p=0.82	-0.8<br>(-85.8; 84.3)<br>p=0.99
	**W10**	27	269.2<br>(160.4; 378.0)<br>p=<.0001	10	280.7<br>(97.3; 464.1)<br>p=0.0038	-11.5<br>(-225.9; 202.9)<br>p=0.91	15	303.7<br>(148.4; 459.1)<br>p=0.0004	12	226.7<br>(78.4; 374.9)<br>p=0.0038	10	280.7<br>(97.1; 464.3)<br>p=0.0038	23.0<br>(-219.6; 265.5)<br>p=0.85	-54.1<br>(-290.5; 182.3)<br>p=0.64
**ITT Population with complete cases at all time points**	**W4**	29	452.0<br>(162.4; 741.7)<br>p=0.0031	13	233.8<br>(29.8; 437.7)<br>p=0.026	218.3<br>(-143.1; 579.7)<br>p=0.23	14	306.1<br>(92.0; 520.3)<br>p=0.0063	15	588.4<br>(78.0; 1098.9)<br>p=0.025	13	233.5<br>(29.4; 437.7)<br>p=0.026	72.6<br>(-229.8; 375.0)<br>p=0.63	354.9<br>(-200.5; 910.3)<br>p=0.20
	**W8**	29	111.8<br>(-22.8; 246.5)<br>p=0.10	13	159.4<br>(-28.6; 347.5)<br>p=0.094	-47.6<br>(-275.2; 180.0)<br>p=0.67	14	42.4<br>(-42.6; 127.4)<br>p=0.32	15	176.8<br>(-66.2; 419.9)<br>p=0.15	13	159.2<br>(-28.8; 347.2)<br>p=0.095	-116.8<br>(-319.8; 86.2)<br>p=0.25	17.6<br>(-286.6; 321.8)<br>p=0.91
	**W10**	29	335.6<br>(195.0; 476.2)<br>p=<.0001	13	355.3<br>(115.8; 594.9)<br>p=0.0047	-19.7<br>(-304.0; 264.6)<br>p=0.89	14	329.6<br>(153.8; 505.4)<br>p=0.0005	15	341.4<br>(128.2; 554.6)<br>p=0.0025	13	355.1<br>(115.3; 594.9)<br>p=0.0048	-25.5<br>(-330.2; 279.3)<br>p=0.87	-13.7<br>(-339.2; 311.9)<br>p=0.93
**ITT Population excl outliers with complete cases at all time points**	**W4**	25	204.7<br>(127.6; 281.7)<br>p=<.0001	9	157.5<br>(79.2; 235.7)<br>p=0.0003	47.2<br>(-62.5; 156.9)<br>p=0.39	13	226.5<br>(108.6; 344.5)<br>p=0.0005	12	180.8<br>(85.4; 276.3)<br>p=0.0006	9	157.6<br>(79.6; 235.7)<br>p=0.0003	68.9<br>(-73.5; 211.3)<br>p=0.33	23.2<br>(-99.3; 145.8)<br>p=0.70
	**W8**	25	53.1<br>(0.3; 105.9)<br>p=0.049	9	69.6<br>(4.0; 135.3)<br>p=0.038	-16.5<br>(-99.2; 66.2)<br>p=0.69	13	48.7<br>(-38.7; 136.0)<br>p=0.26	12	57.8<br>(-2.1; 117.6)<br>p=0.058	9	69.8<br>(4.0; 135.6)<br>p=0.038	-21.1<br>(-128.6; 86.4)<br>p=0.69	-12.0<br>(-100.5; 76.4)<br>p=0.78

		MMRM comparing High+Low dose vs Placebo					MMRM comparing High dose vs Placebo and Low dose vs Placebo							
		High+Low dose		Placebo		High+Low dose vs Placebo	High dose		Low dose		Placebo		High dose vs Placebo	Low dose vs Placebo
Population	Time Point	n	LS mean (95% CI) p-value	n	LS mean (95% CI) p-value	Difference in LS mean (95% CI) p-value	n	LS mean (95% CI) p-value	n	LS mean (95% CI) p-value	n	LS mean (95% CI) p-value	Difference in LS mean (95% CI) p-value	Difference in LS mean (95% CI) p-value
	**W10**	25	277.9<br>(159.9; 395.9)<br>p=<.0001	9	259.8<br>(60.0; 459.7)<br>p=0.013	18.1<br>(-214.9; 251.0)<br>p=0.88	13	321.9<br>(145.9; 497.9)<br>p=0.0008	12	230.0<br>(80.6; 379.4)<br>p=0.0038	9	260.0<br>(60.1; 459.9)<br>p=0.013	61.9<br>(-206.0; 329.8)<br>p=0.64	-30.0<br>(-279.9; 220.0)<br>p=0.81
**Subgroup population according to the protocol for feces analyses with non-missing value at any time point**	**W4**	27	462.5<br>(155.0; 770.1)<br>p=0.0042	14	215.9<br>(22.0; 409.9)<br>p=0.030	246.6<br>(-123.8; 617.0)<br>p=0.19	14	290.3<br>(76.7; 503.9)<br>p=0.0091	13	643.2<br>(68.4; 1217.9)<br>p=0.029	14	214.9<br>(20.7; 409.0)<br>p=0.031	75.4<br>(-218.0; 368.7)<br>p=0.61	428.3<br>(-184.9;<br>1041.4)<br>p=0.17
	**W8**	27	114.4<br>(-25.6; 254.4)<br>p=0.11	13	155.3<br>(-33.0; 343.5)<br>p=0.10	-40.9<br>(-271.0; 189.2)<br>p=0.72	13	18.1<br>(-52.3; 88.4)<br>p=0.61	14	209.2<br>(-49.2; 467.6)<br>p=0.11	13	154.6<br>(-33.3; 342.6)<br>p=0.10	-136.6<br>(-334.5; 61.4)<br>p=0.17	54.6<br>(-261.0; 370.2)<br>p=0.73
	**W10**	28	331.9<br>(189.3; 474.5)<br>p=<.0001	14	360.0<br>(137.5; 582.5)<br>p=0.0022	-28.1<br>(-298.6; 242.5)<br>p=0.83	15	331.1<br>(169.1; 493.0)<br>p=0.0002	13	335.4<br>(94.7; 576.2)<br>p=0.0076	14	358.9<br>(136.4; 581.5)<br>p=0.0023	-27.9<br>(-310.4; 254.6)<br>p=0.84	-23.5<br>(-355.7; 308.6)<br>p=0.89
**Subgroup population according to the protocol for feces analyses excl outliers with non-missing value at any time point**	**W4**	23	194.9<br>(111.1; 278.7)<br>p=<.0001	10	141.5<br>(64.9; 218.1)<br>p=0.0007	53.4<br>(-59.6; 166.3)<br>p=0.34	13	212.0<br>(91.3; 332.8)<br>p=0.0012	10	171.9<br>(58.0; 285.8)<br>p=0.0044	10	141.0<br>(63.9; 218.2)<br>p=0.0008	71.0<br>(-72.3; 214.3)<br>p=0.32	30.9<br>(-105.9; 167.6)<br>p=0.65
	**W8**	23	55.0<br>(5.7; 104.3)<br>p=0.030	9	64.8<br>(-0.1; 129.8)<br>p=0.051	-9.8<br>(-90.5; 70.8)<br>p=0.81	12	28.9<br>(-45.3; 103.1)<br>p=0.43	11	85.5<br>(26.3; 144.6)<br>p=0.0061	9	64.5<br>(-0.4; 129.4)<br>p=0.051	-35.6<br>(-133.8; 62.6)<br>p=0.46	20.9<br>(-65.3; 107.2)<br>p=0.62
	**W10**	24	267.6<br>(150.9; 384.4)<br>p=<.0001	10	277.3<br>(95.3; 459.2)<br>p=0.0040	-9.6<br>(-226.4; 207.2)<br>p=0.93	14	322.2<br>(161.5; 482.9)<br>p=0.0003	10	195.5<br>(34.0; 356.9)<br>p=0.019	10	276.8<br>(94.4; 459.2)<br>p=0.0042	45.4<br>(-198.9; 289.8)<br>p=0.71	-81.3<br>(-325.0; 162.3)<br>p=0.50
**Subgroup population according to the protocol for feces analyses with complete cases at all time points**	**W4**	26	480.8<br>(158.6; 802.9)<br>p=0.0046	13	233.5<br>(29.5; 437.5)<br>p=0.026	247.2<br>(-140.6; 635.1)<br>p=0.20	13	320.3<br>(89.9; 550.6)<br>p=0.0078	13	641.7<br>(55.5; 1228.0)<br>p=0.033	13	233.0<br>(28.8; 437.2)<br>p=0.027	87.3<br>(-225.8; 400.4)<br>p=0.57	408.7<br>(-217.7;<br>1035.2)<br>p=0.19
	**W8**	26	112.0<br>(-36.4; 260.3)<br>p=0.13	13	159.2<br>(-29.8; 348.2)<br>p=0.096	-47.2<br>(-283.4; 188.9)<br>p=0.69	13	16.4<br>(-57.7; 90.5)<br>p=0.66	13	208.1<br>(-70.0; 486.1)<br>p=0.14	13	158.7<br>(-30.1; 347.5)<br>p=0.097	-142.3<br>(-342.5; 57.9)<br>p=0.16	49.4<br>(-283.0; 381.7)<br>p=0.76
	**W10**	26	341.6<br>(187.9; 495.2)<br>p=<.0001	13	355.1<br>(115.8; 594.4)<br>p=0.0048	-13.5<br>(-304.0; 277.0)<br>p=0.93	13	350.9<br>(166.9; 534.9)<br>p=0.0005	13	332.8<br>(89.8; 575.7)<br>p=0.0087	13	354.6<br>(115.1; 594.1)<br>p=0.0049	-3.7<br>(-312.5; 305.2)<br>p=0.98	-21.8<br>(-367.3; 323.7)<br>p=0.90
**Subgroup population according to the protocol for feces analyses excl outliers with complete cases at all time points**	**W4**	22	206.0<br>(119.4; 292.7)<br>p=<.0001	9	154.2<br>(77.4; 231.0)<br>p=0.0003	51.8<br>(-63.2; 166.9)<br>p=0.36	12	235.5<br>(109.0; 362.0)<br>p=0.0007	10	170.8<br>(57.5; 284.1)<br>p=0.0046	9	154.1<br>(77.1; 231.2)<br>p=0.0003	81.4<br>(-66.7; 229.4)<br>p=0.27	16.7<br>(-119.3; 152.7)<br>p=0.80

		MMRM comparing High+Low dose vs Placebo					MMRM comparing High dose vs Placebo and Low dose vs Placebo							
		High+Low dose		Placebo		High+Low dose vs Placebo	High dose		Low dose		Placebo		High dose vs Placebo	Low dose vs Placebo
Population	Time Point	n	LS mean (95% CI) p-value	n	LS mean (95% CI) p-value	Difference in LS mean (95% CI) p-value	n	LS mean (95% CI) p-value	n	LS mean (95% CI) p-value	n	LS mean (95% CI) p-value	Difference in LS mean (95% CI) p-value	Difference in LS mean (95% CI) p-value
	**W8**	22	46.4<br>(-6.5; 99.3)<br>p=0.083	9	66.4<br>(0.5; 132.2)<br>p=0.048	-19.9<br>(-103.5; 63.6)<br>p=0.63	12	21.3<br>(-57.4; 100.0)<br>p=0.58	10	76.7<br>(11.4; 142.0)<br>p=0.023	9	66.3<br>(0.4; 132.2)<br>p=0.049	-45.0<br>(-147.4; 57.4)<br>p=0.37	10.4<br>(-80.6; 101.4)<br>p=0.82
	**W10**	22	278.2<br>(150.0; 406.4)<br>p=0.0001	9	256.6<br>(57.4; 455.8)<br>p=0.014	21.7<br>(-215.6; 259.0)<br>p=0.85	12	344.6<br>(161.4; 527.9)<br>p=0.0007	10	198.6<br>(35.6; 361.6)<br>p=0.019	9	256.5<br>(56.9; 456.1)<br>p=0.014	88.2<br>(-183.7; 360.0)<br>p=0.51	-57.8<br>(-315.5; 199.8)<br>p=0.65
2020-01-02 Analys.sas														

Table 14.2.5.8 Posthoc Analyses - Isobutyrate - MMRM on various populations

		MMRM comparing High+Low dose vs Placebo					MMRM comparing High dose vs Placebo and Low dose vs Placebo							
		High+Low dose		Placebo		High+Low dose vs Placebo	High dose		Low dose		Placebo		High dose vs Placebo	Low dose vs Placebo
Population	Time Point	n	LS mean<br>(95% CI)<br>p-value	n	LS mean<br>(95% CI)<br>p-value	Difference in<br>LS mean<br>(95% CI)<br>p-value	n	LS mean<br>(95% CI)<br>p-value	n	LS mean<br>(95% CI)<br>p-value	n	LS mean<br>(95% CI)<br>p-value	Difference in<br>LS mean<br>(95% CI)<br>p-value	Difference in<br>LS mean<br>(95% CI)<br>p-value
ITT Population with non-missing value at any time point	W4	31	29.2<br>(10.5; 48.0)<br>p=0.0031	14	57.5<br>(0.7; 114.3)<br>p=0.048	-28.2<br>(-86.6; 30.1)<br>p=0.33	15	43.3<br>(16.1; 70.5)<br>p=0.0026	16	15.6<br>(-10.1; 41.3)<br>p=0.23	14	58.1<br>(1.0; 115.2)<br>p=0.046	-14.8<br>(-77.8; 48.1)<br>p=0.64	-42.5<br>(-103.1; 18.1)<br>p=0.16
	W8	30	20.2<br>(0.2; 40.2)<br>p=0.048	13	17.6<br>(-12.4; 47.5)<br>p=0.24	2.6<br>(-33.0; 38.1)<br>p=0.88	14	31.4<br>(-3.8; 66.6)<br>p=0.079	16	9.8<br>(-10.9; 30.6)<br>p=0.34	13	18.2<br>(-11.3; 47.8)<br>p=0.22	13.2<br>(-31.6; 58.0)<br>p=0.56	-8.4<br>(-45.0; 28.2)<br>p=0.65
	W10	32	31.1<br>(6.7; 55.5)<br>p=0.014	14	21.9<br>(-28.5; 72.3)<br>p=0.39	9.2<br>(-46.5; 64.9)<br>p=0.74	16	29.4<br>(-4.4; 63.2)<br>p=0.086	16	32.3<br>(-2.3; 66.9)<br>p=0.066	14	22.5<br>(-28.0; 73.1)<br>p=0.37	6.9<br>(-54.9; 68.7)<br>p=0.82	9.8<br>(-49.6; 69.2)<br>p=0.74
ITT Population excl outliers with non-missing value at any time point	W4	25	16.4<br>(-2.0; 34.7)<br>p=0.079	12	16.0<br>(-10.5; 42.5)<br>p=0.23	0.3<br>(-32.0; 32.7)<br>p=0.98	10	25.7<br>(1.8; 49.6)<br>p=0.036	15	10.3<br>(-15.1; 35.6)<br>p=0.42	12	16.0<br>(-10.6; 42.5)<br>p=0.23	9.7<br>(-26.5; 46.0)<br>p=0.59	-5.7<br>(-42.5; 31.1)<br>p=0.76
	W8	24	-5.5<br>(-20.8; 9.8)<br>p=0.47	11	11.8<br>(-15.7; 39.3)<br>p=0.39	-17.3<br>(-48.7; 14.1)<br>p=0.27	9	-12.6<br>(-31.4; 6.2)<br>p=0.18	15	-1.1<br>(-21.9; 19.7)<br>p=0.92	11	11.8<br>(-15.7; 39.3)<br>p=0.39	-24.4<br>(-57.8; 8.9)<br>p=0.15	-12.9<br>(-47.0; 21.2)<br>p=0.45
	W10	26	7.4<br>(-7.1; 22.0)<br>p=0.31	12	-10.3<br>(-28.9; 8.3)<br>p=0.27	17.7<br>(-6.2; 41.7)<br>p=0.14	11	4.4<br>(-10.6; 19.4)<br>p=0.56	15	9.7<br>(-12.8; 32.2)<br>p=0.39	12	-10.4<br>(-28.9; 8.2)<br>p=0.26	14.8<br>(-9.4; 38.9)<br>p=0.22	20.1<br>(-9.4; 49.5)<br>p=0.18
ITT Population with complete cases at all time points	W4	30	30.1<br>(10.7; 49.5)<br>p=0.0033	13	60.8<br>(-0.0; 121.7)<br>p=0.051	-30.8<br>(-93.1; 31.6)<br>p=0.32	14	45.9<br>(16.9; 74.9)<br>p=0.0028	16	15.8<br>(-9.8; 41.3)<br>p=0.22	13	61.5<br>(0.2; 122.8)<br>p=0.049	-15.6<br>(-83.1; 51.8)<br>p=0.64	-45.7<br>(-110.1; 18.7)<br>p=0.16
	W8	30	20.8<br>(0.6; 41.0)<br>p=0.044	13	18.7<br>(-11.6; 49.0)<br>p=0.22	2.1<br>(-33.8; 38.1)<br>p=0.91	14	32.5<br>(-3.6; 68.7)<br>p=0.076	16	10.0<br>(-10.7; 30.7)<br>p=0.33	13	19.3<br>(-10.6; 49.3)<br>p=0.20	13.2<br>(-32.7; 59.1)<br>p=0.56	-9.3<br>(-46.1; 27.4)<br>p=0.61
	W10	30	32.9<br>(6.9; 58.9)<br>p=0.014	13	25.9<br>(-27.7; 79.6)<br>p=0.33	7.0<br>(-52.2; 66.2)<br>p=0.81	14	32.9<br>(-5.5; 71.3)<br>p=0.091	16	32.5<br>(-2.2; 67.1)<br>p=0.065	13	26.6<br>(-27.4; 80.6)<br>p=0.32	6.3<br>(-60.8; 73.4)<br>p=0.85	5.9<br>(-56.3; 68.1)<br>p=0.85
ITT Population excl outliers with complete cases at all time points	W4	24	16.5<br>(-2.8; 35.7)<br>p=0.090	11	16.1<br>(-12.9; 45.0)<br>p=0.27	0.4<br>(-34.4; 35.2)<br>p=0.98	9	26.5<br>(-0.5; 53.4)<br>p=0.054	15	10.5<br>(-14.8; 35.9)<br>p=0.40	11	16.0<br>(-13.0; 45.0)<br>p=0.27	10.5<br>(-29.5; 50.4)<br>p=0.60	-5.5<br>(-44.0; 33.0)<br>p=0.77
	W8	24	-5.2<br>(-20.5; 10.0)<br>p=0.49	11	12.1<br>(-15.5; 39.6)<br>p=0.38	-17.3<br>(-48.7; 14.1)<br>p=0.27	9	-12.5<br>(-31.5; 6.5)<br>p=0.19	15	-0.9<br>(-21.7; 20.0)<br>p=0.93	11	12.0<br>(-15.5; 39.5)<br>p=0.38	-24.5<br>(-58.1; 9.1)<br>p=0.15	-12.9<br>(-47.0; 21.3)<br>p=0.45

		MMRM comparing High+Low dose vs Placebo					MMRM comparing High dose vs Placebo and Low dose vs Placebo							
		High+Low dose		Placebo		High+Low dose vs Placebo	High dose		Low dose		Placebo		High dose vs Placebo	Low dose vs Placebo
Population	Time Point	n	LS mean<br>(95% CI)<br>p-value	n	LS mean<br>(95% CI)<br>p-value	Difference in<br>LS mean<br>(95% CI)<br>p-value	n	LS mean<br>(95% CI)<br>p-value	n	LS mean<br>(95% CI)<br>p-value	n	LS mean<br>(95% CI)<br>p-value	Difference in<br>LS mean<br>(95% CI)<br>p-value	Difference in<br>LS mean<br>(95% CI)<br>p-value
	**W10**	24	7.9<br>(-8.0; 23.8)<br>p=0.32	11	-8.6<br>(-28.4; 11.1)<br>p=0.38	16.5<br>(-9.1; 42.1)<br>p=0.20	9	4.5<br>(-14.1; 23.0)<br>p=0.63	15	9.9<br>(-12.7; 32.5)<br>p=0.38	11	-8.7<br>(-28.4; 11.0)<br>p=0.37	13.1<br>(-14.1; 40.4)<br>p=0.33	18.6<br>(-11.5; 48.8)<br>p=0.22
**Subgroup population according to the protocol for feces analyses with non-missing value at any time point**	**W4**	28	30.0<br>(9.6; 50.5)<br>p=0.0051	14	54.0<br>(-2.5; 110.5)<br>p=0.061	-24.0<br>(-82.5; 34.6)<br>p=0.41	14	43.4<br>(12.8; 74.1)<br>p=0.0067	14	16.0<br>(-11.7; 43.7)<br>p=0.25	14	54.5<br>(-2.1; 111.1)<br>p=0.059	-11.0<br>(-74.9; 52.8)<br>p=0.73	-38.4<br>(-99.0; 22.1)<br>p=0.21
	**W8**	27	16.3<br>(-4.0; 36.7)<br>p=0.11	13	14.3<br>(-15.6; 44.2)<br>p=0.34	2.0<br>(-34.5; 38.6)<br>p=0.91	13	21.6<br>(-12.4; 55.6)<br>p=0.21	14	10.8<br>(-11.2; 32.8)<br>p=0.33	13	14.8<br>(-14.9; 44.5)<br>p=0.32	6.8<br>(-38.1; 51.7)<br>p=0.76	-4.0<br>(-41.5; 33.6)<br>p=0.83
	**W10**	29	17.1<br>(-3.3; 37.5)<br>p=0.098	14	18.4<br>(-31.5; 68.3)<br>p=0.46	-1.3<br>(-55.3; 52.7)<br>p=0.96	15	20.2<br>(-11.2; 51.5)<br>p=0.20	14	13.3<br>(-8.2; 34.9)<br>p=0.22	14	18.9<br>(-31.0; 68.8)<br>p=0.45	1.3<br>(-59.7; 62.3)<br>p=0.97	-5.5<br>(-57.2; 46.1)<br>p=0.83
**Subgroup population according to the protocol for feces analyses excl outliers with non-missing value at any time point**	**W4**	24	14.4<br>(-4.5; 33.2)<br>p=0.13	12	15.8<br>(-10.8; 42.4)<br>p=0.23	-1.5<br>(-34.2; 31.3)<br>p=0.93	10	25.4<br>(1.4; 49.3)<br>p=0.039	14	6.5<br>(-19.9; 32.9)<br>p=0.62	12	15.8<br>(-10.8; 42.4)<br>p=0.23	9.5<br>(-26.8; 45.9)<br>p=0.60	-9.3<br>(-46.9; 28.3)<br>p=0.62
	**W8**	23	-4.3<br>(-19.9; 11.4)<br>p=0.58	11	11.5<br>(-16.0; 38.9)<br>p=0.40	-15.7<br>(-47.3; 15.8)<br>p=0.32	9	-13.0<br>(-31.5; 5.6)<br>p=0.16	14	1.3<br>(-20.3; 22.9)<br>p=0.90	11	11.5<br>(-16.0; 38.9)<br>p=0.40	-24.4<br>(-57.6; 8.7)<br>p=0.14	-10.2<br>(-44.8; 24.4)<br>p=0.55
	**W10**	25	3.9<br>(-9.7; 17.5)<br>p=0.56	12	-10.5<br>(-29.2; 8.2)<br>p=0.26	14.4<br>(-9.0; 37.8)<br>p=0.22	11	4.0<br>(-10.8; 18.9)<br>p=0.58	14	3.8<br>(-17.4; 25.1)<br>p=0.72	12	-10.5<br>(-29.2; 8.2)<br>p=0.26	14.6<br>(-9.6; 38.7)<br>p=0.23	14.3<br>(-14.3; 42.9)<br>p=0.32
**Subgroup population according to the protocol for feces analyses with complete cases at all time points**	**W4**	27	30.8<br>(9.4; 52.1)<br>p=0.0060	13	57.5<br>(-3.0; 118.1)<br>p=0.062	-26.8<br>(-89.3; 35.8)<br>p=0.39	13	46.1<br>(13.1; 79.1)<br>p=0.0075	14	16.1<br>(-11.5; 43.6)<br>p=0.24	13	58.0<br>(-2.7; 118.8)<br>p=0.061	-11.9<br>(-80.5; 56.7)<br>p=0.73	-42.0<br>(-106.2; 22.2)<br>p=0.19
	**W8**	27	16.6<br>(-3.9; 37.1)<br>p=0.11	13	15.4<br>(-14.9; 45.6)<br>p=0.31	1.2<br>(-35.7; 38.2)<br>p=0.95	13	22.3<br>(-12.4; 57.0)<br>p=0.20	14	10.9<br>(-11.1; 32.8)<br>p=0.32	13	15.9<br>(-14.2; 45.9)<br>p=0.29	6.4<br>(-39.3; 52.1)<br>p=0.78	-5.0<br>(-42.7; 32.7)<br>p=0.79
	**W10**	27	18.0<br>(-3.8; 39.8)<br>p=0.10	13	22.6<br>(-30.5; 75.8)<br>p=0.39	-4.6<br>(-61.9; 52.7)<br>p=0.87	13	22.5<br>(-13.4; 58.5)<br>p=0.21	14	13.4<br>(-8.2; 35.0)<br>p=0.22	13	23.1<br>(-30.1; 76.4)<br>p=0.38	-0.6<br>(-66.8; 65.6)<br>p=0.99	-9.7<br>(-64.4; 45.0)<br>p=0.72
**Subgroup population according to the protocol for feces analyses excl outliers with complete cases at all time points**	**W4**	23	14.3<br>(-5.5; 34.1)<br>p=0.15	11	15.8<br>(-13.2; 44.9)<br>p=0.27	-1.5<br>(-36.6; 33.6)<br>p=0.93	9	26.1<br>(-0.9; 53.2)<br>p=0.057	14	6.8<br>(-19.6; 33.1)<br>p=0.60	11	15.8<br>(-13.2; 44.9)<br>p=0.27	10.3<br>(-29.8; 50.4)<br>p=0.60	-9.1<br>(-48.2; 30.1)<br>p=0.64

		MMRM comparing High+Low dose vs Placebo					MMRM comparing High dose vs Placebo and Low dose vs Placebo							
		High+Low dose		Placebo		High+Low dose vs Placebo	High dose		Low dose		Placebo		High dose vs Placebo	Low dose vs Placebo
**Population**	**Time Point**	**n**	**LS mean (95% CI) p-value**	**n**	**LS mean (95% CI) p-value**	**Difference in LS mean (95% CI) p-value**	**n**	**LS mean (95% CI) p-value**	**n**	**LS mean (95% CI) p-value**	**n**	**LS mean (95% CI) p-value**	**Difference in LS mean (95% CI) p-value**	**Difference in LS mean (95% CI) p-value**
	**W8**	23	-4.1 (-19.7; 11.6) p=0.60	11	11.8 (-15.7; 39.4) p=0.39	-15.9 (-47.6; 15.8) p=0.31	9	-12.8 (-31.8; 6.1) p=0.18	14	1.6 (-20.0; 23.2) p=0.88	11	11.8 (-15.8; 39.4) p=0.39	-24.7 (-58.3; 9.0) p=0.14	-10.2 (-44.9; 24.5) p=0.55
	**W10**	23	4.1 (-10.8; 19.0) p=0.58	11	-8.9 (-28.7; 11.0) p=0.37	13.0 (-12.1; 38.0) p=0.30	9	4.1 (-14.3; 22.5) p=0.65	14	4.1 (-17.3; 25.4) p=0.70	11	-8.9 (-28.7; 10.9) p=0.37	13.0 (-14.3; 40.3) p=0.34	13.0 (-16.3; 42.3) p=0.37
2020-01-02 Analys.sas														

Table 14.2.5.9 Posthoc Analyses - Isovalerate - MMRM on various populations

		MMRM comparing High+Low dose vs Placebo					MMRM comparing High dose vs Placebo and Low dose vs Placebo							
		High+Low dose		Placebo		High+Low dose vs Placebo	High dose		Low dose		Placebo		High dose vs Placebo	Low dose vs Placebo
Population	Time Point	n	LS mean (95% CI) p-value	n	LS mean (95% CI) p-value	Difference in LS mean (95% CI) p-value	n	LS mean (95% CI) p-value	n	LS mean (95% CI) p-value	n	LS mean (95% CI) p-value	Difference in LS mean (95% CI) p-value	Difference in LS mean (95% CI) p-value
ITT Population with non-missing value at any time point	W4	31	31.0 (11.8; 50.2) p=0.0023	14	78.2 (1.5; 154.9) p=0.046	-47.3 (-123.9; 29.4) p=0.22	15	47.0 (18.4; 75.7) p=0.0019	16	15.3 (-10.7; 41.3) p=0.24	14	79.0 (1.9; 156.2) p=0.045	-32.0 (-112.8; 48.7) p=0.43	-63.8 (-142.2; 14.6) p=0.11
	W8	30	17.3 (-2.6; 37.2) p=0.087	13	27.0 (-11.3; 65.2) p=0.16	-9.7 (-51.4; 32.1) p=0.64	14	29.0 (-2.4; 60.3) p=0.070	16	6.3 (-17.7; 30.4) p=0.60	13	27.6 (-10.5; 65.8) p=0.15	1.3 (-47.2; 49.8) p=0.96	-21.3 (-64.9; 22.3) p=0.33
	W10	32	29.3 (3.3; 55.4) p=0.028	14	47.2 (-34.5; 128.9) p=0.25	-17.8 (-102.2; 66.5) p=0.67	16	29.2 (-8.9; 67.4) p=0.13	16	28.8 (-4.8; 62.3) p=0.091	14	48.0 (-34.1; 130.0) p=0.24	-18.7 (-109.9; 72.4) p=0.68	-19.2 (-104.1; 65.7) p=0.65
ITT Population excl outliers with non-missing value at any time point	W4	28	23.5 (4.7; 42.2) p=0.016	12	19.8 (-4.8; 44.3) p=0.11	3.7 (-27.3; 34.7) p=0.81	13	35.0 (7.4; 62.5) p=0.014	15	13.4 (-11.2; 38.0) p=0.28	12	20.0 (-4.6; 44.5) p=0.11	15.0 (-22.6; 52.7) p=0.42	-6.6 (-41.2; 28.0) p=0.70
	W8	27	5.1 (-13.5; 23.6) p=0.58	11	9.1 (-17.1; 35.4) p=0.49	-4.1 (-36.7; 28.5) p=0.80	12	9.9 (-16.7; 36.4) p=0.46	15	1.1 (-23.6; 25.8) p=0.93	11	9.2 (-16.9; 35.4) p=0.48	0.6 (-37.2; 38.4) p=0.97	-8.1 (-44.3; 28.1) p=0.65
	W10	29	12.3 (-6.8; 31.4) p=0.20	12	-6.0 (-27.4; 15.3) p=0.57	18.3 (-10.4; 47.0) p=0.20	14	12.6 (-16.2; 41.4) p=0.38	15	11.8 (-13.7; 37.3) p=0.35	12	-5.9 (-27.1; 15.4) p=0.58	18.5 (-17.4; 54.4) p=0.30	17.6 (-15.6; 50.9) p=0.29
ITT Population with complete cases at all time points	W4	30	32.0 (12.1; 51.8) p=0.0023	13	81.1 (-2.2; 164.3) p=0.056	-49.1 (-132.3; 34.2) p=0.24	14	50.4 (20.3; 80.4) p=0.0016	16	15.3 (-10.7; 41.2) p=0.24	13	81.8 (-2.0; 165.7) p=0.056	-31.5 (-119.5; 56.6) p=0.47	-66.6 (-151.2; 18.1) p=0.12
	W8	30	17.8 (-2.2; 37.9) p=0.079	13	29.0 (-10.7; 68.8) p=0.15	-11.2 (-54.4; 32.0) p=0.60	14	30.3 (-2.0; 62.6) p=0.066	16	6.3 (-17.7; 30.3) p=0.60	13	29.8 (-9.9; 69.5) p=0.14	0.5 (-50.1; 51.0) p=0.99	-23.5 (-68.3; 21.2) p=0.29
	W10	30	31.0 (3.4; 58.6) p=0.029	13	54.3 (-33.5; 142.1) p=0.22	-23.3 (-113.9; 67.3) p=0.61	14	32.8 (-10.4; 76.0) p=0.13	16	28.7 (-4.9; 62.3) p=0.091	13	55.0 (-33.3; 143.4) p=0.21	-22.2 (-121.5; 77.1) p=0.65	-26.3 (-117.0; 64.4) p=0.56
ITT Population excl outliers with complete cases at all time points	W4	27	23.9 (4.4; 43.3) p=0.018	11	17.1 (-9.0; 43.3) p=0.19	6.7 (-25.7; 39.2) p=0.68	12	37.0 (7.5; 66.5) p=0.016	15	13.3 (-11.3; 38.0) p=0.28	11	17.3 (-8.8; 43.4) p=0.19	19.7 (-20.5; 59.8) p=0.33	-4.0 (-39.5; 31.5) p=0.82
	W8	27	5.3 (-13.4; 24.0) p=0.57	11	10.2 (-16.3; 36.7) p=0.44	-4.9 (-37.7; 28.0) p=0.77	12	10.5 (-17.5; 38.5) p=0.45	15	1.1 (-23.6; 25.8) p=0.93	11	10.4 (-16.0; 36.7) p=0.43	0.2 (-38.9; 39.2) p=0.99	-9.3 (-45.6; 27.1) p=0.61

		MMRM comparing High+Low dose vs Placebo					MMRM comparing High dose vs Placebo and Low dose vs Placebo							
		High+Low dose		Placebo		High+Low dose vs Placebo	High dose		Low dose		Placebo		High dose vs Placebo	Low dose vs Placebo
Population	Time Point	n	LS mean (95% CI) p-value	n	LS mean (95% CI) p-value	Difference in LS mean (95% CI) p-value	n	LS mean (95% CI) p-value	n	LS mean (95% CI) p-value	n	LS mean (95% CI) p-value	Difference in LS mean (95% CI) p-value	Difference in LS mean (95% CI) p-value
	**W10**	27	13.1<br>(-7.4; 33.7)<br>p=0.20	11	-3.1<br>(-25.2; 19.0)<br>p=0.78	16.2<br>(-13.9; 46.3)<br>p=0.28	12	14.7<br>(-18.9; 48.3)<br>p=0.38	15	11.7<br>(-13.8; 37.3)<br>p=0.36	11	-3.0<br>(-25.0; 19.1)<br>p=0.79	17.7<br>(-22.7; 58.0)<br>p=0.38	14.7<br>(-18.9; 48.3)<br>p=0.38
**Subgroup population according to the protocol for feces analyses with non-missing value at any time point**	**W4**	28	32.8<br>(12.5; 53.1)<br>p=0.0023	14	77.1<br>(-0.1; 154.2)<br>p=0.051	-44.2<br>(-121.3; 32.8)<br>p=0.25	14	49.9<br>(19.8; 79.9)<br>p=0.0018	14	14.8<br>(-13.8; 43.3)<br>p=0.30	14	77.9<br>(0.4; 155.3)<br>p=0.049	-28.0<br>(-109.5; 53.5)<br>p=0.49	-63.1<br>(-142.0; 15.8)<br>p=0.11
	**W8**	27	16.4<br>(-4.8; 37.6)<br>p=0.13	13	25.6<br>(-12.8; 63.9)<br>p=0.19	-9.2<br>(-51.6; 33.3)<br>p=0.66	13	24.3<br>(-8.6; 57.3)<br>p=0.14	14	8.0<br>(-19.3; 35.2)<br>p=0.56	13	26.3<br>(-12.0; 64.6)<br>p=0.17	-1.9<br>(-51.4; 47.5)<br>p=0.94	-18.3<br>(-63.8; 27.2)<br>p=0.42
	**W10**	29	16.4<br>(-7.6; 40.4)<br>p=0.18	14	46.0<br>(-36.2; 128.1)<br>p=0.26	-29.6<br>(-113.8; 54.6)<br>p=0.48	15	20.8<br>(-16.5; 58.0)<br>p=0.27	14	10.9<br>(-14.9; 36.7)<br>p=0.40	14	46.8<br>(-35.6; 129.2)<br>p=0.26	-26.0<br>(-117.5; 65.4)<br>p=0.57	-35.9<br>(-117.7; 45.9)<br>p=0.38
**Subgroup population according to the protocol for feces analyses excl outliers with non-missing value at any time point**	**W4**	26	23.1<br>(3.3; 42.9)<br>p=0.024	12	18.3<br>(-6.2; 42.7)<br>p=0.14	4.8<br>(-26.5; 36.1)<br>p=0.76	12	37.7<br>(8.5; 66.9)<br>p=0.013	14	10.5<br>(-15.0; 36.0)<br>p=0.41	12	18.5<br>(-5.9; 42.9)<br>p=0.13	19.2<br>(-19.1; 57.5)<br>p=0.32	-8.0<br>(-43.3; 27.2)<br>p=0.65
	**W8**	25	3.6<br>(-15.5; 22.6)<br>p=0.71	11	7.7<br>(-18.7; 34.2)<br>p=0.56	-4.2<br>(-37.5; 29.2)<br>p=0.80	11	3.4<br>(-22.5; 29.4)<br>p=0.79	14	3.7<br>(-22.2; 29.6)<br>p=0.78	11	7.9<br>(-18.5; 34.2)<br>p=0.55	-4.5<br>(-42.5; 33.6)<br>p=0.81	-4.2<br>(-41.5; 33.0)<br>p=0.82
	**W10**	27	4.5<br>(-13.0; 22.0)<br>p=0.60	12	-7.5<br>(-28.7; 13.6)<br>p=0.48	12.0<br>(-16.0; 40.0)<br>p=0.39	13	2.1<br>(-21.0; 25.1)<br>p=0.86	14	6.6<br>(-18.7; 31.9)<br>p=0.60	12	-7.3<br>(-28.4; 13.8)<br>p=0.49	9.4<br>(-22.7; 41.5)<br>p=0.56	13.9<br>(-19.2; 47.0)<br>p=0.40
**Subgroup population according to the protocol for feces analyses with complete cases at all time points**	**W4**	27	33.7<br>(12.6; 54.9)<br>p=0.0026	13	79.9<br>(-3.9; 163.7)<br>p=0.061	-46.1<br>(-130.0; 37.7)<br>p=0.27	13	53.5<br>(21.7; 85.3)<br>p=0.0016	14	14.7<br>(-13.8; 43.2)<br>p=0.30	13	80.7<br>(-3.6; 164.9)<br>p=0.060	-27.2<br>(-116.3; 62.0)<br>p=0.54	-66.0<br>(-151.1; 19.2)<br>p=0.12
	**W8**	27	16.7<br>(-4.7; 38.1)<br>p=0.12	13	27.9<br>(-12.2; 67.9)<br>p=0.17	-11.2<br>(-55.2; 32.8)<br>p=0.61	13	25.4<br>(-8.6; 59.4)<br>p=0.14	14	7.9<br>(-19.4; 35.1)<br>p=0.56	13	28.7<br>(-11.3; 68.7)<br>p=0.15	-3.3<br>(-55.0; 48.4)<br>p=0.90	-20.8<br>(-67.4; 25.8)<br>p=0.37
	**W10**	27	17.2<br>(-8.3; 42.8)<br>p=0.18	13	53.1<br>(-35.2; 141.4)<br>p=0.23	-35.9<br>(-126.4; 54.6)<br>p=0.43	13	23.4<br>(-19.2; 66.0)<br>p=0.27	14	10.8<br>(-15.0; 36.6)<br>p=0.40	13	53.9<br>(-34.8; 142.6)<br>p=0.23	-30.5<br>(-130.4; 69.4)<br>p=0.54	-43.1<br>(-130.8; 44.7)<br>p=0.33
**Subgroup population according to the protocol for feces analyses excl outliers with complete cases at all time points**	**W4**	25	23.5<br>(2.8; 44.1)<br>p=0.027	11	15.7<br>(-10.4; 41.8)<br>p=0.23	7.8<br>(-25.2; 40.8)<br>p=0.63	11	40.0<br>(8.6; 71.4)<br>p=0.014	14	10.4<br>(-15.2; 35.9)<br>p=0.41	11	15.9<br>(-10.2; 42.0)<br>p=0.22	24.1<br>(-17.0; 65.2)<br>p=0.24	-5.5<br>(-41.7; 30.6)<br>p=0.76

		MMRM comparing High+Low dose vs Placebo					MMRM comparing High dose vs Placebo and Low dose vs Placebo							
		High+Low dose		Placebo		High+Low dose vs Placebo	High dose		Low dose		Placebo		High dose vs Placebo	Low dose vs Placebo
Population	Time Point	n	LS mean (95% CI) p-value	n	LS mean (95% CI) p-value	Difference in LS mean (95% CI) p-value	n	LS mean (95% CI) p-value	n	LS mean (95% CI) p-value	n	LS mean (95% CI) p-value	Difference in LS mean (95% CI) p-value	Difference in LS mean (95% CI) p-value
	**W8**	25	3.6 (-15.6; 22.8) p=0.70	11	8.7 (-18.0; 35.5) p=0.51	-5.1 (-38.8; 28.5) p=0.76	11	3.4 (-23.6; 30.5) p=0.80	14	3.6 (-22.4; 29.5) p=0.78	11	8.9 (-17.7; 35.6) p=0.50	-5.5 (-44.6; 33.6) p=0.78	-5.4 (-42.8; 32.0) p=0.77
	**W10**	25	4.8 (-14.1; 23.7) p=0.61	11	-4.6 (-26.4; 17.3) p=0.67	9.4 (-19.9; 38.6) p=0.52	11	2.4 (-24.8; 29.6) p=0.86	14	6.5 (-18.9; 31.9) p=0.61	11	-4.4 (-26.2; 17.5) p=0.69	6.8 (-28.9; 42.5) p=0.70	10.9 (-22.5; 44.3) p=0.51
2020-01-02 Analys.sas														

Table 14.2.5.10 Posthoc Analyses - Succinate - MMRM on various populations

		MMRM comparing High+Low dose vs Placebo					MMRM comparing High dose vs Placebo and Low dose vs Placebo							
		High+Low dose		Placebo		High+Low dose vs Placebo	High dose		Low dose		Placebo		High dose vs Placebo	Low dose vs Placebo
**Population**	**Time Point**	**n**	**LS mean (95% CI) p-value**	**n**	**LS mean (95% CI) p-value**	**Difference in LS mean (95% CI) p-value**	**n**	**LS mean (95% CI) p-value**	**n**	**LS mean (95% CI) p-value**	**n**	**LS mean (95% CI) p-value**	**Difference in LS mean (95% CI) p-value**	**Difference in LS mean (95% CI) p-value**
**ITT Population with non-missing value at any time point**	**W4**	31	5.6<br>(-26.7; 37.9)<br>p=0.73	14	-19.1<br>(-64.2; 25.9)<br>p=0.40	24.7<br>(-28.2; 77.6)<br>p=0.35	15	28.7<br>(-30.7; 88.1)<br>p=0.33	16	-16.4<br>(-49.3; 16.4)<br>p=0.32	14	-18.7<br>(-64.0; 26.6)<br>p=0.41	47.4<br>(-22.9; 117.7)<br>p=0.18	2.3<br>(-55.3; 59.8)<br>p=0.94
	**W8**	30	262.4<br>(-83.7; 608.6)<br>p=0.13	13	-8.7<br>(-31.7; 14.3)<br>p=0.45	271.1<br>(-73.0; 615.2)<br>p=0.12	14	174.5<br>(-77.1; 426.1)<br>p=0.17	16	337.1<br>(-270.8; 945.1)<br>p=0.27	13	-8.6<br>(-32.0; 14.7)<br>p=0.46	183.1<br>(-60.5; 426.7)<br>p=0.14	345.8<br>(-262.8; 954.3)<br>p=0.26
	**W10**	32	19.6<br>(-32.9; 72.2)<br>p=0.46	14	6.3<br>(-48.0; 60.6)<br>p=0.82	13.4<br>(-61.8; 88.5)<br>p=0.72	16	19.0<br>(-33.1; 71.1)<br>p=0.47	16	19.9<br>(-72.0; 111.8)<br>p=0.66	14	6.7<br>(-47.9; 61.3)<br>p=0.80	12.3<br>(-65.4; 89.9)<br>p=0.75	13.2<br>(-91.9; 118.2)<br>p=0.80
**ITT Population excl outliers with non-missing value at any time point**	**W4**	26	-5.7<br>(-36.7; 25.3)<br>p=0.71	12	-50.5<br>(-66.9; -34.2)<br>p=<.0001	44.8<br>(10.1; 79.6)<br>p=0.013	13	1.8<br>(-48.6; 52.2)<br>p=0.94	13	-13.9<br>(-49.6; 21.8)<br>p=0.44	12	-50.3<br>(-66.8; -33.9)<br>p=<.0001	52.1<br>(-0.3; 104.5)<br>p=0.051	36.5<br>(-3.3; 76.2)<br>p=0.071
	**W8**	25	17.7<br>(-6.0; 41.4)<br>p=0.14	11	-19.8<br>(-42.9; 3.3)<br>p=0.090	37.5<br>(4.4; 70.7)<br>p=0.028	12	19.7<br>(-16.1; 55.5)<br>p=0.27	13	15.2<br>(-17.7; 48.2)<br>p=0.35	11	-19.5<br>(-42.6; 3.6)<br>p=0.095	39.2<br>(-2.2; 80.5)<br>p=0.062	34.7<br>(-7.0; 76.5)<br>p=0.10
	**W10**	27	-6.2<br>(-28.1; 15.6)<br>p=0.57	12	-21.5<br>(-62.6; 19.6)<br>p=0.30	15.3<br>(-31.7; 62.3)<br>p=0.51	14	4.9<br>(-29.7; 39.4)<br>p=0.78	13	-18.4<br>(-42.4; 5.6)<br>p=0.13	12	-21.3<br>(-62.5; 19.9)<br>p=0.30	26.2<br>(-28.4; 80.7)<br>p=0.34	2.9<br>(-44.9; 50.7)<br>p=0.90
**ITT Population with complete cases at all time points**	**W4**	30	0.2<br>(-31.2; 31.6)<br>p=0.99	13	-15.6<br>(-62.8; 31.6)<br>p=0.51	15.8<br>(-38.1; 69.7)<br>p=0.56	14	18.3<br>(-41.9; 78.4)<br>p=0.54	16	-15.8<br>(-48.8; 17.2)<br>p=0.34	13	-15.3<br>(-62.8; 32.1)<br>p=0.52	33.6<br>(-37.8; 105.0)<br>p=0.35	-0.5<br>(-59.6; 58.7)<br>p=0.99
	**W8**	30	254.3<br>(-93.0; 601.7)<br>p=0.15	13	-5.6<br>(-30.0; 18.8)<br>p=0.65	259.9<br>(-85.5; 605.3)<br>p=0.14	14	158.7<br>(-101.0; 418.5)<br>p=0.22	16	337.8<br>(-271.7; 947.2)<br>p=0.27	13	-5.3<br>(-29.8; 19.2)<br>p=0.66	164.1<br>(-87.9; 416.0)<br>p=0.20	343.1<br>(-266.9; 953.1)<br>p=0.26
	**W10**	30	15.4<br>(-40.5; 71.3)<br>p=0.58	13	10.9<br>(-46.4; 68.2)<br>p=0.70	4.5<br>(-75.2; 84.3)<br>p=0.91	14	9.4<br>(-48.0; 66.8)<br>p=0.74	16	20.5<br>(-71.8; 112.8)<br>p=0.65	13	11.2<br>(-46.4; 68.7)<br>p=0.70	-1.8<br>(-85.1; 81.5)<br>p=0.97	9.4<br>(-97.8; 116.6)<br>p=0.86
**ITT Population excl outliers with complete cases at all time points**	**W4**	25	-13.6<br>(-42.2; 15.1)<br>p=0.34	11	-49.7<br>(-67.3; -32.2)<br>p=<.0001	36.2<br>(2.7; 69.7)<br>p=0.035	12	-13.9<br>(-60.2; 32.4)<br>p=0.54	13	-13.3<br>(-49.1; 22.6)<br>p=0.46	11	-49.7<br>(-67.3; -32.0)<br>p=<.0001	35.7<br>(-12.8; 84.3)<br>p=0.14	36.4<br>(-4.1; 76.8)<br>p=0.076
	**W8**	25	14.9<br>(-8.5; 38.3)<br>p=0.20	11	-18.7<br>(-42.0; 4.6)<br>p=0.11	33.6<br>(0.4; 66.7)<br>p=0.047	12	13.8<br>(-21.4; 49.0)<br>p=0.43	13	15.8<br>(-17.5; 49.1)<br>p=0.34	11	-18.6<br>(-42.0; 4.8)<br>p=0.11	32.4<br>(-8.3; 73.2)<br>p=0.11	34.4<br>(-7.9; 76.6)<br>p=0.11

		MMRM comparing High+Low dose vs Placebo					MMRM comparing High dose vs Placebo and Low dose vs Placebo							
		High+Low dose		Placebo		High+Low dose vs Placebo	High dose		Low dose		Placebo		High dose vs Placebo	Low dose vs Placebo
Population	Time Point	n	LS mean (95% CI) p-value	n	LS mean (95% CI) p-value	Difference in LS mean (95% CI) p-value	n	LS mean (95% CI) p-value	n	LS mean (95% CI) p-value	n	LS mean (95% CI) p-value	Difference in LS mean (95% CI) p-value	Difference in LS mean (95% CI) p-value
	**W10**	25	-13.3<br>(-33.9; 7.4)<br>p=0.20	11	-19.1<br>(-63.9; 25.6)<br>p=0.39	5.9<br>(-43.9; 55.6)<br>p=0.81	12	-8.4<br>(-42.0; 25.2)<br>p=0.61	13	-17.8<br>(-41.7; 6.1)<br>p=0.14	11	-19.0<br>(-63.9; 25.8)<br>p=0.39	10.6<br>(-45.9; 67.2)<br>p=0.70	1.2<br>(-49.9; 52.3)<br>p=0.96
**Subgroup population according to the protocol for feces analyses with non-missing value at any time point**	**W4**	28	-0.6<br>(-34.0; 32.8)<br>p=0.97	14	-21.5<br>(-66.0; 23.0)<br>p=0.33	20.9<br>(-31.7; 73.5)<br>p=0.43	14	21.0<br>(-42.1; 84.0)<br>p=0.50	14	-22.7<br>(-54.6; 9.3)<br>p=0.16	14	-20.8<br>(-65.8; 24.2)<br>p=0.35	41.8<br>(-29.9; 113.6)<br>p=0.25	-1.8<br>(-59.2; 55.6)<br>p=0.95
	**W8**	27	293.8<br>(-90.1; 677.8)<br>p=0.13	13	-11.7<br>(-34.6; 11.1)<br>p=0.31	305.6<br>(-76.0; 687.1)<br>p=0.11	13	186.2<br>(-85.2; 457.5)<br>p=0.17	14	390.5<br>(-301.0;<br>1082.0)<br>p=0.26	13	-11.5<br>(-35.0; 12.1)<br>p=0.33	197.6<br>(-63.8; 459.1)<br>p=0.13	402.0<br>(-290.0;<br>1094.0)<br>p=0.25
	**W10**	29	28.4<br>(-28.5; 85.4)<br>p=0.32	14	3.9<br>(-50.0; 57.9)<br>p=0.88	24.5<br>(-53.4; 102.5)<br>p=0.53	15	25.2<br>(-28.1; 78.6)<br>p=0.34	14	31.2<br>(-72.6; 135.1)<br>p=0.55	14	4.6<br>(-49.9; 59.0)<br>p=0.87	20.7<br>(-57.4; 98.7)<br>p=0.60	26.7<br>(-89.3; 142.7)<br>p=0.64
**Subgroup population according to the protocol for feces analyses excl outliers with non-missing value at any time point**	**W4**	23	-15.5<br>(-44.8; 13.7)<br>p=0.29	12	-51.0<br>(-67.0; -34.9)<br>p=<.0001	35.4<br>(1.6; 69.3)<br>p=0.041	12	-11.7<br>(-57.8; 34.5)<br>p=0.61	11	-20.0<br>(-53.8; 13.7)<br>p=0.24	12	-51.0<br>(-67.1; -35.0)<br>p=<.0001	39.4<br>(-9.5; 88.3)<br>p=0.11	31.0<br>(-6.9; 68.9)<br>p=0.11
	**W8**	22	16.7<br>(-6.4; 39.9)<br>p=0.15	11	-20.1<br>(-43.2; 2.9)<br>p=0.084	36.9<br>(3.6; 70.1)<br>p=0.031	11	7.1<br>(-22.7; 37.0)<br>p=0.63	11	25.9<br>(-8.9; 60.6)<br>p=0.14	11	-20.3<br>(-43.5; 2.9)<br>p=0.084	27.4<br>(-9.7; 64.6)<br>p=0.14	46.2<br>(3.0; 89.3)<br>p=0.037
	**W10**	24	0.1<br>(-22.9; 23.2)<br>p=0.99	12	-21.9<br>(-63.3; 19.4)<br>p=0.29	22.1<br>(-25.4; 69.6)<br>p=0.35	13	8.5<br>(-28.0; 45.0)<br>p=0.64	11	-9.6<br>(-34.4; 15.2)<br>p=0.43	12	-22.0<br>(-63.4; 19.3)<br>p=0.29	30.5<br>(-24.7; 85.8)<br>p=0.27	12.4<br>(-35.9; 60.7)<br>p=0.60
**Subgroup population according to the protocol for feces analyses with complete cases at all time points**	**W4**	27	-6.7<br>(-38.9; 25.5)<br>p=0.67	13	-18.0<br>(-64.6; 28.6)<br>p=0.44	11.3<br>(-42.0; 64.6)<br>p=0.67	13	9.2<br>(-55.0; 73.3)<br>p=0.77	14	-21.8<br>(-53.9; 10.2)<br>p=0.18	13	-17.6<br>(-64.6; 29.4)<br>p=0.45	26.8<br>(-46.3; 99.9)<br>p=0.46	-4.2<br>(-63.2; 54.7)<br>p=0.88
	**W8**	27	281.0<br>(-104.4; 666.4)<br>p=0.15	13	-8.0<br>(-31.8; 15.8)<br>p=0.50	289.0<br>(-94.2; 672.1)<br>p=0.13	13	161.7<br>(-120.4; 443.9)<br>p=0.25	14	391.3<br>(-302.1;<br>1084.8)<br>p=0.26	13	-7.6<br>(-31.8; 16.6)<br>p=0.53	169.3<br>(-103.6; 442.3)<br>p=0.22	398.9<br>(-295.1;<br>1092.9)<br>p=0.25
	**W10**	27	24.5<br>(-36.7; 85.7)<br>p=0.42	13	8.5<br>(-48.4; 65.4)<br>p=0.76	16.0<br>(-67.2; 99.2)<br>p=0.70	13	15.9<br>(-43.6; 75.5)<br>p=0.59	14	32.1<br>(-72.3; 136.5)<br>p=0.54	13	8.9<br>(-48.4; 66.2)<br>p=0.75	7.1<br>(-77.2; 91.3)<br>p=0.87	23.2<br>(-94.9; 141.2)<br>p=0.69
**Subgroup population according to the protocol for feces analyses excl outliers with complete cases at all time points**	**W4**	22	-25.3<br>(-50.3; -0.4)<br>p=0.047	11	-49.8<br>(-67.1; -32.5)<br>p=<.0001	24.5<br>(-6.5; 55.6)<br>p=0.12	11	-30.5<br>(-67.3; 6.2)<br>p=0.099	11	-19.6<br>(-53.3; 14.1)<br>p=0.24	11	-50.3<br>(-67.6; -33.1)<br>p=<.0001	19.8<br>(-21.1; 60.6)<br>p=0.33	30.7<br>(-7.7; 69.1)<br>p=0.11

		MMRM comparing High+Low dose vs Placebo					MMRM comparing High dose vs Placebo and Low dose vs Placebo							
		High+Low dose		Placebo		High+Low dose vs Placebo	High dose		Low dose		Placebo		High dose vs Placebo	Low dose vs Placebo
Population	Time Point	n	LS mean (95% CI) p-value	n	LS mean (95% CI) p-value	Difference in LS mean (95% CI) p-value	n	LS mean (95% CI) p-value	n	LS mean (95% CI) p-value	n	LS mean (95% CI) p-value	Difference in LS mean (95% CI) p-value	Difference in LS mean (95% CI) p-value
	**W8**	22	13.5 (-9.3; 36.3) p=0.24	11	-18.8 (-42.0; 4.4) p=0.11	32.3 (-0.8; 65.5) p=0.056	11	1.2 (-26.9; 29.4) p=0.93	11	26.3 (-9.0; 61.6) p=0.14	11	-19.3 (-42.7; 4.1) p=0.10	20.5 (-15.7; 56.7) p=0.26	45.6 (1.9; 89.3) p=0.041
	**W10**	22	-7.5 (-29.4; 14.5) p=0.49	11	-19.2 (-64.5; 26.0) p=0.39	11.8 (-38.9; 62.4) p=0.64	11	-5.2 (-41.3; 30.9) p=0.77	11	-9.2 (-33.7; 15.3) p=0.45	11	-19.7 (-64.9; 25.5) p=0.38	14.5 (-43.4; 72.4) p=0.61	10.5 (-41.2; 62.3) p=0.68
2020-01-02 Analys.sas														

Table 14.2.6.1 Secondary Analysis - Colonization with F. prausnitzii and D. Piger in stool (ITT Population)

					Difference and test between groups		
**Variable**	**High+Low dose (n=34)**	**High dose (n=18)**	**Low dose (n=16)**	**Placebo (n=16)**	**High+Low dose vs Placebo**	**High dose vs Placebo**	**Low dose vs Placebo**
**Colonization with F. prausnitzii in stool SL3.3 at randomization**	399113 (300581) 261943 (64423; 1322383) n=30	486085 (381409) 386948 (87115; 1322383) n=14	323012 (188047) 250282 (64423; 648169) n=16	447790 (427354) 344404 (65739; 1495280) n=13			
**Colonization with F. prausnitzii in stool SL3.3 at week 8**	320272 (227638) 268179 (47263; 1068229) n=30	357639 (276514) 268179 (73590; 1068229) n=14	287577 (177410) 273553 (47263; 589660) n=16	370992 (257997) 337055 (48369; 772443) n=13			
**Change in Colonization with F. prausnitzii in stool SL3.3 from randomization to week 8**	-78841 (297566) -36366 (-934033; 557397) n=30	-128447 (417713) -24407 (-934033; 557397) n=14	-35435 (125147) -50520 (-314365; 247943) n=16	-76798 (313430) -4527 (-1027009; 285842) n=13	-2043 (-217060; 188801)	-51649 (-342949; 237378)	41363 (-119126; 209031)
**Colonization with F. prausnitzii in stool L2.6 at randomization**	445500 (455431) 296051 (97458; 2395226) n=30	473748 (604906) 282870 (97458; 2395226) n=14	420783 (287205) 298545 (155219; 1110434) n=16	407073 (270661) 334194 (105446; 910201) n=13			
**Colonization with F. prausnitzii in stool L2.6 at week 8**	397702 (219729) 362098 (19808; 908861) n=30	372734 (266605) 296784 (19808; 908861) n=14	419550 (175074) 394096 (142853; 738990) n=16	415627 (310731) 314303 (69199; 1164523) n=13			
**Change in Colonization with F. prausnitzii in stool L2.6 from randomization to week 8**	-47798 (362123) 15685 (-1486365; 350957) n=30	-101014 (445470) 2744 (-1486365; 350957) n=14	-1233 (276738) 32574 (-774315; 322342) n=16	8554 (162939) -14273 (-326705; 351161) n=13	-56352 (-282598; 123781)	-109569 (-372257; 121404)	-9787 (-189735; 159805)
**Colonization with F. prausnitzii in stool KLE1255 at randomization**	421180 (455384) 317422 (56316; 2104040) n=30	388569 (440720) 298151 (56316; 1795932) n=14	449714 (480340) 325233 (64786; 2104040) n=16	462245 (308874) 349653 (147476; 1214343) n=13			
**Colonization with F. prausnitzii in stool KLE1255 at week 8**	380187 (304637) 312475 (11004; 1336302) n=30	387879 (368884) 276252 (11004; 1336302) n=14	373456 (247759) 338288 (117256; 1091759) n=16	552283 (406738) 346611 (104113; 1321771) n=13			
**Change in Colonization with F. prausnitzii in stool KLE1255 from randomization to week 8**	-40993 (270906) -38455 (-1012281; 409225) n=30	-690 (212672) 17596 (-459630; 390499) n=14	-76258 (315984) -47318 (-1012281; 409225) n=16	90037 (204623) 18813 (-183783; 508825) n=13	-131030 (-304241; 26987)	-90727 (-256195; 75252)	-166296 (-373715; 30226)

					Difference and test between groups		
**Variable**	**High+Low dose (n=34)**	**High dose (n=18)**	**Low dose (n=16)**	**Placebo (n=16)**	**High+Low dose vs Placebo**	**High dose vs Placebo**	**Low dose vs Placebo**
**Colonization with F. prausnitzii in stool A2.165 at randomization**	475369 (558059)<br>282274 (68859; 2981157)<br>n=30	406020 (320415)<br>275428 (117096; 1027944)<br>n=14	536050 (710426)<br>292417 (68859; 2981157)<br>n=16	447652 (240108)<br>515762 (108885; 882020)<br>n=13			
**Colonization with F. prausnitzii in stool A2.165 at week 8**	487469 (508402)<br>273623 (54363; 1843881)<br>n=30	500360 (518283)<br>256521 (54363; 1806828)<br>n=14	476189 (516354)<br>279135 (65977; 1843881)<br>n=16	483607 (307096)<br>438861 (126888; 1396880)<br>n=13			
**Change in Colonization with F. prausnitzii in stool A2.165 from randomization to week 8**	12100 (312100)<br>-6637 (-1137276; 801222)<br>n=30	94340 (266990)<br>21030 (-174526; 801222)<br>n=14	-59860 (338679)<br>-18954 (-1137276; 557233)<br>n=16	35955 (200955)<br>7182 (-235870; 514860)<br>n=13	-23855 (-216967; 155763)	58386 (-123416; 247130)	-95815 (-311070; 103564)
**Colonization with F. prausnitzii in stool Total at randomization**	1741162 (1032122)<br>1492610 (503848; 4691391)<br>n=30	1754422 (1020550)<br>1492610 (532972; 4691391)<br>n=14	1729559 (1075441)<br>1500193 (503848; 4531826)<br>n=16	1764760 (783823)<br>1791817 (533480; 3106899)<br>n=13			
**Colonization with F. prausnitzii in stool Total at week 8**	1585630 (844839)<br>1285484 (166081; 3263089)<br>n=30	1618611 (951827)<br>1518390 (166081; 3263089)<br>n=14	1556772 (769962)<br>1285484 (631767; 3072278)<br>n=16	1822508 (689936)<br>1846956 (718809; 2905463)<br>n=13			
**Change in Colonization with F. prausnitzii in stool Total from randomization to week 8**	-155531 (736061)<br>-115538 (-1719439; 1666978)<br>n=30	-135811 (784580)<br>-83617 (-1719439; 1666978)<br>n=14	-172787 (716436)<br>-142049 (-1516829; 974435)<br>n=16	57748 (455050)<br>-29279 (-809756; 819246)<br>n=13	-213280 (-658418; 224028)<br>p=0.34	-193559 (-694598; 310230)<br>p=0.46	-230535 (-696633; 230268)<br>p=0.33
**Colonization with D. Piger in stool at randomization**	9389 (17874)<br>3 (0; 56828)<br>n=30	11651 (19326)<br>3 (0; 49271)<br>n=14	7410 (16882)<br>4 (0; 56828)<br>n=16	9706 (27312)<br>4 (0; 96395)<br>n=13			
**Colonization with D. Piger in stool at week 8**	20769 (36161)<br>11 (0; 154186)<br>n=30	38760 (46093)<br>32378 (0; 154186)<br>n=14	5026 (10966)<br>8 (0; 29886)<br>n=16	9481 (29229)<br>5 (0; 105371)<br>n=13			
**Change in Colonization with D. Piger in stool from randomization to week 8**	11379 (34585)<br>3 (-26942; 154183)<br>n=30	27110 (45986)<br>3401 (-13899; 154183)<br>n=14	-2384 (6838)<br>1 (-26942; 14)<br>n=16	-225.5 (4300.6)<br>1 (-11918; 8976)<br>n=13	11605 (-3398; 32526)	27335 (3893; 53604)	-2159 (-6733; 1704)

For continuous variables Mean (SD) / Median (Min; Max) / n= is presented.
For comparison between groups the Fisher´s Non Parametric Permutation Test was used for continuous variables. The confidence interval for then mean difference between groups is based on Fishers non-parametric permutation test.
2020-01-01 Analys.sas

Table 14.2.6.2 Secondary Analysis - Colonization with F. prausnitzii and D. Piger in stool (PP Population)

					Difference and test between groups		
**Variable**	**High+Low dose (n=33)**	**High dose (n=18)**	**Low dose (n=15)**	**Placebo (n=16)**	**High+Low dose vs Placebo**	**High dose vs Placebo**	**Low dose vs Placebo**
**Colonization with F. prausnitzii in stool SL3.3 at randomization**	404008 (304682) 266737 (64423; 1322383) n=29	486085 (381409) 386948 (87115; 1322383) n=14	327403 (193796) 243415 (64423; 648169) n=15	447790 (427354) 344404 (65739; 1495280) n=13			
**Colonization with F. prausnitzii in stool SL3.3 at week 8**	313899 (228927) 229458 (47263; 1068229) n=29	357639 (276514) 268179 (73590; 1068229) n=14	273076 (173544) 213301 (47263; 589660) n=15	370992 (257997) 337055 (48369; 772443) n=13			
**Change in Colonization with F. prausnitzii in stool SL3.3 from randomization to week 8**	-90109 (296247) -38353 (-934033; 557397) n=29	-128447 (417713) -24407 (-934033; 557397) n=14	-54327 (103258) -53436 (-314365; 151473) n=15	-76798 (313430) -4527 (-1027009; 285842) n=13	-13311 (-225569; 177506)	-51649 (-342949; 237378)	22471 (-132260; 187996)
**Colonization with F. prausnitzii in stool L2.6 at randomization**	450817 (462543) 300793 (97458; 2395226) n=29	473748 (604906) 282870 (97458; 2395226) n=14	429414 (295129) 305780 (155219; 1110434) n=15	407073 (270661) 334194 (105446; 910201) n=13			
**Colonization with F. prausnitzii in stool L2.6 at week 8**	392863 (221985) 347839 (19808; 908861) n=29	372734 (266605) 296784 (19808; 908861) n=14	411650 (178242) 347839 (142853; 738990) n=15	415627 (310731) 314303 (69199; 1164523) n=13			
**Change in Colonization with F. prausnitzii in stool L2.6 from randomization to week 8**	-57954 (364159) 10304 (-1486365; 350957) n=29	-101014 (445470) 2744 (-1486365; 350957) n=14	-17765 (278153) 21065 (-774315; 322342) n=15	8554 (162939) -14273 (-326705; 351161) n=13	-66509 (-289900; 116949)	-109569 (-372257; 121404)	-26319 (-205713; 145858)
**Colonization with F. prausnitzii in stool KLE1255 at randomization**	430738 (460372) 327602 (56316; 2104040) n=29	388569 (440720) 298151 (56316; 1795932) n=14	470096 (489986) 343225 (64786; 2104040) n=15	462245 (308874) 349653 (147476; 1214343) n=13			
**Colonization with F. prausnitzii in stool KLE1255 at week 8**	382164 (309833) 308512 (11004; 1336302) n=29	387879 (368884) 276252 (11004; 1336302) n=14	376831 (256074) 353741 (117256; 1091759) n=15	552283 (406738) 346611 (104113; 1321771) n=13			
**Change in Colonization with F. prausnitzii in stool KLE1255 from randomization to week 8**	-48574 (272444) -39854 (-1012281; 409225) n=29	-690 (212672) 17596 (-459630; 390499) n=14	-93265 (319405) -50826 (-1012281; 409225) n=15	90037 (204623) 18813 (-183783; 508825) n=13	-138611 (-315042; 23804)	-90727 (-256195; 75252)	-183303 (-395155; 20105)

Variable	High+Low dose (n=33)	High dose (n=18)	Low dose (n=15)	Placebo (n=16)	Difference and test between groups: High+Low dose vs Placebo	High dose vs Placebo	Low dose vs Placebo
**Colonization with F. prausnitzii in stool A2.165 at randomization**	487816 (563683)<br>283563 (68859; 2981157)<br>n=29	406020 (320415)<br>275428 (117096; 1027944)<br>n=14	564160 (726092)<br>303848 (68859; 2981157)<br>n=15	447652 (240108)<br>515762 (108885; 882020)<br>n=13			
**Colonization with F. prausnitzii in stool A2.165 at week 8**	495564 (515430)<br>279045 (54363; 1843881)<br>n=29	500360 (518283)<br>256521 (54363; 1806828)<br>n=14	491087 (530906)<br>279224 (65977; 1843881)<br>n=15	483607 (307096)<br>438861 (126888; 1396880)<br>n=13			
**Change in Colonization with F. prausnitzii in stool A2.165 from randomization to week 8**	7748 (316696)<br>-10391 (-1137276; 801222)<br>n=29	94340 (266990)<br>21030 (-174526; 801222)<br>n=14	-73072 (346272)<br>-35026 (-1137276; 557233)<br>n=15	35955 (200955)<br>7182 (-235870; 514860)<br>n=13	-28207 (-224120; 156399)	58386 (-123416; 247130)	-109027 (-331941; 99361)
**Colonization with F. prausnitzii in stool Total at randomization**	1773379 (1034925)<br>1596111 (503848; 4691391)<br>n=29	1754422 (1020550)<br>1492610 (532972; 4691391)<br>n=14	1791073 (1083659)<br>1675642 (503848; 4531826)<br>n=15	1764760 (783823)<br>1791817 (533480; 3106899)<br>n=13			
**Colonization with F. prausnitzii in stool Total at week 8**	1584490 (859770)<br>1261909 (166081; 3263089)<br>n=29	1618611 (951827)<br>1518390 (166081; 3263089)<br>n=14	1552644 (796803)<br>1261909 (631767; 3072278)<br>n=15	1822508 (689936)<br>1846956 (718809; 2905463)<br>n=13			
**Change in Colonization with F. prausnitzii in stool Total from randomization to week 8**	-188889 (725644)<br>-123613 (-1719439; 1666978)<br>n=29	-135811 (784580)<br>-83617 (-1719439; 1666978)<br>n=14	-238429 (689982)<br>-160485 (-1516829; 974435)<br>n=15	57748 (455050)<br>-29279 (-809756; 819246)<br>n=13	-246638 (-681486; 197480)<br>p=0.27	-193559 (-694598; 310230)<br>p=0.46	-296178 (-752188; 161648)<br>p=0.21
**Colonization with D. Piger in stool at randomization**	9713 (18100)<br>3 (0; 56828)<br>n=29	11651 (19326)<br>3 (0; 49271)<br>n=14	7904 (17354)<br>5 (0; 56828)<br>n=15	9706 (27312)<br>4 (0; 96395)<br>n=13			
**Colonization with D. Piger in stool at week 8**	21485 (36584)<br>11 (0; 154186)<br>n=29	38760 (46093)<br>32378 (0; 154186)<br>n=14	5361 (11266)<br>9 (0; 29886)<br>n=15	9481 (29229)<br>5 (0; 105371)<br>n=13			
**Change in Colonization with D. Piger in stool from randomization to week 8**	11772 (35129)<br>3 (-26942; 154183)<br>n=29	27110 (45986)<br>3401 (-13899; 154183)<br>n=14	-2543 (7047)<br>1 (-26942; 14)<br>n=15	-225.5 (4300.6)<br>1 (-11918; 8976)<br>n=13	11997 (-3365; 33395)	27335 (3893; 53604)	-2318 (-6739; 1706)

For continuous variables Mean (SD) / Median (Min; Max) / n= is presented.
For comparison between groups the Fisher´s Non Parametric Permutation Test was used for continuous variables. The confidence interval for then mean difference between groups is based on Fishers non-parametric permutation test.
2020-01-01 Analys.sas

Table 14.2.6.3 Post-hoc Subgroup Analysis - Colonization with F. prausnitzii and D. Piger in stool (Subgroup population according to the protocol for feces analyses)

					Difference and test between groups		
**Variable**	**High+Low dose (n=30)**	**High dose (n=16)**	**Low dose (n=14)**	**Placebo (n=16)**	**High+Low dose vs Placebo**	**High dose vs Placebo**	**Low dose vs Placebo**
**Colonization with F. prausnitzii in stool SL3.3 at randomization**	410594 (314227) 266737 (64423; 1322383) n=27	505919 (389396) 424046 (87115; 1322383) n=13	322078 (199969) 243211 (64423; 648169) n=14	447790 (427354) 344404 (65739; 1495280) n=13			
**Colonization with F. prausnitzii in stool SL3.3 at week 8**	314027 (235578) 229458 (47263; 1068229) n=27	369646 (283981) 306900 (73590; 1068229) n=13	262381 (174891) 192118 (47263; 589660) n=14	370992 (257997) 337055 (48369; 772443) n=13			
**Change in Colonization with F. prausnitzii in stool SL3.3 from randomization to week 8**	-96567 (306336) -47603 (-934033; 557397) n=27	-136273 (433700) -22112 (-934033; 557397) n=13	-59696 (104960) -55802 (-314365; 151473) n=14	-76798 (313430) -4527 (-1027009; 285842) n=13	-19769 (-236305; 178575)	-59475 (-364734; 246413)	17102 (-142738; 189188)
**Colonization with F. prausnitzii in stool L2.6 at randomization**	452280 (479594) 271256 (97458; 2395226) n=27	471763 (629559) 264946 (97458; 2395226) n=13	434188 (305669) 288518 (155219; 1110434) n=14	407073 (270661) 334194 (105446; 910201) n=13			
**Colonization with F. prausnitzii in stool L2.6 at week 8**	373826 (218150) 336119 (19808; 908861) n=27	350119 (263144) 217212 (19808; 908861) n=13	395839 (173713) 341979 (142853; 738990) n=14	415627 (310731) 314303 (69199; 1164523) n=13			
**Change in Colonization with F. prausnitzii in stool L2.6 from randomization to week 8**	-78454 (369161) -4816 (-1486365; 350957) n=27	-121644 (456647) -4816 (-1486365; 350957) n=13	-38349 (276543) -3689 (-774315; 322342) n=14	8554 (162939) -14273 (-326705; 351161) n=13	-87008 (-316908; 98279)	-130198 (-399457; 112137)	-46903 (-227767; 126806)
**Colonization with F. prausnitzii in stool KLE1255 at randomization**	437799 (476959) 307241 (56316; 2104040) n=27	393258 (458352) 268700 (56316; 1795932) n=13	479158 (507176) 374425 (64786; 2104040) n=14	462245 (308874) 349653 (147476; 1214343) n=13			
**Colonization with F. prausnitzii in stool KLE1255 at week 8**	379902 (321105) 293907 (11004; 1336302) n=27	382092 (383284) 258596 (11004; 1336302) n=13	377869 (265708) 335090 (117256; 1091759) n=14	552283 (406738) 346611 (104113; 1321771) n=13			
**Change in Colonization with F. prausnitzii in stool KLE1255 from randomization to week 8**	-57897 (279939) -43810 (-1012281; 409225) n=27	-11167 (217564) 9984 (-459630; 390499) n=13	-101289 (329890) -51560 (-1012281; 409225) n=14	90037 (204623) 18813 (-183783; 508825) n=13	-147934 (-327911; 21734)	-101204 (-272622; 69409)	-191326 (-410812; 24617)

					Difference and test between groups		
**Variable**	**High+Low dose (n=30)**	**High dose (n=16)**	**Low dose (n=14)**	**Placebo (n=16)**	**High+Low dose vs Placebo**	**High dose vs Placebo**	**Low dose vs Placebo**
**Colonization with F. prausnitzii in stool A2.165 at randomization**	477842 (572743)<br>283563 (68859; 2981157)<br>n=27	358179 (276603)<br>267293 (117096; 1005606)<br>n=13	588958 (746881)<br>320985 (68859; 2981157)<br>n=14	447652 (240108)<br>515762 (108885; 882020)<br>n=13			
**Colonization with F. prausnitzii in stool A2.165 at week 8**	490329 (528533)<br>268200 (54363; 1843881)<br>n=27	473202 (528976)<br>253679 (54363; 1806828)<br>n=13	506233 (547574)<br>306846 (65977; 1843881)<br>n=14	483607 (307096)<br>438861 (126888; 1396880)<br>n=13			
**Change in Colonization with F. prausnitzii in stool A2.165 from randomization to week 8**	12487 (326492)<br>-10391 (-1137276; 801222)<br>n=27	115022 (265964)<br>52450 (-101736; 801222)<br>n=13	-82725 (357243)<br>-52474 (-1137276; 557233)<br>n=14	35955 (200955)<br>7182 (-235870; 514860)<br>n=13	-23468 (-223995; 165046)	79068 (-109564; 269471)	-118680 (-349496; 100831)
**Colonization with F. prausnitzii in stool Total at randomization**	1778515 (1068642)<br>1596111 (503848; 4691391)<br>n=27	1729120 (1057641)<br>1389109 (532972; 4691391)<br>n=13	1824382 (1116569)<br>1830018 (503848; 4531826)<br>n=14	1764760 (783823)<br>1791817 (533480; 3106899)<br>n=13			
**Colonization with F. prausnitzii in stool Total at week 8**	1558085 (883739)<br>1259476 (166081; 3263089)<br>n=27	1575059 (976066)<br>1220361 (166081; 3263089)<br>n=13	1542323 (825841)<br>1260693 (631767; 3072278)<br>n=14	1822508 (689936)<br>1846956 (718809; 2905463)<br>n=13			
**Change in Colonization with F. prausnitzii in stool Total from randomization to week 8**	-220430 (742073)<br>-131127 (-1719439; 1666978)<br>n=27	-154061 (813518)<br>-107462 (-1719439; 1666978)<br>n=13	-282059 (694224)<br>-193651 (-1516829; 974435)<br>n=14	57748 (455050)<br>-29279 (-809756; 819246)<br>n=13	-278179 (-732021; 172297)<br>p=0.23	-211809 (-743432; 320599)<br>p=0.43	-339808 (-807376; 128587)<br>p=0.15
**Colonization with D. Piger in stool at randomization**	8937 (17676)<br>3 (0; 56828)<br>n=27	9442 (18183)<br>3 (0; 49271)<br>n=13	8469 (17866)<br>5 (0; 56828)<br>n=14	9706 (27312)<br>4 (0; 96395)<br>n=13			
**Colonization with D. Piger in stool at week 8**	21471 (37486)<br>11 (0; 154186)<br>n=27	38408 (47956)<br>21416 (0; 154186)<br>n=13	5744 (11590)<br>9 (0; 29886)<br>n=14	9481 (29229)<br>5 (0; 105371)<br>n=13			
**Change in Colonization with D. Piger in stool from randomization to week 8**	12534 (36333)<br>3 (-26942; 154183)<br>n=27	28966 (47315)<br>3826 (-13899; 154183)<br>n=13	-2725 (7277)<br>1 (-26942; 14)<br>n=14	-225.5 (4300.6)<br>1 (-11918; 8976)<br>n=13	12759 (-3514; 34429)	29191 (4697; 56368)	-2500 (-7137; 1705)

For continuous variables Mean (SD) / Median (Min; Max) / n= is presented.
For comparison between groups the Fisher´s Non Parametric Permutation Test was used for continuous variables. The confidence interval for then mean difference between groups is based on Fishers non-parametric permutation test.
2020-01-01 Analys.sas

Table 14.3.1 Duration of Exposure and Compliance (Safety Population)

	**High dose (n=18)**	**Low dose (n=16)**	**Placebo (n=16)**
**Follow-up days from randomizaiton to end of treatment or early discontinuation**	51.5 (16.4)<br>56 (6; 66)<br>n=18	56.9 (2.2)<br>56 (53; 61)<br>n=16	50.2 (17.1)<br>55.5 (3; 61)<br>n=16
**Follow-up days from randomizaiton to end of study or early discontinuation**	63.9 (20.9)<br>70 (6; 78)<br>n=18	70.7 (4.4)<br>71 (58; 77)<br>n=16	62.7 (21.9)<br>70 (3; 75)<br>n=16
**Compliance (cont.)**	97.8 (4.3)<br>100 (86.2; 100)<br>n=16	96.6 (4.8)<br>100 (85; 100)<br>n=16	97.0 (3.1)<br>96.4 (91.7; 100)<br>n=14
**Compliance (cat.)**			
**>75%**	16 (100.0%)	16 (100.0%)	14 (100.0%)
**Missing**	2	0	2

For categorical variables n (%) is presented.
For continuous variables Mean (SD) / Median (Min; Max) / n= is presented.

2020-01-01 Analys.sas

Table 14.3.2.1 Summary of Adverse Events (Safety Population)

	High dose (n=18)	Low dose (n=16)	Placebo (n=16)
**Any adverse event**	13 (72.2%)	7 (43.8%)	10 (62.5%)
**Any adverse event related to IP**	6 (33.3%)	6 (37.5%)	6 (37.5%)
For categorical variables n (%) is presented.<br><br>2020-01-01 Analys.sas			

Table 14.3.2.2 Adverse Events, by System Organ Class and Preferred Term (Safety Population)

SOC<br>PT	High dose<br>(n=18)		Low dose<br>(n=16)		Placebo<br>(n=16)	
	Events	Subjects with Events n (%)	Events	Subjects with Events n (%)	Events	Subjects with Events n (%)
**Any AE**	**26**	**13 (72.2%)**	**14**	**7 (43.8%)**	**12**	**10 (62.5%)**
**Gastrointestinal disorders**	**14**	**6 (33.3%)**	**9**	**6 (37.5%)**	**6**	**6 (37.5%)**
Dyspepsia NOS	1	1 (5.6%)				
change in bowel habit NOS	13	6 (33.3%)	9	6 (37.5%)	6	6 (37.5%)
**General disorders and administration site conditions**	**3**	**2 (11.1%)**	**3**	**3 (18.8%)**	**2**	**2 (12.5%)**
Abnormal serum enzyme levels					1	1 (6.3%)
Headache	1	1 (5.6%)	2	2 (12.5%)		
pain					1	1 (6.3%)
tiredness	2	1 (5.6%)	1	1 (6.3%)		
**Infections and infestations**	**2**	**2 (11.1%)**			**1**	**1 (6.3%)**
Bacterial foodborne intoxication, unspecified					1	1 (6.3%)
Viral intestinal infection, unspecified	2	2 (11.1%)				
**Injury, poisoning and procedural complications**	**1**	**1 (5.6%)**				
Sprain and strain of ankle	1	1 (5.6%)				
**Psychiatric disorders**	**1**	**1 (5.6%)**				
insomnia	1	1 (5.6%)				
**Reproductive system and breast disorders**	**1**	**1 (5.6%)**	**1**	**1 (6.3%)**		
Dysmenorrhoea, unspecified			1	1 (6.3%)		
Irregular menstruation, unspecified	1	1 (5.6%)				
**Respiratory, thoracic and mediastinal disorders**	**4**	**4 (22.2%)**	**1**	**1 (6.3%)**	**3**	**3 (18.8%)**
Acute nasopharyngitis [common cold]	3	3 (16.7%)	1	1 (6.3%)	3	3 (18.8%)
Acute sinusitis, unspecified	1	1 (5.6%)				
2020-01-01 Analys.sas						

Table 14.3.2.3 Adverse Events, by System Organ Class, Preferred Term and Maximum Reported Intensity (Safety Population)

SOC<br>PT	High dose/Mild (n=18)	High dose/Moderate (n=18)	High dose/Severe (n=18)	Low dose/Mild (n=16)	Low dose/Moderate (n=16)	Low dose/Severe (n=16)	Placebo/Mild (n=16)	Placebo/Moderate (n=16)	Placebo/Severe (n=16)
**Any AE**	**13 (72.2%)**	**0 (0.0%)**	**0 (0.0%)**	**7 (43.8%)**	**0 (0.0%)**	**0 (0.0%)**	**10 (62.5%)**	**0 (0.0%)**	**0 (0.0%)**
**Gastrointestinal disorders**	**6 (33.3%)**			**6 (37.5%)**			**6 (37.5%)**		
Dyspepsia NOS	1 (5.6%)								
change in bowel habit NOS	6 (33.3%)			6 (37.5%)			6 (37.5%)		
**General disorders and administration site conditions**	**2 (11.1%)**			**3 (18.8%)**			**2 (12.5%)**		
Abnormal serum enzyme levels							1 (6.3%)		
Headache	1 (5.6%)			2 (12.5%)					
pain							1 (6.3%)		
tiredness	1 (5.6%)			1 (6.3%)					
**Infections and infestations**	**2 (11.1%)**						**1 (6.3%)**		
Bacterial foodborne intoxication, unspecified							1 (6.3%)		
Viral intestinal infection, unspecified	2 (11.1%)								
**Injury, poisoning and procedural complications**	**1 (5.6%)**								
Sprain and strain of ankle	1 (5.6%)								
**Psychiatric disorders**	**1 (5.6%)**								
insomnia	1 (5.6%)								
**Reproductive system and breast disorders**	**1 (5.6%)**			**1 (6.3%)**					
Dysmenorrhoea, unspecified				1 (6.3%)					
Irregular menstruation, unspecified	1 (5.6%)								
**Respiratory, thoracic and mediastinal disorders**	**4 (22.2%)**			**1 (6.3%)**			**3 (18.8%)**		
Acute nasopharyngitis [common cold]	3 (16.7%)			1 (6.3%)			3 (18.8%)		
Acute sinusitis, unspecified	1 (5.6%)								
2020-01-01 Analys.sas									

Table 14.3.2.4 Adverse Events, by System Organ Class, Preferred Term and Causality Assessment (Safety Population)

SOC<br>PT	High dose/Unlikely (n=18)	High dose/Possibly (n=18)	Low dose/Unlikely (n=16)	Low dose/Possibly (n=16)	Placebo/Unlikely (n=16)	Placebo/Possibly (n=16)
**Any AE**	**9 (50.0%)**	**6 (33.3%)**	**4 (25.0%)**	**6 (37.5%)**	**6 (37.5%)**	**6 (37.5%)**
**Gastrointestinal disorders**		**6 (33.3%)**	**1 (6.3%)**	**6 (37.5%)**		**6 (37.5%)**
Dyspepsia NOS		1 (5.6%)				
change in bowel habit NOS		6 (33.3%)	1 (6.3%)	6 (37.5%)		6 (37.5%)
**General disorders and administration site conditions**	**2 (11.1%)**		**3 (18.8%)**		**2 (12.5%)**	
Abnormal serum enzyme levels					1 (6.3%)	
Headache	1 (5.6%)		2 (12.5%)			
pain					1 (6.3%)	
tiredness	1 (5.6%)		1 (6.3%)			
**Infections and infestations**	**2 (11.1%)**				**1 (6.3%)**	
Bacterial foodborne intoxication, unspecified					1 (6.3%)	
Viral intestinal infection, unspecified	2 (11.1%)					
**Injury, poisoning and procedural complications**	**1 (5.6%)**					
Sprain and strain of ankle	1 (5.6%)					
**Psychiatric disorders**	**1 (5.6%)**					
insomnia	1 (5.6%)					
**Reproductive system and breast disorders**	**1 (5.6%)**		**1 (6.3%)**			
Dysmenorrhoea, unspecified			1 (6.3%)			
Irregular menstruation, unspecified	1 (5.6%)					
**Respiratory, thoracic and mediastinal disorders**	**4 (22.2%)**		**1 (6.3%)**		**3 (18.8%)**	
Acute nasopharyngitis [common cold]	3 (16.7%)		1 (6.3%)		3 (18.8%)	
Acute sinusitis, unspecified	1 (5.6%)					
2020-01-01 Analys.sas						

Figure 14.2.2.1.1 Boxplots for GSRS (score 0-45) over time

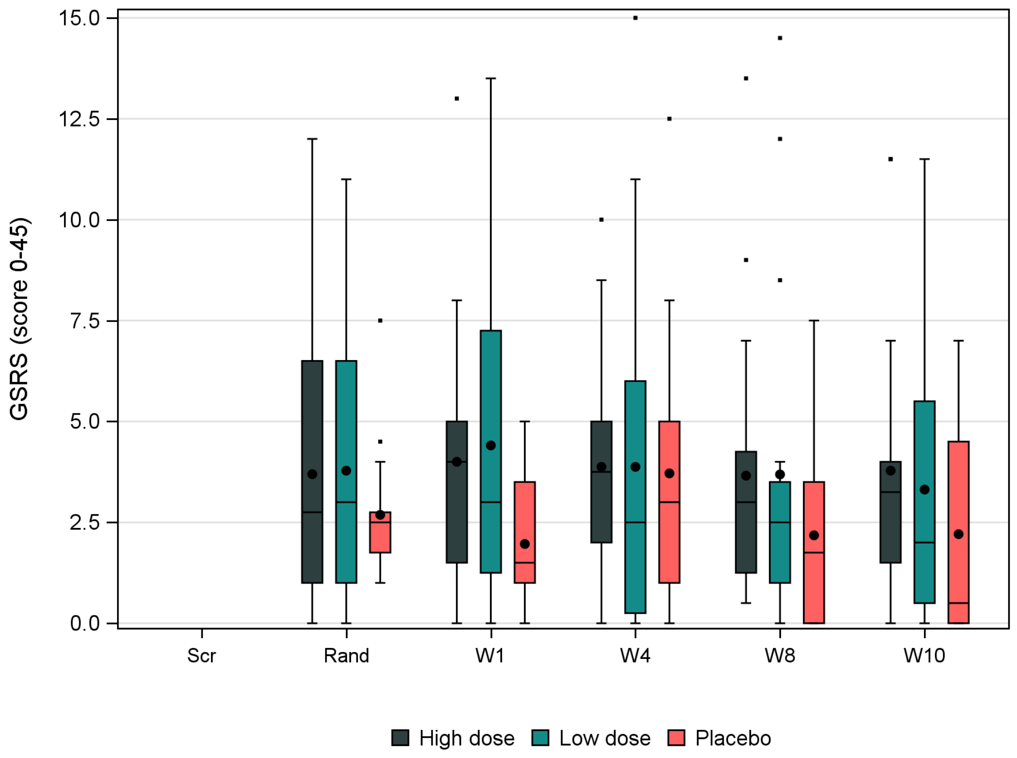

Figure 14.2.2.1.2 Individual values for GSRS (score 0-45) over time

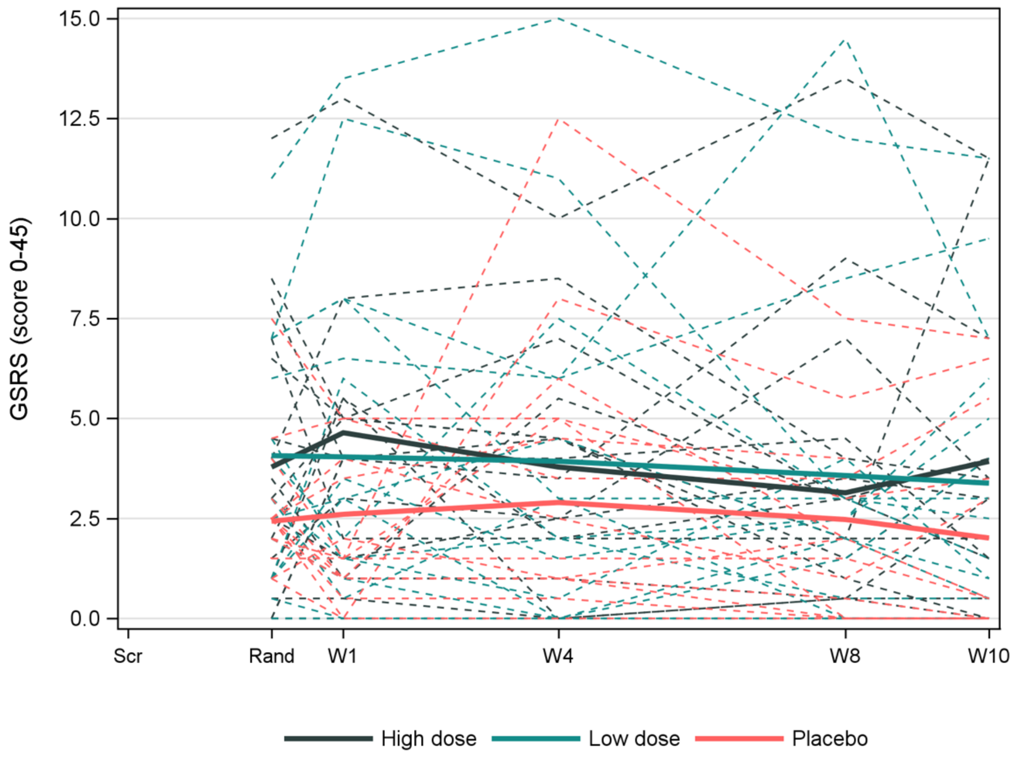

Figure 14.2.3.1.1 Boxplots for Erythrocyte sedimentation rate (mm/hour) over time

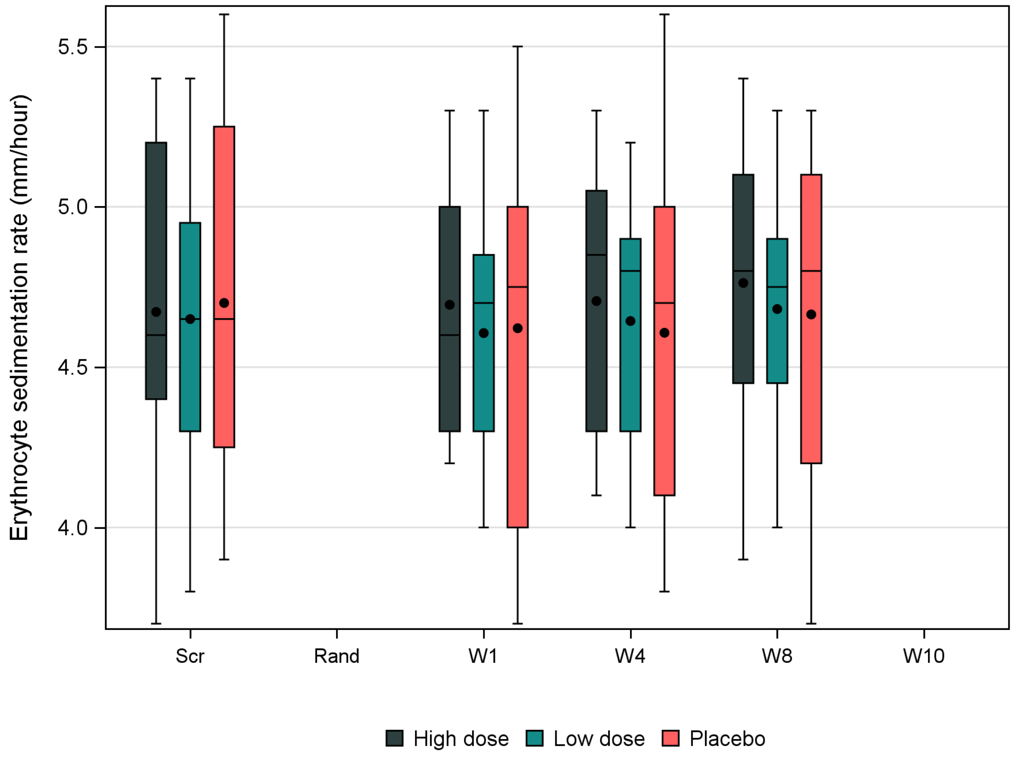

Figure 14.2.3.1.2 Individual values for Erythrocyte sedimentation rate (mm/hour) over time

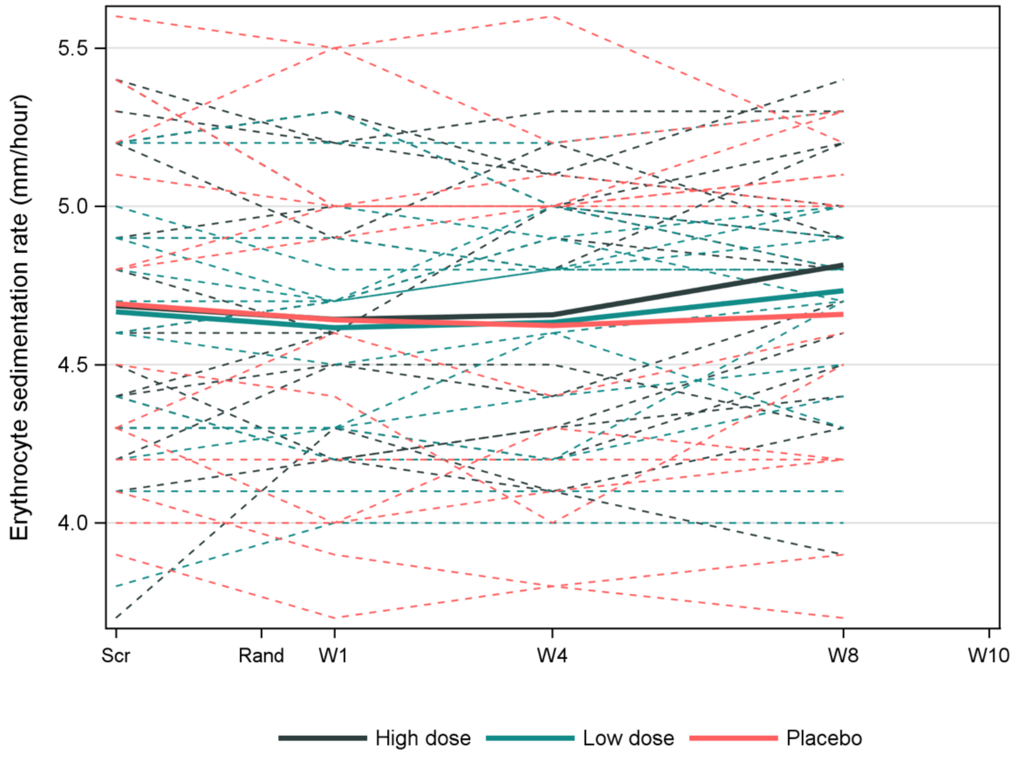

Figure 14.2.3.2.1 Boxplots for C-reactive protein (mg/L) over time

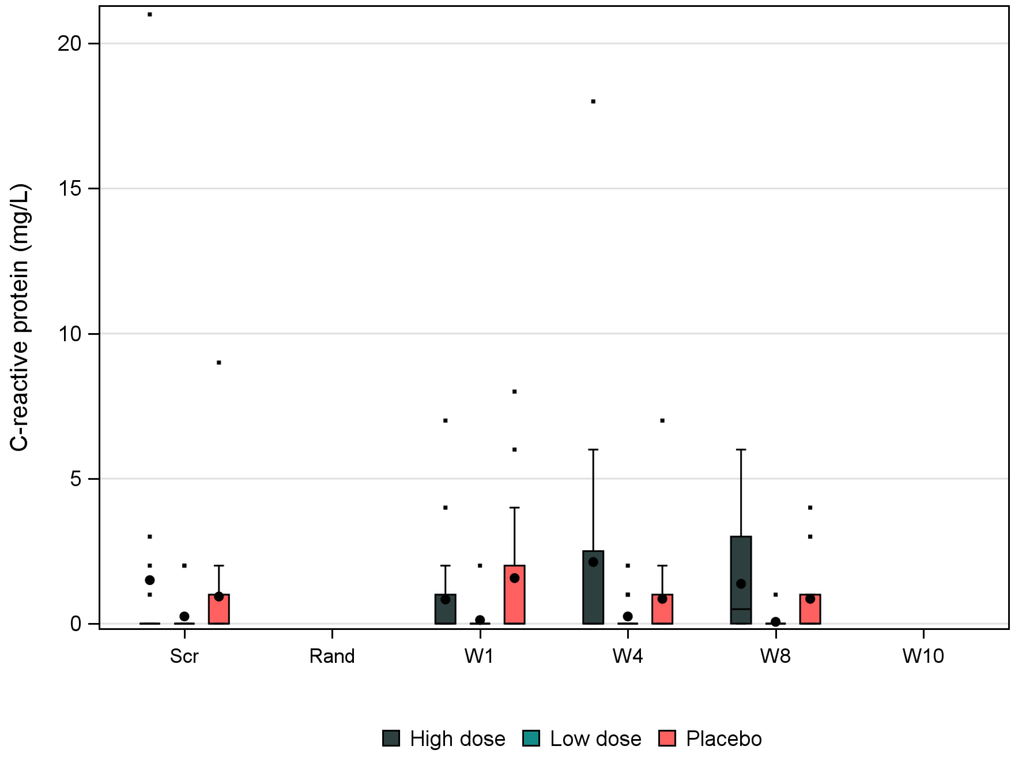

Figure 14.2.3.2.2 Individual values for C-reactive protein (mg/L) over time

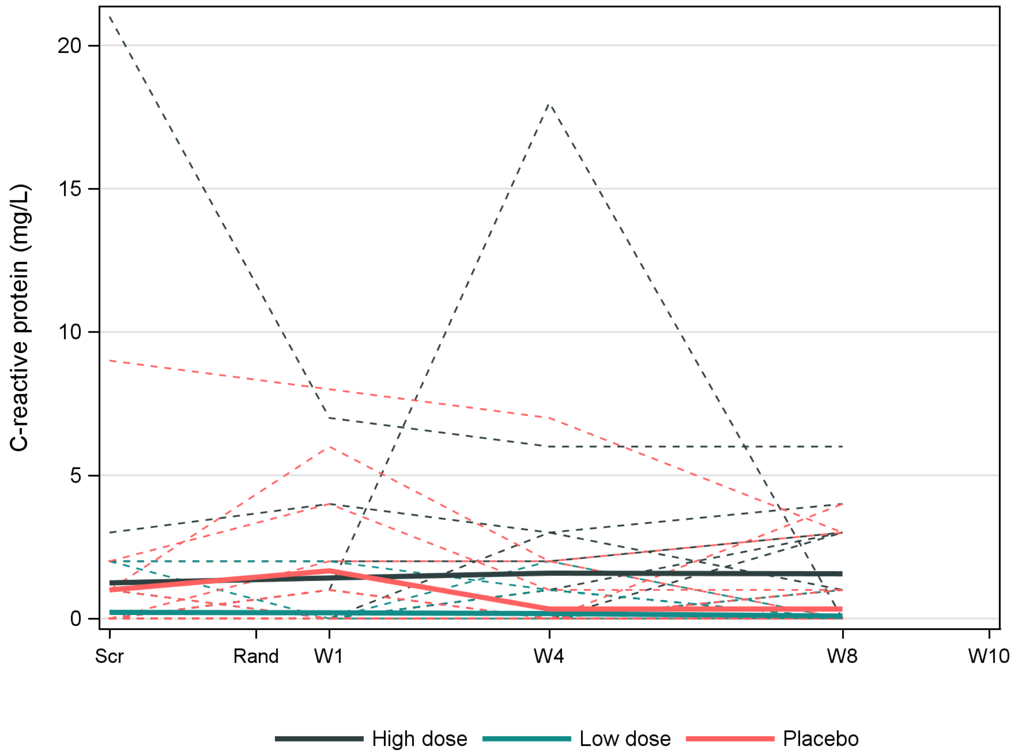

Figure 14.2.3.3.1 Boxplots for Red blood cell count (10^9/L) over time

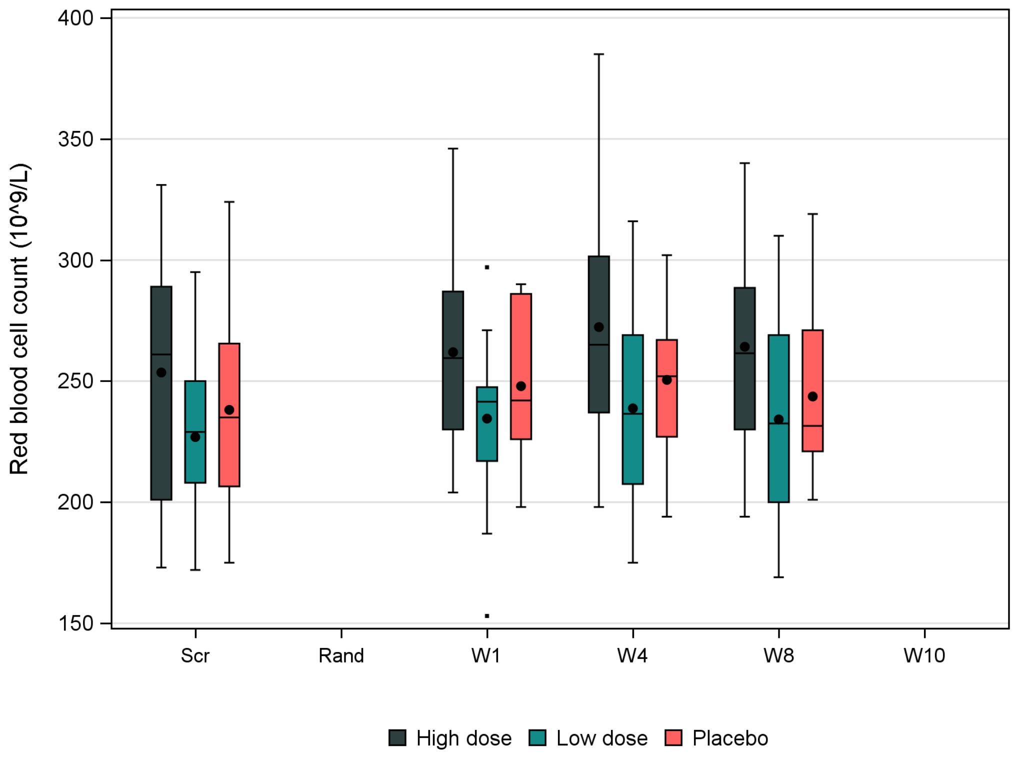

Figure 14.2.3.3.2 Individual values for Red blood cell count (10^9/L) over time

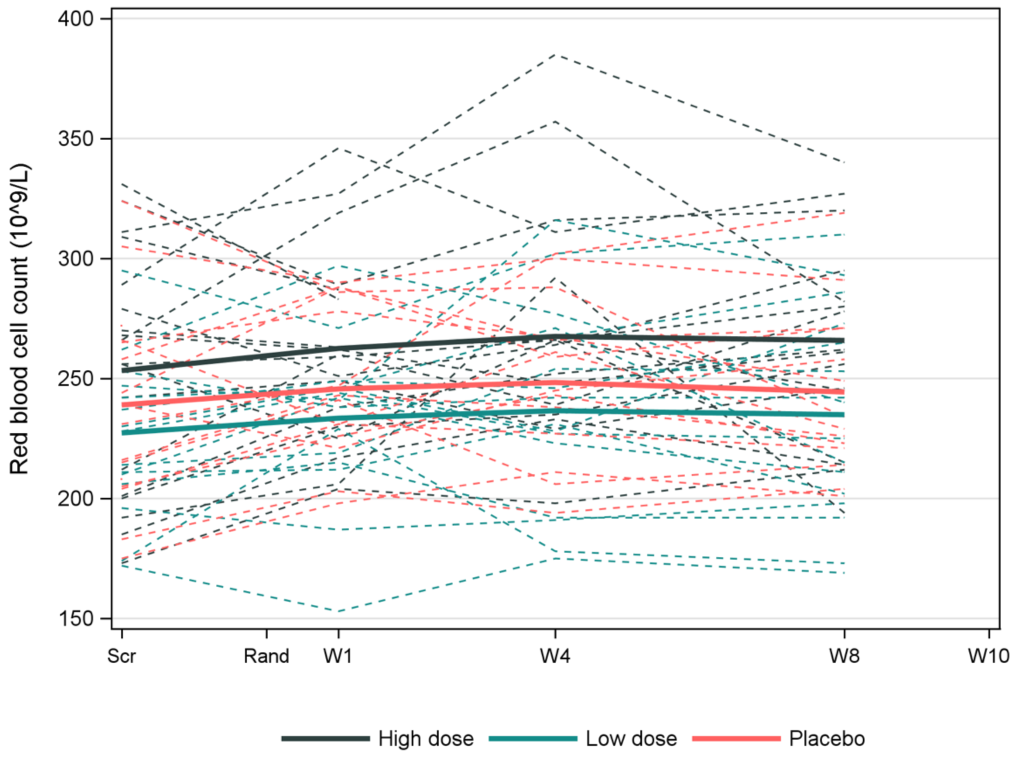

Figure 14.2.3.4.1 Boxplots for White blood cell count (10^9/L) over time

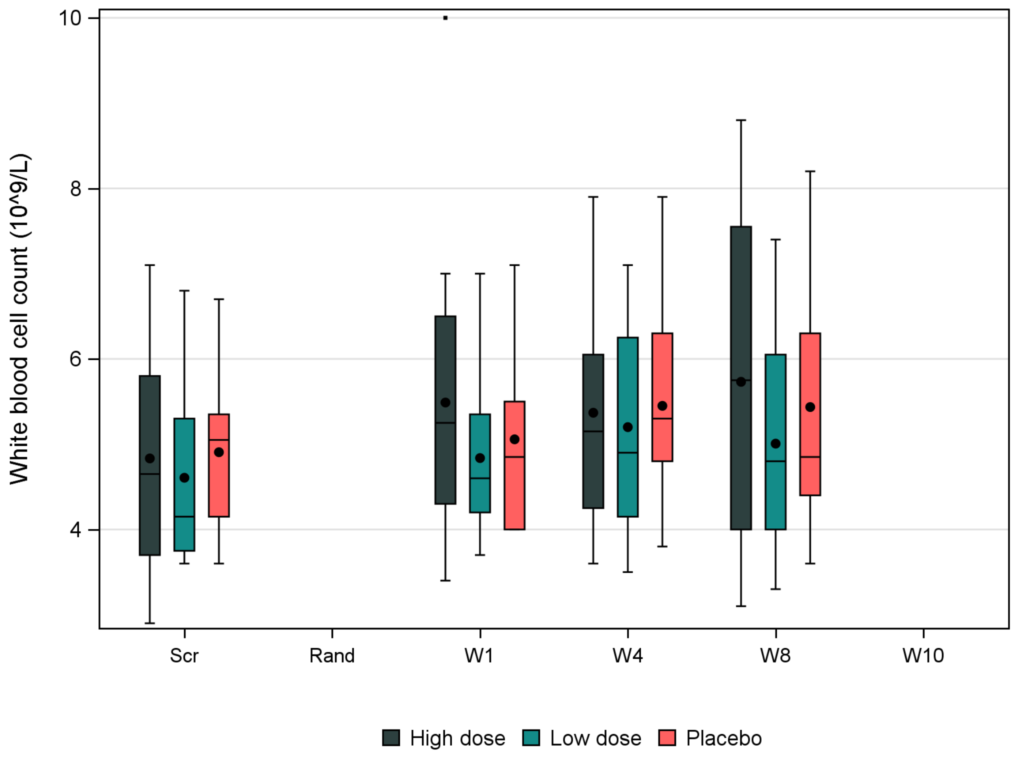

Figure 14.2.3.4.2 Individual values for White blood cell count (10^9/L) over time

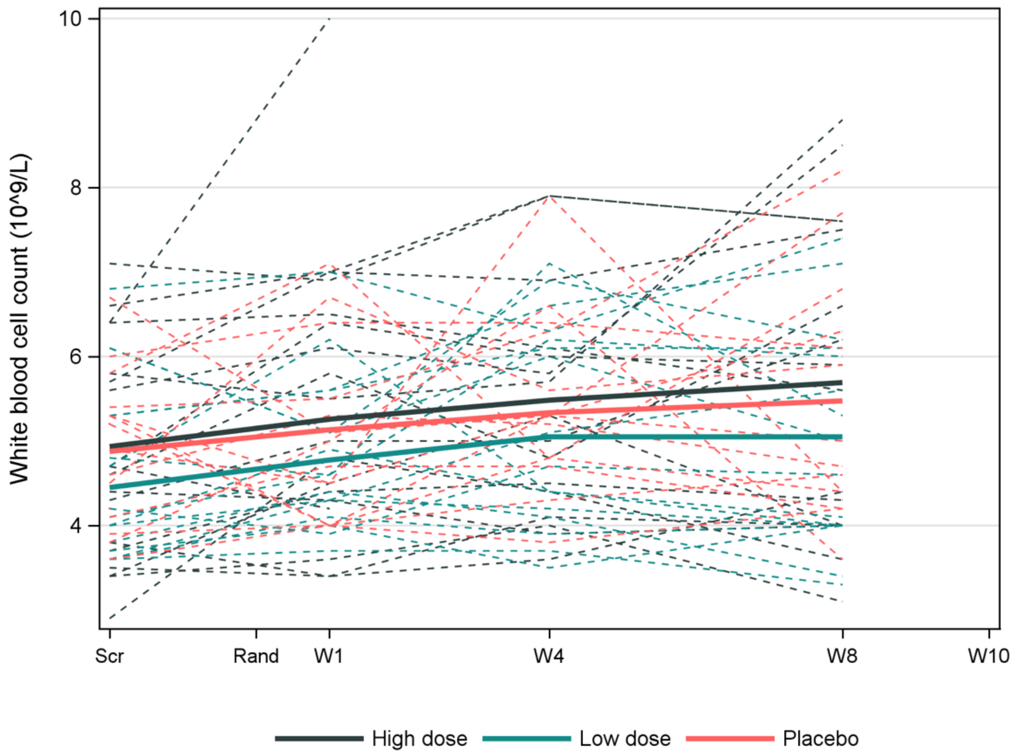

Figure 14.2.3.5.1 Boxplots for Platelet count (10^9/L) over time

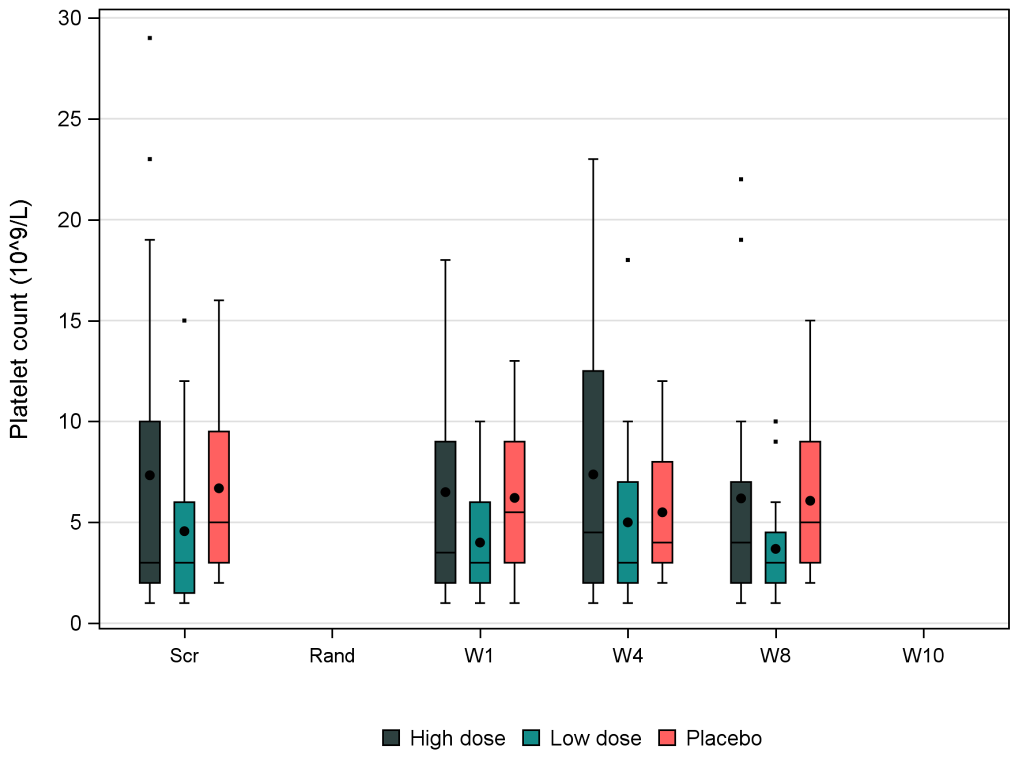

Figure 14.2.3.5.2 Individual values for Platelet count (10^9/L) over time

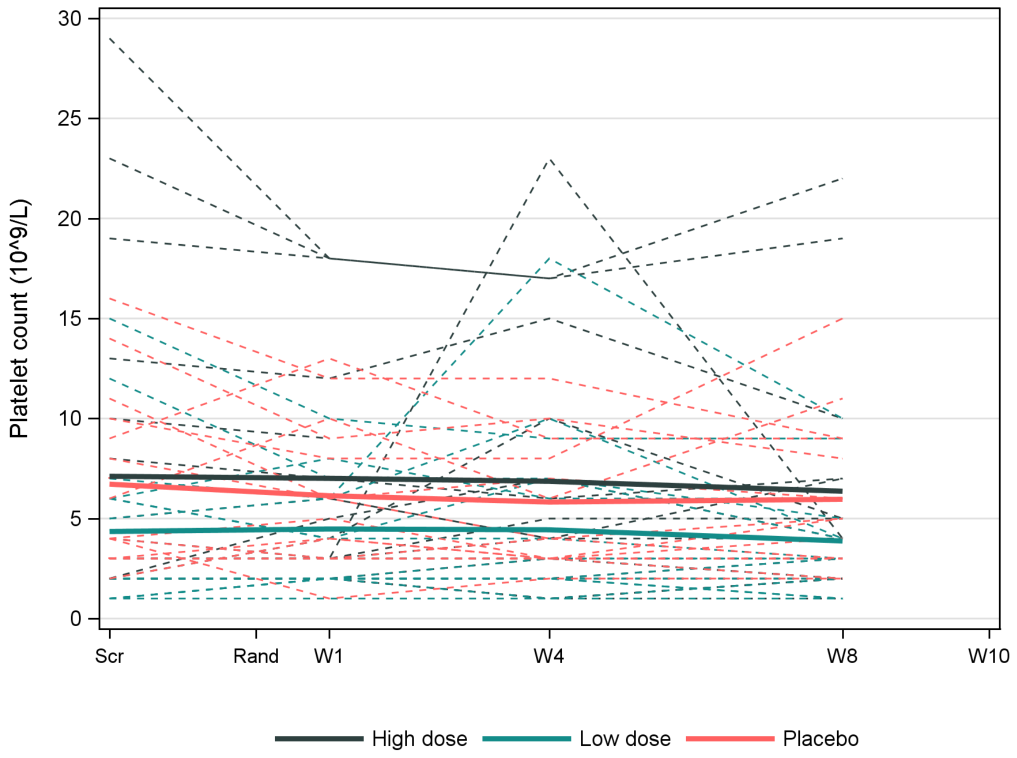

Figure 14.2.3.6.1 Boxplots for Alanine transaminase (µkat/L) over time

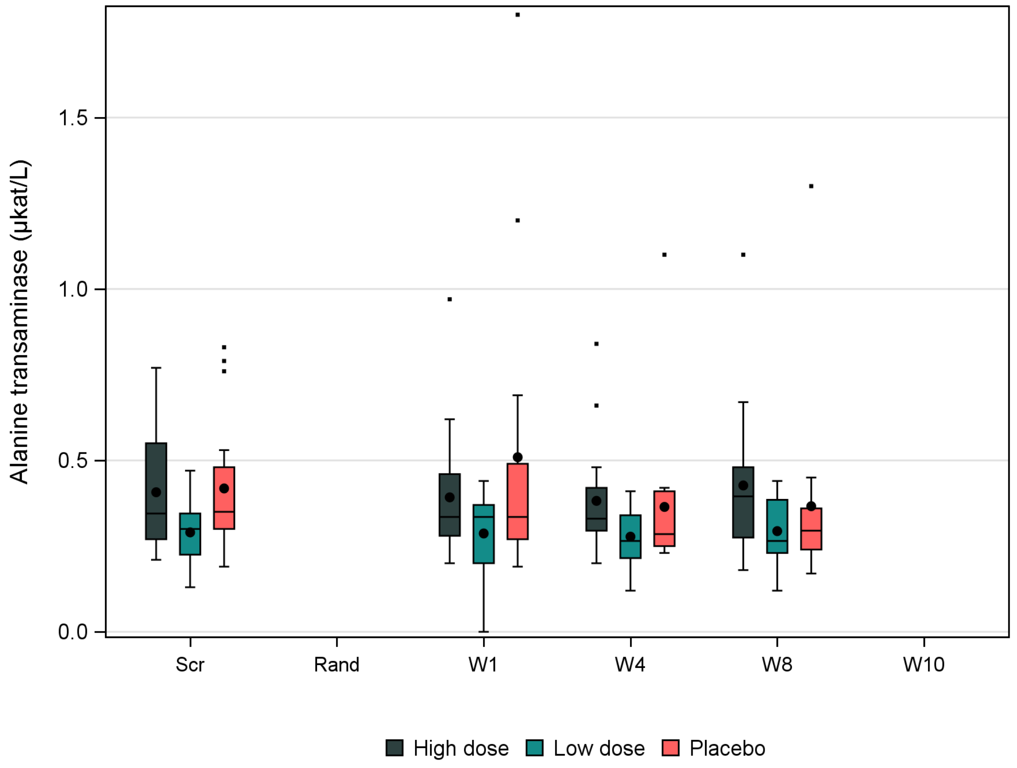

Figure 14.2.3.6.2 Individual values for Alanine transaminase (µkat/L) over time

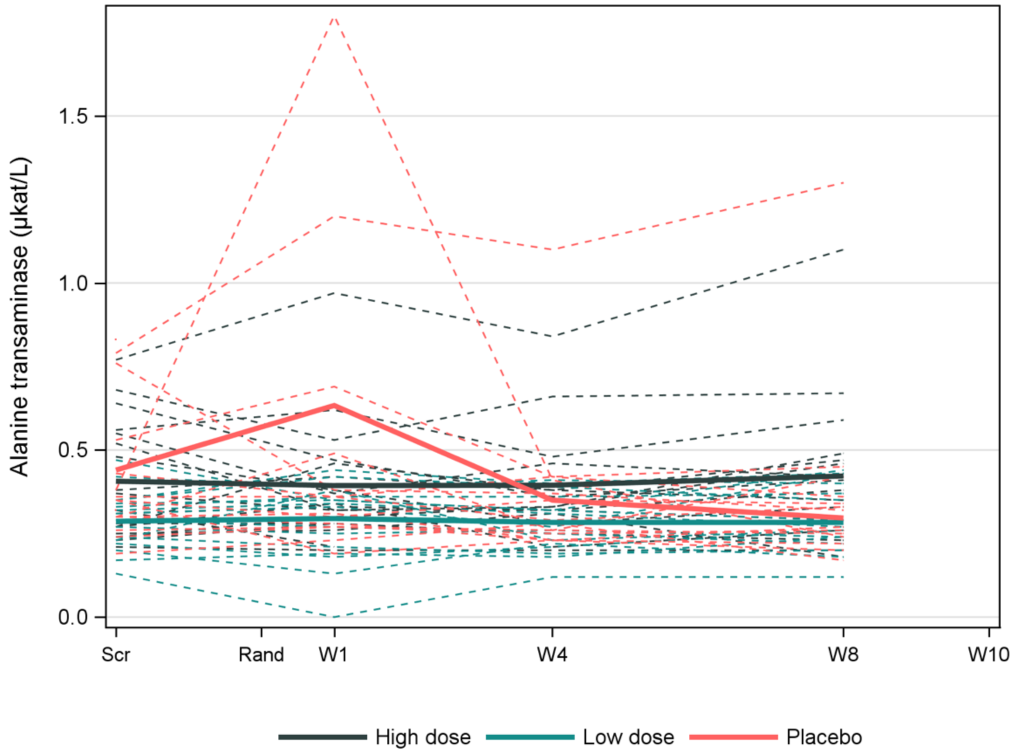

Figure 14.2.3.7.1 Boxplots for Aspartate transaminase (µkat/L) over time

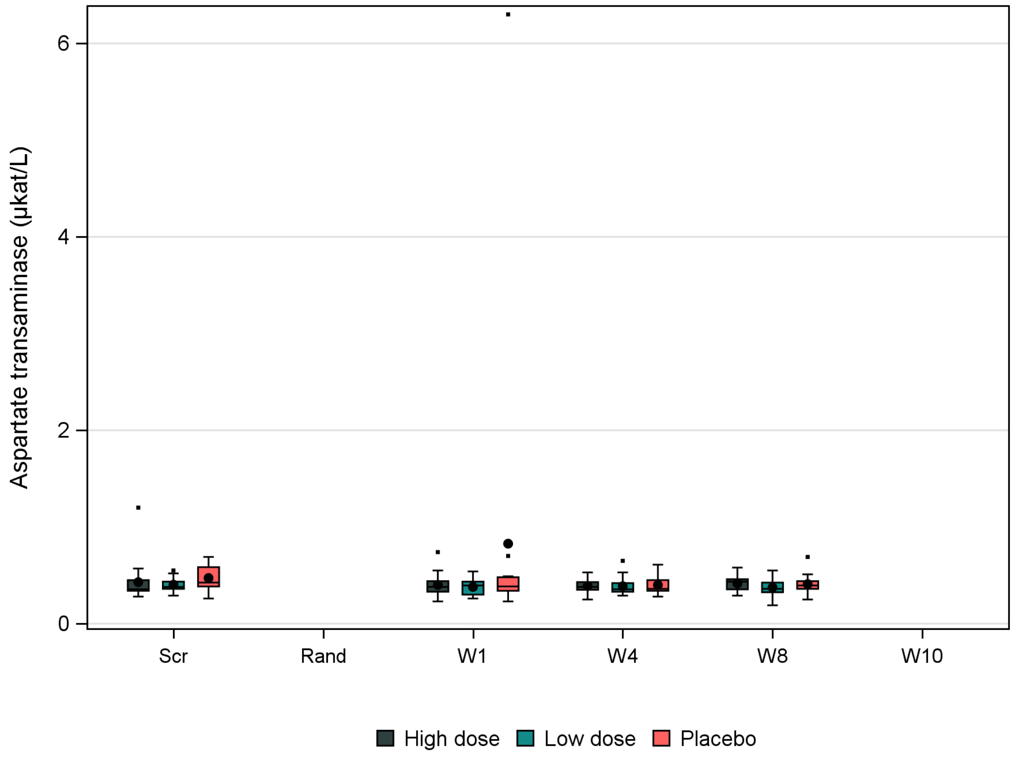

Figure 14.2.3.7.2 Individual values for Aspartate transaminase (µkat/L) over time

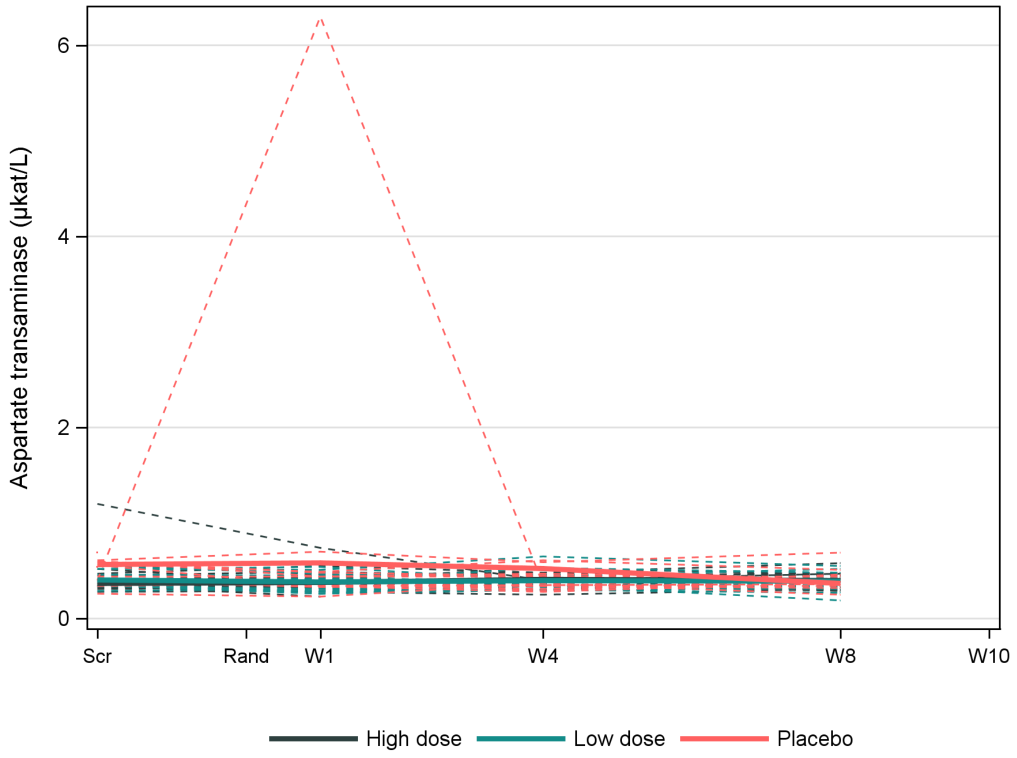

Figure 14.2.3.8.1 Boxplots for Alkaline phosphatase (µkat/L) over time

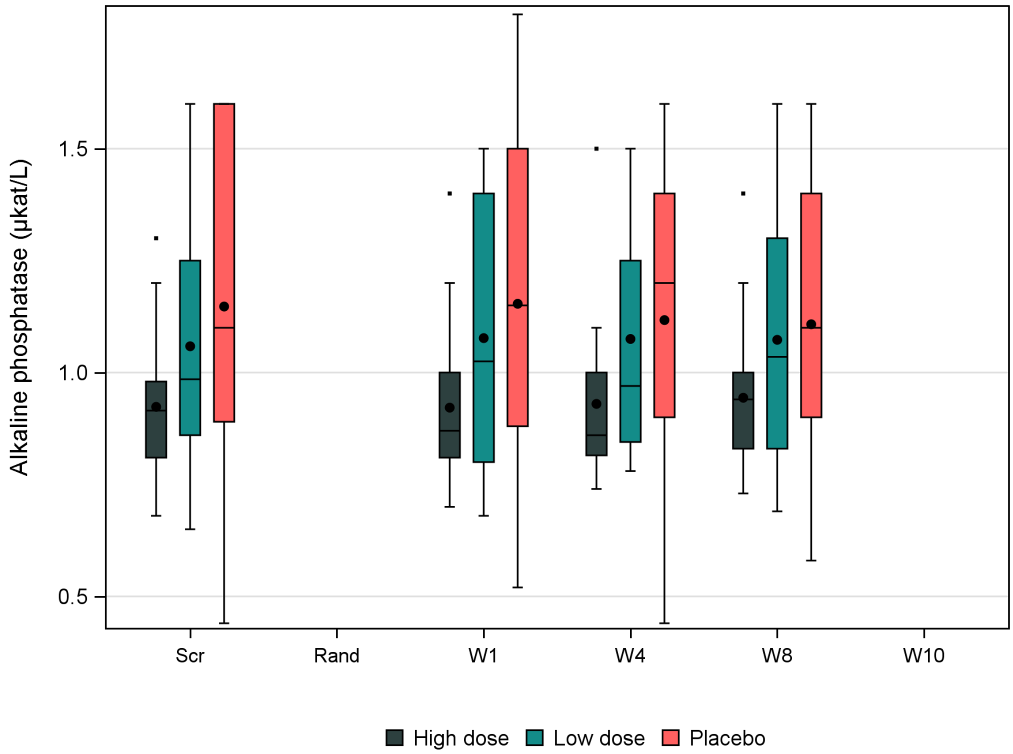

Figure 14.2.3.8.2 Individual values for Alkaline phosphatase (µkat/L) over time

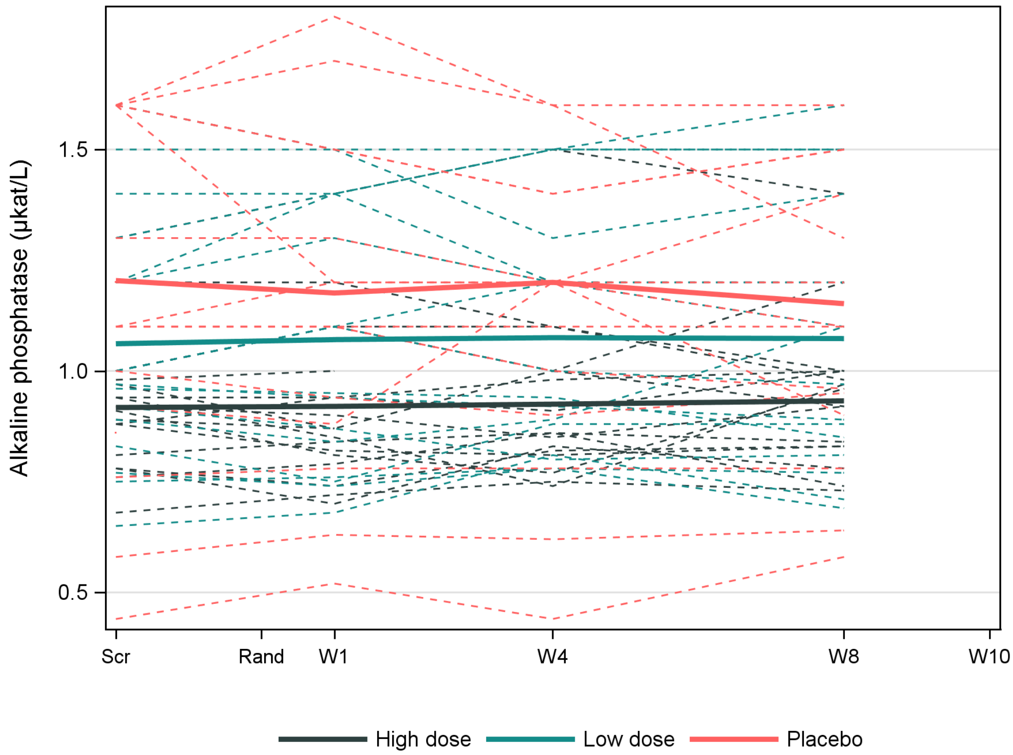

Figure 14.2.3.9.1 Boxplots for Bilirubin (µmol/L) over time

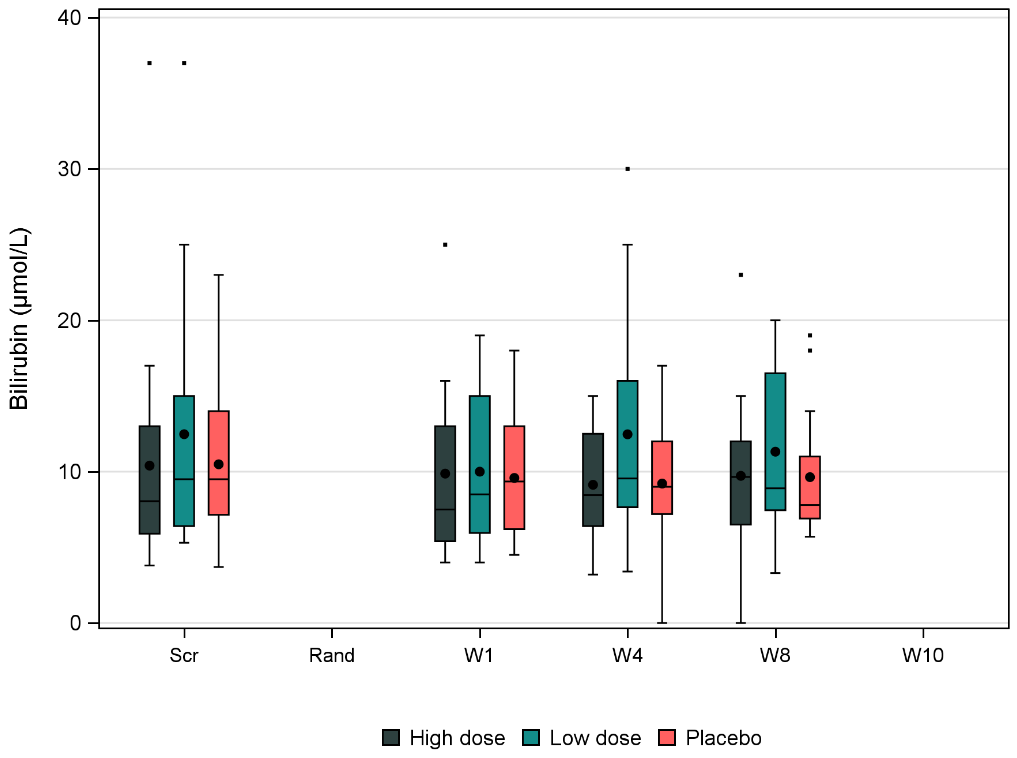

Figure 14.2.3.9.2 Individual values for Bilirubin (µmol/L) over time

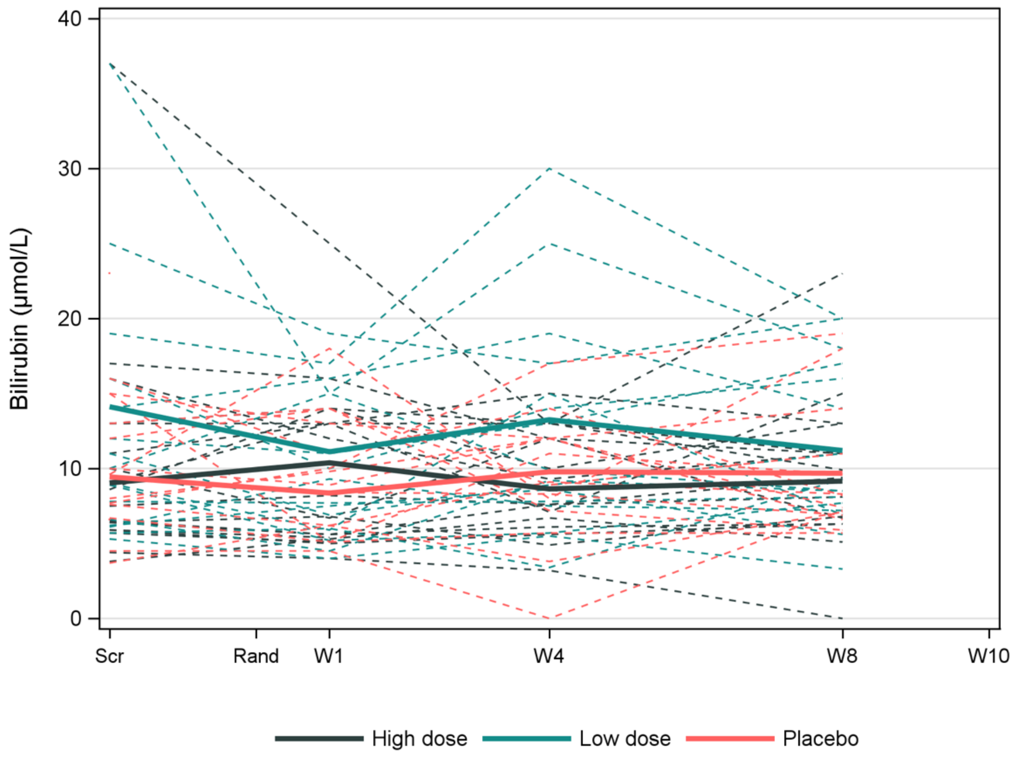

Figure 14.2.3.10.1 Boxplots for Renal function eGFR (ml/min) over time

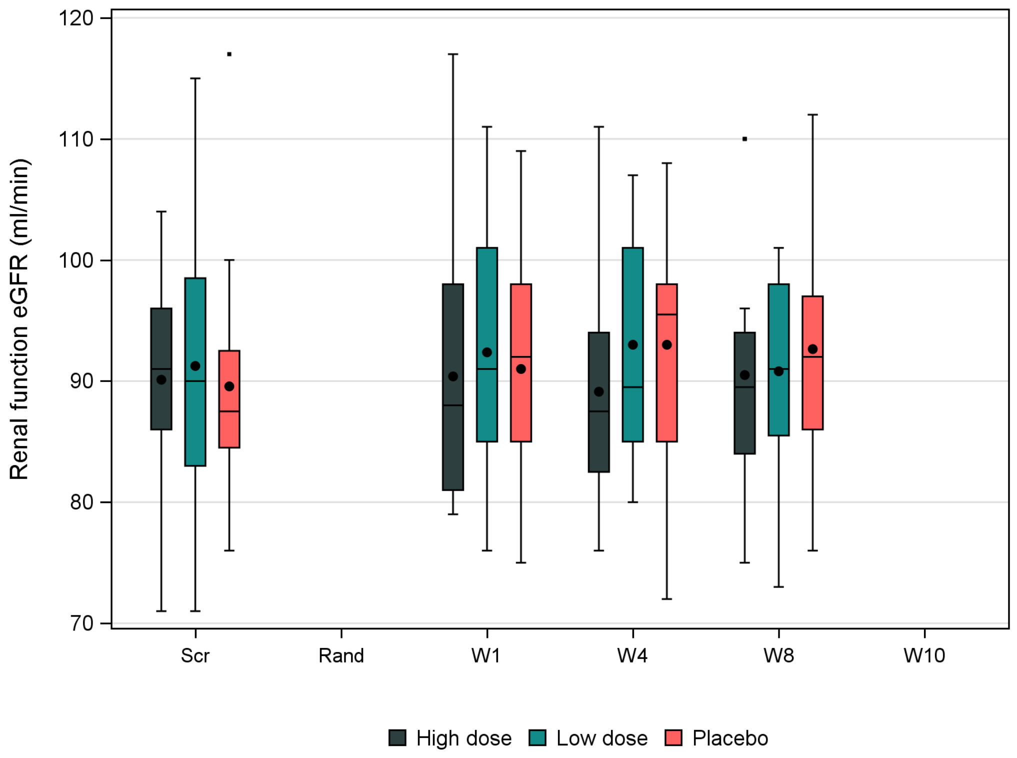

Figure 14.2.3.10.2 Individual values for Renal function eGFR (ml/min) over time

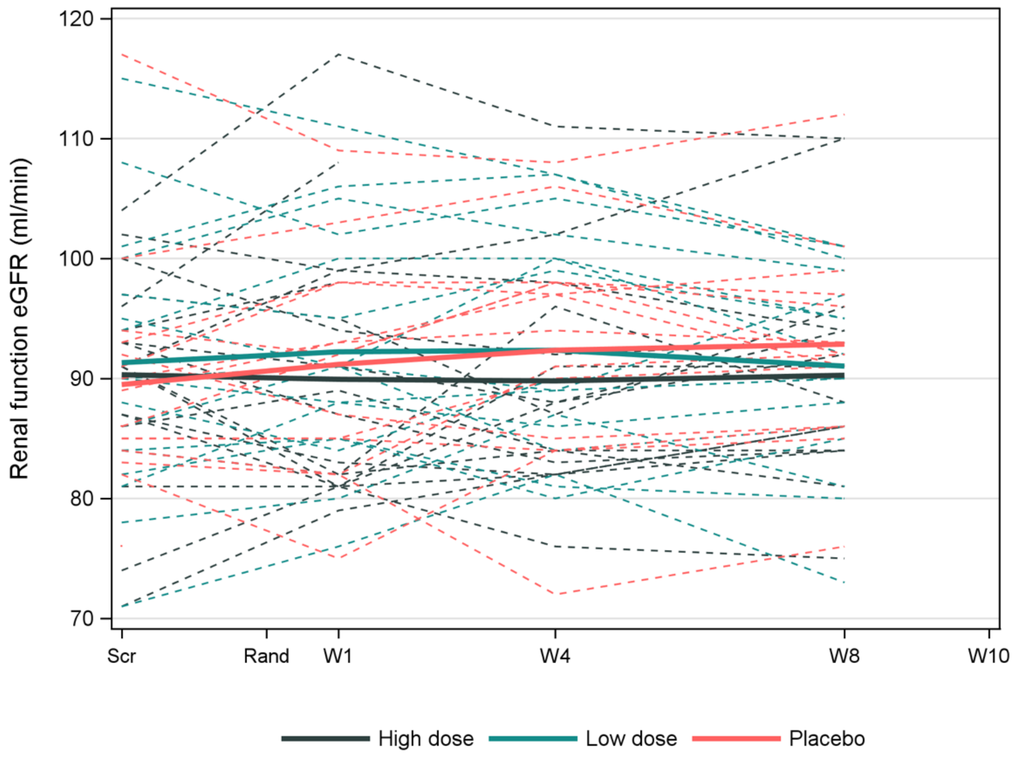

Figure 14.2.3.11.1 Boxplots for Blood total protein (g/l) over time

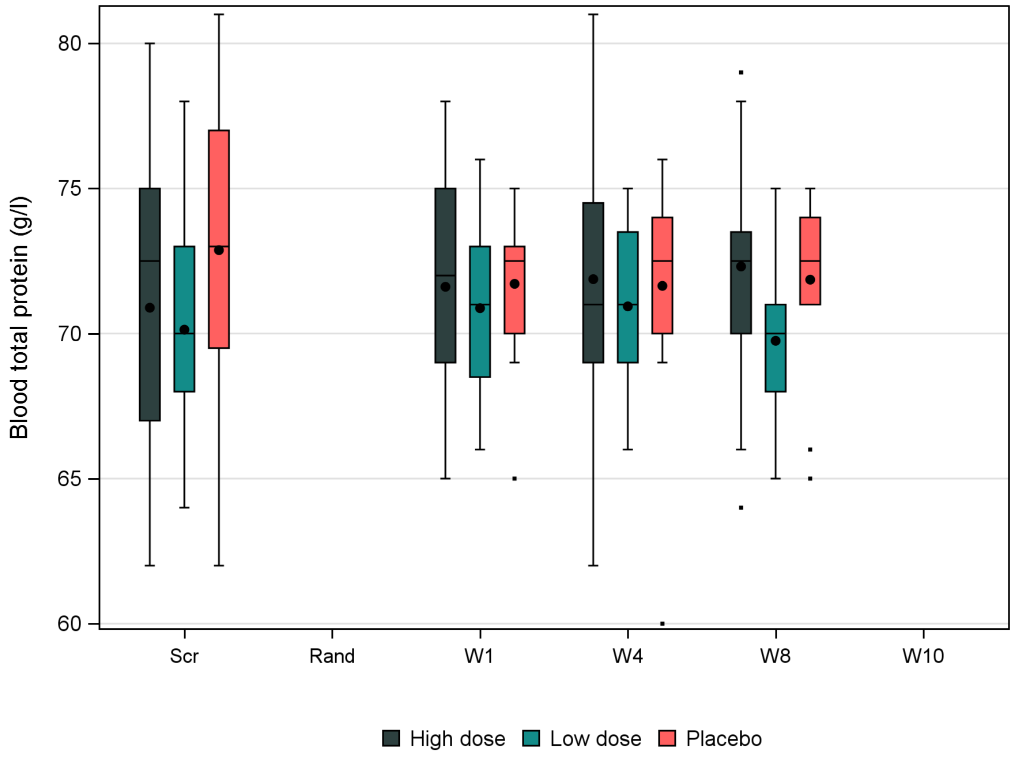

Figure 14.2.3.11.2 Individual values for Blood total protein (g/l) over time

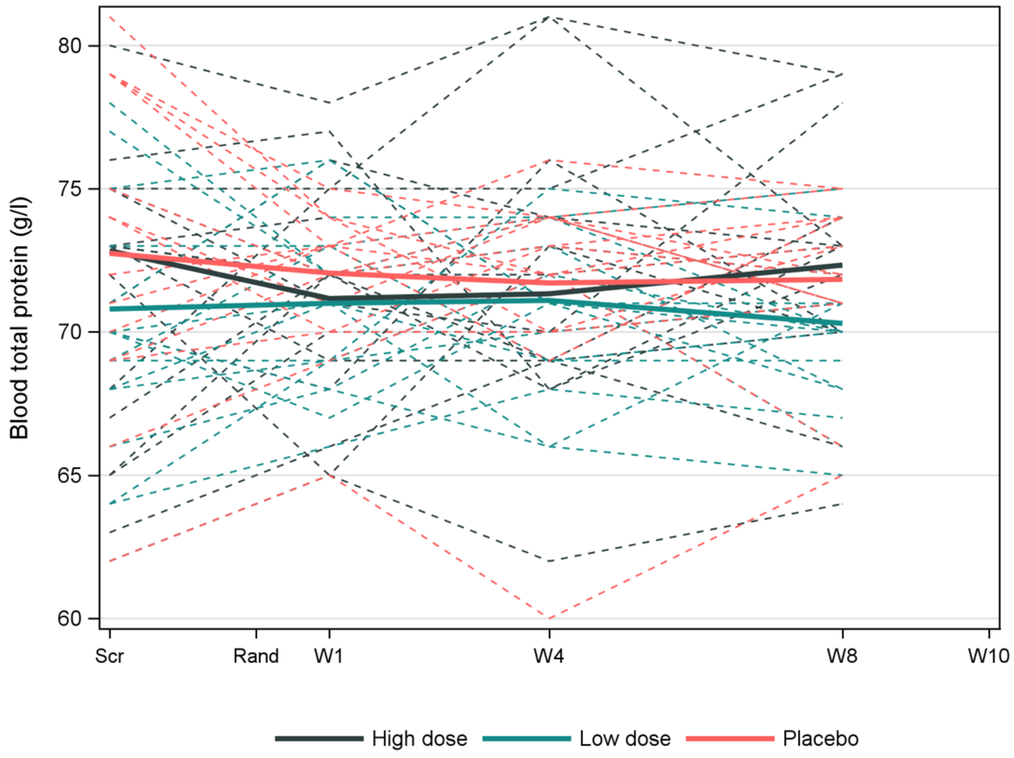

Figure 14.2.3.12.1 Boxplots for Blood haemoglobin (g/l) over time

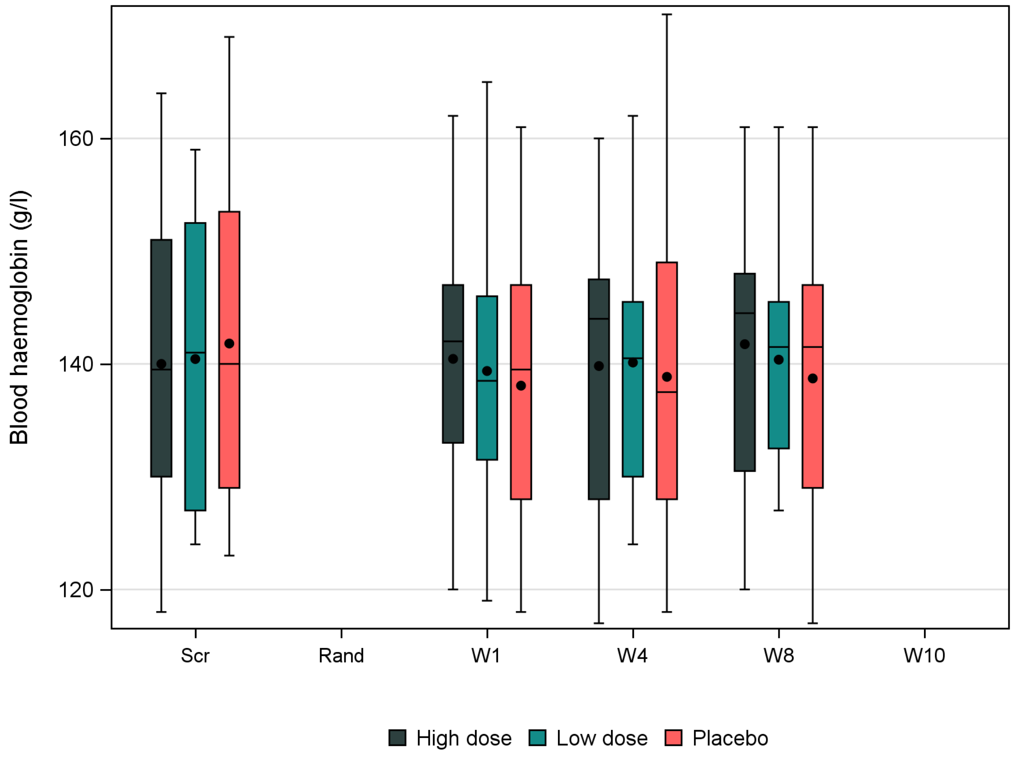

Figure 14.2.3.12.2 Individual values for Blood haemoglobin (g/l) over time

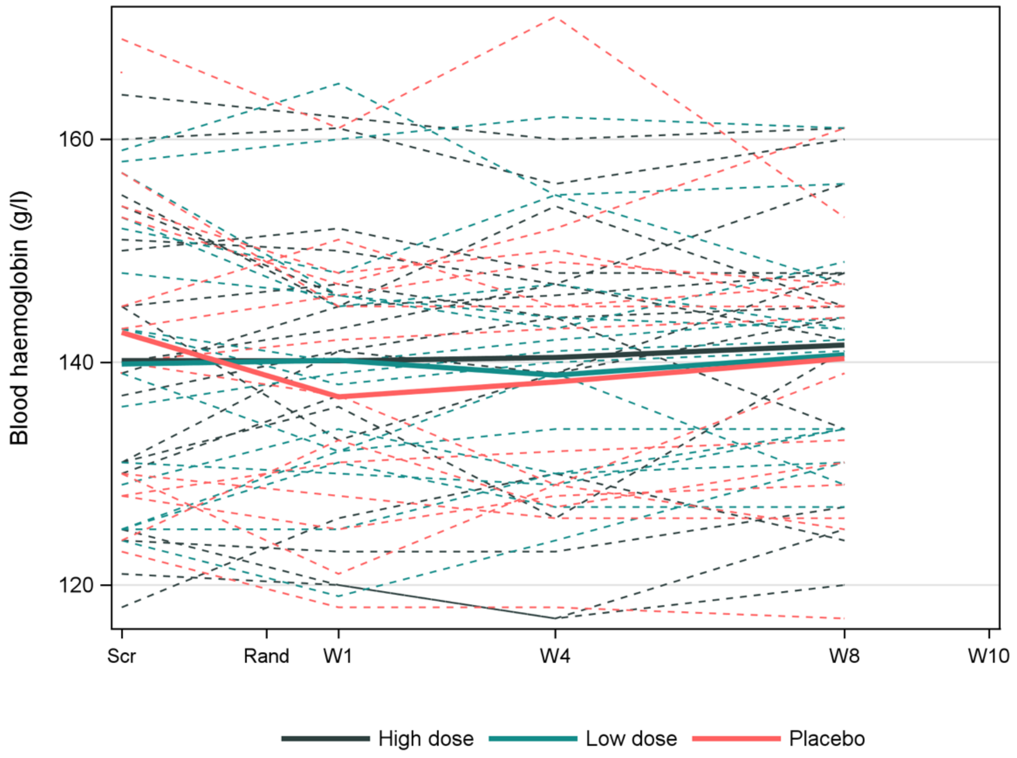

Figure 14.2.4.1.1 Boxplots for Fasting blood glucose (mmol/L) over time

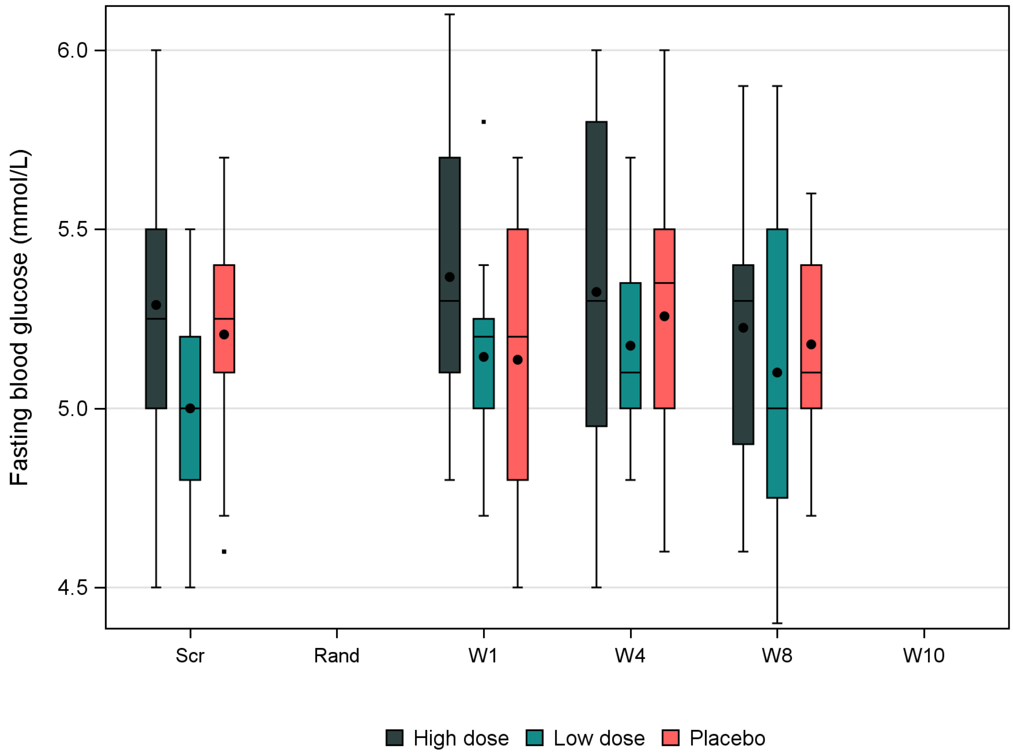

Figure 14.2.4.1.2 Individual values for Fasting blood glucose (mmol/L) over time

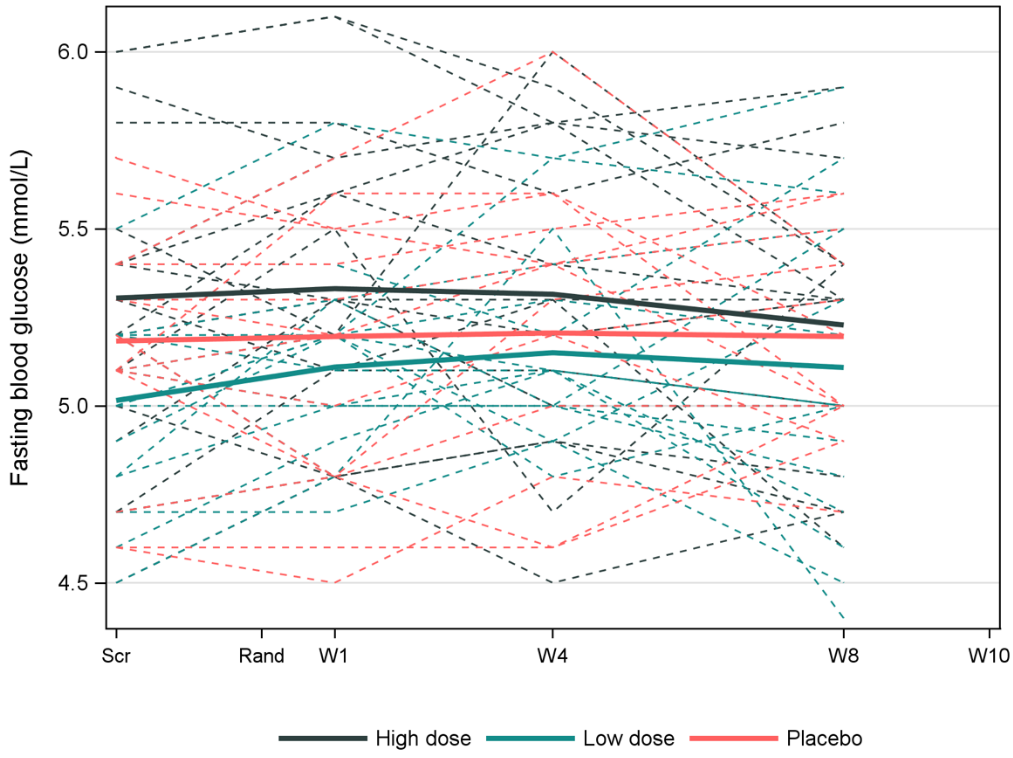

Figure 14.2.4.2.1 Boxplots for Blood HBA1C (mmol/mol) over time

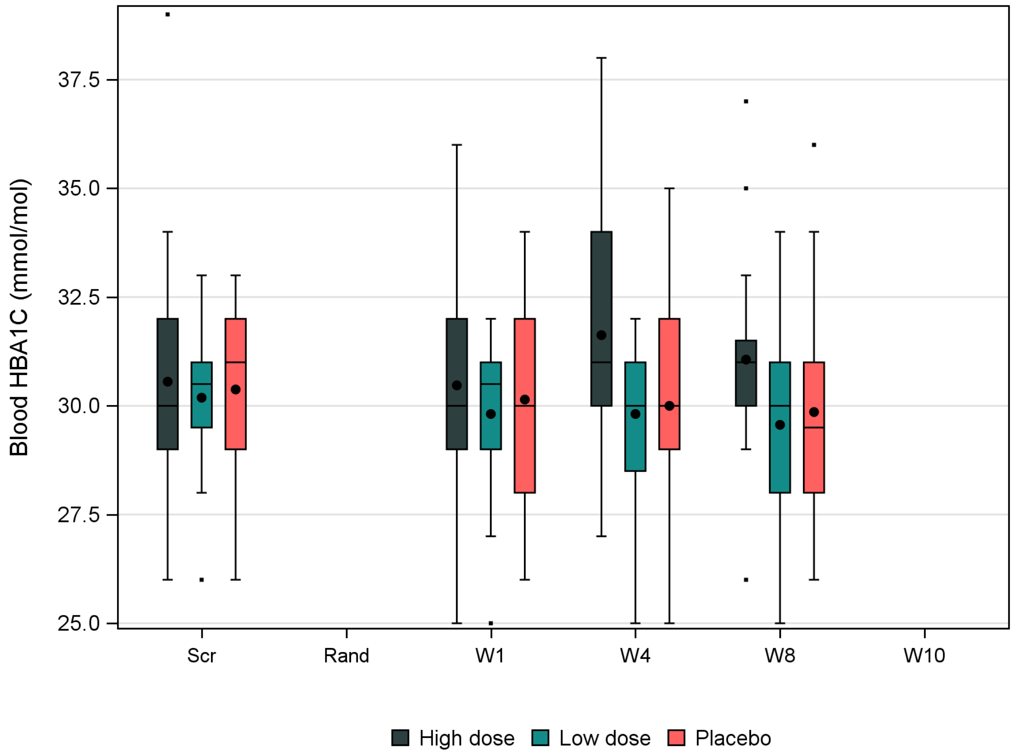

Figure 14.2.4.2.2 Individual values for Blood HBA1C (mmol/mol) over time

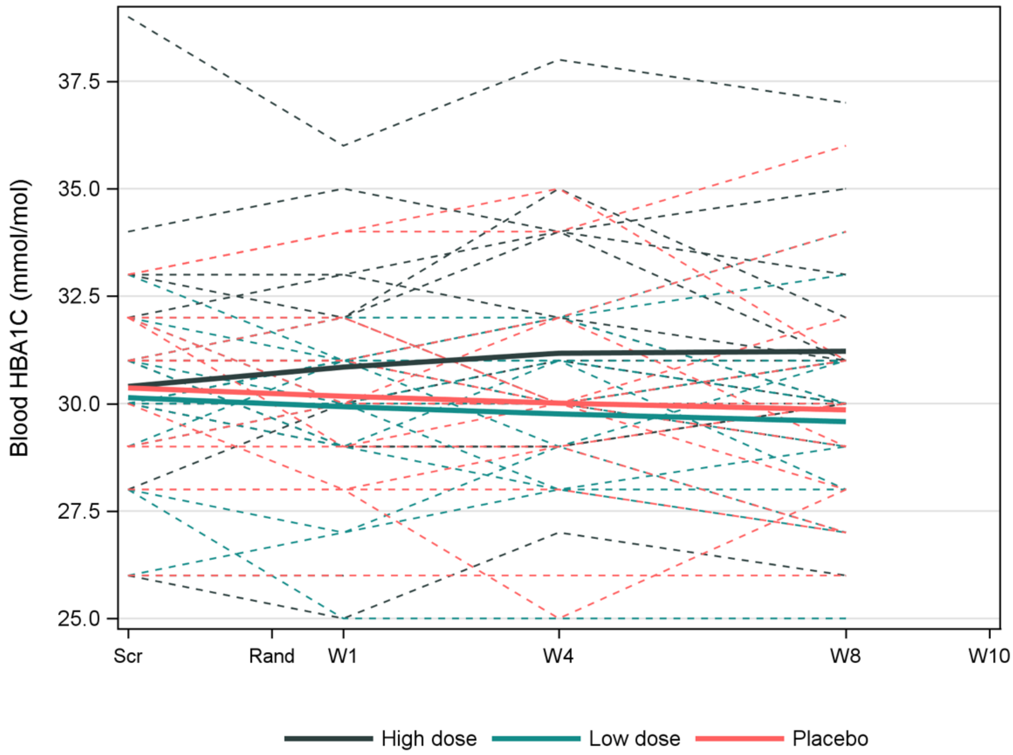

Figure 14.2.5.1.1 Boxplots for Butyrate over time

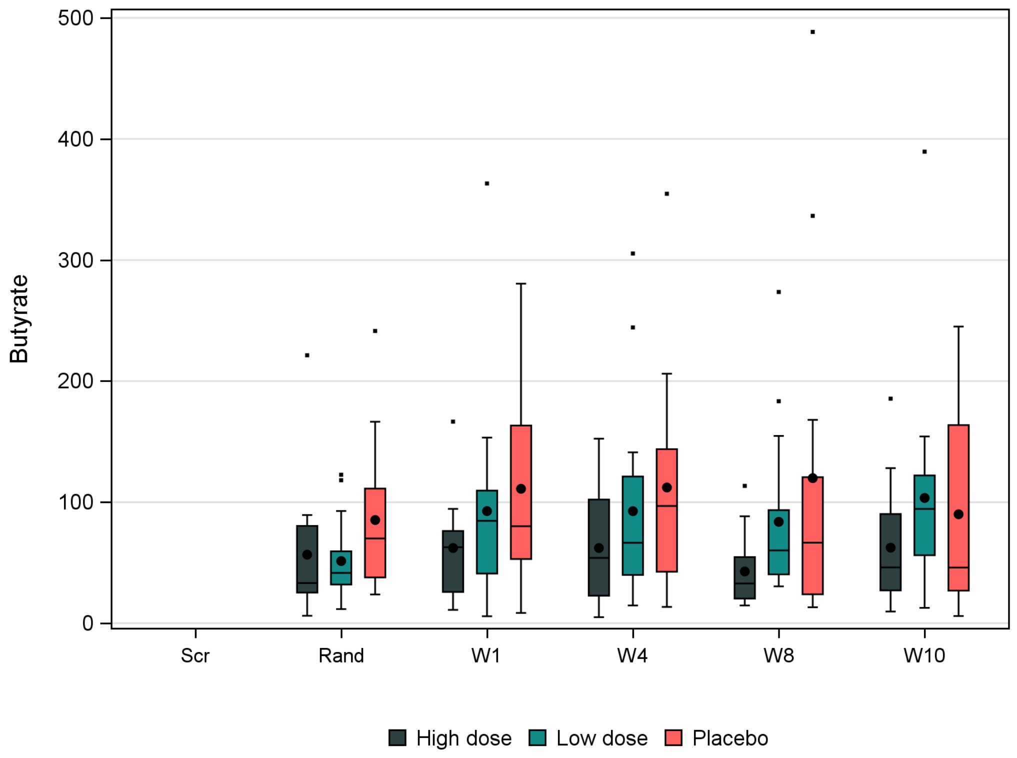

Figure 14.2.5.1.2 Individual values for Butyrate over time

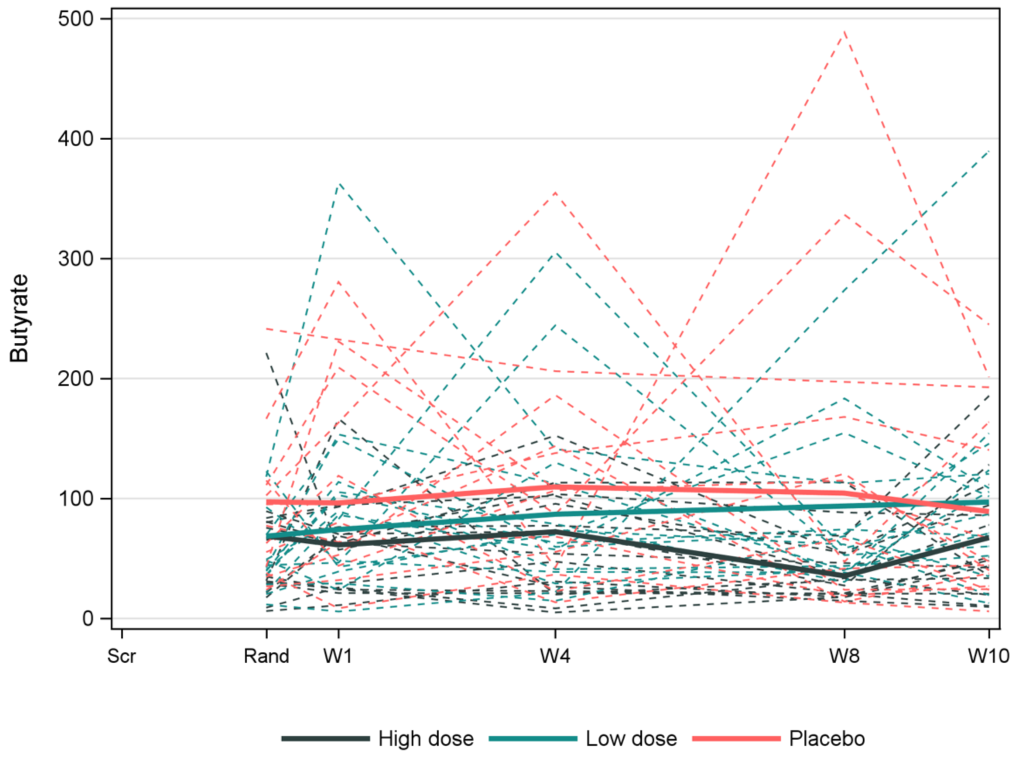

Figure 14.2.5.2.1 Boxplots for Propionate over time

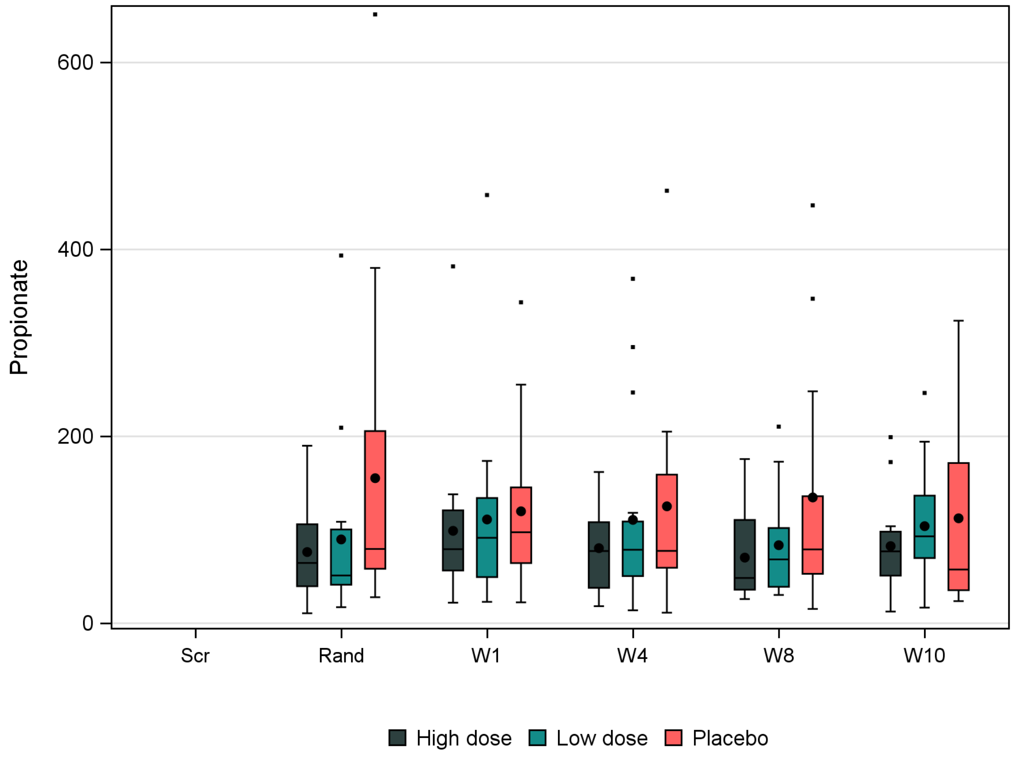

Figure 14.2.5.2.2 Individual values for Propionate over time

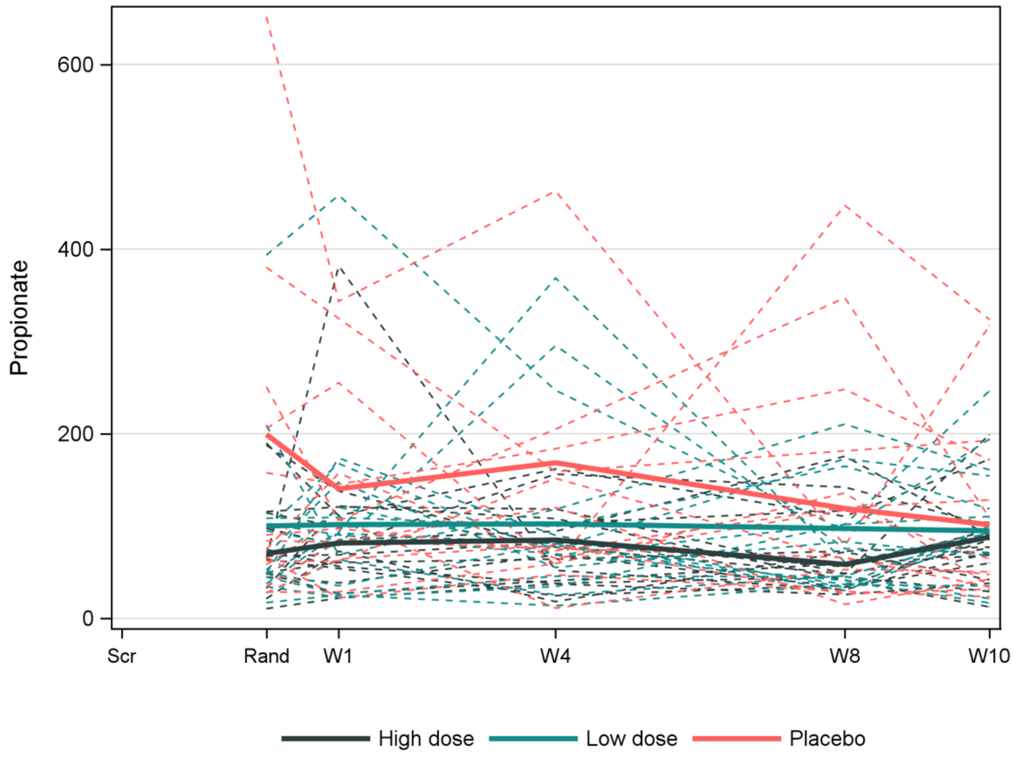

Figure 14.2.5.3.1 Boxplots for Acetate over time

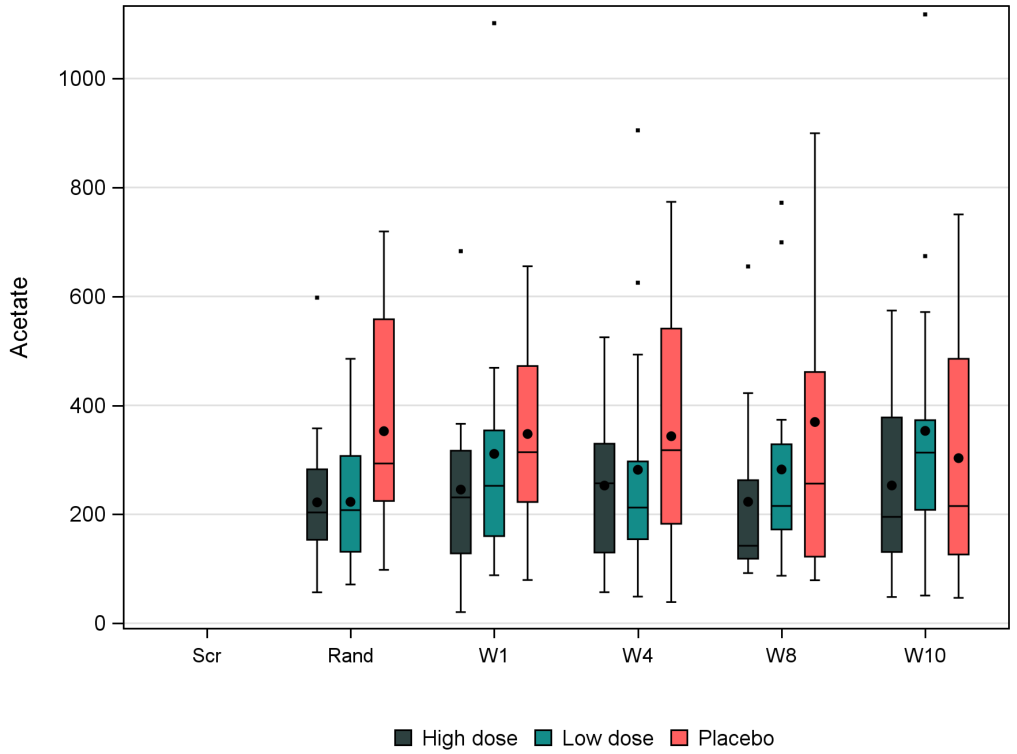

Figure 14.2.5.3.2 Individual values for Acetate over time

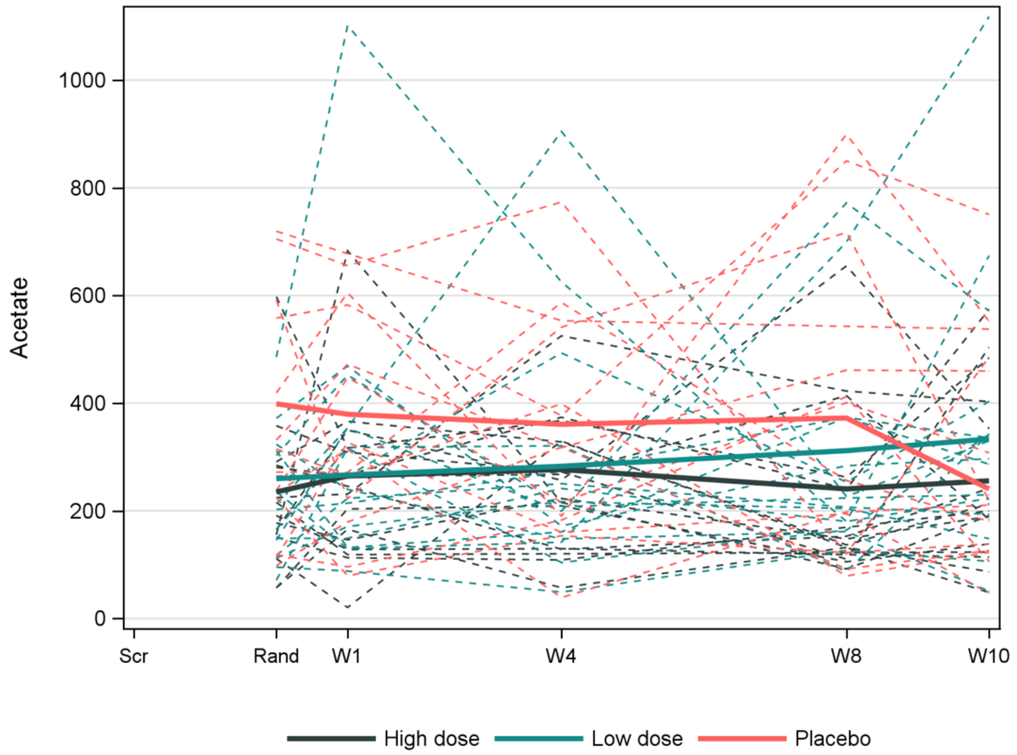

Figure 14.2.5.4.1 Boxplots for Lactate over time

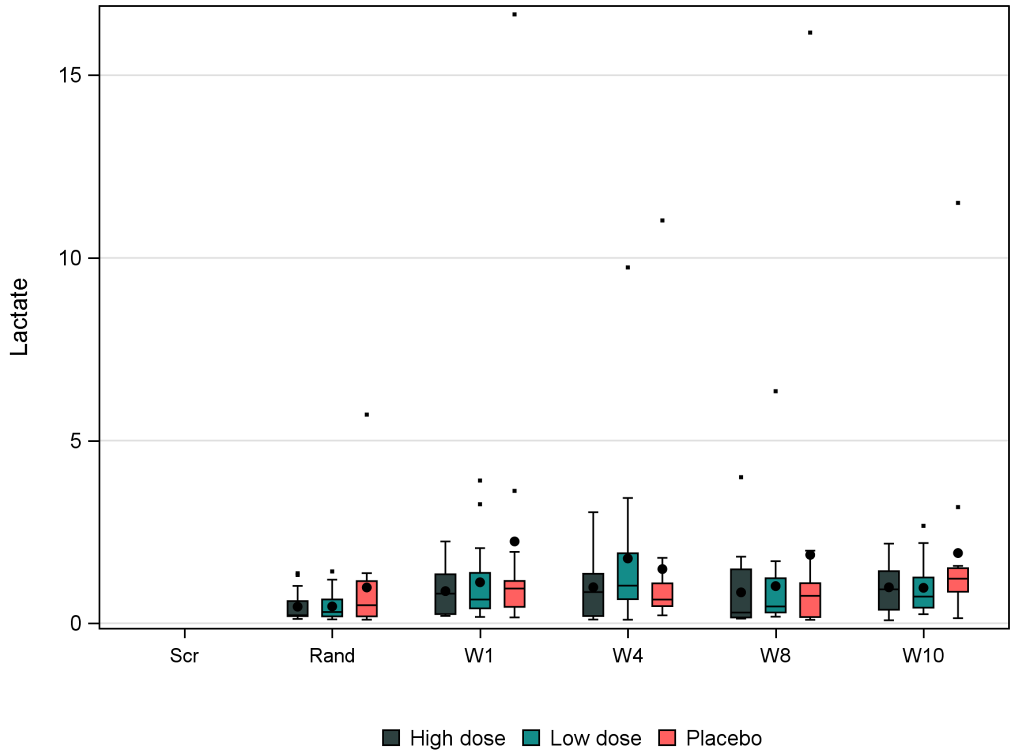

Figure 14.2.5.4.2 Individual values for Lactate over time

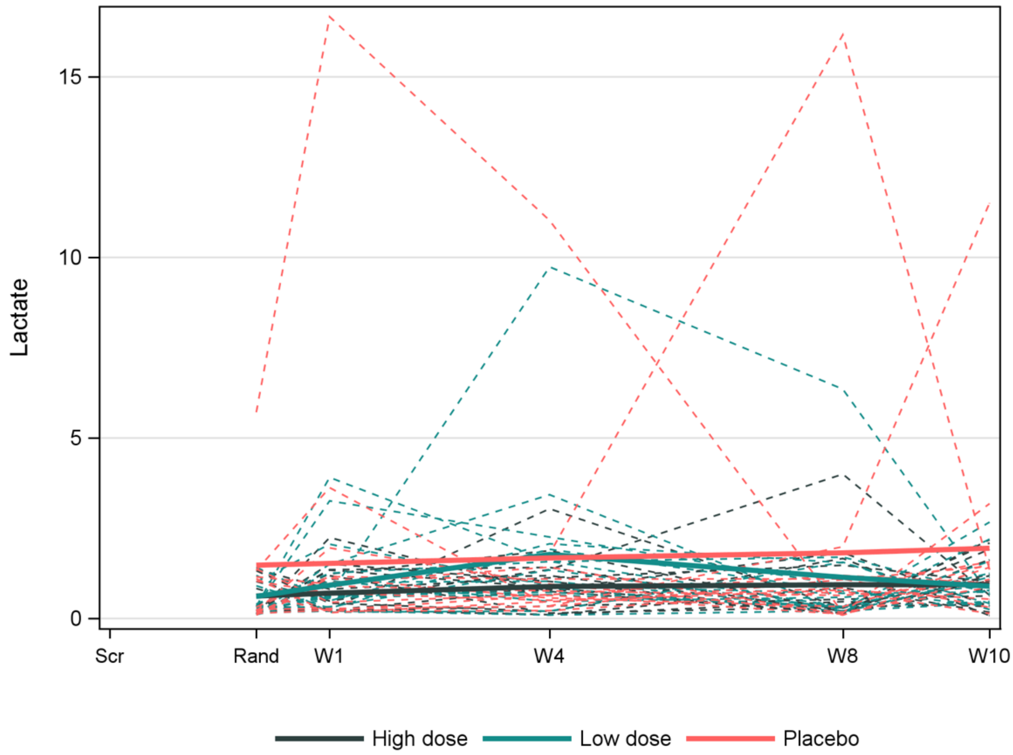

Figure 14.2.5.5.1 Boxplots for Isobutyrate over time

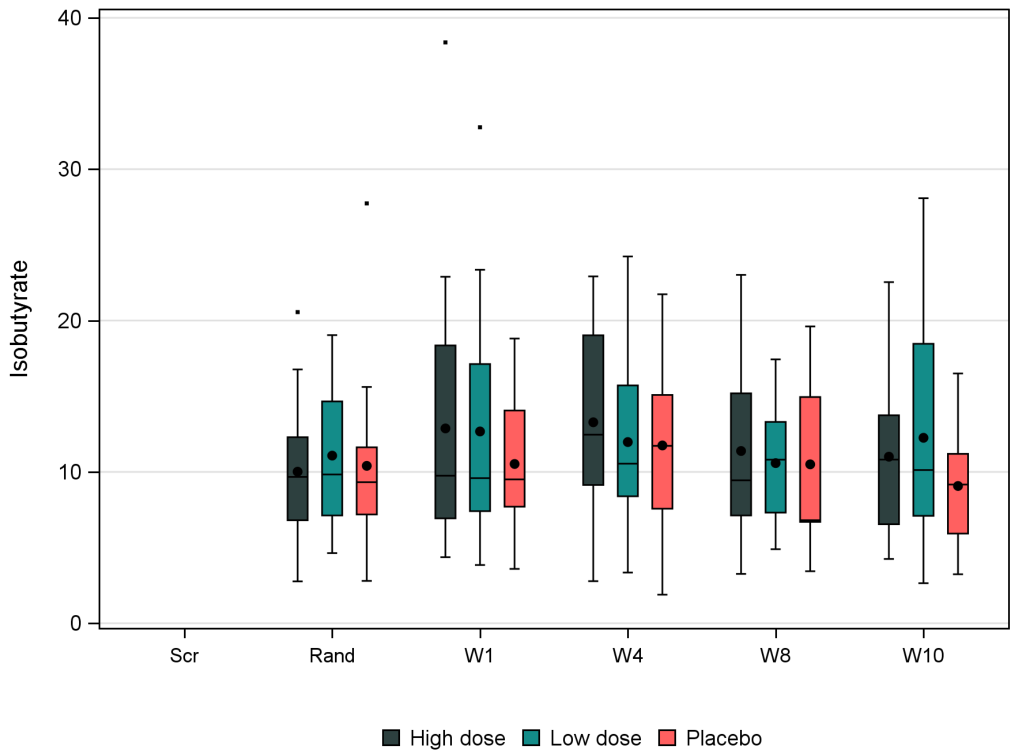

Figure 14.2.5.5.2 Individual values for Isobutyrate over time

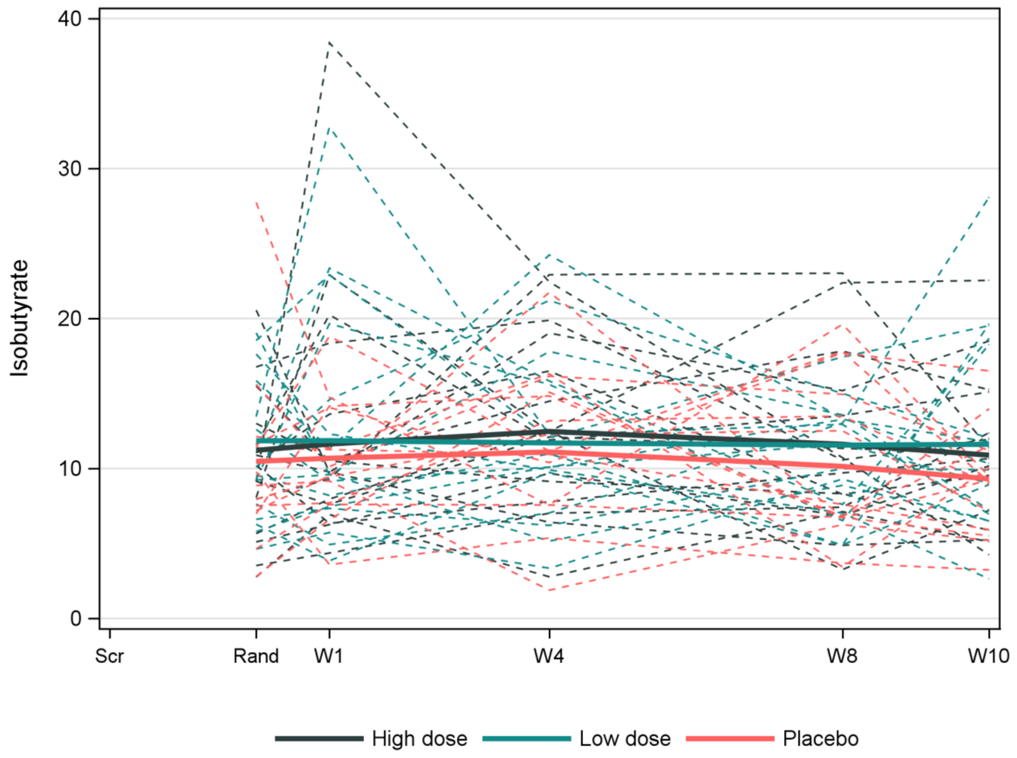

Figure 14.2.5.6.1 Boxplots for Isovalerate over time

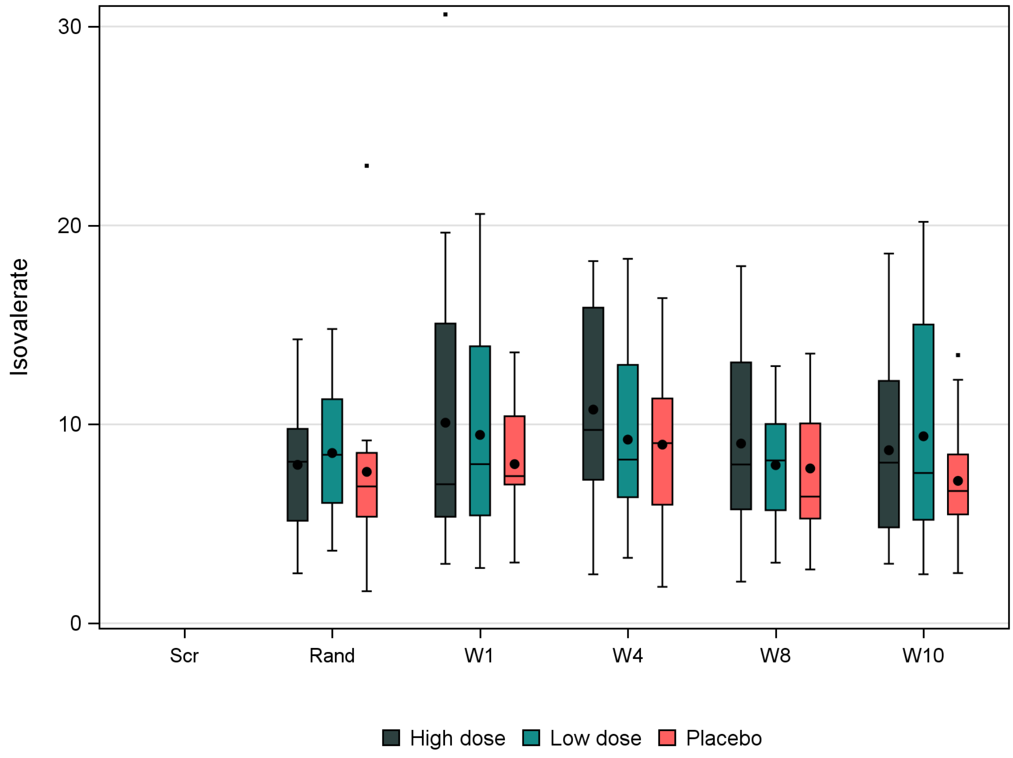

Figure 14.2.5.6.2 Individual values for Isovalerate over time

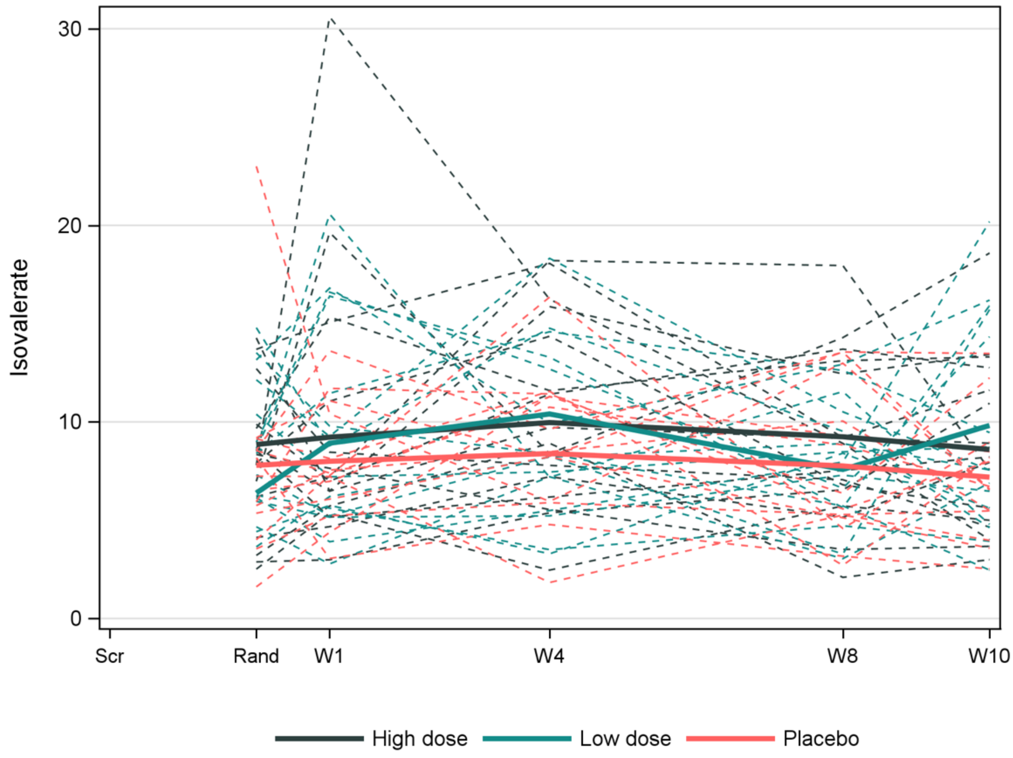

Figure 14.2.5.7.1 Boxplots for Succinate over time

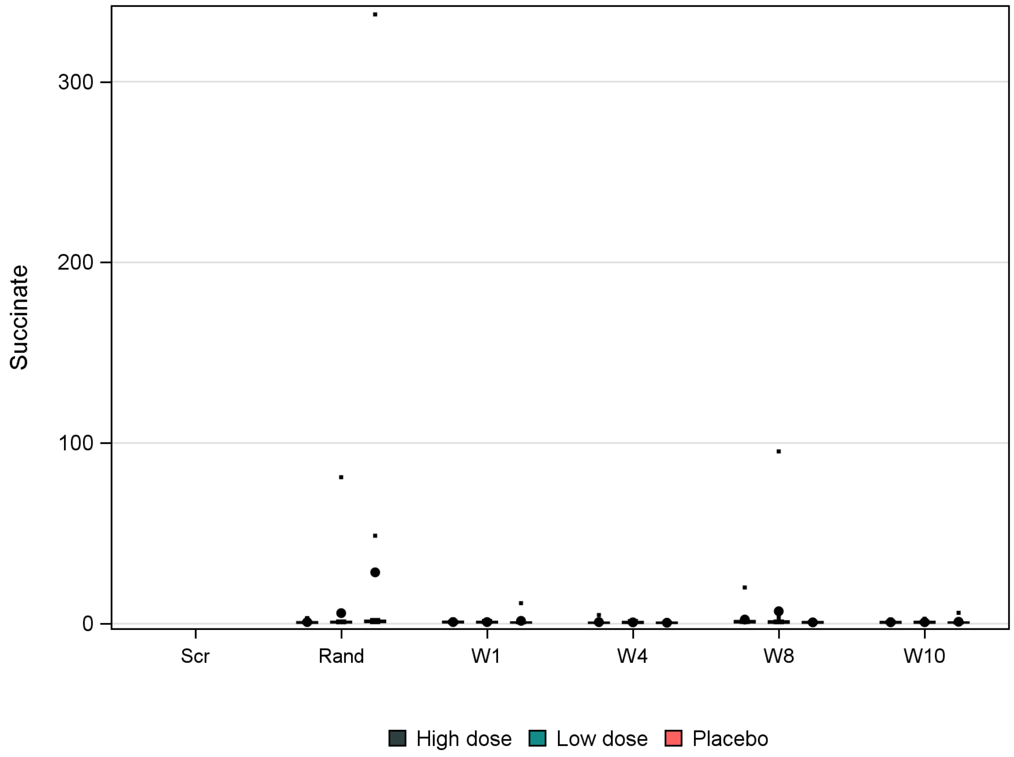

Figure 14.2.5.7.2 Individual values for Succinate over time

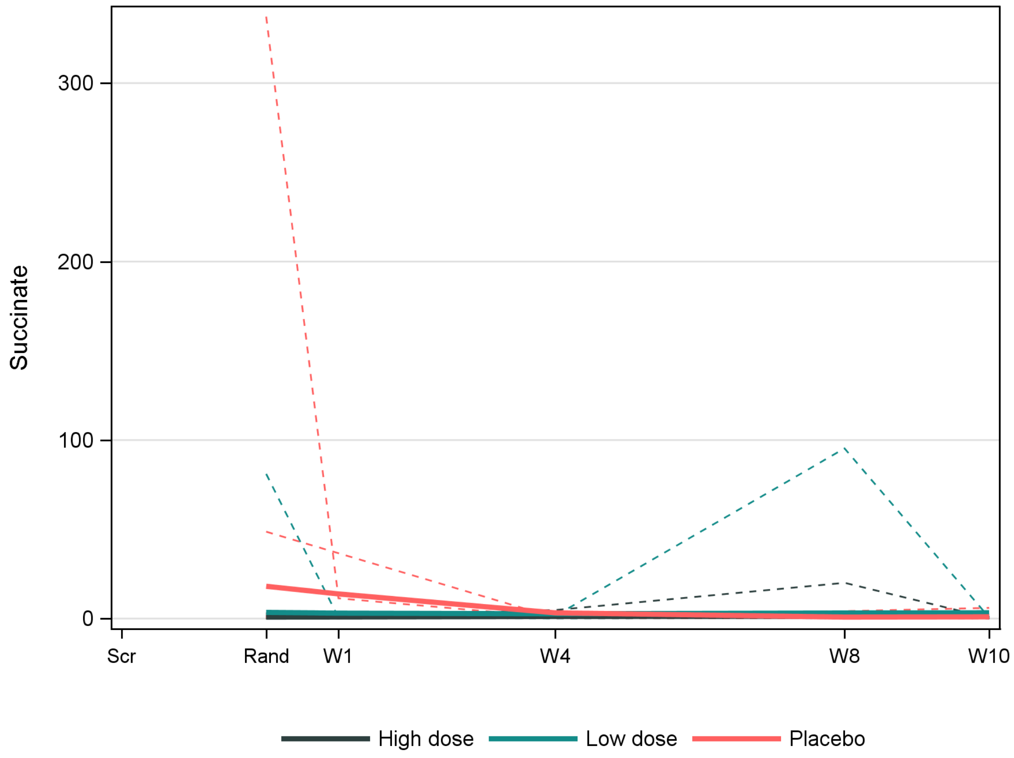

Figure 14.2.6.1.1 Boxplots for Colonization with F. prausnitzii in stool SL3.3 over time

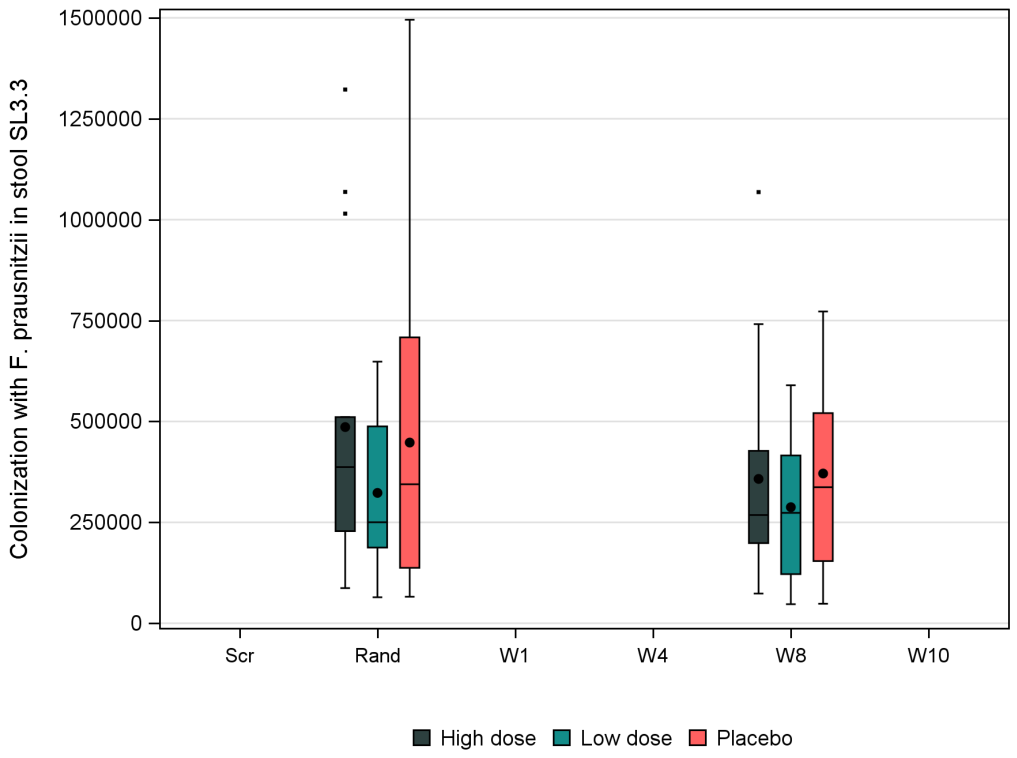

Figure 14.2.6.1.2 Individual values for Colonization with F. prausnitzii in stool SL3.3 over time

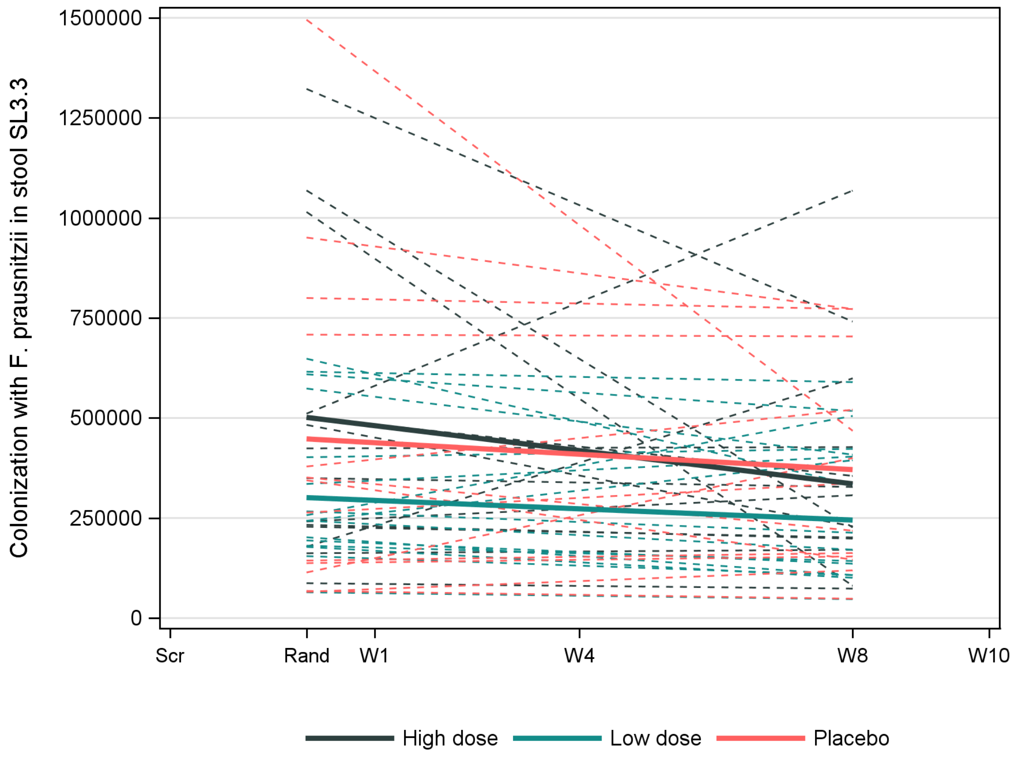

Figure 14.2.6.2.1 Boxplots for Colonization with F. prausnitzii in stool L2.6 over time

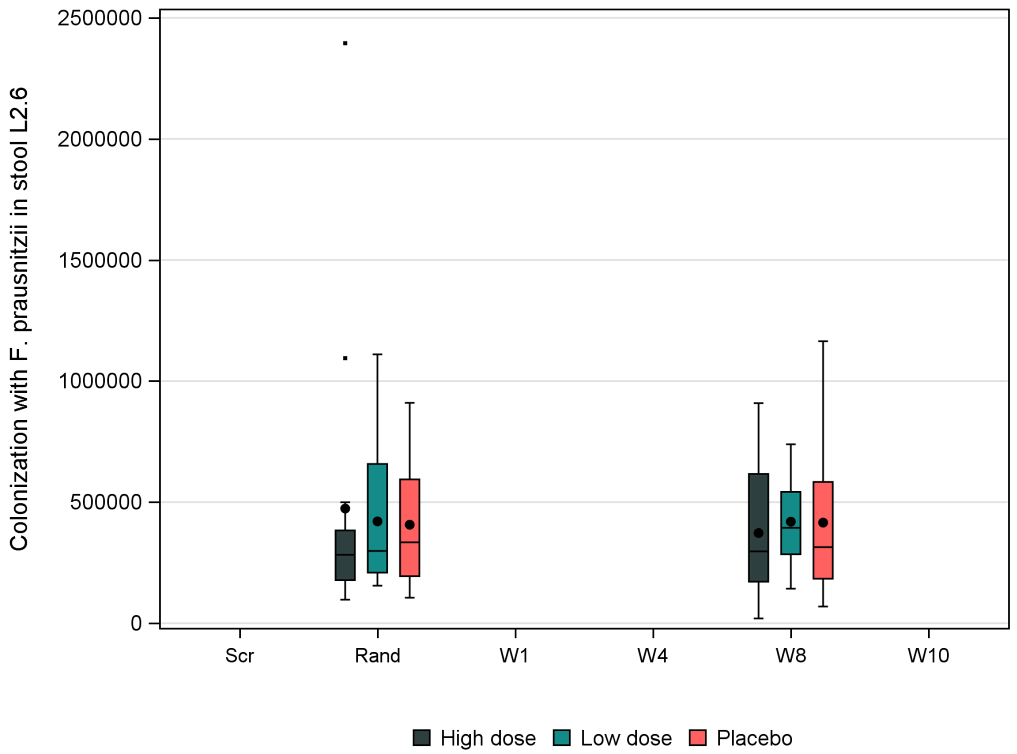

Figure 14.2.6.2.2 Individual values for Colonization with F. prausnitzii in stool L2.6 over time

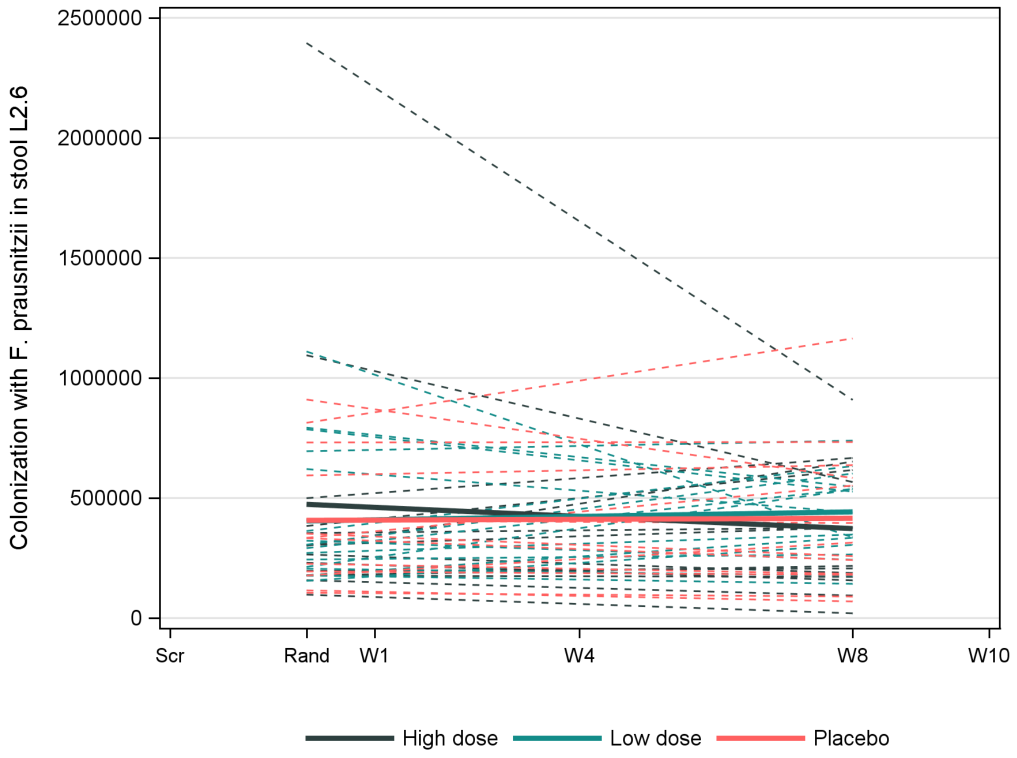

Figure 14.2.6.3.1 Boxplots for Colonization with F. prausnitzii in stool KLE1255 over time

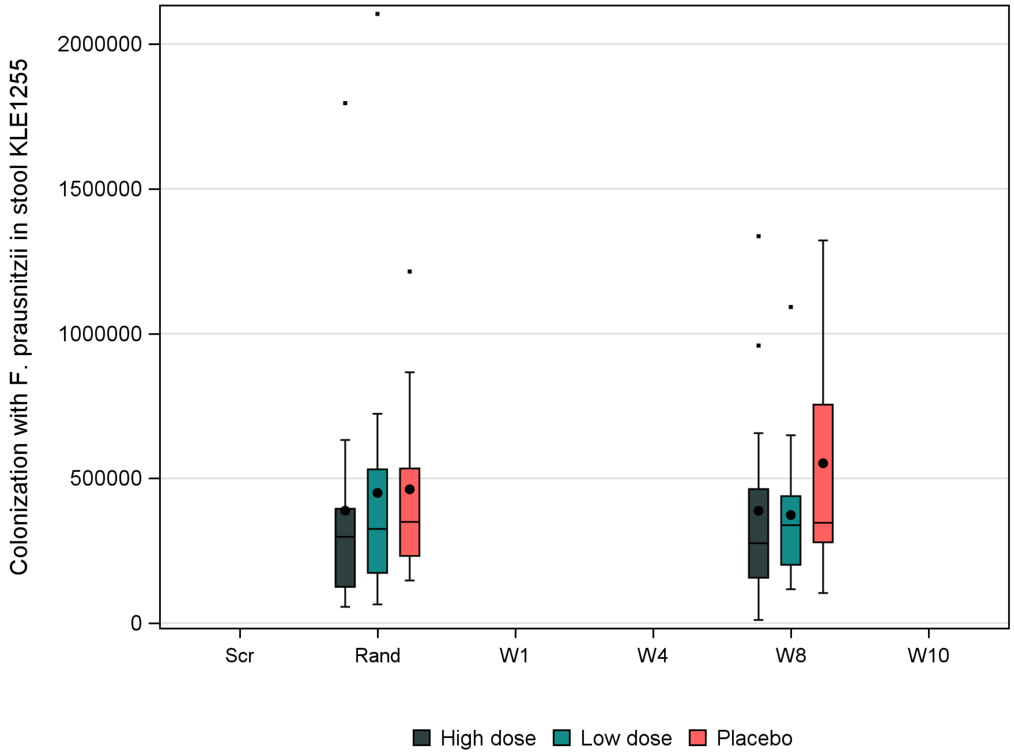

Figure 14.2.6.3.2 Individual values for Colonization with F. prausnitzii in stool KLE1255 over time

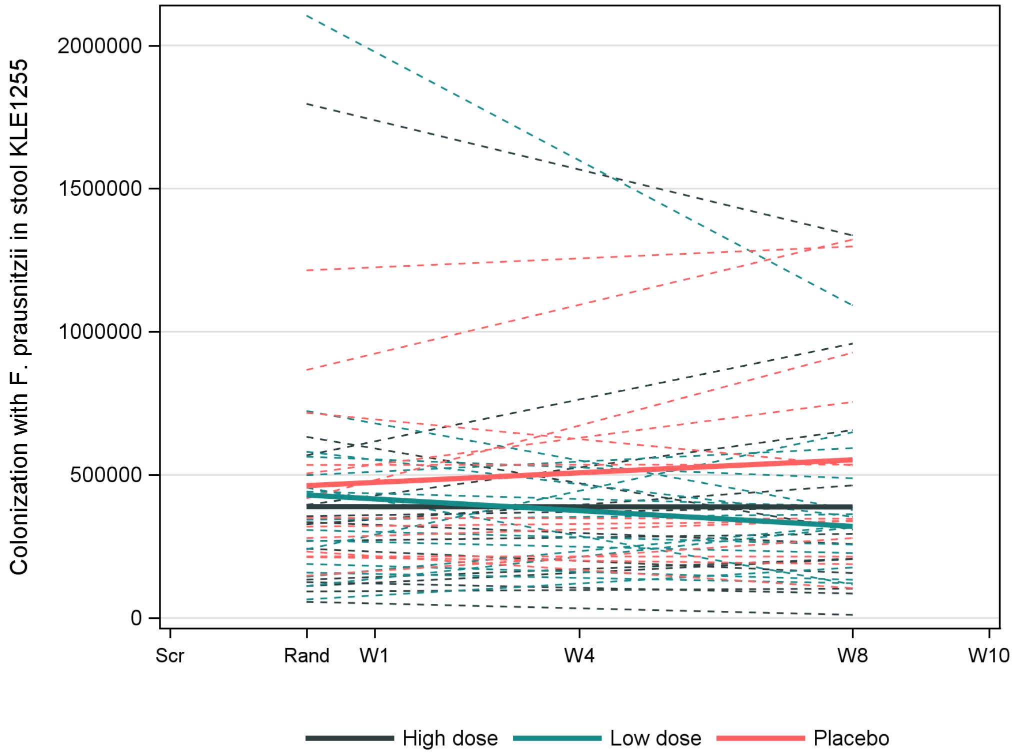

Figure 14.2.6.4.1 Boxplots for Colonization with F. prausnitzii in stool A2.165 over time

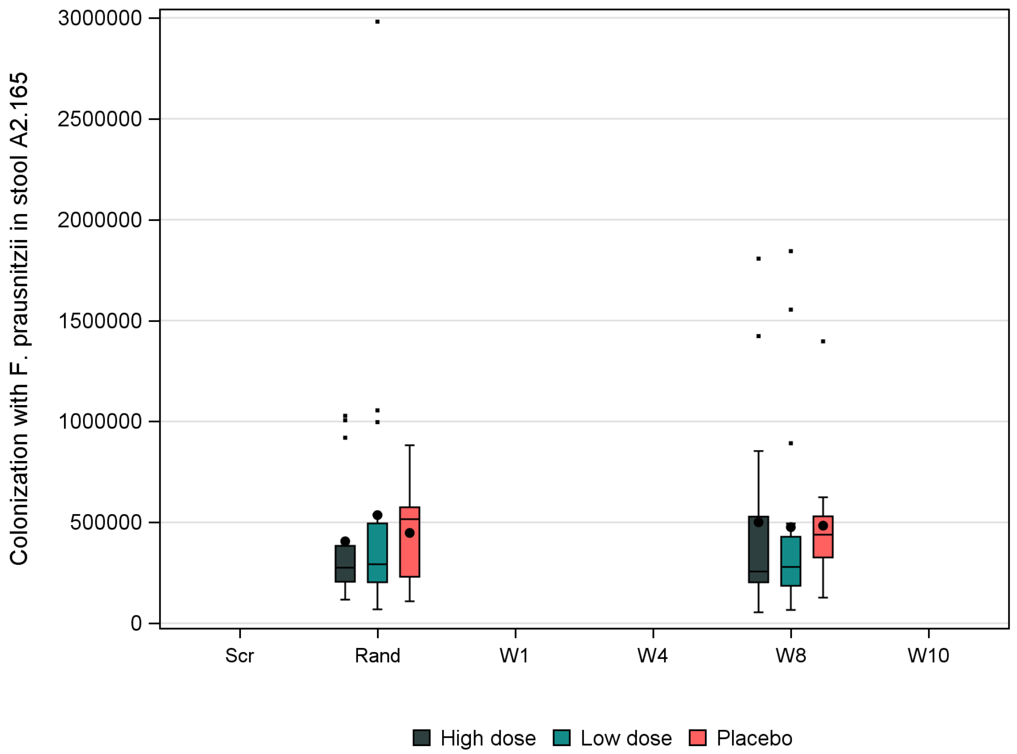

Figure 14.2.6.4.2 Individual values for Colonization with F. prausnitzii in stool A2.165 over time

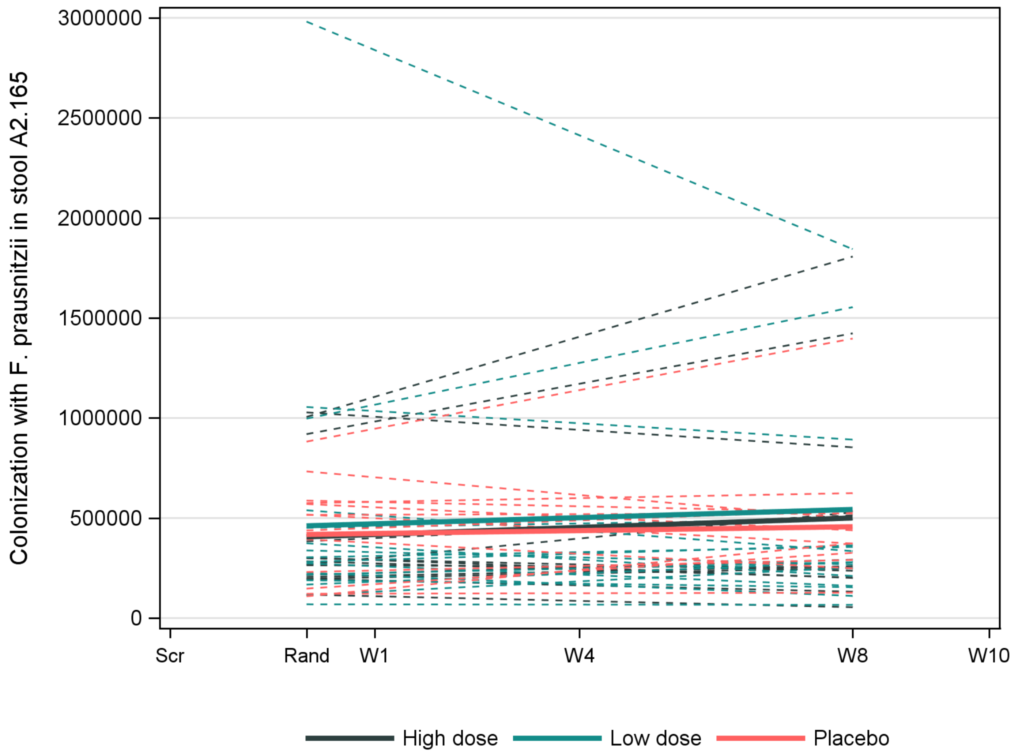

Figure 14.2.6.5.1 Boxplots for Colonization with F. prausnitzii in stool Total over time

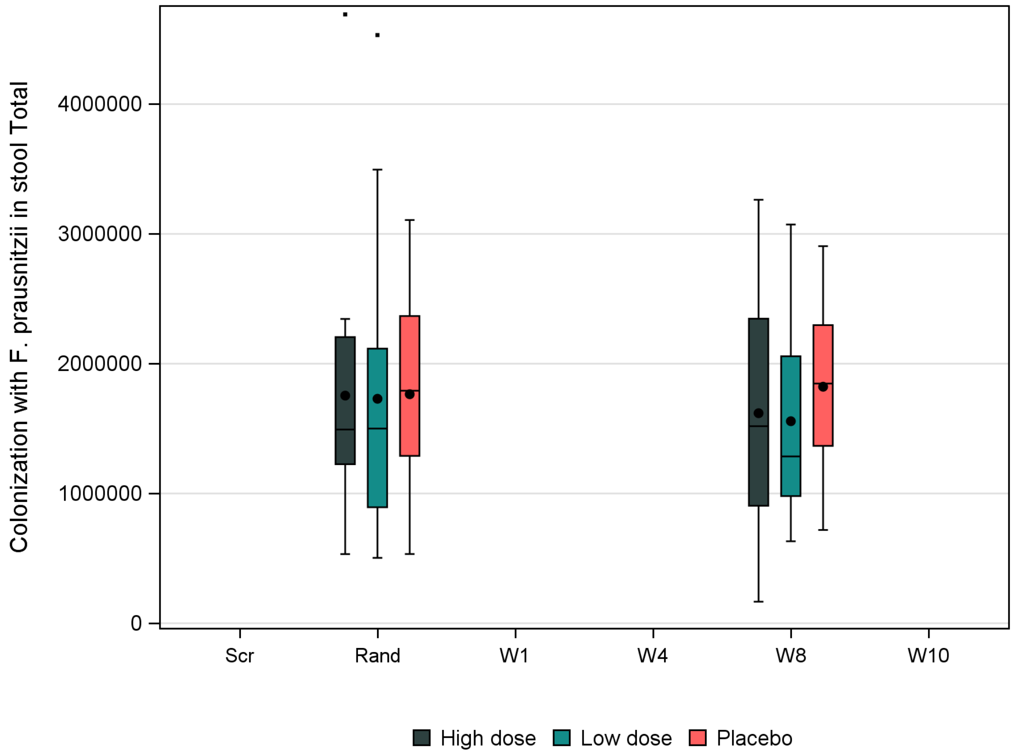

Figure 14.2.6.5.2 Individual values for Colonization with F. prausnitzii in stool Total over time

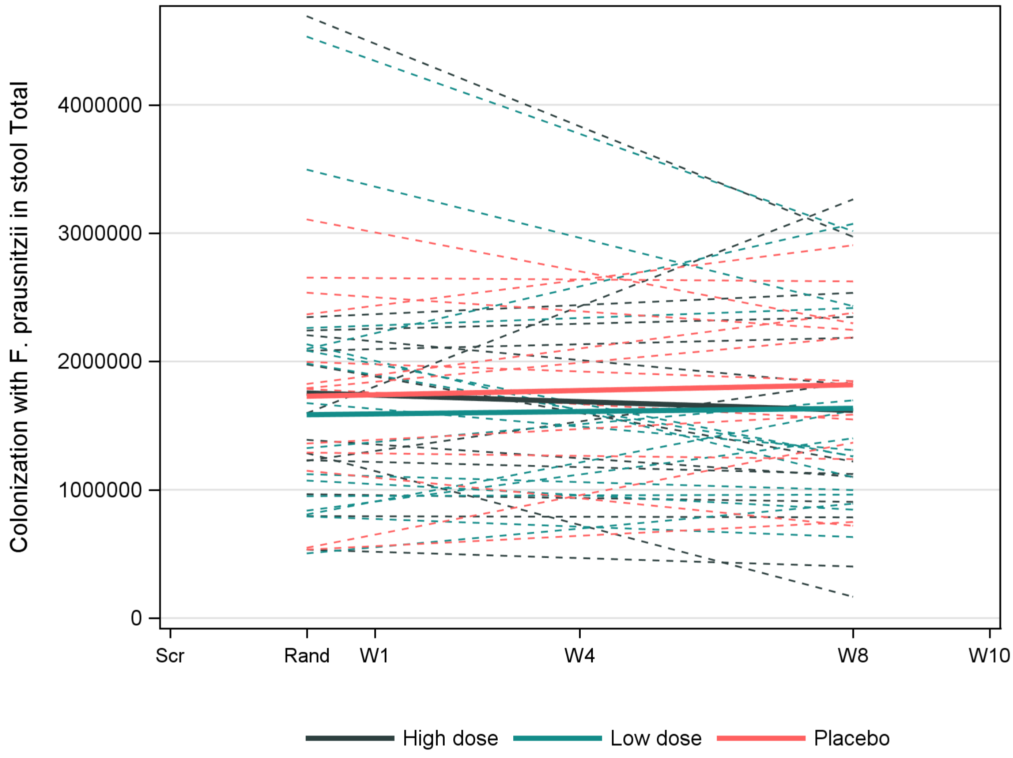

Figure 14.2.6.6.1 Boxplots for Colonization with D. Piger in stool over time

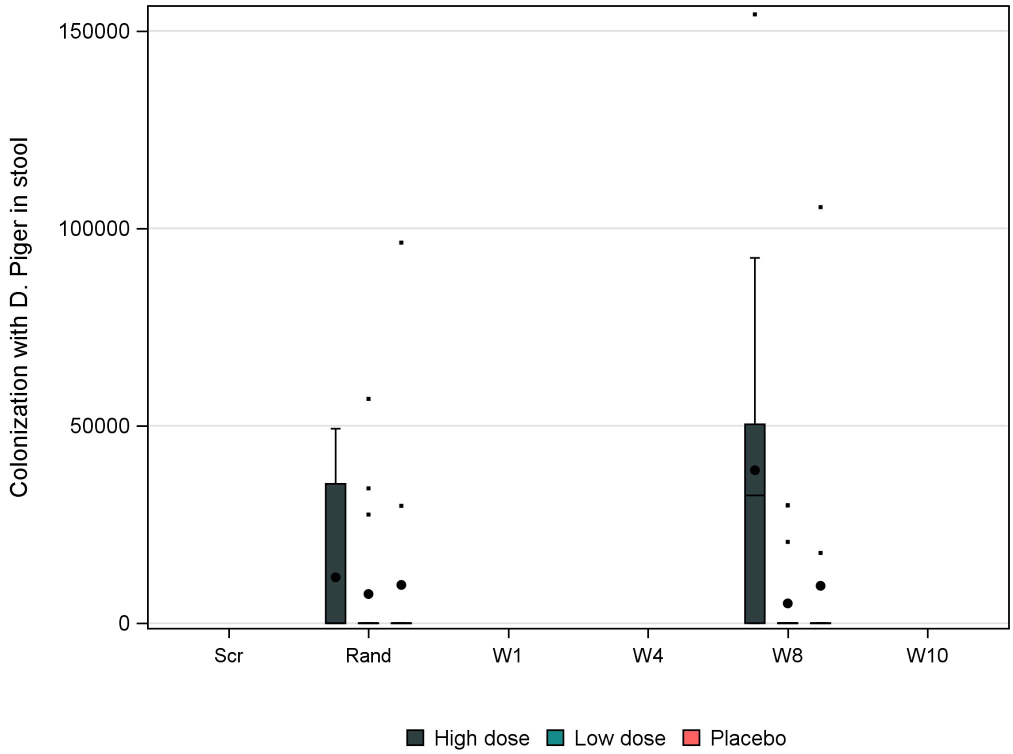

Figure 14.2.6.6.2 Individual values for Colonization with D. Piger in stool over time

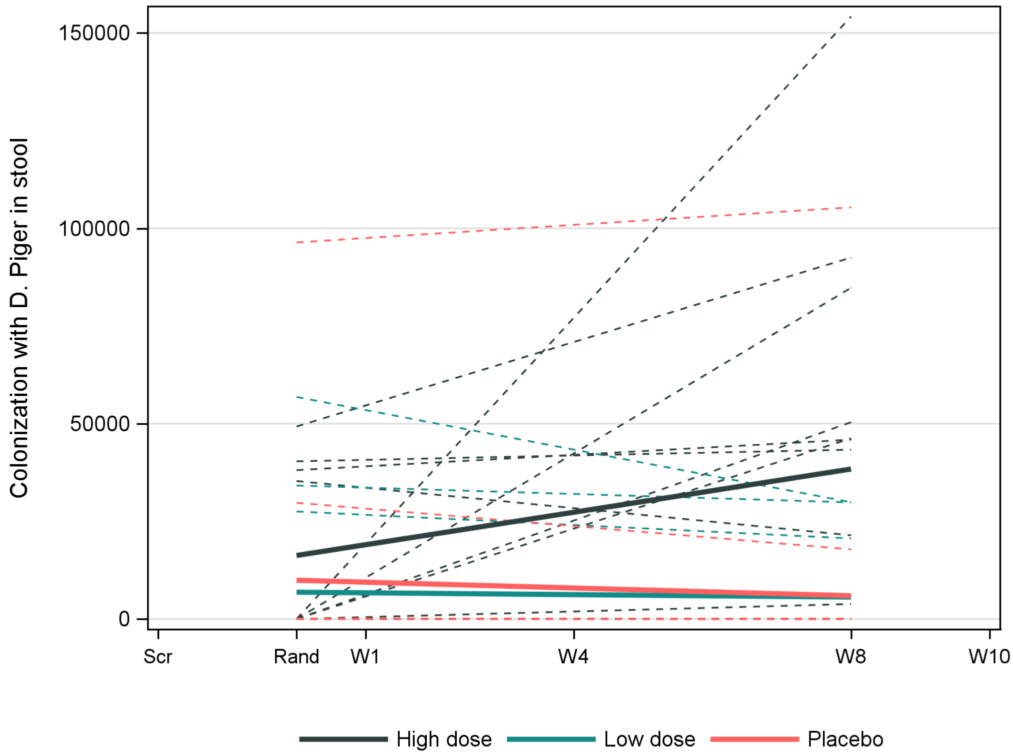

Supplement: Supplementary file 1 — Supplementary Information containing Supplementary Tables 1–7, trial details and statistical report. [file 41586_2023_6378_MOESM1_ESM.pdf]
