# Supplementary material for: Synergy and oxygen adaptation for development of next-generation probiotics
Source: Nature. 2023 Aug 2;620(7973):381–5. doi: 10.1038/s41586-023-06378-w (PMC10412450; doi:10.1038/s41586-023-06378-w)
Supplement: Supplementary file 2 — Reporting Summary [file 41586_2023_6378_MOESM2_ESM.pdf]

nature research

Corresponding author(s): Fredrik Bäckhed

Last updated by author(s): May 22, 2023

Reporting Summary

Nature Research wishes to improve the reproducibility of the work that we publish. This form provides structure for consistency and transparency in reporting. For further information on Nature Research policies, see our Editorial Policies and the Editorial Policy Checklist.

Statistics

For all statistical analyses, confirm that the following items are present in the figure legend, table legend, main text, or Methods section.

n/a	Confirmed	
☐	☒	The exact sample size (*n*) for each experimental group/condition, given as a discrete number and unit of measurement
☐	☒	A statement on whether measurements were taken from distinct samples or whether the same sample was measured repeatedly
☐	☒	The statistical test(s) used AND whether they are one- or two-sided<br>*Only common tests should be described solely by name; describe more complex techniques in the Methods section.*
☐	☒	A description of all covariates tested
☒	☐	A description of any assumptions or corrections, such as tests of normality and adjustment for multiple comparisons
☐	☒	A full description of the statistical parameters including central tendency (e.g. means) or other basic estimates (e.g. regression coefficient) AND variation (e.g. standard deviation) or associated estimates of uncertainty (e.g. confidence intervals)
☐	☒	For null hypothesis testing, the test statistic (e.g. *F*, *t*, *r*) with confidence intervals, effect sizes, degrees of freedom and *P* value noted<br>*Give P values as exact values whenever suitable.*
☒	☐	For Bayesian analysis, information on the choice of priors and Markov chain Monte Carlo settings
☒	☐	For hierarchical and complex designs, identification of the appropriate level for tests and full reporting of outcomes
☒	☐	Estimates of effect sizes (e.g. Cohen's *d*, Pearson's *r*), indicating how they were calculated

Our web collection on statistics for biologists contains articles on many of the points above.

Software and code

Policy information about availability of computer code

Data collection

No software was used for data collection

Data analysis

GraphPad Prism 8.4.3.
Statistics on clinical data were performed with SAS Software version 9.4 (SAS Institute Inc., Cary, NC, USA). Confidence intervals form primary outcomes were calculated using the Newcombe hybrid score interval. Permutation-based confidence intervals (Extended Data Table 5-8) were calculated using a user-written SAS macro.
The Unicycler pipeline v0.4.8 in hybrid mode used to obtain de novo assemblies. All dependencies for Unicycler were installed in a conda environment. The dependency programs include SPAdes v3.13.0, racon v1.4.1, bowtie2 v2.3.5.1, and pilon v1.23. The hybrid assemblies were annotated using Prokka v1.14.5 (https://github.com/tseemann/prokka).
progressiveMauve and MEGAx were used to producer phylogenetic trees.
Genetic variants were detected using snippy v4.4.5 in default setting (https://github.com/tseemann/snippy).
For production of high quality reads fastX toolkit (https://github.com/lianos/fastx-toolkit/) and Bowtie253 (v2.4.4) were used.
For annotation and quantification of species in metagenome data Kraken 2 (v2.1.2) RefSeq database (release 107) and Bracken (v2.6.2) were used.
Hidden markov models were used to screen microbial genes .
Vegan package in R (https://github.com/vegandevs/vegan/) was used for microbiota composition analysis.

For manuscripts utilizing custom algorithms or software that are central to the research but not yet described in published literature, software must be made available to editors and reviewers. We strongly encourage code deposition in a community repository (e.g. GitHub). See the Nature Research guidelines for submitting code & software for further information.

Data

Policy information about availability of data

All manuscripts must include a data availability statement. This statement should provide the following information, where applicable:

- Accession codes, unique identifiers, or web links for publicly available datasets
- A list of figures that have associated raw data
- A description of any restrictions on data availability

Supplementary information on data availability is linked to the online version of the paper at www.nature.com/nature. Genome assemblies and raw metagenomic sequence data have been deposited in the EMBL-EBI European Nucleotide Archive (ENA) under accession number PRJEB62463. Processed sequence data required for reanalysis of the results can be made available by the corresponding author upon request. Processed pseudonymized per-subject metadata are provided in Supplementary Tables 2–9. Source data can be made available by the corresponding author upon request. For questions on the clinical cohort contact M.L. Bacterial strains are proprietary of Metabogen AB and should be requested from them.

Field-specific reporting

Please select the one below that is the best fit for your research. If you are not sure, read the appropriate sections before making your selection.

☒ Life sciences ☐ Behavioural & social sciences ☐ Ecological, evolutionary & environmental sciences

For a reference copy of the document with all sections, see nature.com/documents/nr-reporting-summary-flat.pdf

Life sciences study design

All studies must disclose on these points even when the disclosure is negative.

Sample size	Statistical power in the clinical trial "Tolerability and Risk of Adverse Events with a Probiotic Supplement: A Randomised and Placebo Controlled Study in Healthy Individuals" was calculated based on anticipated differences in the proportions of study subjects discontinuing due to adverse events. With a discontinuation rate of 0.50 vs. 0.05 due to investigational product in the two treatment groups vs. placebo group (randomized in 2:1, 32 vs. 16 subjects), respectively, with an alpha level of 0.05, using the two-sided Fisher's exact test, a power of 88% was achieved.<br>No sample size calculation was performed for mouse studies.
Data exclusions	No data was excluded
Replication	In vitro studies were repeated at least in three independent experiments for the main figures.
Randomization	Study subjects were randomized to three arms: High dose investigational product (IP), low dose IP and placebo. Randomization was carried out using blocks with varying block size and was stratified by sex.
Blinding	Treatment allocation was blinded for the participants, care provider, investigator, and outcomes assessor. Blinding was maintained until study end and completion of the clean file (final database) and statistical analysis plan.<br>In the mouse study clinical observations, body weight, food consumption, organ weight assessments, and autopsies was performed without blinding. Blood haematology, clinical biochemistry and histopathology was assessed by blinded external personnel.<br>For practical reasons, no blinding was performed in the in vitro studies

Reporting for specific materials, systems and methods

We require information from authors about some types of materials, experimental systems and methods used in many studies. Here, indicate whether each material, system or method listed is relevant to your study. If you are not sure if a list item applies to your research, read the appropriate section before selecting a response.

Materials & experimental systems

n/a	Involved in the study	
☒	☐	Antibodies
☐	☒	Eukaryotic cell lines
☒	☐	Palaeontology and archaeology
☐	☒	Animals and other organisms
☐	☒	Human research participants
☐	☒	Clinical data
☒	☐	Dual use research of concern

Methods

n/a	Involved in the study	
☒	☐	ChIP-seq
☒	☐	Flow cytometry
☒	☐	MRI-based neuroimaging

Eukaryotic cell lines

Policy information about cell lines

Cell line source(s)	CACO-2 from ATCC
Authentication	The cell line was not authenticated.
Mycoplasma contamination	All cell lines were negative mycoplasma
Commonly misidentified lines (See ICLAC register)	none

Animals and other organisms

Policy information about studies involving animals; ARRIVE guidelines recommended for reporting animal research

Laboratory animals	Mice, Swiss Webster, 8 weeks, Male and females 8 weeks old Swiss Webster mice were co-housed with 5 mice/cage at a temperature of 20±1°C and an air humidity of 45-70% under specific pathogen-free conditions at a 12-h light/dark cycle (light from 7 am to 7 pm) and were fed an autoclaved chow diet (LabDiet, St. Louis, MO, USA) and water ad libitum.
Wild animals	The study did not involve wild animals
Field-collected samples	The study did not involve field-collected samples
Ethics oversight	Mouse experiments were approved by the Research Animal Ethics Committee in Gothenburg.

Note that full information on the approval of the study protocol must also be provided in the manuscript.

Human research participants

Policy information about studies involving human research participants

Population characteristics	In total, 50 healthy individuals (men and women) between 20 and 40 years old were included.
Recruitment	Study subjects were recruited from the general population using advertisements in social networks, news papers and posters in public areas in Mölndal and Gothenburg.<br>Only healthy individuals were included. Thus, the study participants are not necessarily representative for the general population at the same ages. However, this issue cannot be fully avoided as phase 1 trials should be based on healthy individuals in order to minimize risks of previously untested supplements or drugs. More importantly, the trial was a randomized, placebo-controlled trial, and comparisons between individuals randomized to active treatment and placebo should therefore not be affected by any inclusion bias.
Ethics oversight	The study was approved by the Regional Ethics Review Board in Gothenburg.

Note that full information on the approval of the study protocol must also be provided in the manuscript.

Clinical data

Policy information about clinical studies

All manuscripts should comply with the ICMJE guidelines for publication of clinical research and a completed CONSORT checklist must be included with all submissions.

Clinical trial registration	NCT03728868
Study protocol	The full trial protocol in Swedish (original) and English (certified translation) is available in the Supplementary Information.
Data collection	All recruitment was performed at the Geriatric Medicine Clinical Research Unit at the Sahlgrenska University Hospital in Mölndal. Recruitment started on October 10th, 2018 and was completed by April 2nd, 2019. The study was completed (last study visit) on May 31st, 2019.
Outcomes	The predefined primary outcome was tolerability of the oral intake of F. prausnitzii and D. piger, defined as study discontinuation due to adverse events under 8 weeks of treatment.<br><br>The predefined secondary outcomes were:<br>1. Gastrointestinal side effects, measured using the Gastrointestinal Symptom Rating Scale (GSRS).<br>Assessment of gastrointestinal symptoms the last week, was performed using the GSRS questionnaire. Gastrointestinal symptoms were measured with GSRS, which includes 15 items combined into five symptom clusters measuring 1) reflux, 2) abdominal pain, 3) indigestion, 4) diarrhea and 5) constipation. GSRS has a seven-point graded Likert-type scale, in which 1 represents absence of troublesome symptoms and 7 represents very troublesome symptoms. The total score was derived from all subclasses, resulting in a score between 0 and 45.

2. Effects on inflammation - erythrocyte sedimentation rate (safety parameter).
Change in erythrocyte sedimentation rate before and after the 8-week treatment period was assessed. Erythrocyte sedimentation rate was measured using the Starrsed ST Instrument, Mechatronics.

3. Effects on inflammation - CRP (safety parameter).
Change in C-reactive protein (CRP) level in blood. Analysis of C-reactive protein (CRP) in serum was measured by agglutinate CRP with latex particles, which in turn was covered with anti-CRP-antibodies. The aggregates were measured using turbidimetry. The coefficient of variation was 7% at 20 mg/L and 4% at 80 mg/L and the instrument used was the Cobas 6000. Change in CRP before and after the 8-week treatment period was investigated.

4. Effect on hematopoiesis - red blood cells (safety parameter).
Change in red blood cell count before and after the 8-week treatment period was examined. Erythrocyte count (CV: 3% at 2, 4, and 5 x1012/L) was measured using anti coagulated venous blood with K2-EDTA and measurement of the absorption of light. Instrument used to analyze was the ADVIA 2120i (Siemens Medical Solutions Diagnostics AB).

5. Effect on hematopoiesis - white blood cells (safety parameter).
Change in white blood cells count before and after the 8-week treatment period was examined. Leukocyte count was measured using anti coagulated venous blood with K2-EDTA and measurement of the absorption of light, using the ADVIA 2120i instrument (Siemens Medical Diagnostics AB), with a CV of 7% at concentrations 3 x 10E9/L to 16 x 10E9/L

6. Effect on hematopoiesis - platelets (safety parameter).
Changes in platelets count before and after the 8-week treatment period was examined. Thrombocyte count (CV: 9% at 80, 200, and 500 x10E9/L) was measured using anti coagulated venous blood with K2-EDTA and measurement of the absorption of light. Instrument used to analyze was the ADVIA 2120i.

7. Effects on liver enzymes - ALAT (safety parameter).
Change in liver enzyme ALT (alanine transaminase) before and after the 8-week treatment period was examined. Alanine transaminase (ALT) catalyzes the reaction between L-Alanin and 2 oxoglutarat. Further reaction between the produced pyruvate and NADH generates a measure of NADH oxidation, which was directly proportional to the ALT activity, which was measured via the decrease in absorbance. The coefficient of variation was 6% at 1 µkat/L and 4% at 4 µkat/L and the instrument used was the Cobas 6000.

8. Effects on liver enzymes - AST (safety parameter).
Change in liver enzyme AST (aspartate transaminase) before and after the 8-week treatment period was examined. Aspartate transaminase (AST) catalyzes L-Aspartate and 2-oxoglutatrat to oxaloacetate and L-glutamat. Further reaction between oxaloacetate and NADH generates a measure of NADH oxidation, which was directly proportional to the AST activity, which was measured via the decrease in absorbance. The coefficient of variation was 5% at 1 µkat/L and 3% at 3 µkat/L and the instrument used was the Cobas 6000 .

9. Effects on liver enzymes - ALP (safety parameter).
Change in liver enzyme ASAT (Aspartate transaminase) before and after the 8-week treatment period was examined. ALP was analysed using a colorimetric assay using Cobas 6000 with a CV of 4% at 7 µkat/L.

10. Effects on serum bilirubin (safety parameter).
Serum total bilirubin was measured using a colometric assay on a Cobas system (Roche Diagnostics Scandinavia AB), with a CV of 5% at concentrations 20 and 130 µmol/l.

11. Effects on the blood glucose.
Changes (in percent) in levels of fasting blood glucose and HbA1c before and after the 8 week treatment period. Blood glucose was measured on fresh blood, using Glucose HK (Roche) on a Cobas 6000 instrument. The CV was 3% at concentrations 5 och 15 mmol/L. HbA1c was measured using HPLC (Mono S™, Tricorn™ 50/50 GL (CDP), MonoBeads™ Column (GE Healthcare)). The separated hemoglobin fractions were measured using an UV-detector and absorbance quantified at 417 nm. The CV was 2% at concentrations 42 mmol/mol , 63 mmol/mol and 94 mmol/mol.

12. Effects on abundance of short-chain fatty acids.
Changes in short-chain fatty acids in stool from baseline to week 10 were measured using....

13. Colonization with F. prausnitzii.
Colonization of the intestine with the total amount of F. prausnitzii bacteria, was measured in stool samples, using ....

14. Effect on renal function(safety parameter).
Change in calculated eGFR (Glomerular Filtration Rate, based on serum creatinine) before baseline and after 8 weeks of treatment was investigated. Serum creatinine was measured using CREP2 (Roche/Cobas) on a Cobas 6000 equipment, with a CV of 4% at concentrations 85 and 400 µmol/L.

15. Effect on serum total protein (safety parameter).
Change in serum total protein before baseline and after 8 weeks of treatment was investigated. Total protein was measured using Roche/Cobas on a Cobas 6000 with a CV of 3% at concentrations 50 and 75 g/L.
